# Supplementary material for: NiH-catalysed proximal-selective hydroalkylation of unactivated alkenes and the ligand effects on regioselectivity
Source: Nat Commun. 2022 Apr 7;13:1890. doi: 10.1038/s41467-022-29554-4 (PMC8990077; doi:10.1038/s41467-022-29554-4)
Supplement: Supplementary file 1 — Supplementary Information [file 41467_2022_29554_MOESM1_ESM.pdf]

Supplementary Information

NiH-Catalysed Proximal-Selective Hydroalkylation of Unactivated Alkenes and the Ligand Effects on Regioselectivity

Wang, et al.

Table of Contents

Supplementary Methods S3

General Information S3

Preparation of Alkenes S4

Preparation of Alkyl Halides S13

Proximal-selective Hydroalkylation of Unactivated Alkenes S17

Asymmetric Proximal-selective Hydroalkylation of Unactivated Alkenes S36

Migratory Hydroalkylation of Unactivated Alkenes S40

Synthetic Applications S44

Supplementary Discussions S49

Radical Clock Experiments S49

Deuterium-labelling Experiments S53

Limitations of Substrates in Standard Conditions A..S55

Proposed Mechanism and DFT Calculations..S58

Supplementary Figures S61

Supplementary References S167

Supplementary Methods

General Information

Materials

All the reactions were carried out in oven-dried Schlenk tubes under an argon atmosphere (purity ≥ 99.999%). The following chemicals were purchased and used as received: nickel(II) bromide 2-methoxyethyl ether complex (CAS: 312696-09-6, Aldrich, 459674-5G); bis(triphenylphosphine)nickel(II)chloride (CAS: 14264-16-5, Acros); ethanolamine (CAS: 141-43-5, Sinopharm); trimethoxysilane (CAS: 2487-90-3, Adamas-beta); diethoxymethylsilane (CAS: 2031-62-1, Aladdin); potassium fluoride (CAS: 7789-23-3, Acros, 21350250); sodium carbonate (CAS: 497-19-8, Acros); sodium iodide (CAS: 7681-82-5, Aladdin); *N,N*-dimethylacetamide (CAS: 127-19-5, Adamas-beta). Other commercially available reagents were obtained from Adamas-beta, TCI and Alfa Aesar Chemical Company.

Analytical Methods

^{1}H NMR, ^{13}C NMR, ^{11}B MNR, ^{19}F NMR, and ^{31}P NMR spectra were recorded on a Bruker 400 MHz and 500 MHz spectrometers at 295 K in $CDCl_3$ unless otherwise noted. Data for ^{1}H NMR are reported as follows: chemical shift (δ ppm), multiplicity, integration, and coupling constant (Hz). Data for ^{13}C NMR were reported as follows: chemical shift (δ ppm), multiplicity, and coupling constant (Hz). Data for ^{19}F NMR (^{19}F exp. comp. pulse decoupling, ^{19}F CPD) were reported as follows: chemical shift (δ ppm), multiplicity, coupling constant (Hz). Chemical shifts were reported using the residual solvent $CHCl_3$ as the internal reference for ^{1}H NMR (δ = 7.260 ppm) and $CDCl_3$ peak as the internal reference for ^{13}C NMR (δ = 77.160 ppm). The following abbreviations were used to explain multiplicities: s = singlet, d = doublet, t = triplet, q = quartet, m = multiplet, br = broad. Gas chromatographic (GC) analysis was acquired on a Shimadzu GC-2014 Series GC System equipped with a flame-ionization detector. HPLC analysis was carried out on a Shimadzu system with Daicel columns. HRMS analysis was performed on Finnigan LCQ advantage Max Series MS System and Water XEVO G2 Q-TOF (Waters Corporation). Thin-layer chromatography was performed with silica gel 60 F254 plates eluting with solvents indicated, visualized by a 254 nm UV lamp. Flash chromatography was performed using Silica Gel (300-400 mesh).

Preparation of Alkenes

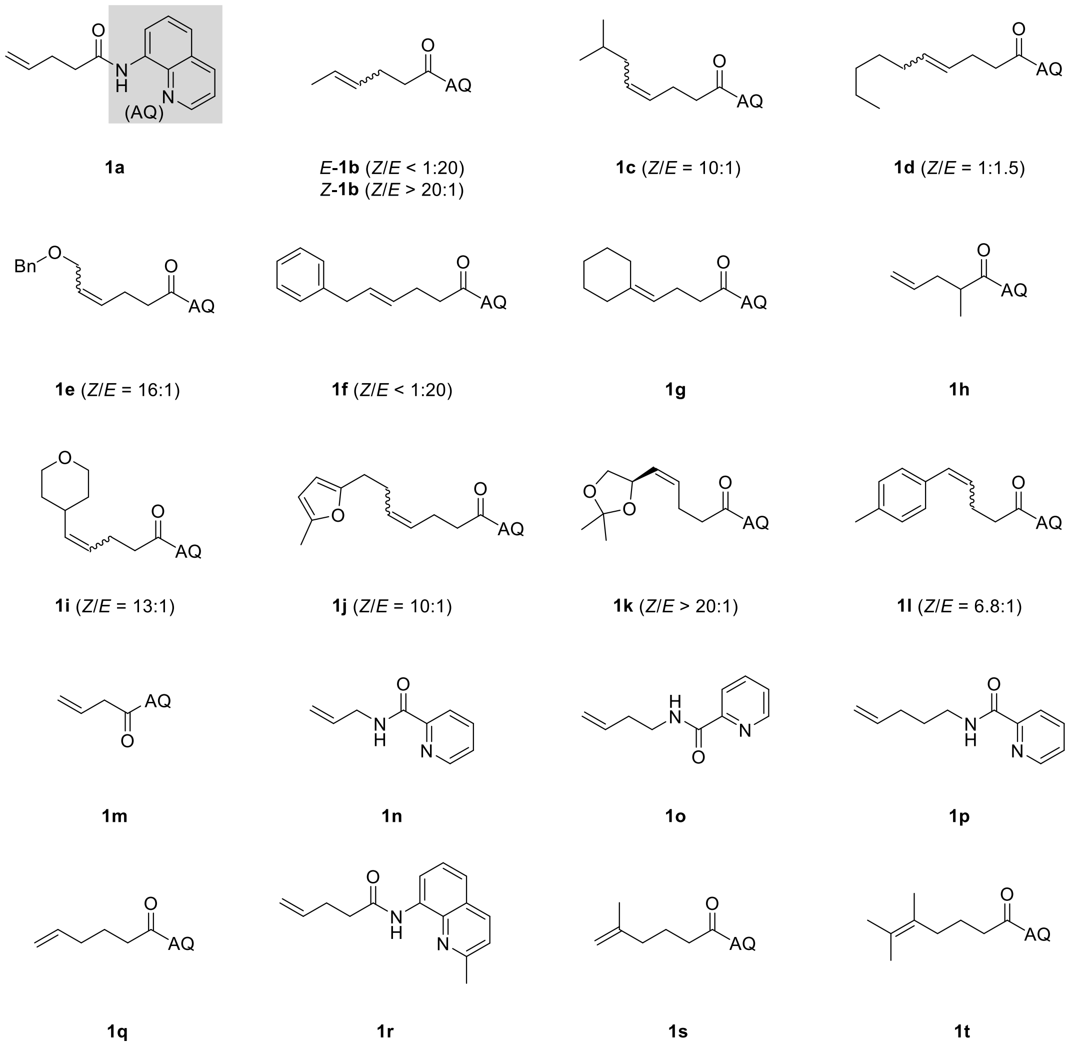

Supplementary Figure 1. List of alkenes

General Procedure for the Alkenes

General procedure A

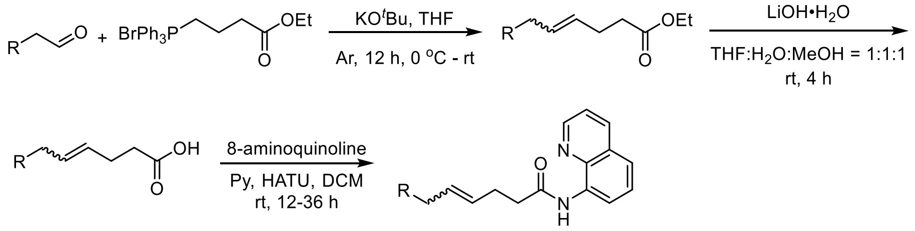

Alkenes were prepared according to the previously reported procedure.[1]

General procedure B

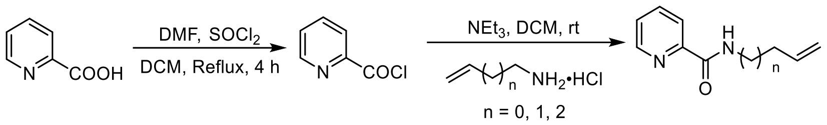

To a solution of picolinic acid (1.0 equiv) in DCM was added DMF (0.1 equiv), and $SOCl_2$ (5.0 equiv). The mixture was stirred at 50 °C for 4 h, and then the solvent was removed in vacuo and dissolved in DCM. The resulting acid chloride solution was used in the next step without further purification.

Another oven-dried round-bottom flask was charged with appropriate allylamine hydrochloride (1.0 equiv), Et_3N (2.0 equiv) in DCM. To this solution was added the acid chloride solution (obtained in the previous step) dropwise at 0 °C. The solution was warmed to room temperature and stirred for 4-8 h. Then the reaction mixture was diluted with saturated sodium chloride solution followed by extraction with EA, dried with anhydrous Na_2SO_4, and concentrated in vacuo. The crude reaction mixture was purified by column chromatography on silica gel to afford the corresponding alkenes **1n-p**.

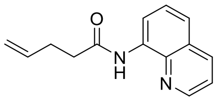

N-(quinoline-8-yl)pent-4-enamide (**1a**)

1a was obtained as white solid from carboxylic acid.

^{1}H NMR (400 MHz, Chloroform-*d*) δ 9.82 (s, 1H), 8.95 – 8.57 (m, 2H), 8.13 (dd, *J* = 8.2, 1.7 Hz, 1H), 7.71 – 7.38 (m, 3H), 5.93 (ddt, *J* = 16.7, 10.2, 6.4 Hz, 1H), 5.15 (dq, *J* = 17.1, 1.6 Hz, 1H), 5.04 (dq, *J* = 10.2, 1.3 Hz, 1H), 2.76 – 2.62 (m, 2H), 2.61 – 2.43 (m, 2H).

^{13}C NMR (101 MHz, Chloroform-*d*) δ 171.0, 148.2, 138.4, 136.9, 136.4, 134.5, 128.0, 127.5, 121.6, 121.5, 116.6, 115.8, 37.4, 29.6.

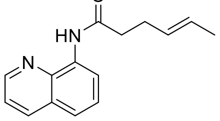

(*E*)-*N*-(quinolin-8-yl)hex-4-enamide (*E*-**1b**)

E-**1b** was obtained as yellow oil from carboxylic acid (*Z*/*E* < 1:20).

^{1}H NMR (400 MHz, Chloroform-*d*) δ 9.82 (s, 1H), 8.78 (dt, *J* = 5.9, 1.6 Hz, 2H), 8.14 (dd, *J* = 8.3, 1.7 Hz, 1H), 7.60 – 7.36 (m, 3H), 5.63 – 5.43 (m, 2H), 2.68 – 2.55 (m, 2H), 2.54 – 2.39 (m, 2H), 1.73 – 1.54 (m, 3H).

^{13}C NMR (101 MHz, Chloroform-*d*) δ 171.3, 148.1, 138.3, 136.4, 134.5, 129.4, 127.9, 127.5, 126.5, 121.6, 121.4, 116.5, 38.1, 28.5, 18.0.

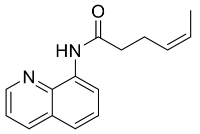

(*Z*)-*N*-(quinolin-8-yl)hex-4-enamide (*Z*-**1b**)

Z-**1b** was obtained as yellow oil from carboxylic acid (*Z*/*E* > 20:1).

^{1}H NMR (500 MHz, Chloroform-*d*) δ 9.82 (s, 1H), 8.83 – 8.72 (m, 2H), 8.14 (dd, *J* = 8.3, 1.7 Hz, 1H), 7.52 (t, *J* = 7.9 Hz, 1H), 7.48 (dd, *J* = 8.2, 1.5 Hz, 1H), 7.43 (dd, *J* = 8.3, 4.2 Hz, 1H), 5.61 – 5.43 (m, 2H), 2.65 – 2.53 (m, 4H), 1.66 (dd, *J* = 6.4, 1.4 Hz, 3H).

^{13}C NMR (126 MHz, Chloroform-*d*) δ 171.4, 148.1, 138.4, 136.5, 134.6, 128.5, 128.0, 127.5, 125.7, 121.6, 121.5, 116.6, 38.0, 23.1, 12.9.

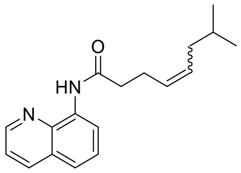

7-methyl-*N*-(quinolin-8-yl)oct-4-enamide (**1c**)

1c was obtained as yellow oil from aldehyde (*Z*/*E* = 10:1).

^{1}H NMR (400 MHz, Chloroform-*d*) δ 9.80 (s, 1H), 8.81 – 8.73 (m, 2H), 8.10 (dd, *J* = 8.3, 1.7 Hz, 1H), 7.52 – 7.47 (m, 1H), 7.45 (dd, *J* = 8.3, 1.6 Hz, 1H), 7.40 (dd, *J* = 8.3, 4.2 Hz, 1H), 5.55 – 5.41 (m, 2H), 2.64 – 2.49 (m, 4H), 2.02 – 1.79 (m, 2H), 1.66 – 1.51 (m, 1H), 0.91 – 0.77 (m, 6H).

^{13}C NMR (101 MHz, Chloroform-*d*) δ 171.3, 148.1, 138.3, 136.4, 134.6, 130.5, 128.2, 128.0, 127.4, 121.6, 121.4, 116.5, 38.1, 36.4, 28.6, 23.6, 22.4.

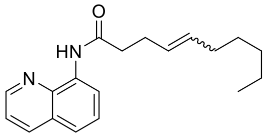

N-(quinolin-8-yl)dec-4-enamide (**1d**)

1d was obtained as yellow oil from carboxylic acid (*Z*/*E* = 1:1.5).

^{1}H NMR (400 MHz, Chloroform-*d*) δ 9.81 (s, 1H), 8.82 – 8.73 (m, 2H), 8.13 (dd, *J* = 8.3, 1.7 Hz, 1H), 7.54 – 7.38 (m, 3H), 5.62 – 5.42 (m, 2H), 2.66 – 2.54 (m, 2H), 2.57 – 2.43 (m, 2H), 2.02 – 1.92 (m, 2H), 1.35 – 1.14 (m, 6H), 0.88 – 0.76 (m, 3H).
^{13}C NMR (101 MHz, Chloroform-*d*) δ 171.4, 148.1, 138.4, 136.5, 134.6, 132.3, 128.1, 128.1, 127.6, 121.6, 121.5, 116.6, 38.4, 32.6, 31.4, 29.2, 28.7, 22.6, 14.1.

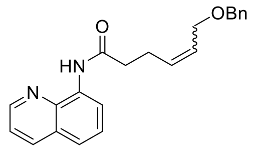

6-(benzyloxy)-*N*-(quinolin-8-yl)hex-4-enamide (**1e**)
1e was obtained as yellow oil from aldehyde (*Z*/*E* = 16:1).
^{1}H NMR (400 MHz, Chloroform-*d*) δ 9.79 (s, 1H), 8.77 (dq, *J* = 6.4, 1.9 Hz, 2H), 8.12 (dd, *J* = 8.3, 1.7 Hz, 1H), 7.55 – 7.47 (m, 1H), 7.47 (dd, *J* = 8.3, 1.7 Hz, 1H), 7.41 (dd, *J* = 8.3, 4.2 Hz, 1H), 7.37 – 7.25 (m, 4H), 7.31 – 7.19 (m, 1H), 5.78 – 5.62 (m, 2H), 4.50 (s, 1.88H), 4.45 (s, 0.13H), 4.15 (d, *J* = 4.8 Hz, 1.87H), 3.97 (dd, *J* = 5.8, 1.1 Hz, 0.14H), 2.70 – 2.50 (m, 4H).
^{13}C NMR (126 MHz, Chloroform-*d*) δ 170.8, 148.2, 138.4, 138.3, 136.5, 134.5, 131.4, 128.4, 128.0, 127.9, 127.9, 127.6, 127.5, 121.7, 121.5, 116.5, 72.3, 65.8, 37.8, 23.8.

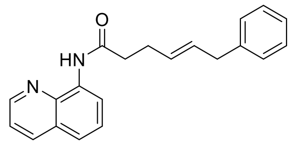

(*E*)-6-phenyl-*N*-(quinolin-8-yl)hex-4-enamide (**1f**)
1f was obtained as yellow oil from aldehyde (*Z*/*E* < 1:20).
^{1}H NMR (400 MHz, Chloroform-*d*) δ 9.83 (s, 1H), 8.83 – 8.75 (m, 2H), 8.15 (dd, *J* = 8.3, 1.7 Hz, 1H), 7.57 – 7.39 (m, 3H), 7.28 – 7.22 (m, 2H), 7.21 – 7.13 (m, 3H), 5.71 – 5.57 (m, 2H), 3.47 (d, *J* = 6.6 Hz, 2H), 2.75 – 2.61 (m, 4H).
^{13}C NMR (101 MHz, Chloroform-*d*) δ 171.1, 148.2, 140.9, 138.4, 136.5, 134.6, 130.0, 128.6, 128.5, 128.5, 128.0, 127.5, 126.0, 121.7, 121.5, 116.6, 38.0, 33.6, 23.5.

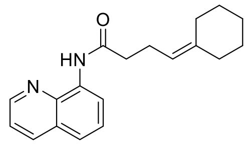

4-cyclohexylidene-*N*-(quinolin-8-yl)butanamide (**1g**)
1g was obtained as yellow oil from aldehyde.

^{1}H NMR (400 MHz, Chloroform-*d*) δ 9.81 (s, 1H), 8.84 – 8.76 (m, 2H), 8.15 (dd, *J* = 8.3, 1.7 Hz, 1H), 7.63 – 7.37 (m, 3H), 5.18 (tt, *J* = 6.9, 1.3 Hz, 1H), 2.62 – 2.55 (m, 2H), 2.55 – 2.46 (m, 2H), 2.21 – 2.15 (m, 2H), 2.10 – 2.04 (m, 2H), 1.55 – 1.38 (m, 6H).

^{13}C NMR (101 MHz, Chloroform-*d*) δ 171.6, 148.2, 141.7, 138.4, 136.5, 134.7, 128.0, 127.6, 121.6, 121.4, 119.2, 116.6, 38.9, 37.2, 28.8, 28.7, 27.9, 27.0, 23.5.

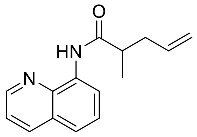

2-methyl-*N*-(quinolin-8-yl)pent-4-enamide (**1h**)

1h was obtained as yellow oil from carboxylic acid.

^{1}H NMR (400 MHz, Chloroform-*d*) δ 9.88 (s, 1H), 8.84 – 8.76 (m, 2H), 8.13 (dd, *J* = 8.3, 1.7 Hz, 1H), 7.52 (dd, *J* = 8.3, 7.4 Hz, 1H), 7.47 (dd, *J* = 8.3, 1.6 Hz, 1H), 7.43 (dd, *J* = 8.2, 4.2 Hz, 1H), 5.86 (ddt, *J* = 17.2, 10.1, 7.1 Hz, 1H), 5.14 (dq, *J* = 17.1, 1.5 Hz, 1H), 5.04 (ddt, *J* = 10.2, 2.0, 1.1 Hz, 1H), 2.74 – 2.65 (m, 1H), 2.65 – 2.53 (m, 1H), 2.38 – 2.26 (m, 1H), 1.34 (d, *J* = 6.8 Hz, 3H).

^{13}C NMR (101 MHz, Chloroform-*d*) δ 174.6, 148.2, 138.5, 136.5, 135.7, 134.6, 128.0, 127.5, 121.6, 121.5, 117.2, 116.6, 42.7, 38.5, 17.6.

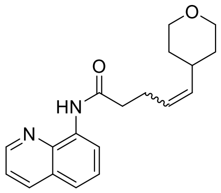

N-(quinolin-8-yl)-5-(tetrahydro-2*H*-pyran-4-yl)pent-4-enamide (**1i**)

1i was obtained as yellow oil from aldehyde (*Z*/*E* = 13:1).

^{1}H NMR (500 MHz, Chloroform-*d*) δ 9.85 (s, 1H), 8.95 – 8.58 (m, 2H), 8.18 (dd, *J* = 8.3, 1.7 Hz, 1H), 7.62 – 7.37 (m, 3H), 5.42 (dt, *J* = 10.9, 6.9 Hz, 1H), 5.35 – 5.18 (m, 1H), 3.98 – 3.79 (m, 2H), 3.39 (td, *J* = 11.6, 2.3 Hz, 2H), 2.75 – 2.42 (m, 5H), 1.55 – 1.34 (m, 4H).

^{13}C NMR (101 MHz, Chloroform-*d*) δ 171.1, 148.2, 138.3, 136.6, 135.9, 134.5, 128.0, 127.6, 126.9, 121.7, 121.6, 116.6, 67.7, 38.3, 33.6, 32.9, 23.8.

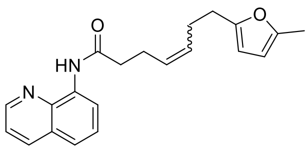

7-(5-methylfuran-2-yl)-*N*-(quinolin-8-yl)hept-4-enamide (**1j**)

1j was obtained as yellow oil from aldehyde (*Z*/*E* = 10:1).

^{1}H NMR (400 MHz, Chloroform-*d*) δ 9.80 (s, 1H), 8.83 – 8.74 (m, 2H), 8.13 (dd, *J* = 8.3, 1.7 Hz, 1H), 7.55 – 7.49 (m, 1H), 7.47 (dd, *J* = 8.3, 1.6 Hz, 1H), 7.42 (dd, *J* = 8.3, 4.2 Hz, 1H), 5.88 – 5.76 (m, 2H), 5.63 – 5.48 (m, 2H), 2.68 – 2.60 (m, 2H), 2.57 – 2.51 (m, 4H), 2.47 – 2.40 (m, 2H), 2.26 – 2.21 (m, 3H).

^{13}C NMR (101 MHz, Chloroform-*d*) δ 171.2, 153.8, 150.3, 148.1, 138.3, 136.4, 134.6, 130.2, 128.6, 128.0, 127.5, 121.6, 121.4, 116.5, 105.9, 105.8, 38.0, 28.1, 26.1, 23.4, 13.6.

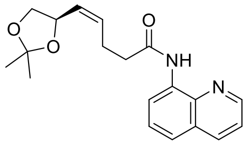

(*R*,*Z*)-5-(2,2-dimethyl-1,3-dioxolan-4-yl)-*N*-(quinolin-8-yl)pent-4-enamide (**1k**)

1k was obtained as yellow oil from aldehyde (*Z*/*E* > 20:1).

^{1}H NMR (400 MHz, Chloroform-*d*) δ 9.80 (s, 1H), 8.79 (dd, *J* = 4.2, 1.7 Hz, 1H), 8.77 (dd, *J* = 7.2, 1.8 Hz, 1H), 8.16 (dd, *J* = 8.3, 1.7 Hz, 1H), 7.56 – 7.48 (m, 2H), 7.45 (dd, *J* = 8.3, 4.2 Hz, 1H), 5.77 – 5.67 (m, 1H), 5.54 – 5.45 (m, 1H), 4.99 – 4.89 (m, 1H), 4.12 – 4.04 (m, 1H), 3.57 – 3.45 (m, 1H), 2.78 – 2.43 (m, 4H), 1.41 (s, 3H), 1.38 (s, 3H).

^{13}C NMR (126 MHz, Chloroform-*d*) δ 170.6, 148.2, 138.3, 136.5, 134.4, 132.7, 129.0, 128.0, 127.5, 121.7, 121.6, 116.6, 109.2, 71.9, 69.5, 37.8, 26.9, 26.0, 23.9.

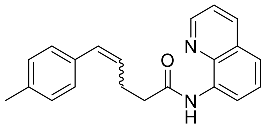

N-(quinolin-8-yl)-5-(*p*-tolyl)pent-4-enamide (**1l**)

1l was obtained as colorless oil from aldehyde (*Z*/*E* = 6.8:1).

^{1}H NMR (400 MHz, Chloroform-*d*) δ 9.85 (d, *J* = 14.3 Hz, 1H), 8.84 – 8.80 (m, 1H), 8.75 – 8.72 (m, 1H), 8.13 – 8.04 (m, 1H), 7.55 – 7.41 (m, 2H), 7.43 – 7.34 (m, 1H), 7.27 – 7.20 (m, 2H), 7.17 – 7.05 (m, 2H), 6.54 – 6.44 (m, 1H), 6.33 – 6.21 (m, 0.13H),

5.71 (dt, *J* = 11.6, 7.1 Hz, 0.89H), 2.93 – 2.83 (m, 1.78H), 2.75 – 2.63 (m, 2.24H), 2.36 – 2.29 (m, 3H).

^{13}C NMR (101 MHz, Chloroform-*d*) δ 170.8, 148.0, 138.2, 136.4, 136.3, 134.4, 134.3, 130.1, 129.7, 128.9, 128.7, 127.9, 127.3, 121.5, 121.4, 116.4, 38.0, 24.7, 21.2.

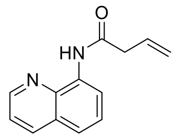

N-(quinolin-8-yl)but-3-enamide (**1m**)

1m was obtained as yellow oil from carboxylic acid.

^{1}H NMR (400 MHz, Chloroform-*d*) δ 9.96 (s, 1H), 8.83 – 8.73 (m, 2H), 8.14 (dd, *J* = 8.3, 1.7 Hz, 1H), 7.57 – 7.45 (m, 2H), 7.43 (dd, *J* = 8.3, 4.2 Hz, 1H), 6.15 (ddt, *J* = 17.2, 10.1, 7.1 Hz, 1H), 5.44 – 5.33 (m, 2H), 3.35 (dt, *J* = 7.1, 1.3 Hz, 2H).

^{13}C NMR (101 MHz, Chloroform-*d*) δ 169.3, 148.3, 138.5, 136.4, 134.4, 131.1, 128.0, 127.4, 121.7, 121.7, 120.1, 116.5, 43.3.

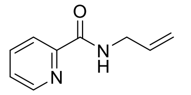

N-allylpicolinamide (**1n**)

1n was obtained as yellow solid.

^{1}H NMR (400 MHz, Chloroform-*d*) δ 8.57 – 8.52 (m, 1H), 8.23 – 8.17 (m, 2H), 7.85 (td, *J* = 7.7, 1.8 Hz, 1H), 7.43 (ddd, *J* = 7.6, 4.8, 1.3 Hz, 1H), 5.94 (ddt, *J* = 17.2, 10.7, 5.6 Hz, 1H), 5.27 (dq, *J* = 17.2, 1.7 Hz, 1H), 5.17 (dq, *J* = 10.2, 1.5 Hz, 1H), 4.10 (tt, *J* = 5.8, 1.7 Hz, 2H).

^{13}C NMR (126 MHz, Chloroform-*d*) δ 164.1, 149.8, 148.0, 137.5, 134.0, 126.2, 122.4, 116.5, 41.8.

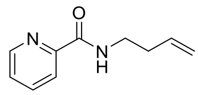

N-(but-3-en-1-yl)picolinamide (**1o**)

1o was obtained as yellow oil.

^{1}H NMR (400 MHz, Chloroform-*d*) δ 8.53 (ddd, *J* = 4.8, 1.8, 0.9 Hz, 1H), 8.18 (dt, *J* = 7.8, 1.1 Hz, 1H), 8.12 (s, 1H), 7.83 (td, *J* = 7.7, 1.7 Hz, 1H), 7.40 (ddd, *J* = 7.6, 4.8,

1.3 Hz, 1H), 6.18 – 5.63 (m, 1H), 5.14 (dq, *J* = 17.2, 1.6 Hz, 1H), 5.09 (ddt, *J* = 10.2, 2.1, 1.2 Hz, 1H), 3.54 (td, *J* = 6.9, 6.0 Hz, 2H), 2.39 (qt, *J* = 6.9, 1.4 Hz, 2H).

^{13}C NMR (126 MHz, Chloroform-*d*) δ 164.4, 150.1, 148.2, 137.4, 135.3, 126.2, 122.3, 117.3, 38.6, 33.9.

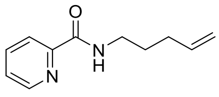

N-(pent-4-en-1-yl)picolinamide (**1p**)

1p was obtained as yellow oil.

^{1}H NMR (400 MHz, Chloroform-*d*) δ 8.52 (ddd, *J* = 4.8, 1.7, 0.9 Hz, 1H), 8.18 (dt, *J* = 7.8, 1.1 Hz, 1H), 8.09 (s, 1H), 7.82 (td, *J* = 7.7, 1.7 Hz, 1H), 7.40 (ddd, *J* = 7.6, 4.8, 1.2 Hz, 1H), 5.82 (ddt, *J* = 16.9, 10.2, 6.6 Hz, 1H), 5.04 (dq, *J* = 17.1, 1.7 Hz, 1H), 4.97 (ddt, *J* = 10.2, 2.2, 1.3 Hz, 1H), 3.47 (td, *J* = 7.2, 6.2 Hz, 2H), 2.20 – 2.10 (m, 2H), 1.78 – 1.67 (m, 2H).

^{13}C NMR (101 MHz, Chloroform-*d*) δ 164.3, 150.1, 148.1, 137.8, 137.5, 126.2, 122.3, 115.3, 39.0, 31.2, 28.9.

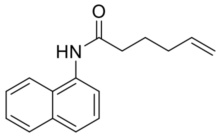

N-(naphthalen-1-yl)hex-5-enamide (**1q**)

1q was obtained as yellow oil from carboxylic acid.

^{1}H NMR (400 MHz, Chloroform-*d*) δ 9.80 (s, 1H), 8.82 – 8.75 (m, 2H), 8.14 (dd, *J* = 8.3, 1.7 Hz, 1H), 7.52 (dd, *J* = 8.3, 7.3 Hz, 1H), 7.48 (dd, *J* = 8.3, 1.7 Hz, 1H), 7.43 (dd, *J* = 8.3, 4.2 Hz, 1H), 5.84 (ddt, *J* = 16.9, 10.1, 6.7 Hz, 1H), 5.08 (dq, *J* = 17.2, 1.7 Hz, 1H), 5.02 (ddt, *J* = 10.2, 2.2, 1.2 Hz, 1H), 2.61 – 2.53 (m, 2H), 2.25 – 2.15 (m, 2H), 1.98 – 1.88 (m, 2H).

^{13}C NMR (126 MHz, Chloroform-*d*) δ 171.7, 148.2, 138.4, 137.9, 136.5, 134.6, 128.0, 127.5, 121.7, 121.5, 116.5, 115.6, 37.4, 33.3, 24.8.

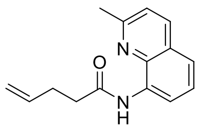

N-(2-methylquinolin-8-yl)pent-4-enamide (**1r**)

1r was obtained as yellow oil from carboxylic acid.

^{1}H NMR (400 MHz, Chloroform-*d*) δ 9.72 (s, 1H), 8.62 – 8.56 (m, 1H), 7.82 (dd, *J* = 8.4, 2.4 Hz, 1H), 7.28 – 7.24 (m, 2H), 7.11 (dd, *J* = 8.4, 2.4 Hz, 1H), 5.84 – 5.72 (m, 1H), 5.02 (d, *J* = 17.1 Hz, 1H), 4.90 (d, *J* = 10.3 Hz, 1H), 2.57 (s, 3H), 2.55 – 2.47 (m, 2H), 2.45 – 2.39 (m, 2H).
^{13}C NMR (126 MHz, Chloroform-*d*) δ 170.9, 157.2, 137.7, 137.1, 136.5, 133.9, 126.4, 126.1, 122.5, 121.3, 116.5, 115.8, 37.4, 29.5, 25.3.

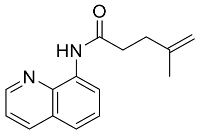

4-methyl-*N*-(quinolin-8-yl)pent-4-enamide (**1s**)
1s was obtained as white solid from carboxylic acid.
^{1}H NMR (500 MHz, Chloroform-*d*) δ 9.86 (s, 1H), 8.83 – 8.76 (m, 2H), 8.17 (dd, *J* = 8.3, 1.8 Hz, 1H), 7.54 (t, *J* = 7.9 Hz, 1H), 7.50 (dd, *J* = 8.3, 1.6 Hz, 1H), 7.46 (dd, *J* = 8.2, 4.3 Hz, 1H), 4.80 (s, 2H), 2.75 – 2.70 (m, 2H), 2.57 – 2.51 (m, 2H), 1.82 (s, 3H).
^{13}C NMR (126 MHz, Chloroform-*d*) δ 171.4, 148.1, 144.4, 138.3, 136.7, 134.6, 128.1, 127.6, 121.7, 121.5, 116.8, 110.7, 36.5, 33.3, 22.8.

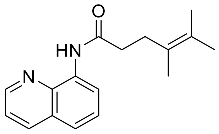

4,5-dimethyl-*N*-(quinolin-8-yl)hex-4-enamide (**1t**)
According to the previously reported procedure,[2] **1t** was obtained as yellow oil.
^{1}H NMR (400 MHz, Chloroform-*d*) δ 9.79 (s, 1H), 8.82 – 8.74 (m, 2H), 8.12 (dq, *J* = 8.3, 1.5 Hz, 1H), 7.51 (t, *J* = 7.9 Hz, 1H), 7.47 – 7.44 (m, 1H), 7.43 – 7.39 (m, 1H), 2.64 – 2.50 (m, 4H), 1.74 – 1.68 (m, 6H), 1.64 (s, 3H).
^{13}C NMR (126 MHz, Chloroform-*d*) δ 171.8, 148.1, 138.4, 136.4, 134.7, 128.0, 127.5, 126.1, 125.9, 121.6, 121.4, 116.5, 36.9, 30.5, 20.8, 20.3, 18.3.

Preparation of Alkyl Halides

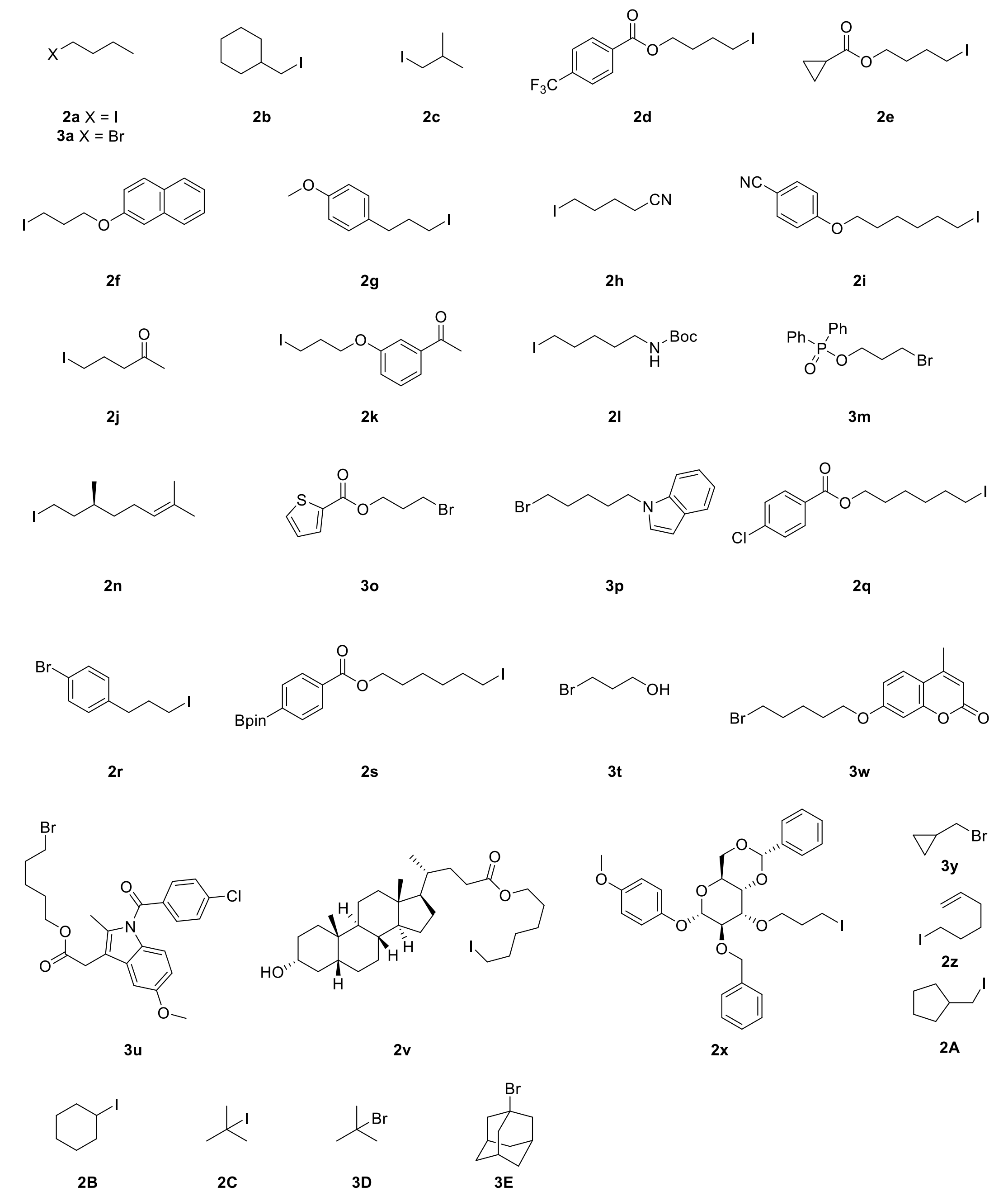

Supplementary Figure 2. List of alkyl halides

General Procedure for the Preparation of Alkyl Halides

General procedure C

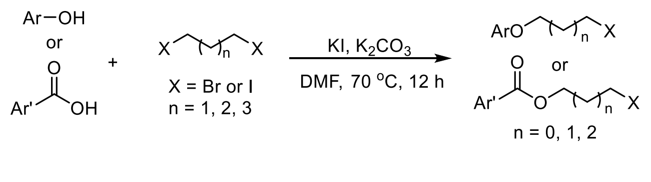

According to the previously reported procedure.[3] To a stirred suspension of phenol or benzoic acid (1.0 equiv) in DMF, K_2CO_3 (2.0 equiv) was added at room temperature (if dibromoalkane was used, 0.1 equiv KI was added at this time). The reaction was stirred for additional 0.5 hours at room temperature, dibromoalkanes or diiodoalkanes were added. The mixture was stirred at 70 °C for 12 h, extracted with EA, washed with saturated sodium chloride solution, dried with Na_2SO_4, filtered, and concentrated under reduced pressure. The resulting mixture was isolated by flash chromatography.

General procedure D

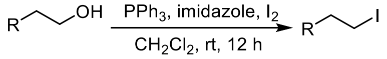

According to the previously reported procedure.[4] To a stirred solution of PPh_3 (1.2 equiv) and imidazole (1.2 equiv) in DCM were added I_2 (1.3 equiv) at 0 °C. Alcohols (1.0 equiv) were added dropwise and stirred for 12 h at room temperature. The mixture was washed with a saturated sodium sulfite solution, dried with Na_2SO_4, filtered, and concentrated under reduced pressure. The resulting mixture was isolated by flash chromatography.

Alkyl halides **2a-c**, **3a**, **3t**, **3y, 2B, 2C, 3D, 3E** were commercially available. Alkyl halides **2d, 2f**, **2i**, **2k**, **2q**, **2s**, **2v**, **2o**, **3p**, **3u** were synthesized from corresponding acid or phenol (General procedure C). Alkyl halides **2e, 2g**, **2j**, **2l**, **2n**, **2r**, **2z**, **3m**, **2A** were synthesized from corresponding alkyl alcohol (General procedure D).

The ^{1}H NMR data of alkyl halides **2f**,[5] **2g**,[6] **2h**,[7] **2i**,[8] **2j**,[9] **2l**,[6] **2n**,[10] **3o**,[11] **3p**,[12] **2r**,[13] **3w**,[14] and **2z**[15] were consistent with previous reports.

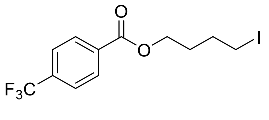

4-iodobutyl 4-(trifluoromethyl)benzoate (**2d**)

^{1}H NMR (400 MHz, Chloroform-*d*) δ 8.14 (d, J = 8.1 Hz, 2H), 7.70 (d, J = 8.2 Hz, 2H), 4.38 (t, J = 6.2 Hz, 2H), 3.25 (t, J = 6.6 Hz, 2H), 2.04 – 1.95 (m, 2H), 1.95 – 1.87 (m, 2H).

^{19}F NMR (376 MHz, Chloroform-*d*) δ -63.10.

^{13}C NMR (101 MHz, Chloroform-*d*) δ 165.4, 134.6 (q, J = 32.7 Hz), 133.5, 130.1, 125.5 (q, J = 3.8 Hz), 123.7 (q, J = 272.6 Hz), 64.5, 30.1, 29.7, 5.9.

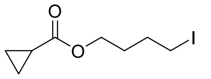

4-iodobutyl cyclopropanecarboxylate (**2e**)

^{1}H NMR (400 MHz, Chloroform-*d*) δ 4.07 (t, *J* = 6.4 Hz, 2H), 3.20 (t, *J* = 6.9 Hz, 2H), 1.95 – 1.83 (m, 2H), 1.79 – 1.68 (m, 2H), 1.62 – 1.52 (m, 1H), 1.00 – 0.93 (m, 2H), 0.87 – 0.81 (m, 2H).

^{13}C NMR (101 MHz, Chloroform-*d*) δ 174.9, 63.3, 30.1, 29.7, 12.9, 8.5, 6.1.

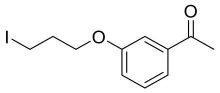

1-(3-(3-iodopropoxy)phenyl)ethan-1-one (**2k**)

^{1}H NMR (400 MHz, Chloroform-*d*) δ 7.54 (ddd, *J* = 7.7, 1.6, 1.0 Hz, 1H), 7.48 (dd, *J* = 2.6, 1.5 Hz, 1H), 7.37 (dd, *J* = 8.2, 7.6 Hz, 1H), 7.11 (ddd, *J* = 8.2, 2.7, 1.0 Hz, 1H), 4.09 (t, *J* = 5.8 Hz, 2H), 3.37 (t, *J* = 6.7 Hz, 2H), 2.59 (s, 3H), 2.28 (tt, *J* = 6.7, 5.7 Hz, 2H).

^{13}C NMR (101 MHz, Chloroform-*d*) δ 197.9, 158.9, 138.5, 129.7, 121.4, 120.0, 113.2, 67.5, 32.9, 26.8, 2.4.

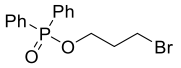

3-bromopropyl diphenylphosphinate (**3m**)

^{1}H NMR (400 MHz, Chloroform-*d*) δ 7.84 – 7.75 (m, 4H), 7.56 – 7.47 (m, 2H), 7.47 – 7.40 (m, 4H), 4.15 (dt, *J* = 7.2, 5.8 Hz, 2H), 3.53 (t, *J* = 6.5 Hz, 2H), 2.23 (p, *J* = 6.2 Hz, 2H).

^{13}C NMR (101 MHz, Chloroform-*d*) δ 132.4 (d, *J* = 2.9 Hz), 131.7 (d, *J* = 10.2 Hz), 131.1 (d, *J* = 137.0 Hz), 128.7 (d, *J* = 13.1 Hz), 62.6 (d, *J* = 5.7 Hz), 33.5 (d, *J* = 6.5 Hz), 29.4.

^{31}P NMR (162 MHz, Chloroform-*d*) δ 32.12.

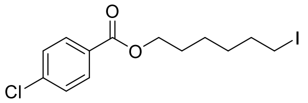

6-iodohexyl 4-chlorobenzoate (**2q**)

^{1}H NMR (500 MHz, Chloroform-*d*) δ 7.97 (d, *J* = 8.4 Hz, 2H), 7.41 (d, *J* = 8.4 Hz, 2H), 4.31 (t, *J* = 6.6 Hz, 2H), 3.19 (t, *J* = 7.0 Hz, 2H), 1.85 (p, *J* = 7.0 Hz, 2H), 1.78 (p, *J* = 6.7 Hz, 2H), 1.49 – 1.44 (m, 4H).

^{13}C NMR (101 MHz, Chloroform-*d*) δ 165.9, 139.4, 131.0, 129.0, 128.8, 65.2, 33.4, 30.2, 28.6, 25.1, 6.9.

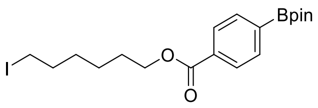

6-iodohexyl 4-(4,4,5,5-tetramethyl-1,3,2-dioxaborolan-2-yl)benzoate (**2s**)

^{1}H NMR (400 MHz, Chloroform-*d*) δ 8.00 (d, *J* = 8.2 Hz, 2H), 7.85 (d, *J* = 8.2 Hz, 2H), 4.30 (t, *J* = 6.6 Hz, 2H), 3.16 (t, *J* = 6.9 Hz, 2H), 1.88 – 1.72 (m, 4H), 1.45 (p, *J* = 3.6 Hz, 4H), 1.33 (s, 12H).

^{13}C NMR (101 MHz, Chloroform-*d*) δ 166.6, 134.7, 132.6, 128.6, 84.2, 64.9, 33.3, 30.2, 28.5, 25.1, 24.9, 6.9.

^{11}B NMR (128 MHz, Chloroform-*d*) δ 30.31.

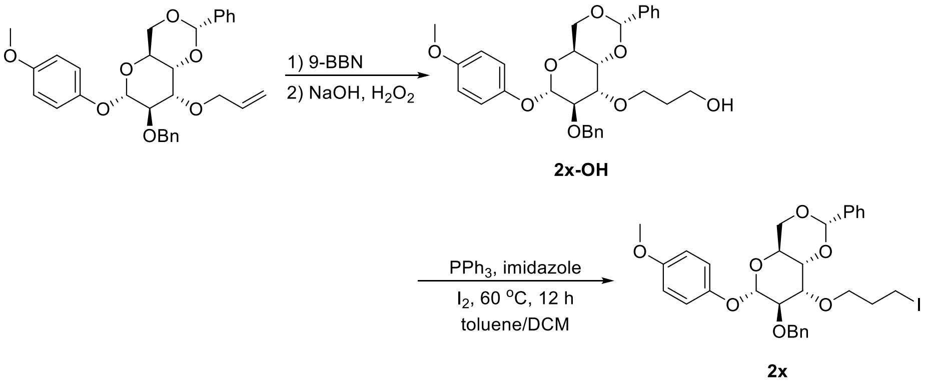

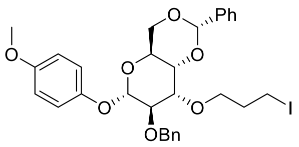

(2*S*,4a*S*,6*S*,7*R*,8*S*,8a*S*)-7-(benzyloxy)-8-(3-iodopropoxy)-6-(4-methoxyphenoxy)-2-phenylhexahydropyrano[3,2-*d*][1,3]dioxine (**2x**)

According to the previously reported procedure.[16] **2x-OH** was obtained as white solid. To a stirred solution of triphenylphosphine (2.6 equiv) and imidazole (2.8 equiv) in dichloromethane was added iodine (2.8 equiv) at 0 °C. Stirring was continued for 5-10 min, the solution of **2x-OH** in toluene/dichloromethane was added. After complete conversion of **2x-OH** (as indicated by TLC), the reaction was quenched with an aqueous solution of sodium sulfite. The mixture was extracted with EA, dried with anhydrous Na_2SO_4, and concentrated in vacuo. The residue was purified by column chromatography to afford the target product as white solid.

^{1}H NMR (400 MHz, Chloroform-*d*) δ 7.64 – 7.45 (m, 2H), 7.43 – 7.27 (m, 8H), 7.11 – 6.96 (m, 2H), 6.89 – 6.65 (m, 2H), 5.61 (s, 1H), 4.96 (d, *J* = 10.9 Hz, 1H), 4.90 (d, *J* = 7.8 Hz, 1H), 4.80 (d, *J* = 10.9 Hz, 1H), 4.42 – 4.32 (m, 2H), 4.11 (dd, *J* = 12.3, 1.9 Hz, 1H), 3.98 (dd, *J* = 9.7, 7.8 Hz, 1H), 3.83 – 3.65 (m, 5H), 3.53 (dd, *J* = 9.7, 3.6 Hz, 1H), 3.49 (s, 1H), 3.41 – 3.21 (m, 2H), 2.17 – 1.94 (m, 2H).
^{13}C NMR (101 MHz, Chloroform-*d*) δ 155.5, 151.8, 138.8, 137.9, 129.1, 128.5, 128.3, 128.1, 127.8, 126.7, 119.2, 114.6, 103.4, 101.5, 80.5, 77.9, 75.5, 73.3, 69.4, 69.1, 66.7, 55.8, 33.6, 4.5.

Proximal-selective Hydroalkylation of Unactivated Alkenes

Supplementary Table 1. Optimization of the Proximal-selective Hydroalkylation of Unactivated Alkenes

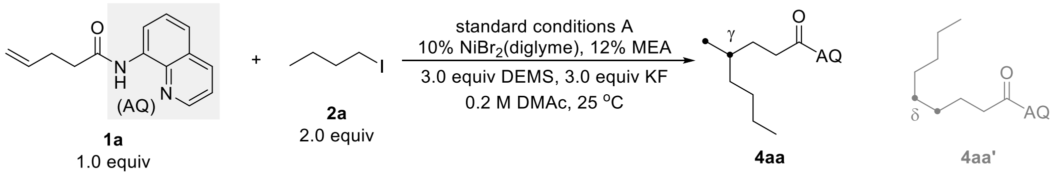

entry	Ni source	silane	base	solvent	Yield/%[a]	r.r.[b]
1	$NiBr_2$(diglyme)	DEMS	KF	DMAc	90 (87[c])	> 20:1
2	w/o $NiBr_2$(diglyme)	DEMS	KF	DMAc	N.R.	-
3	$NiBr_2$(DME)	DEMS	KF	DMAc	85	18:1
4	$Ni(acac)_2$	DEMS	KF	DMAc	79	14:1
5	$NiCl_2(PPh_3)_2$	DEMS	KF	DMAc	48	1.1:1[d]
6	$NiBr_2$(diglyme)	$(MeO)_3SiH$	KF	DMAc	80	5.4:1
7	$NiBr_2$(diglyme)	PMHS	KF	DMAc	77	18:1
8	$NiBr_2$(diglyme)	Ph_2SiH_2	KF	DMAc	72	>20:1
9	$NiBr_2$(diglyme)	$MeEt_2SiH$	KF	DMAc	N.R.	-
10	$NiBr_2$(diglyme)	DEMS	Na_2CO_3	DMAc	80	3.5:1
11	$NiBr_2$(diglyme)	DEMS	CsF	DMAc	34	>20:1
12	$NiBr_2$(diglyme)	DEMS	$NaHCO_3$	DMAc	57	6.2:1
13	$NiBr_2$(diglyme)	DEMS	$K_3PO_4(H_2O)$	DMAc	87	7.0:1
14	$NiBr_2$(diglyme)	DEMS	KF	DMF	81	17:1
15	$NiBr_2$(diglyme)	DEMS	KF	MeCN	25	12:1

16	$NiBr_2$(diglyme)	DEMS	KF	DCE	33	10:1
17	$NiBr_2$(diglyme)	DEMS	KF	toluene	20	8.1:1
18	$NiBr_2$(diglyme)	DEMS	KF	THF	12	1.0:1

Reactions were carried out under an argon atmosphere. Conditions: **1a** (0.10 mmol, 1.0 equiv), **2a** (0.20 mmol, 2.0 equiv), nickel catalyst (0.01 mmol, 10 mol%), MEA (0.012 mmol, 12 mol%), silane (0.30 mmol, 3.0 equiv), base (0.30 mmol, 3.0 equiv), solvent (0.50 mL, 0.2 M), 12 h. [a]Yields and regioisomeric ratios were determined by GC analysis with triphenylmethane as an internal standard. Total yield for the mixture of all regioisomers. [b]r.r. refers to the regioisomeric ratio, that of the major product to the sum of all other isomers. Proximal-selective hydroalkylation product **4aa** was obtained as the major regioisomer, and distal-selective hydroalkylation product **4aa’** as the minor regioisomer; other regioisomers could hardly be detected. [c]Isolated yield in parentheses. [d]A large amount of β-selective product was observed by GC analysis. DEMS, diethoxymethylsilane. PMHS, polymethylhydrosiloxane. DMAc, *N*,*N*-dimethylacetamide. DMF, *N*,*N*-dimethylformamide. DME, 1,2-dimethoxyethane. DCE, 1,2-dichloroethane. THF, tetrahydrofuran. acac, acetylacetanoate. w/o, without. N.R., no reaction.

In air, a 10 mL Schlenk tube equipped with a stir bar was charged with nickel catalyst (0.01 mmol, 10 mol%), base (0.30 mmol, 3.0 equiv), alkene (0.10 mmol, 1.0 equiv). The Schlenk tube was evacuated and filled with argon (three cycles). To these solids, a solution of ethanolamine (0.012 mmol, 12 mol%) in solvent (0.50 mL) was added under an argon atmosphere. Then, alkyl halide (0.20 mmol, 2.0 equiv), silane (0.30 mmol, 3.0 equiv) were added under an argon atmosphere. The mixture was stirred at 25 °C for 12 h. The GC yield was determined using triphenylmethane as an internal standard.

Effect of nitrogen-containing ligands

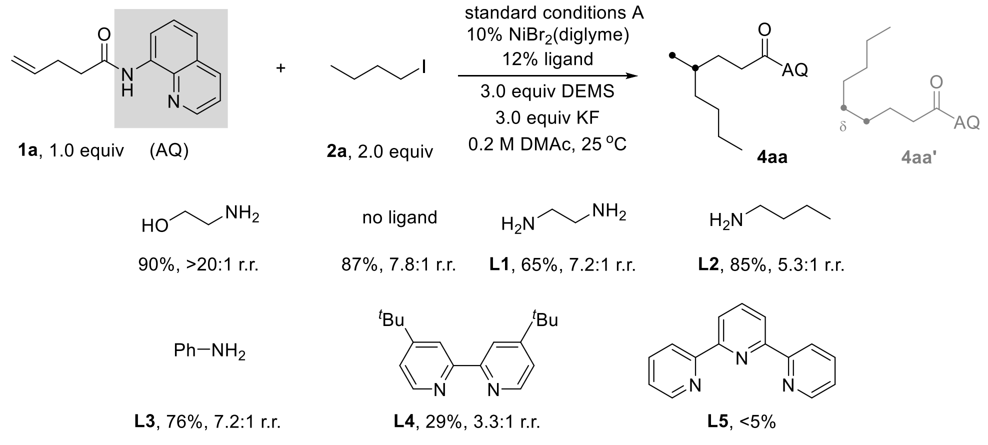

Supplementary Figure 3. Reactions were carried out under an argon atmosphere. Conditions: **1a** (0.10 mmol, 1.0 equiv), **2a** (0.20 mmol, 2.0 equiv), $NiBr_2$(diglyme) (0.01 mmol, 10 mol%), ligand (0.012 mmol, 12 mol%), DEMS (0.30 mmol, 3.0 equiv), KF (0.30 mmol, 3.0 equiv), DMAc (0.50 mL, 0.2 M), 12 h. Yields and regioisomeric ratios

were determined by GC analysis with triphenylmethane as an internal standard. Total yield for the mixture of all regioisomers. r.r. refers to the regioisomeric ratio, that of the major product to the sum of all other isomers. Proximal-selective hydroalkylation product **4aa** was obtained as the major regioisomer, and distal-selective hydroalkylation product **4aa’** as the minor regioisomer; other regioisomers could hardly be detected. DEMS = diethoxymethylsilane. Diglyme = 2-methoxyethyl ether. DMAc = *N*,*N*-dimethylacetamide.

In air, a 10 mL Schlenk tube equipped with a stir bar was charged with $NiBr_2$(diglyme) (0.01 mmol, 10 mol%), ligand (0.012 mmol, 12 mol%), KF (0.30 mmol, 3.0 equiv), alkene (0.10 mmol, 1.0 equiv). The Schlenk tube was evacuated and filled with argon (three cycles). To these solids, DMAc (0.50 mL) was added under an argon atmosphere. Then, alkyl halide (0.20 mmol, 2.0 equiv), DEMS (0.30 mmol, 3.0 equiv) were added under an argon atmosphere. The mixture was stirred at 25 °C for 12 h. The GC yield was determined using triphenylmethane as an internal standard.

Determination of Regioisomeric Ratio in Standard Conditions A

For terminal alkene **1a** under standard conditions A, we obtained γ-selective hydroalkylation product **4aa** as the major regioisomer, and δ-hydroalkylation product **4aa’** as the minor regioisomer; other regioisomers were not observed (lower than the detection limit of gas chromatography).

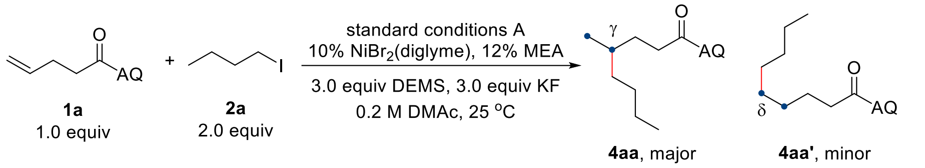

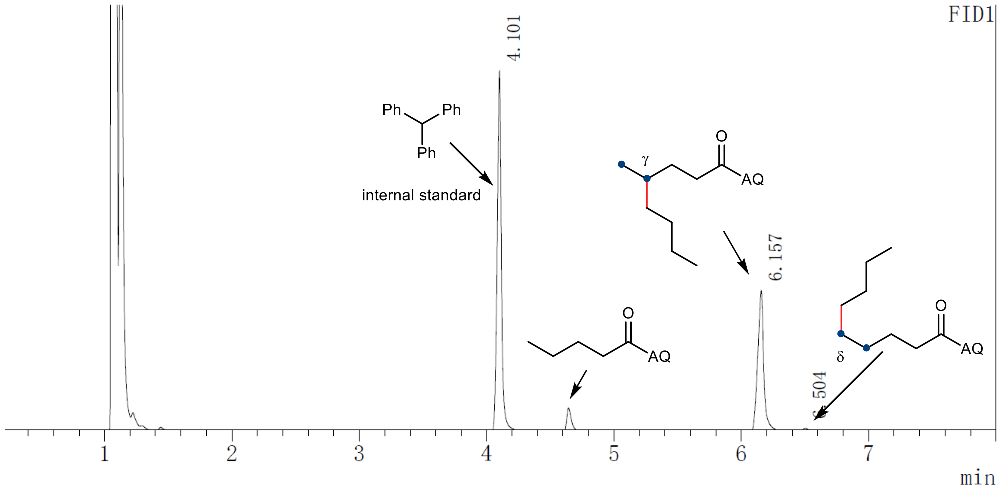

Supplementary Figure 4. GC spectra of **4aa** under standard conditions A

For internal alkene **1d** under standard conditions A, the outcome was similar to terminal alkene **1a** under standard conditions.

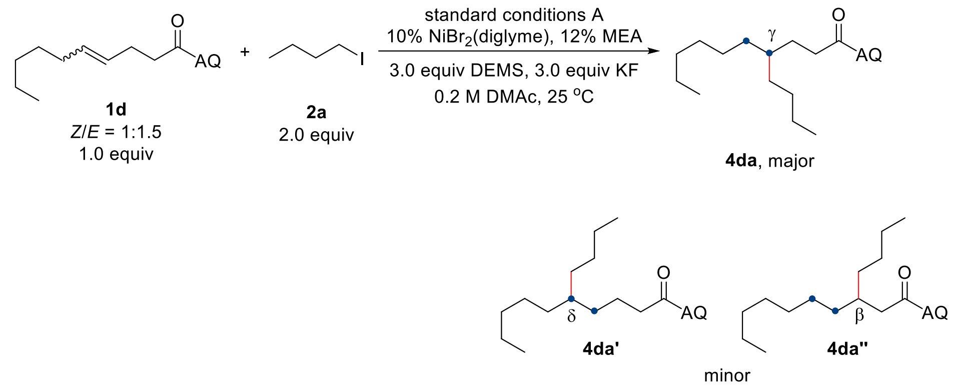

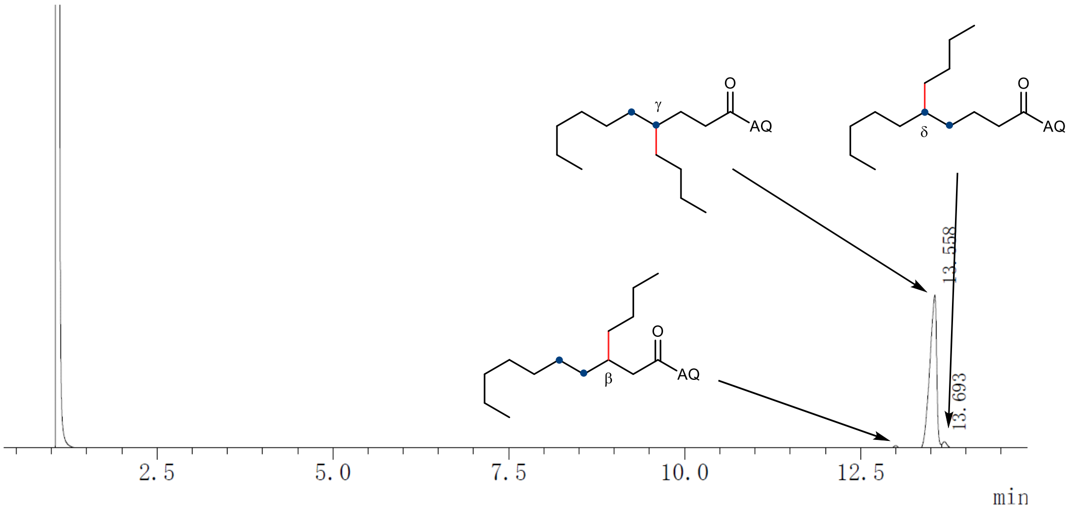

Supplementary Figure 5. GC spectra of **4da**

General Procedure 1 for the Synthesis of Proximal Alkylation Product

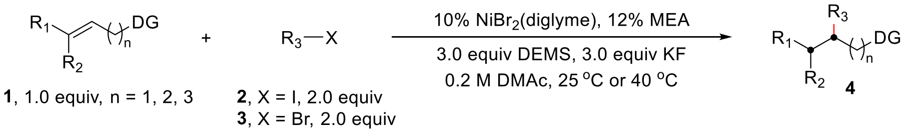

In air, a 10 mL Schlenk tube equipped with a stir bar was charged with $NiBr_2$(diglyme) (0.02 mmol, 10 mol%), KF (0.60 mmol, 3.0 equiv), alkene (0.20 mmol, 1.0 equiv), and alkyl halide (0.40 mmol, 2.0 equiv) (0.5 equiv NaI was added when alkyl bromide was used). The Schlenk tube was evacuated and filled with argon (three cycles). To these solids, a solution of ethanolamine (MEA) (0.024 mmol, 12 mol%) in anhydrous DMAc (1.0 mL) was added under an argon atmosphere. Then, DEMS (0.60 mmol, 3.0 equiv) (if the alkene or alkyl halide was liquid, it was also added at this time) was added under an argon atmosphere. The mixture was stirred at 25 °C or 40 °C for 12 h, diluted with H_2O followed by extraction with ethyl acetate, dried with anhydrous Na_2SO_4, and concentrated in vacuo. The residue was purified by column chromatography to produce the target product. Regioisomeric ratios (r.r.) were determined by GC and ^{1}H NMR

analysis.

Examples Described in Figure 2

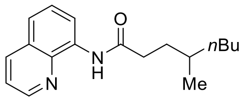

4-methyl-*N*-(quinolin-8-yl)octanamide (**4aa**)

Following general procedure 1. From **2a** (*n*Bu-I), the product was isolated by column chromatography as colorless oil (49.5 mg, 87%, r.r. > 20:1).

Following general procedure 1. From **3a** (*n*Bu-Br), the product was isolated by column chromatography as colorless oil (50.1 mg, 88%, r.r. > 20:1).

Rf (petroleum ether : ethyl acetate = 5:1) = 0.55

^{1}H NMR (500 MHz, Chloroform-*d*) δ 9.81 (s, 1H), 8.95 – 8.57 (m, 2H), 8.15 (dd, *J* = 8.3, 1.7 Hz, 1H), 7.70 – 7.35 (m, 3H), 2.66 – 2.45 (m, 2H), 1.94 – 1.77 (m, 1H), 1.71 – 1.58 (m, 1H), 1.59 – 1.48 (m, 1H), 1.41 – 1.25 (m, 5H), 1.23 – 1.14 (m, 1H), 0.95 (d, *J* = 6.6 Hz, 3H), 0.89 (t, J = 6.9 Hz, 3H).

^{13}C NMR (101 MHz, Chloroform-*d*) δ 172.3, 148.2, 138.4, 136.6, 134.7, 128.1, 127.6, 121.7, 121.4, 116.6, 36.6, 36.1, 32.7, 32.7, 29.3, 23.1, 19.6, 14.3.

HRMS (ESI) calculated for $C_{18}H_{25}N_2O^+$ $[M+H]^+$: 285.1961, found: 285.1964.

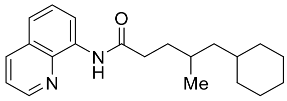

5-cyclohexyl-4-methyl-*N*-(quinolin-8-yl)pentanamide (**4ab**)

Following general procedure 1. The product was isolated by column chromatography, as colorless oil (37.2 mg, 57%, r.r. > 20:1).

Rf (petroleum ether : ethyl acetate = 5:1) = 0.50

^{1}H NMR (500 MHz, Chloroform-*d*) δ 9.81 (s, 1H), 8.87 – 8.74 (m, 2H), 8.15 (dd, *J* = 8.3, 1.7 Hz, 1H), 7.58 – 7.39 (m, 3H), 2.68 – 2.45 (m, 2H), 1.87 – 1.78 (m, 1H), 1.76 – 1.52 (m, 7H), 1.42 – 1.29 (m, 1H), 1.28 – 1.11 (m, 4H), 1.10 – 1.01 (m, 1H), 0.93 (d, *J* = 6.3 Hz, 3H), 0.90 – 0.71 (m, 2H).

^{13}C NMR (126 MHz, Chloroform-*d*) δ 172.4, 148.1, 138.3, 136.8, 134.7, 128.1, 127.7, 121.7, 121.5, 116.9, 45.1, 36.1, 35.0, 34.3, 33.3, 33.1, 29.5, 26.9, 26.6, 26.5, 19.9.

HRMS (ESI) calculated for $C_{21}H_{29}N_2O^+$ $[M+H]^+$: 325.2274, found: 325.2284.

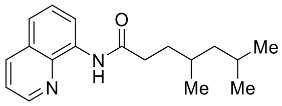

4,6-dimethyl-*N*-(quinolin-8-yl)heptanamide (**4ac**)

Following general procedure 1. The product was isolated by column chromatography, as colorless oil (29.0 mg, 51%, r.r. = 10:1).

Rf (petroleum ether : ethyl acetate = 5:1) = 0.55

^{1}H NMR (500 MHz, Chloroform-*d*) δ 9.81 (s, 1H), 8.93 – 8.57 (m, 2H), 8.15 (dd, *J* = 8.2, 1.7 Hz, 1H), 7.61 – 7.29 (m, 3H), 2.70 – 2.40 (m, 2H), 1.91 – 1.77 (m, 1H), 1.69 (tdd, *J* = 8.7, 6.1, 4.4 Hz, 1H), 1.65 – 1.54 (m, 2H), 1.20 (ddd, *J* = 13.2, 8.1, 5.0 Hz, 1H), 1.08 (ddd, *J* = 13.6, 7.8, 5.9 Hz, 1H), 0.94 (d, *J* = 6.1 Hz, 3H), 0.89 (d, *J* = 6.6 Hz, 3H), 0.85 (d, *J* = 6.5 Hz, 3H).

^{13}C NMR (126 MHz, Chloroform-*d*) δ 172.3, 148.2, 138.4, 136.5, 134.7, 128.1, 127.6, 121.7, 121.4, 116.6, 46.6, 36.0, 33.0, 30.3, 25.3, 23.5, 22.4, 19.7.

HRMS (ESI) calculated for $C_{18}H_{25}N_2O^+$ [M+H]$^+$: 285.1961, found: 285.1962.

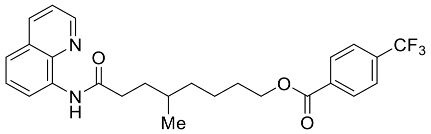

5-methyl-8-oxo-8-(quinolin-8-ylamino)octyl 4-(trifluoromethyl)benzoate (**4ad**)

Following general procedure 1. The product was isolated by column chromatography, as colorless oil (78.8 mg, 83%, r.r. = 10:1).

Rf (petroleum ether : ethyl acetate = 5:1) = 0.50

^{1}H NMR (400 MHz, Chloroform-*d*) δ 9.81 (s, 1H), 8.89 – 8.66 (m, 2H), 8.24 – 7.97 (m, 3H), 7.67 (d, *J* = 7.9 Hz, 2H), 7.56 – 7.38 (m, 3H), 4.34 (t, *J* = 6.6 Hz, 2H), 2.67 – 2.47 (m, 2H), 1.94 – 1.83 (m, 1H), 1.83 – 1.72 (m, 2H), 1.70 – 1.54 (m, 2H), 1.53 – 1.40 (m, 3H), 1.34 – 1.21 (m, 1H), 0.97 (d, *J* = 6.3 Hz, 3H).

^{19}F NMR (376 MHz, Chloroform-*d*) δ -63.06.

^{13}C NMR (126 MHz, Chloroform-*d*) δ 172.0, 165.5, 148.2, 138.4, 136.5, 134.6, 134.4 (q, *J* = 32.5 Hz), 133.8, 130.0, 128.0, 127.5, 125.5 (q, *J* = 3.7 Hz), 123.8 (q, *J* = 272.7 Hz), 121.7, 121.5, 116.5, 65.7, 36.5, 35.9, 32.5, 32.5, 29.0, 23.5, 19.5.

HRMS (ESI) calculated for $C_{26}H_{28}F_3N_2O_3^+$ [M+H]$^+$: 473.2047, found: 473.2049.

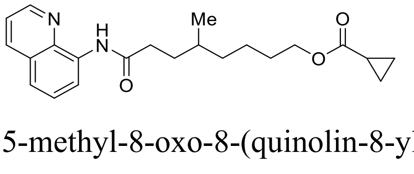

5-methyl-8-oxo-8-(quinolin-8-ylamino)octyl cyclopropanecarboxylate (**4ae**)

Following general procedure 1. The product was isolated by column chromatography, as colorless oil (58.6 mg, 80%, r.r. = 11:1).

Rf (petroleum ether : ethyl acetate = 5:1) = 0.50

^{1}H NMR (400 MHz, Chloroform-*d*) δ 9.79 (s, 1H), 8.91 – 8.66 (m, 2H), 8.13 (dd, *J* = 8.3, 1.7 Hz, 1H), 7.61 – 7.37 (m, 3H), 4.05 (t, *J* = 6.7 Hz, 2H), 2.72 – 2.43 (m, 2H), 1.91 – 1.78 (m, 1H), 1.68 – 1.48 (m, 5H), 1.45 – 1.30 (m, 3H), 1.25 – 1.16 (m, 1H), 1.00 – 0.91 (m, 5H), 0.81 (dt, *J* = 8.2, 3.4 Hz, 2H).

^{13}C NMR (126 MHz, Chloroform-*d*) δ 175.0, 172.0, 148.2, 138.4, 136.5, 134.6, 128.0, 127.5, 121.6, 121.4, 116.5, 64.6, 36.5, 35.9, 32.5, 32.5, 29.0, 23.4, 19.4, 13.0, 8.4.

HRMS (ESI) calculated for $C_{22}H_{29}N_2O_3^+$ [M+H]$^+$: 369.2173, found: 369.2185.

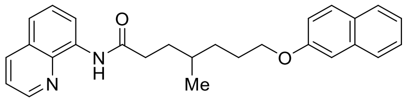

4-methyl-7-(naphthalen-2-yloxy)-*N*-(quinolin-8-yl)heptanamide (**4af**)

Following general procedure 1. The product was isolated by column chromatography, as white solid (65.6 mg, 80%, r.r. = 13:1).

Rf (petroleum ether : ethyl acetate = 5:1) = 0.45

^{1}H NMR (500 MHz, Chloroform-*d*) δ 9.83 (s, 1H), 8.87 – 8.69 (m, 2H), 8.12 (dd, *J* = 8.2, 1.8 Hz, 1H), 7.79 – 7.63 (m, 3H), 7.52 (t, *J* = 7.9 Hz, 1H), 7.47 (dd, *J* = 8.2, 1.5 Hz, 1H), 7.45 – 7.37 (m, 2H), 7.33 – 7.27 (m, 1H), 7.17 – 7.07 (m, 2H), 4.05 (t, *J* = 6.5 Hz, 2H), 2.74 – 2.48 (m, 2H), 2.00 – 1.79 (m, 3H), 1.76 – 1.55 (m, 3H), 1.44 – 1.34 (m, 1H), 1.02 (d, *J* = 6.2 Hz, 3H).

^{13}C NMR (101 MHz, Chloroform-*d*) δ 172.0, 157.1, 148.1, 138.2, 136.5, 134.6, 134.5, 129.3, 128.9, 128.0, 127.6, 127.5, 126.7, 126.3, 123.5, 121.6, 121.4, 119.0, 116.6, 106.6, 68.2, 35.9, 33.1, 32.5, 32.4, 26.8, 19.4.

HRMS (ESI) calculated for $C_{27}H_{29}N_2O_2^+$ [M+H]$^+$: 413.2224, found: 413.2229.

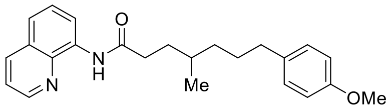

7-(4-methoxyphenyl)-4-methyl-*N*-(quinolin-8-yl)heptanamide (**4ag**)

Following general procedure 1. The product was isolated by column chromatography, as colorless oil (61.9 mg, 82%, r.r. = 11:1).

Rf (petroleum ether : ethyl acetate = 5:1) = 0.50

^{1}H NMR (400 MHz, Chloroform-*d*) δ 9.82 (s, 1H), 9.04 – 8.60 (m, 2H), 8.16 (dt, *J* = 8.3, 1.5 Hz, 1H), 7.71 – 7.35 (m, 3H), 7.18 – 7.05 (m, 2H), 6.90 – 6.62 (m, 2H), 3.78 (s, 3H), 2.67 – 2.44 (m, 4H), 1.95 – 1.78 (m, 1H), 1.75 – 1.51 (m, 4H), 1.50 – 1.36 (m, 1H), 1.31 – 1.16 (m, 1H), 0.96 (d, *J* = 6.4 Hz, 3H).

^{13}C NMR (126 MHz, Chloroform-*d*) δ 172.2, 157.7, 148.2, 138.4, 136.5, 134.9, 134.7, 129.3, 128.0, 127.6, 121.7, 121.4, 116.5, 113.8, 55.3, 36.4, 36.0, 35.4, 32.6, 32.5, 29.2, 19.5.

HRMS (ESI) calculated for $C_{24}H_{29}N_2O_2^+$ $[M+H]^+$: 377.2224, found: 377.2227.

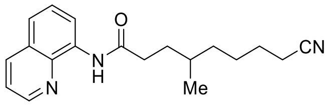

8-cyano-4-methyl-*N*-(quinolin-8-yl)octanamide (**4ah**)

Following general procedure 1. The product was isolated by column chromatography, as colorless oil (44.9 mg, 73%, r.r. > 20:1).

Rf (petroleum ether : ethyl acetate = 4:1) = 0.30

^{1}H NMR (500 MHz, Chloroform-*d*) δ 9.80 (s, 1H), 8.92 – 8.62 (m, 2H), 8.14 (dd, *J* = 8.3, 1.7 Hz, 1H), 7.62 – 7.34 (m, 3H), 2.65 – 2.45 (m, 2H), 2.32 (t, *J* = 7.2 Hz, 2H), 1.92 – 1.81 (m, 1H), 1.68 – 1.58 (m, 3H), 1.57 – 1.33 (m, 4H), 1.28 – 1.13 (m, 1H), 0.95 (d, *J* = 6.5 Hz, 3H).

^{13}C NMR (126 MHz, Chloroform-*d*) δ 171.9, 148.2, 138.3, 136.5, 134.6, 128.0, 127.5, 121.7, 121.5, 119.9, 116.5, 36.0, 35.8, 32.5, 32.4, 26.2, 25.7, 19.4, 17.2.

HRMS (ESI) calculated for $C_{19}H_{24}N_3O^+$ $[M+H]^+$: 310.1914, found: 310.1919.

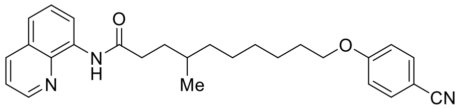

10-(4-cyanophenoxy)-4-methyl-*N*-(quinolin-8-yl)decanamide (**4ai**)

Following general procedure 1. The product was isolated by column chromatography, as colorless oil (66.2 mg, 77%, r.r. = 18:1).

Rf (petroleum ether : ethyl acetate = 4:1) = 0.35

^{1}H NMR (500 MHz, Chloroform-*d*) δ 9.81 (s, 1H), 8.79 (dd, *J* = 8.2, 5.8 Hz, 2H), 8.16 (dt, *J* = 8.4, 1.7 Hz, 1H), 7.62 – 7.39 (m, 5H), 6.91 (d, *J* = 8.4 Hz, 2H), 3.97 (t, *J* = 6.6 Hz, 2H), 2.65 – 2.47 (m, 2H), 1.91 – 1.83 (m, 1H), 1.83 – 1.73 (m, 2H), 1.69 – 1.59 (m, 1H), 1.58 – 1.51 (m, 1H), 1.48 – 1.41 (m, 2H), 1.41 – 1.29 (m, 5H), 1.27 – 1.16 (m, 1H), 0.96 (d, *J* = 6.5 Hz, 3H).

^{13}C NMR (126 MHz, Chloroform-*d*) δ 172.1, 162.5, 148.1, 138.3, 136.5, 134.6, 133.9, 128.0, 127.5, 121.6, 121.4, 119.4, 116.5, 115.2, 103.6, 68.4, 36.7, 35.9, 32.5, 32.5, 29.6, 29.0, 26.8, 25.9, 19.5.

HRMS (ESI) calculated for $C_{27}H_{32}N_3O_2^+$ $[M+H]^+$: 430.2489, found:430.2493.

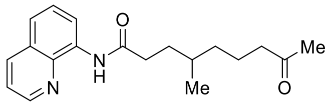

4-methyl-8-oxo-*N*-(quinolin-8-yl)nonanamide (**4aj**)

Following general procedure 1. The product was isolated by column chromatography, as colorless oil (39.6 mg, 64%, r.r. > 20:1).

Rf (petroleum ether : ethyl acetate = 4:1) = 0.40

^{1}H NMR (500 MHz, Chloroform-*d*) δ 9.81 (s, 1H), 8.94 – 8.65 (m, 2H), 8.15 (dt, *J* = 8.3, 1.6 Hz, 1H), 7.62 – 7.36 (m, 3H), 2.66 – 2.47 (m, 2H), 2.41 (t, *J* = 7.5 Hz, 2H), 2.11 (s, 3H), 1.91 – 1.81 (m, 1H), 1.69 – 1.51 (m, 4H), 1.40 – 1.33 (m, 1H), 1.24 – 1.14 (m, 1H), 0.96 (d, *J* = 6.6 Hz, 3H).

^{13}C NMR (126 MHz, Chloroform-*d*) δ 209.3, 172.1, 148.2, 138.4, 136.6, 134.6, 128.1, 127.6, 121.7, 121.5, 116.6, 44.0, 36.3, 35.9, 32.5, 32.5, 30.0, 21.4, 19.4.

HRMS (ESI) calculated for $C_{19}H_{25}N_2O_2^+$ $[M+H]^+$: 313.1911, found: 313.1917.

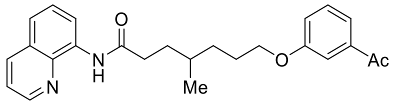

7-(3-acetylphenoxy)-4-methyl-*N*-(quinolin-8-yl)heptanamide (**4ak**)

Following general procedure 1. The product was isolated by column chromatography, as colorless oil (48.5 mg, 60%, r.r. > 20:1).

Rf (petroleum ether : ethyl acetate = 3:1) = 0.35

^{1}H NMR (500 MHz, Chloroform-*d*) δ 9.82 (s, 1H), 8.92 – 8.67 (m, 2H), 8.14 (dt, *J* = 8.2, 1.4 Hz, 1H), 7.59 – 7.39 (m, 5H), 7.33 (t, *J* = 7.9 Hz, 1H), 7.08 (dd, *J* = 8.2, 2.6 Hz, 1H), 3.99 (t, *J* = 6.4 Hz, 2H), 2.66 – 2.51 (m, 5H), 1.96 – 1.74 (m, 3H), 1.74 – 1.53 (m, 3H), 1.41 – 1.32 (m, 1H), 1.01 (d, *J* = 6.2 Hz, 3H).

^{13}C NMR (126 MHz, Chloroform-*d*) δ 198.1, 172.0, 159.4, 148.2, 138.5, 138.4, 136.5, 134.6, 129.6, 128.0, 127.5, 121.7, 121.5, 121.0, 120.1, 116.6, 113.3, 68.5, 35.9, 33.1, 32.5, 32.4, 26.8, 26.8, 19.5.

HRMS (ESI) calculated for $C_{25}H_{29}N_2O_3^+$ $[M+H]^+$: 405.2173, found: 405.2181.

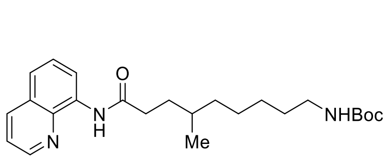

tert-butyl (6-methyl-9-oxo-9-(quinolin-8-ylamino)nonyl)carbamate (**4al**)

Following general procedure 1. The product was isolated by column chromatography, as colorless oil (61.4 mg, 74%, r.r. > 20:1).

Rf (petroleum ether : ethyl acetate = 4:1) = 0.40

^{1}H NMR (500 MHz, Chloroform-*d*) δ 9.81 (s, 1H), 8.92 – 8.69 (m, 2H), 8.16 (dt, *J* = 8.3, 1.7 Hz, 1H), 7.67 – 7.33 (m, 3H), 4.52 (s, 1H), 3.21 – 2.91 (m, 2H), 2.67 – 2.45 (m, 2H), 1.94 – 1.79 (m, 1H), 1.68 – 1.59 (m, 1H), 1.54 (q, *J* = 7.4, 6.3 Hz, 1H), 1.49 – 1.41 (m, 11H), 1.39 – 1.33 (m, 2H), 1.32 – 1.26 (m, 3H), 1.22 – 1.14 (m, 1H), 0.94 (d, *J* = 6.4 Hz, 3H).

^{13}C NMR (126 MHz, Chloroform-*d*) δ 172.2, 156.1, 148.2, 138.4, 136.6, 134.7, 128.1, 127.6, 121.7, 121.5, 116.6, 79.1, 40.7, 36.8, 36.0, 32.7, 32.6, 30.2, 28.6, 27.2, 26.8, 19.5.

HRMS (ESI) calculated for $C_{24}H_{35}N_3NaO_3^+$ [M+Na]$^+$: 436.2571, found: 436.2577.

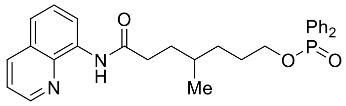

4-methyl-7-oxo-7-(quinolin-8-ylamino)heptyl diphenylphosphinate (**4am**)

Following general procedure 1. The product was isolated by column chromatography, as colorless oil (75.9 mg, 78%, r.r. > 20:1).

Rf (petroleum ether : ethyl acetate = 1:1) = 0.35

^{1}H NMR (500 MHz, Chloroform-*d*) δ 9.81 (s, 1H), 8.88 – 8.60 (m, 2H), 8.16 (dt, *J* = 8.3, 1.9 Hz, 1H), 7.91 – 7.73 (m, 4H), 7.58 – 7.33 (m, 9H), 4.03 (q, *J* = 6.7 Hz, 2H), 2.66 – 2.43 (m, 2H), 1.90 – 1.68 (m, 3H), 1.67 – 1.45 (m, 3H), 1.34 – 1.21 (m, 1H), 0.95 (d, *J* = 6.5 Hz, 3H).

^{13}C NMR (126 MHz, Chloroform-*d*) δ 172.0, 148.2, 138.3, 136.6, 134.6, 132.2 (d, *J* = 2.8 Hz), 131.8 (d, *J* = 137.0 Hz), 131.7 (d, *J* = 10.1 Hz), 128.6 (d, *J* = 13.0 Hz), 128.1, 127.6, 121.7, 121.5, 116.7, 65.3 (d, *J* = 6.0 Hz), 35.9, 32.7, 32.5, 32.3, 28.2 (d, *J* = 6.5 Hz), 19.4.

^{31}P NMR (162 MHz, Chloroform-*d*) δ 31.25.

HRMS (ESI) calculated for $C_{29}H_{32}N_2O_3P^+$ [M+H]$^+$: 487.2145, found: 487.2148.

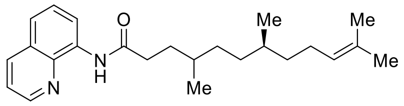

(7*S*)-4,7,11-trimethyl-*N*-(quinolin-8-yl)dodec-10-enamide (**4an**)

Following general procedure 1. The product was isolated by column chromatography, as colorless oil (46.6 mg, 64%, r.r. > 20:1, 1.2:1 d.r.).

Rf (petroleum ether : ethyl acetate = 4:1) = 0.55

^{1}H NMR (500 MHz, Chloroform-*d*) δ 9.82 (s, 1H), 8.87 – 8.63 (m, 2H), 8.16 (dt, *J* = 8.4, 1.8 Hz, 1H), 7.62 – 7.40 (m, 3H), 5.09 (t, *J* = 7.3 Hz, 1H), 2.68 – 2.46 (m, 2H), 2.05 – 1.81 (m, 3H), 1.71 – 1.57 (m, 7H), 1.55 – 1.47 (m, 1H), 1.45 – 1.28 (m, 4H), 1.23 – 1.06 (m, 3H), 0.96 (dd, *J* = 6.6, 2.4 Hz, 3H), 0.87 (dd, *J* = 6.4, 2.5 Hz, 3H).

^{13}C NMR (126 MHz, Chloroform-*d*) δ 172.3, 148.2, 138.4, 136.6, 134.7, 131.1, 128.1, 127.6, 125.2, 121.7, 121.4, 116.6, 37.3, 37.1, 36.1, 36.1, 34.4, 34.3, 34.2, 33.1, 33.0, 32.9, 32.9, 32.8, 32.7, 25.9, 25.7, 25.7, 19.8, 19.7, 19.7, 19.6, 17.8.
HRMS (ESI) calculated for $C_{24}H_{35}N_2O^+$ [M+H]$^+$: 367.2744, found: 367.2747.

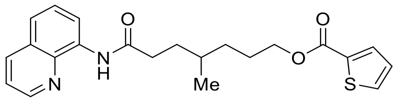

4-methyl-7-oxo-7-(quinolin-8-ylamino)heptyl thiophene-2-carboxylate (**4ao**)
Following general procedure 1. The product was isolated by column chromatography, as colorless oil (57.1 mg, 72%, r.r. = 16:1).
Rf (petroleum ether : ethyl acetate = 4:1) = 0.30
^{1}H NMR (400 MHz, Chloroform-*d*) δ 9.85 (s, 1H), 8.80 (ddd, *J* = 8.9, 5.8, 1.7 Hz, 2H), 8.18 (dd, *J* = 8.2, 1.7 Hz, 1H), 7.79 (dd, *J* = 3.8, 1.3 Hz, 1H), 7.63 – 7.39 (m, 4H), 7.08 (dd, *J* = 5.0, 3.7 Hz, 1H), 4.30 (t, *J* = 6.7 Hz, 2H), 2.71 – 2.48 (m, 2H), 1.95 – 1.87 (m, 1H), 1.86 – 1.74 (m, 2H), 1.70 – 1.60 (m, 2H), 1.58 – 1.49 (m, 1H), 1.37 – 1.29 (m, 1H), 1.00 (d, *J* = 6.2 Hz, 3H).
^{13}C NMR (126 MHz, Chloroform-*d*) δ 172.0, 162.4, 148.1, 138.3, 136.7, 134.6, 134.2, 133.4, 132.3, 128.1, 127.8, 127.7, 121.7, 121.5, 116.8, 65.6, 35.9, 33.1, 32.5, 32.4, 26.3, 19.5.
HRMS (ESI) calculated for $C_{22}H_{25}N_2O_3S^+$ [M+H]$^+$: 397.1580, found: 397.1590.

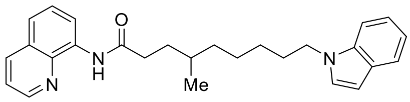

9-(1*H*-indol-1-yl)-4-methyl-*N*-(quinolin-8-yl)nonanamide (**4ap**)
Following general procedure 1. The product was isolated by column chromatography, as yellow oil (64.5 mg, 78%, r.r. > 20:1).
Rf (petroleum ether : ethyl acetate = 3:1) = 0.50
^{1}H NMR (400 MHz, Chloroform-*d*) δ 9.83 (s, 1H), 8.92 – 8.64 (m, 2H), 8.16 (dd, *J* = 8.3, 1.7 Hz, 1H), 7.64 (d, *J* = 7.9 Hz, 1H), 7.59 – 7.48 (m, 2H), 7.45 (dd, *J* = 8.3, 4.2 Hz, 1H), 7.35 (d, *J* = 8.2 Hz, 1H), 7.21 (t, *J* = 7.6 Hz, 1H), 7.10 (dd, *J* = 7.8, 5.3 Hz, 2H), 6.49 (d, *J* = 3.1 Hz, 1H), 4.10 (t, *J* = 7.1 Hz, 2H), 2.65 – 2.46 (m, 2H), 1.94 – 1.78 (m, 3H), 1.67 – 1.58 (m, 1H), 1.56 – 1.48 (m, 1H), 1.45 – 1.25 (m, 5H), 1.21 – 1.12 (m, 1H), 0.95 (d, *J* = 6.5 Hz, 3H).

^{13}C NMR (101 MHz, Chloroform-*d*) δ 172.2, 148.2, 138.4, 136.6, 136.0, 134.7, 128.7, 128.1, 127.9, 127.6, 121.7, 121.5, 121.4, 121.0, 119.2, 116.6, 109.5, 100.9, 46.5, 36.7, 36.0, 32.6, 32.5, 30.3, 27.4, 26.7, 19.6.

HRMS (ESI) calculated for $C_{27}H_{32}N_3O^+$ $[M+H]^+$: 414.2540, found: 414.2544.

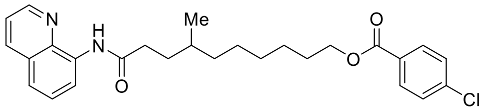

7-methyl-10-oxo-10-(quinolin-8-ylamino)decyl 4-chlorobenzoate (**4aq**)

Following general procedure 1. The product was isolated by column chromatography, as colorless oil (73.8 mg, 79%, r.r. = 12:1).

Rf (petroleum ether : acetone = 5:1) = 0.45

^{1}H NMR (400 MHz, Chloroform-*d*) δ 9.82 (s, 1H), 8.88 – 8.64 (m, 2H), 8.16 (dt, *J* = 8.3, 1.7 Hz, 1H), 7.96 (d, *J* = 8.5 Hz, 2H), 7.57 – 7.35 (m, 5H), 4.29 (t, *J* = 6.7 Hz, 2H), 2.71 – 2.42 (m, 2H), 1.93 – 1.80 (m, 1H), 1.78 – 1.69 (m, 2H), 1.69 – 1.59 (m, 1H), 1.57 – 1.51 (m, 1H), 1.47 – 1.27 (m, 7H), 1.27 – 1.15 (m, 1H), 0.95 (d, *J* = 6.5 Hz, 3H).

^{13}C NMR (126 MHz, Chloroform-*d*) δ 172.2, 165.9, 148.1, 139.3, 138.3, 136.7, 134.6, 131.1, 129.1, 128.8, 128.1, 127.6, 121.7, 121.5, 116.7, 65.5, 36.8, 36.0, 32.6, 29.7, 28.8, 27.0, 26.1, 19.6.

HRMS (ESI) calculated for $C_{27}H_{32}ClN_2O_3^+$ $[M+H]^+$: 467.2096, found: 467.2100.

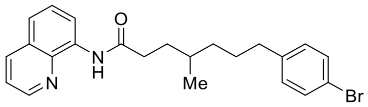

7-(4-bromophenyl)-4-methyl-*N*-(quinolin-8-yl)heptanamide (**4ar**)

Following general procedure 1. The product was isolated by column chromatography, as colorless oil (70.6 mg, 83%, r.r. = 15:1).

Rf (petroleum ether : ethyl acetate = 4:1) = 0.60

^{1}H NMR (500 MHz, Chloroform-*d*) δ 9.81 (s, 1H), 8.89 – 8.64 (m, 2H), 8.15 (dd, *J* = 8.3, 1.8 Hz, 1H), 7.57 – 7.47 (m, 2H), 7.44 (dd, *J* = 8.4, 4.2 Hz, 1H), 7.36 (d, *J* = 8.1 Hz, 2H), 7.03 (d, *J* = 8.0 Hz, 2H), 2.66 – 2.32 (m, 4H), 1.93 – 1.83 (m, 1H), 1.70 – 1.54 (m, 4H), 1.48 – 1.37 (m, 1H), 1.27 – 1.17 (m, 1H), 0.96 (d, *J* = 6.4 Hz, 3H).

^{13}C NMR (126 MHz, Chloroform-*d*) δ 172.0, 148.2, 141.7, 138.4, 136.5, 134.6, 131.4, 130.3, 128.0, 127.6, 121.7, 121.5, 119.4, 116.6, 36.3, 35.9, 35.6, 32.5, 32.5, 28.8, 19.5.

HRMS (ESI) calculated for $C_{23}H_{26}BrN_2O^+$ $[M+H]^+$: 425.1223, found: 425.1236.

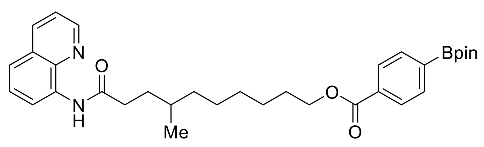

7-methyl-10-oxo-10-(quinolin-8-ylamino)decyl 4-(4,4,5,5-tetramethyl-1,3,2-dioxaborolan-2-yl)benzoate (**4as**)

Following general procedure 1. The product was isolated by column chromatography, as colorless oil (73.7 mg, 66%, r.r. > 20:1).

Rf (petroleum ether : acetone = 5:1) = 0.55

^{1}H NMR (400 MHz, Chloroform-*d*) δ 9.82 (s, 1H), 8.93 – 8.67 (m, 2H), 8.26 – 8.10 (m, 1H), 8.09 – 7.95 (m, 2H), 7.91 – 7.74 (m, 2H), 7.61 – 7.39 (m, 3H), 4.30 (t, *J* = 6.7 Hz, 2H), 2.70 – 2.47 (m, 2H), 1.92 – 1.81 (m, 1H), 1.80 – 1.70 (m, 2H), 1.70 – 1.60 (m, 1H), 1.60 – 1.49 (m, 1H), 1.47 – 1.27 (m, 20H), 0.95 (d, *J* = 6.5 Hz, 3H).

^{13}C NMR (101 MHz, Chloroform-*d*) δ 172.2, 166.8, 148.1, 138.4, 136.6, 134.8, 134.7, 132.8, 128.7, 128.1, 127.6, 121.7, 121.4, 116.6, 84.3, 65.3, 36.8, 36.1, 32.7, 32.7, 29.7, 28.8, 27.0, 26.2, 25.0, 19.6.

^{11}B NMR (128 MHz, Chloroform-*d*) δ 31.50.

HRMS (ESI) calculated for $C_{33}H_{44}BN_2O_5^+$ $[M+H]^+$: 559.3338, found: 559.3356.

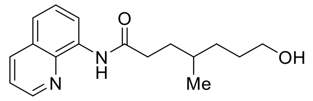

7-hydroxy-4-methyl-*N*-(quinolin-8-yl)heptanamide (**4at**)

Following general procedure 1. The product was isolated by column chromatography, as colorless oil (40.1 mg, 70%, r.r. = 18:1).

Rf (petroleum ether : ethyl acetate = 2:1) = 0.40

^{1}H NMR (500 MHz, Chloroform-*d*) δ 9.82 (s, 1H), 8.95 – 8.59 (m, 2H), 8.17 (dd, *J* = 8.2, 1.7 Hz, 1H), 7.67 – 7.34 (m, 3H), 3.65 (t, *J* = 6.5 Hz, 2H), 2.69 – 2.52 (m, 2H), 1.91 – 1.79 (m, 1H), 1.72 – 1.62 (m, 3H), 1.61 – 1.55 (m, 2H), 1.54 – 1.45 (m, 1H), 1.31 – 1.20 (m, 1H), 0.97 (d, *J* = 6.6 Hz, 3H).

^{13}C NMR (126 MHz, Chloroform-*d*) δ 172.2, 148.2, 138.3, 136.7, 134.6, 128.1, 127.6, 121.7, 121.6, 116.8, 63.2, 35.9, 32.7, 32.6, 32.3, 30.2, 19.6.

HRMS (ESI) calculated for $C_{17}H_{23}N_2O_2^+$ $[M+H]^+$: 287.1754, found: 287.1762.

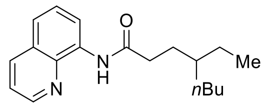

4-ethyl-*N*-(quinolin-8-yl)octanamide (**4ba**)

Following general procedure 1. From *E*-**1b**, the product was isolated by column chromatography, as colorless oil (44.2 mg, 74%, r.r. = 14:1).

Following general procedure 1. From *Z*-**1b**, the product was isolated by column chromatography, as colorless oil (43.0 mg, 72%, r.r. = 13:1).

Rf (petroleum ether : ethyl acetate = 5:1) = 0.55

^{1}H NMR (500 MHz, Chloroform-*d*) δ 9.82 (s, 1H), 8.92 – 8.54 (m, 2H), 8.16 (d, *J* = 8.1 Hz, 1H), 7.67 – 7.31 (m, 3H), 2.55 (dd, *J* = 9.5, 6.6 Hz, 2H), 1.79 (dt, *J* = 11.4, 5.9 Hz, 2H), 1.44 – 1.25 (m, 9H), 0.97 – 0.84 (m, 6H).

^{13}C NMR (126 MHz, Chloroform-*d*) δ 172.4, 148.2, 138.4, 136.6, 134.7, 128.1, 127.6, 121.7, 121.4, 116.6, 38.7, 35.8, 32.7, 29.0, 29.0, 25.8, 23.3, 14.3, 10.9.

HRMS (ESI) calculated for $C_{19}H_{27}N_2O^+$ [M+H]$^+$: 299.2118, found: 299.2130.

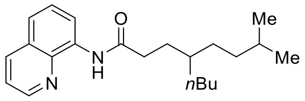

4-butyl-7-methyl-*N*-(quinolin-8-yl)octanamide (**4ca**)

Following general procedure 1. The product was isolated by column chromatography, as colorless oil (49.7 mg, 73%, r.r. > 20:1).

Rf (petroleum ether : ethyl acetate = 5:1) = 0.50

^{1}H NMR (500 MHz, Chloroform-*d*) δ 9.82 (s, 1H), 8.88 – 8.63 (m, 2H), 8.15 (dd, *J* = 8.2, 1.8 Hz, 1H), 7.64 – 7.36 (m, 3H), 2.61 – 2.45 (m, 2H), 1.86 – 1.72 (m, 2H), 1.57 – 1.46 (m, 1H), 1.42 (p, *J* = 5.9 Hz, 1H), 1.36 – 1.25 (m, 8H), 1.24 – 1.14 (m, 2H), 1.00 – 0.77 (m, 9H).

^{13}C NMR (126 MHz, Chloroform-*d*) δ 172.4, 148.2, 138.4, 136.5, 134.7, 128.1, 127.6, 121.7, 121.4, 116.6, 37.5, 35.9, 35.7, 33.2, 31.2, 29.5, 29.0, 28.5, 23.3, 22.8, 14.3.

HRMS (ESI) calculated for $C_{22}H_{33}N_2O^+$ [M+H]$^+$: 341.2587, found: 341.2596.

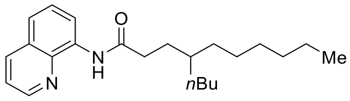

4-butyl-*N*-(quinolin-8-yl)decanamide (**4da**)

Following general procedure 1. The product was isolated by column chromatography, as colorless oil (60.9 mg, 86%, r.r. > 20:1).

Rf (petroleum ether : ethyl acetate = 5:1) = 0.50

^{1}H NMR (500 MHz, Chloroform-*d*) δ 9.82 (s, 1H), 8.80 (t, *J* = 6.2 Hz, 2H), 8.15 (dd, *J* = 8.3, 1.7 Hz, 1H), 7.71 – 7.33 (m, 3H), 2.62 – 2.42 (m, 2H), 1.88 – 1.66 (m, 2H), 1.50 – 1.38 (m, 1H), 1.37 – 1.14 (m, 16H), 1.01 – 0.78 (m, 6H).

^{13}C NMR (101 MHz, Chloroform-*d*) δ 172.4, 148.2, 138.4, 136.6, 134.7, 128.1, 127.6, 121.7, 121.4, 116.6, 37.2, 35.7, 33.5, 33.2, 32.0, 29.9, 29.5, 29.0, 26.7, 23.3, 22.8, 14.3, 14.2.

HRMS (ESI) calculated for $C_{23}H_{35}N_2O^+$ [M+H]$^+$: 355.2744, found: 355.2754.

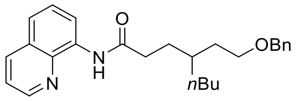

4-(2-(benzyloxy)ethyl)-*N*-(quinolin-8-yl)octanamide (**4ea**)

Following general procedure 1. The product was isolated by column chromatography, as colorless oil (54.4 mg, 67%, r.r. = 19:1).

Rf (petroleum ether : ethyl acetate = 3:1) = 0.45

^{1}H NMR (500 MHz, Chloroform-*d*) δ 9.81 (s, 1H), 8.83 – 8.68 (m, 2H), 8.14 (dd, *J* = 8.3, 1.7 Hz, 1H), 7.60 – 7.40 (m, 3H), 7.36 – 7.21 (m, 5H), 4.50 (s, 2H), 3.54 (t, *J* = 6.7 Hz, 2H), 2.55 (dd, *J* = 8.7, 7.3 Hz, 2H), 1.87 – 1.77 (m, 2H), 1.70 – 1.57 (m, 3H), 1.38 – 1.19 (m, 6H), 0.96 – 0.80 (m, 3H).

^{13}C NMR (126 MHz, Chloroform-*d*) δ 172.1, 148.2, 138.7, 138.4, 136.5, 134.7, 128.4, 128.0, 127.7, 127.6, 121.7, 121.4, 116.6, 73.1, 68.6, 35.5, 34.5, 33.6, 33.3, 29.5, 28.8, 23.2, 14.2.

HRMS (ESI) calculated for $C_{26}H_{33}N_2O_2^+$ [M+H]$^+$: 405.2537, found: 405.2544.

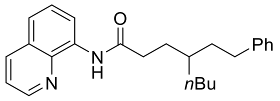

4-phenethyl-*N*-(quinolin-8-yl)octanamide (**4fa**)

Following general procedure 1. The product was isolated by column chromatography, as colorless oil (56.3 mg, 75%, r.r. > 20:1).

Rf (petroleum ether : ethyl acetate = 4:1) = 0.45

^{1}H NMR (500 MHz, Chloroform-*d*) δ 9.81 (s, 1H), 8.84 – 8.60 (m, 2H), 8.14 (dd, *J* = 8.3, 1.7 Hz, 1H), 7.75 – 7.34 (m, 3H), 7.31 – 7.06 (m, 5H), 2.68 – 2.60 (m, 2H), 2.55 (t, *J* = 8.0 Hz, 2H), 1.90 – 1.80 (m, 2H), 1.70 – 1.60 (m, 2H), 1.55 – 1.48 (m, 1H), 1.42 – 1.27 (m, 6H), 0.98 – 0.83 (m, 3H).

^{13}C NMR (126 MHz, Chloroform-*d*) δ 172.1, 148.2, 143.0, 138.4, 136.5, 134.7, 128.5, 128.4, 128.1, 127.6, 125.7, 121.7, 121.4, 116.6, 37.0, 35.6, 35.6, 33.2, 33.0, 29.3, 28.8, 23.2, 14.3.

HRMS (ESI) calculated for $C_{25}H_{31}N_2O^+$ [M+H]$^+$: 375.2431, found: 375.2438.

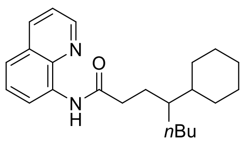

4-cyclohexyl-*N*-(quinolin-8-yl)octanamide (**4ga**)

Following general procedure 1. The product was isolated by column chromatography, as colorless oil (28.2 mg, 40%, r.r. > 20:1).

Rf (petroleum ether : ethyl acetate = 5:1) = 0.50

^{1}H NMR (400 MHz, Chloroform-*d*) δ 9.81 (s, 1H), 8.79 (t, *J* = 6.5 Hz, 2H), 8.15 (d, *J* = 8.2 Hz, 1H), 7.62 – 7.37 (m, 3H), 2.63 – 2.45 (m, 2H), 1.94 – 1.79 (m, 1H), 1.79 – 1.53 (m, 6H), 1.44 – 1.15 (m, 11H), 1.15 – 1.00 (m, 2H), 0.93 – 0.81 (m, 3H).

^{13}C NMR (126 MHz, Chloroform-*d*) δ 172.4, 148.2, 138.4, 136.6, 134.7, 128.1, 127.6, 121.7, 121.4, 116.6, 43.1, 40.2, 36.7, 30.4, 30.1, 29.9, 29.8, 27.1, 27.0, 26.7, 23.3, 14.3.

HRMS (ESI) calculated for $C_{23}H_{33}N_2O^+$ $[M+H]^+$: 353.2587, found: 353.2600.

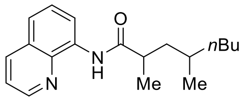

2,4-dimethyl-*N*-(quinolin-8-yl)octanamide (**4ha**)

Following general procedure 1. The product was isolated by column chromatography, as colorless oil (46.9 mg, 79%, r.r. = 20:1, 3.1:1 d.r.).

Rf (petroleum ether : ethyl acetate = 4:1) = 0.60

^{1}H NMR (400 MHz, Chloroform-*d*) δ 9.89 (s, 1H), 8.81 (dt, *J* = 5.9, 1.6 Hz, 2H), 8.16 (dd, *J* = 8.3, 1.7 Hz, 1H), 7.64 – 7.39 (m, 3H), 2.86 – 2.55 (m, 1H), 2.02 – 1.76 (m, 1H), 1.59 – 1.48 (m, 1H), 1.37 – 1.09 (m, 10H), 1.00 – 0.90 (m, 3H), 0.90 – 0.80 (m, 3H).

^{13}C NMR (126 MHz, Chloroform-*d*) δ 175.8, 175.5, 148.1, 138.4, 136.5, 134.6, 128.0, 127.5, 121.6, 121.3, 116.6, 42.1, 41.8, 40.8, 40.7, 37.0, 36.8, 30.8, 30.7, 29.2, 29.1, 23.0, 19.8, 19.8, 19.0, 18.0, 14.1.

HRMS (ESI) calculated for $C_{19}H_{27}N_2O^+$ $[M+H]^+$: 299.2118, found: 299.2126.

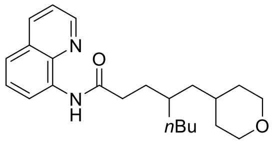

N-(quinolin-8-yl)-4-((tetrahydro-2*H*-pyran-4-yl)methyl)octanamide (**4ia**)

Following general procedure 1. The product was isolated by column chromatography, as colorless oil (54.5 mg, 74%, r.r. > 20:1).

Rf (petroleum ether : ethyl acetate = 2:1) = 0.55

¹H NMR (400 MHz, Chloroform-*d*) δ 9.81 (s, 1H), 8.91 – 8.72 (m, 2H), 8.16 (dt, *J* = 8.3, 1.4 Hz, 1H), 7.62 – 7.37 (m, 3H), 3.97 – 3.83 (m, 2H), 3.45 – 3.22 (m, 2H), 2.62 – 2.43 (m, 2H), 1.91 – 1.72 (m, 2H), 1.67 – 1.48 (m, 4H), 1.36 – 1.15 (m, 10H), 1.00 – 0.75 (m, 3H).

¹³C NMR (101 MHz, Chloroform-*d*) δ 172.1, 148.2, 138.4, 136.6, 134.6, 128.1, 127.6, 121.7, 121.5, 116.6, 68.2, 41.5, 35.4, 33.7, 33.6, 33.6, 33.5, 32.5, 29.5, 28.8, 23.2, 14.2.

HRMS (ESI) calculated for $C_{23}H_{33}N_2O_2^+$ $[M+H]^+$: 369.2537, found: 369.2545.

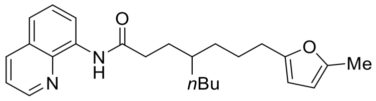

4-(3-(5-methylfuran-2-yl)propyl)-*N*-(quinolin-8-yl)octanamide (**4ja**)

Following general procedure 1. The product was isolated by column chromatography, as brown oil (60.3 mg, 77%, r.r. > 20:1).

Rf (petroleum ether : ethyl acetate = 4:1) = 0.60

¹H NMR (500 MHz, Chloroform-*d*) δ 9.81 (s, 1H), 8.88 – 8.59 (m, 2H), 8.15 (dq, *J* = 8.3, 1.7 Hz, 1H), 7.66 – 7.33 (m, 3H), 5.98 – 5.44 (m, 2H), 2.64 – 2.44 (m, 4H), 2.24 (s, 3H), 1.85 – 1.76 (m, 2H), 1.74 – 1.61 (m, 2H), 1.52 – 1.43 (m, 1H), 1.41 – 1.34 (m, 2H), 1.34 – 1.23 (m, 6H), 0.95 – 0.79 (m, 3H).

¹³C NMR (126 MHz, Chloroform-*d*) δ 172.2, 154.6, 150.2, 148.2, 138.4, 136.6, 134.7, 128.1, 127.6, 121.7, 121.4, 116.6, 105.9, 105.4, 37.1, 35.6, 33.1, 33.0, 29.4, 28.9, 28.5, 25.3, 23.2, 14.3, 13.6.

HRMS (ESI) calculated for $C_{25}H_{33}N_2O_2^+$ $[M+H]^+$: 393.2537, found: 393.2553.

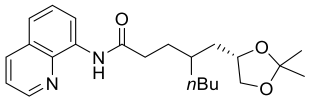

4-(((*S*)-2,2-dimethyl-1,3-dioxolan-4-yl)methyl)-*N*-(quinolin-8-yl)octanamide (**4ka**)

Following general procedure 1. The product was isolated by column chromatography, as colorless oil (51.5 mg, 67%, r.r. > 20:1, 2.8:1 d.r.).

Rf (petroleum ether : ethyl acetate = 3:1) = 0.40

¹H NMR (500 MHz, Chloroform-*d*) δ 9.83 (s, 1H), 8.98 – 8.56 (m, 2H), 8.17 (dt, *J* = 8.3, 1.8 Hz, 1H), 7.72 – 7.30 (m, 3H), 4.22 (qd, *J* = 7.5, 5.5 Hz, 1H), 4.14 – 4.02 (m, 1H), 3.49 (q, *J* = 7.5 Hz, 1H), 2.63 – 2.50 (m, 2H), 1.94 – 1.80 (m, 2H), 1.77 – 1.65 (m, 1H), 1.64 – 1.55 (m, 1H), 1.53 – 1.44 (m, 1H), 1.41 (s, 3H), 1.39 – 1.28 (m, 9H), 0.96 – 0.82 (m, 3H).

^{13}C NMR (126 MHz, Chloroform-*d*) δ 172.0, 148.1, 138.4, 136.7, 134.6, 128.1, 127.6, 121.7, 121.5, 116.7, 108.8, 74.7, 74.3, 70.1, 37.9, 37.8, 35.6, 35.4, 34.9, 34.6, 33.6, 33.3, 29.7, 29.4, 28.7, 28.7, 27.2, 26.0, 23.2, 23.1, 14.2.

HRMS (ESI) calculated for $C_{23}H_{33}N_2O_3^+$ $[M+H]^+$: 385.2486, found: 385.2494.

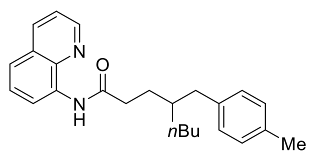

4-(4-methylbenzyl)-*N*-(quinolin-8-yl)octanamide (**4la**)

Following general procedure 1. The product was isolated by column chromatography, as colorless oil (56.9 mg, 76%, r.r. = 9.0:1).

Rf (petroleum ether : ethyl acetate = 4:1) = 0.60

^{1}H NMR (400 MHz, Chloroform-*d*) δ 9.79 (s, 1H), 8.99 – 8.70 (m, 2H), 8.16 (dd, *J* = 8.2, 1.7 Hz, 1H), 7.66 – 7.37 (m, 3H), 7.08 (s, 4H), 2.68 – 2.46 (m, 4H), 2.30 (s, 3H), 1.91 – 1.70 (m, 3H), 1.41 – 1.22 (m, 6H), 0.89 (t, *J* = 7.1 Hz, 2.71H), 0.82 (t, *J* = 7.2 Hz, 0.3H).

^{13}C NMR (126 MHz, Chloroform-d) δ 172.1, 148.1, 138.4, 138.1, 136.6, 135.2, 134.7, 129.2, 129.0, 128.1, 127.6, 121.6, 121.4, 116.6, 40.0, 39.5, 35.8, 32.9, 29.2, 28.8, 23.1, 21.1, 14.2.

HRMS (ESI) calculated for $C_{25}H_{31}N_2O^+$ $[M+H]^+$: 375.2431, found: 375.2432.

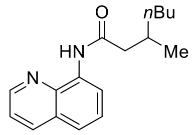

3-methyl-*N*-(quinolin-8-yl)heptanamide (**4ma**)

Following general procedure 1. The product was isolated by column chromatography, as colorless oil (36.2 mg, 67%, r.r. = 16:1).

Rf (petroleum ether : ethyl acetate = 5:1) = 0.60

^{1}H NMR (400 MHz, Chloroform-*d*) δ 9.80 (s, 1H), 8.80 (dt, *J* = 5.2, 1.5 Hz, 2H), 8.16 (dd, *J* = 8.3, 1.7 Hz, 1H), 7.69 – 7.37 (m, 3H), 2.57 (dd, *J* = 14.2, 6.1 Hz, 1H), 2.35 (dd, *J* = 14.2, 8.2 Hz, 1H), 2.25 – 2.11 (m, 1H), 1.50 – 1.40 (m, 1H), 1.40 – 1.21 (m, 5H), 1.05 (d, *J* = 6.7 Hz, 3H), 0.94 – 0.80 (m, 3H).

^{13}C NMR (101 MHz, Chloroform-*d*) δ 171.6, 148.2, 138.4, 136.6, 134.7, 128.1, 127.6, 121.7, 121.5, 116.6, 46.1, 36.7, 31.0, 29.4, 23.0, 19.9, 14.2.

HRMS (ESI) calculated for $C_{17}H_{23}N_2O^+$ $[M+H]^+$: 271.1805, found: 271.1818.

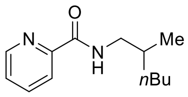

N-(2-methylhexyl)picolinamide (**4na**)

Following general procedure 1. The product was isolated by column chromatography, as brown oil (37.0 mg, 84%, r.r. = 4.8:1).

Rf (petroleum ether : ethyl acetate = 4:1) = 0.50

^{1}H NMR (400 MHz, Chloroform-*d*) δ 8.54 (ddd, *J* = 4.7, 1.8, 0.9 Hz, 1H), 8.20 (dt, *J* = 7.8, 1.1 Hz, 1H), 8.16 – 8.03 (m, 1H), 7.84 (td, *J* = 7.7, 1.7 Hz, 1H), 7.41 (ddd, *J* = 7.6, 4.8, 1.3 Hz, 1H), 3.50 – 3.37 (m, 1H), 3.27 (ddd, *J* = 13.4, 7.3, 6.3 Hz, 1H), 1.85 – 1.70 (m, 1H), 1.48 – 1.13 (m, 6.5H), 0.97 (d, *J* = 6.7 Hz, 2.5H), 0.89 (t, *J* = 6.8 Hz, 3H).

^{13}C NMR (126 MHz, Chloroform-*d*) δ 164.4, 150.2, 148.1, 137.5, 126.1, 122.4, 45.6, 34.3, 33.6, 29.3, 23.0, 17.9, 14.2.

HRMS (ESI) calculated for $C_{13}H_{21}N_2O^+$ $[M+H]^+$: 221.1648, found: 221.1652.

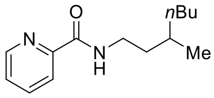

N-(3-methylheptyl)picolinamide (**4oa**)

Following general procedure 1. The product was isolated by column chromatography, as brown oil (23.5 mg, 51%, r.r. = 4.4:1).

Rf (petroleum ether : ethyl acetate = 4:1) = 0.50

^{1}H NMR (400 MHz, Chloroform-*d*) δ 8.57 – 8.44 (m, 1H), 8.20 (dt, *J* = 7.8, 1.1 Hz, 1H), 8.03 (s, 1H), 7.84 (td, *J* = 7.7, 1.7 Hz, 1H), 7.41 (ddd, *J* = 7.6, 4.8, 1.2 Hz, 1H), 3.58 – 3.36 (m, 2H), 1.73 – 1.60 (m, 1H), 1.58 – 1.51 (m, 1H), 1.51 – 1.41 (m, 1H), 1.39 – 1.23 (m, 6.7H), 0.95 (d, *J* = 6.5 Hz, 2.45H), 0.94 – 0.82 (m, 3H).

^{13}C NMR (126 MHz, Chloroform-*d*) δ 164.2, 150.2, 148.1, 137.5, 126.1, 122.3, 39.6, 37.7, 36.8, 36.7, 31.9, 30.8, 29.8, 29.4, 29.3, 29.2, 27.1, 23.1, 22.8, 19.7, 14.2, 14.2.

HRMS (ESI) calculated for $C_{14}H_{23}N_2O^+$ $[M+H]^+$: 235.1805, found: 235.1817.

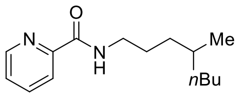

N-(4-methyloctyl)picolinamide (**4pa**)

Following general procedure 1. The product was isolated by column chromatography, as brown oil (42.2 mg, 85%, r.r. = 4.0:1).

Rf (petroleum ether : ethyl acetate = 4:1) = 0.50

^{1}H NMR (400 MHz, Chloroform-*d*) δ 8.54 (ddd, *J* = 4.8, 1.8, 0.9 Hz, 1H), 8.20 (dt, *J* = 7.9, 1.1 Hz, 1H), 8.07 (s, 1H), 7.84 (tt, *J* = 7.8, 1.3 Hz, 1H), 7.41 (ddt, *J* = 7.5, 4.8, 1.1 Hz, 1H), 3.53 – 3.39 (m, 2H), 1.72 – 1.52 (m, 2H), 1.47 – 1.34 (m, 2H), 1.32 – 1.06 (m, 7.72H), 0.90 – 0.80 (m, 5.38H).

^{13}C NMR (101 MHz, Chloroform-*d*) δ 164.2, 150.2, 148.0, 137.5, 126.1, 122.4, 40.0, 39.6, 36.7, 34.4, 32.7, 32.0, 29.8, 29.6, 29.5, 29.4, 29.4, 27.4, 27.1, 23.1, 22.8, 19.7, 14.3, 14.2.

HRMS (ESI) calculated for $C_{15}H_{25}N_2O^+$ [M+H]$^+$: 249.1962, found: 249.1972.

Asymmetric Proximal-selective Hydroalkylation of Unactivated Alkenes

Supplementary Table 2. Optimization of the Asymmetric Proximal-selective Hydroalkylation of Unactivated Alkenes

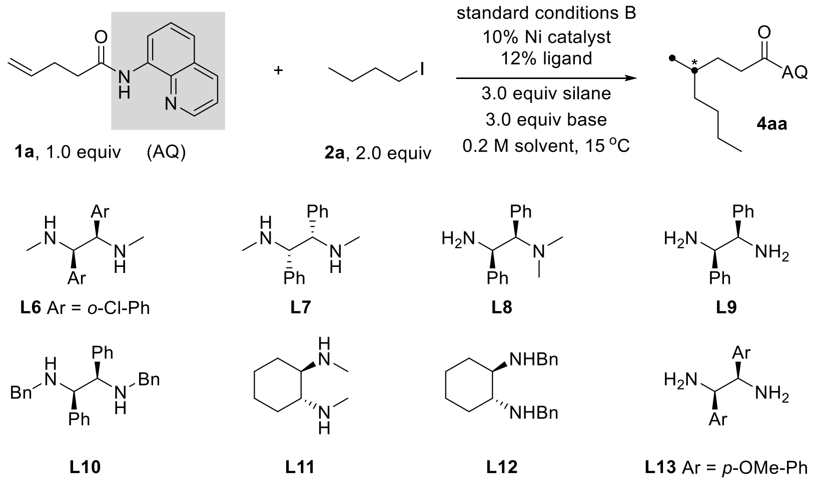

entry	Ni catalyst	ligand	silane	base	solvent	Yield/%[a]	e.e./%[b]	r.r.[a]
1	$NiBr_2$(diglyme)	L6	DEMS	Na_2CO_3	DMAc	21	80	20:1
2	$NiBr_2$(diglyme)	**L7**	DEMS	Na_2CO_3	DMAc	36	-40	20:1
3	$NiBr_2$(diglyme)	**L8**	DEMS	Na_2CO_3	DMAc	62	10	7.2:1
4	$NiBr_2$(diglyme)	**L9**	DEMS	Na_2CO_3	DMAc	77	<2%	3.3:1
5	$NiBr_2$(diglyme)	**L10**	DEMS	Na_2CO_3	DMAc	52	10	8.9:1
6	$NiBr_2$(diglyme)	**L11**	DEMS	Na_2CO_3	DMAc	78	12	12:1
7	$NiBr_2$(diglyme)	**L12**	DEMS	Na_2CO_3	DMAc	49	0	9.3:1
8	$NiBr_2$(diglyme)	**L13**	DEMS	Na_2CO_3	DMAc	51	0	3.7:1
9	$NiCl_2$(DME)	**L6**	DEMS	Na_2CO_3	DMAc	59	53	13:1
10	NiI_2	**L6**	DEMS	Na_2CO_3	DMAc	20	71	>20:1

11	$NiBr_2$(diglyme)	**L6**	$(MeO)_3SiH$	Na_2CO_3	DMAc	60	23	15:1
12	$NiBr_2$(diglyme)	**L6**	Ph_2SiH_2	Na_2CO_3	DMAc	9	-	-
13	$NiBr_2$(diglyme)	**L6**	DMMS	Na_2CO_3	DMAc	31	50	>20:1
14	$NiBr_2$(diglyme)	**L6**	DEMS	KF	DMAc	84	20	5.7:1
15	$NiBr_2$(diglyme)	**L6**	DEMS	CsF	DMAc	78	23	5.1:1
16	$NiBr_2$(diglyme)	**L6**	DEMS	K_2CO_3	DMAc	24	63	14:1
17	$NiBr_2$(diglyme)	**L6**	DEMS	$KHCO_3$	DMAc	28	53	19:1
18	$NiBr_2$(diglyme)	**L6**	DEMS	Na_2CO_3	CH_3CN	9	31	4.3:1
19	$NiBr_2$(diglyme)	**L6**	DEMS	Na_2CO_3	DMSO	39	37	14:1
20	$NiBr_2$(diglyme)	**L6**	DEMS	Na_2CO_3	DMF	27	76	>20:1
21	$NiBr_2$(diglyme)	**L6**	DEMS	Na_2CO_3	DMAc/DCE (v:v = 1:4)	18	48	14:1
22	$NiBr_2$(diglyme)	**L6**	DEMS	Na_2CO_3	DMAc/Toluene (v:v = 1:4)	21	70	16:1
23	$NiBr_2$(diglyme)	**L6**	$(MeO)_3SiH$	Na_2CO_3	DMAc/2-Me-THF (v:v = 1:4)	36	58	11:1

Reactions were carried out under an argon atmosphere. [a]Yields and regioisomeric ratios were determined by GC analysis with triphenylmethane as an internal standard. Total yield for the mixture of all regioisomers. r.r. refers to the regioisomeric ratio, that of the major product to the sum of all other isomers. Proximal-selective hydroalkylation product **4aa** was obtained as the major regioisomer, and distal-selective hydroalkylation product **4aa'** as the minor regioisomer; other regioisomers could hardly be detected. [b]The e.e. value was determined by high-performance liquid chromatography (HPLC). DEMS = diethoxymethylsilane. DMMS = dimethoxy(methyl)silane. Diglyme = 2-methoxyethyl ether. DMAc = *N*,*N*-dimethylacetamide. DMF = *N*,*N*-dimethylformamide. DCE = 1,2-dichloroethane. 2-Me-THF = 2-methyltetrahydrofuran.

Supplementary Table 3. Effect of Reaction Temperature

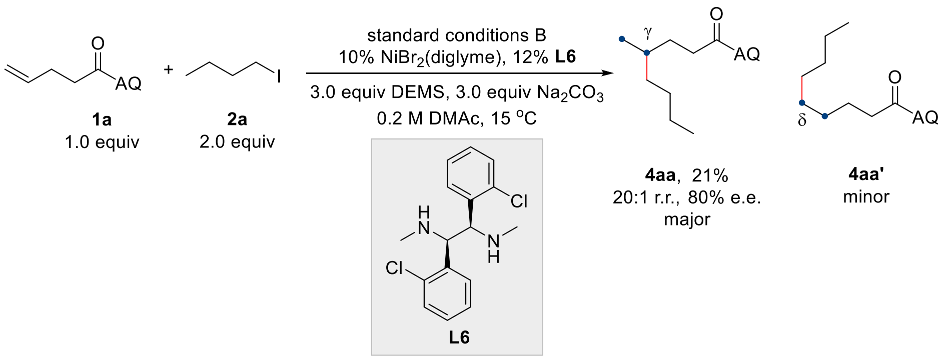

entry	variation from standard conditions B	yield/%[a]	r.r.[a]	e.e./%[b]
1	none	21	20:1	80

2	5 °C	19	20:1	70
3	40 °C	64	20:1	72
4	60 °C	61	20:1	58

Reactions were carried out under an argon atmosphere. [a]Yields and regioisomeric ratios were determined by GC analysis with triphenylmethane as an internal standard. Total yield for the mixture of all regioisomers. r.r. refers to the regioisomeric ratio, that of the major product to the sum of all other isomers. Proximal-selective hydroalkylation product **4aa** was obtained as the major regioisomer, and distal-selective hydroalkylation product **4aa'** as the minor regioisomer; other regioisomers could hardly be detected. [b]The e.e. value was determined by high-performance liquid chromatography (HPLC).

In air, a 10 mL Schlenk tube equipped with a stir bar was charged with Ni catalyst (0.01 mmol, 10 mol%), ligand (0.012 mmol, 12 mol%), base (0.30 mmol, 3.0 equiv), and alkene (0.10 mmol, 1.0 equiv). The Schlenk tube was evacuated and filled with argon (three cycles). To these solids, solvent (0.50 mL), alkyl halide (0.20 mmol, 2.0 equiv), silane (0.30 mmol, 3.0 equiv) were added under an argon atmosphere. The mixture was stirred at 15 °C for 12 h. The GC yield was determined using triphenylmethane as an internal standard.

Determination of Regioisomeric Ratio in Standard Conditions B

For terminal alkene **1a** under asymmetric catalytic conditions, the outcome was similar to terminal alkene **1a** under standard conditions A.

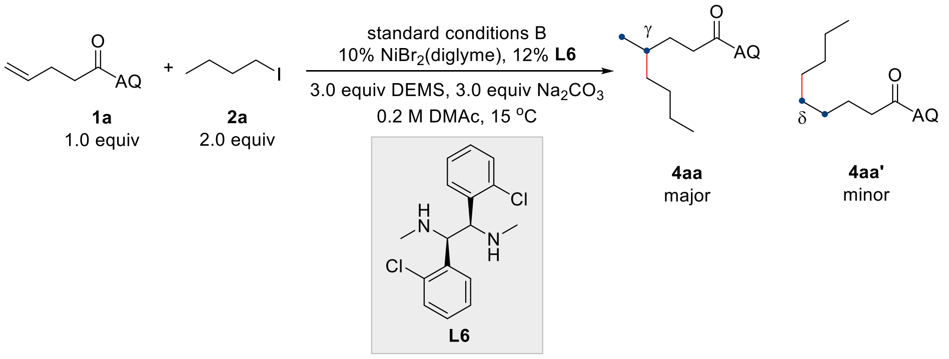

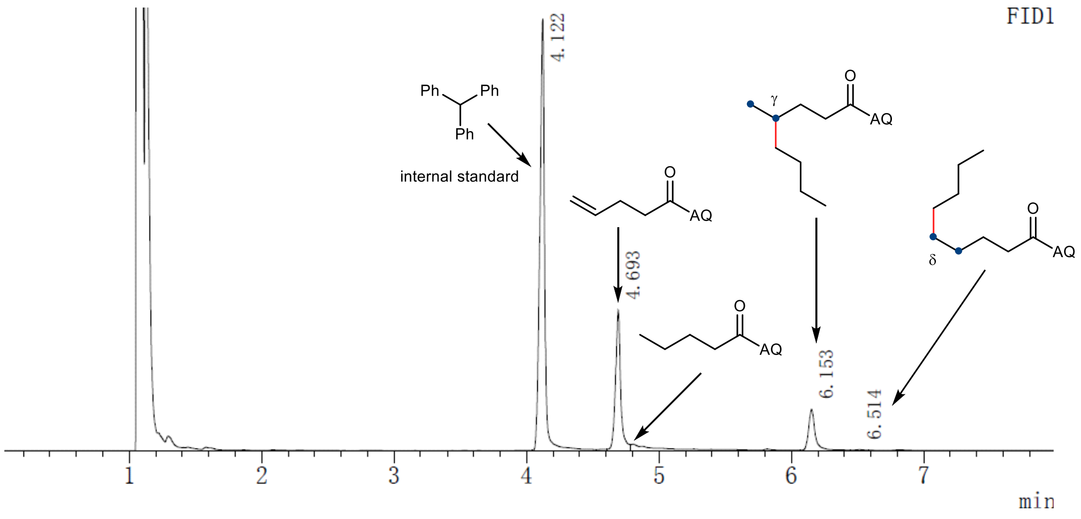

Supplementary Figure 6. GC spectra of **4aa** under asymmetric catalytic conditions B

Preliminary Results of Asymmetric Synthesis

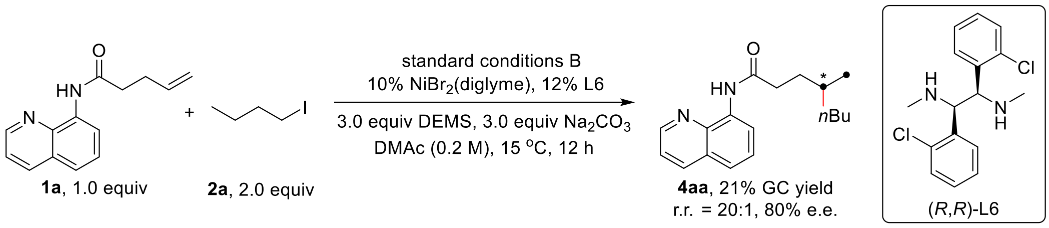

In air, a 10 mL Schlenk tube equipped with a stir bar was charged with $NiBr_2$(diglyme) (3.5 mg, 0.01 mmol, 10 mol%), **L6** (3.7 mg, 0.012 mmol, 12 mol%), *N*-(quinolin-8-yl)pent-4-enamide (**1a**, 22.6 mg, 0.10 mmol, 1.0 equiv), Na_2CO_3 (32.0 mg, 0.30 mmol, 3.0 equiv). The Schlenk tube was evacuated and filled with argon (three cycles). To these solids, anhydrous DMAc (0.5 mL) was added under an argon atmosphere. Then, 1-iodobutane (36.8 mg, 0.20 mmol, 2.0 equiv), DEMS (40.3 mg, 0.30 mmol, 3.0 equiv) were added. The mixture was stirred at 15 °C for 12 h, and diluted with H_2O and EA. Yield and r.r. were determined by GC analysis with triphenylmethane as an internal standard.

HPLC analysis: The e.e. was determined to be 80% on a CHIRALCEL OD-H column (2% *i*PrOH in hexane, 1.0 mL/min, 40 ºC); retention times for compound obtained using **L6**: 29.2 min (major), 33.6 min (minor).

HPLC data of rac-4aa

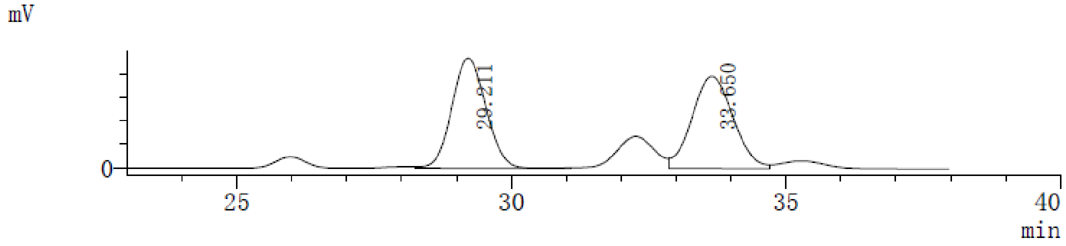

检测器A Ch1 214nm

Peak[#]	RetTime[min]	Height[uV]	Width[min]	Area[uV*s]	Area[%]
1	29. 211	94725	0. 674	4109694	49. 868
2	33. 650	79340	0. 813	4131424	50. 132

HPLC data of 4aa using (R, R)-L6

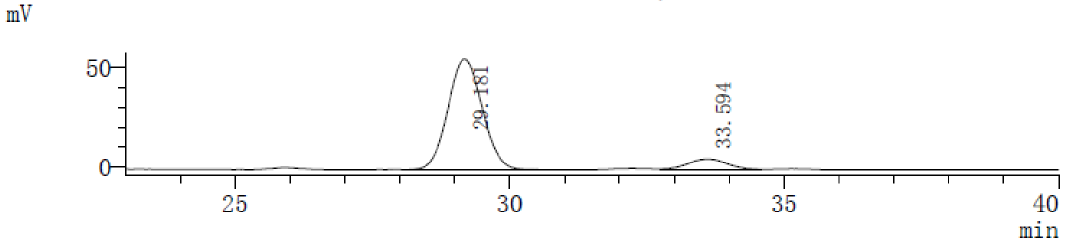

检测器A Ch1 214nm

Peak[#]	RetTime[min]	Height[uV]	Width[min]	Area[uV*s]	Area[%]
1	29. 181	55268	0. 677	2389752	89. 988
2	33. 594	5223	0. 796	265897	10. 012

Supplementary Figure 7. HPLC spectra of **4aa**

Migratory Hydroalkylation of Unactivated Alkenes

Supplementary Table 4. Optimization of the Migratory Hydroalkylation of Unactivated Alkenes.

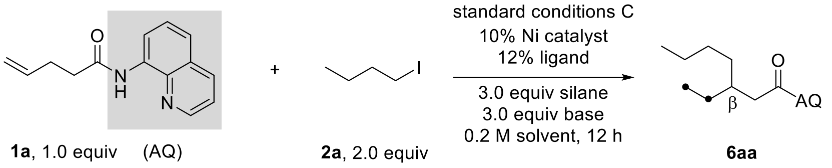

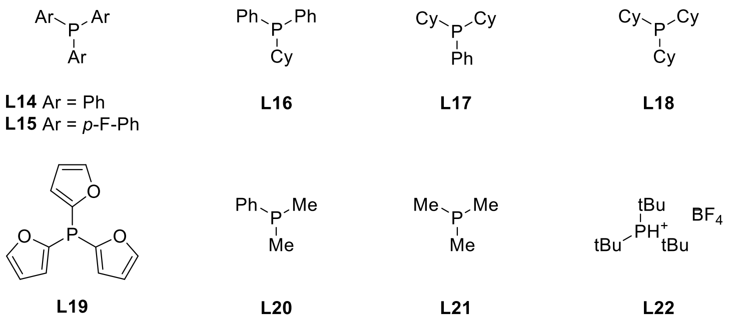

entry	Ni catalyst	ligand	silane	base	solvent	Temp./°C	Yield/%[a]	r.r.[b]
1[d]	$NiCl_2(PPh_3)_2$	--	$(MeO)_3SiH$	KF	DMAc	100	50 (42[c])	19:1
2	$NiBr_2$(diglyme)	**L14**	$(MeO)_3SiH$	KF	DMAc	25	76	2.6:1
3	$NiBr_2$(diglyme)	**L15**	$(MeO)_3SiH$	KF	DMAc	25	83	1.9:1
4	$NiBr_2$(diglyme)	**L16**	$(MeO)_3SiH$	KF	DMAc	25	78	1.8:1
5	$NiBr_2$(diglyme)	**L17**	$(MeO)_3SiH$	KF	DMAc	25	87	1:1.5
6	$NiBr_2$(diglyme)	**L18**	$(MeO)_3SiH$	KF	DMAc	25	67	1:2.3
7	$NiBr_2$(diglyme)	**L19**	$(MeO)_3SiH$	KF	DMAc	25	71	1:2.7
8	$NiBr_2$(diglyme)	**L20**	$(MeO)_3SiH$	KF	DMAc	25	<2	--
9	$NiBr_2$(diglyme)	**L21**	$(MeO)_3SiH$	KF	DMAc	25	42	3.2:1
10	$NiBr_2$(diglyme)	**L22**	$(MeO)_3SiH$	KF	DMAc	25	70	1:2.2
11	$NiBr_2$(diglyme)	**L14**	DEMS	KF	DMAc	25	61	1.1:1
12	$NiBr_2$(diglyme)	**L14**	$(EtO)_3SiH$	KF	DMAc	25	71	2.2:1
13	$NiBr_2$(diglyme)	**L14**	Ph_2SiH_2	KF	DMAc	100	<2	--
14	$NiBr_2$(diglyme)	**L14**	$(MeO)_3SiH$	Na_2CO_3	DMAc	25	68	1:1.4
15	$NiBr_2$(diglyme)	**L14**	$(MeO)_3SiH$	NaF	DMAc	25	30	>20:1
16	$NiBr_2$(diglyme)	**L14**	$(MeO)_3SiH$	CsF	DMAc	100	36	6.3:1
17	$NiBr_2$(diglyme)	**L14**	$(MeO)_3SiH$	KF	DMF	100	61	1.8:1
18	$NiBr_2$(diglyme)	**L14**	$(MeO)_3SiH$	KF	NMP	100	28	>20:1
19[d]	$NiBr_2$(diglyme)	**L14**	$(MeO)_3SiH$	KF	DMAc	100	59	5.2:1

Reactions were carried out under an argon atmosphere. Conditions: **1a** (0.10 mmol, 1.0 equiv), **2a** (0.20 mmol, 2.0 equiv), nickel catalyst (0.01 mmol, 10 mol%), ligand (0.012 mmol, 12 mol%), silane (0.30 mmol, 3.0 equiv), base (0.30 mmol, 3.0 equiv), solvent (0.50 mL, 0.2 M), 12 h. [a]Yields and regioisomeric ratios were determined by GC analysis with triphenylmethane as an internal standard. Total yield for the mixture of all regioisomers. [b]r.r. refers to the regioisomeric ratio, that of the major product to the sum of all other isomers. In most cases, a mixture of β- and γ-selective products was obtained; we could hardly observe other isomers. [c]Isolated yield in parentheses. [d]Ratio of 1a:2a = 2:1. DEMS = diethoxymethylsilane. Diglyme = 2-methoxyethyl ether. DMAc = *N*,*N*-dimethylacetamide. DMF = *N*,*N*-dimethylformamide. NMP = 1-Methyl-2-pyrrolidinone.

In air, a 10 mL Schlenk tube equipped with a stir bar was charged with Ni catalyst (0.01 mmol, 10 mol%), ligand (0.012 mmol, 12 mol%), base (0.30 mmol, 3.0 equiv), and alkene (0.10 mmol, 1.0 equiv). The Schlenk tube was evacuated and filled with argon (three cycles). To these solids, solvent (0.50 mL), alkyl halide (0.20 mmol, 2.0 equiv), silane (0.30 mmol, 3.0 equiv) were added under argon atmosphere. The mixture was stirred for 12 h. The GC yield was determined using triphenylmethane as an internal standard.

Determination of Regioisomeric Ratio in Standard Conditions C

For terminal alkene **1a** under migratory β-selective conditions, we obtained β-selective hydroalkylation product **6aa** as the major regioisomer, and γ-hydroalkylation product **6aa’** as the minor regioisomer; other regioisomers were not observed (lower than the detection limit of gas chromatography).

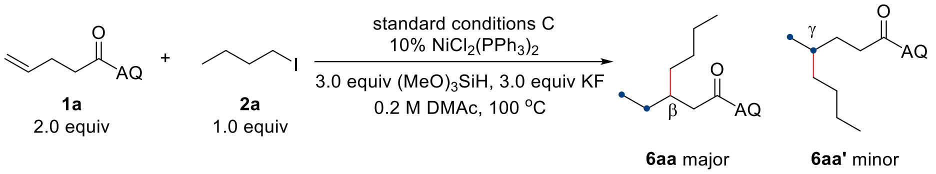

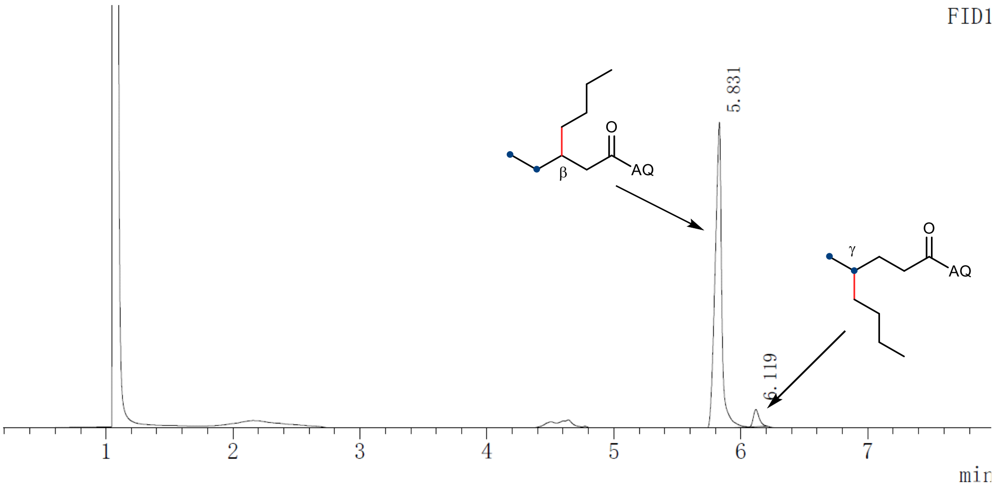

Supplementary Figure 8. GC spectra of **6aa** under standard conditions C

General Procedure 2 for the Synthesis of Migratory Alkylation Product

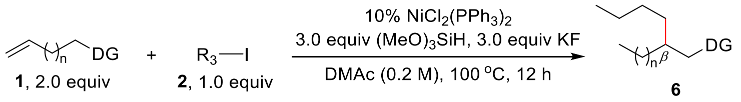

In air, a 10 mL Schlenk tube equipped with a stir bar was charged with $NiCl_2(PPh_3)_2$ (0.02 mmol, 10 mol%), KF (0.60 mmol, 3.0 equiv), alkene (0.40 mmol, 2.0 equiv), and alkyl halide (0.20 mmol, 1.0 equiv). The Schlenk tube was evacuated and filled with argon (three cycles). To these solids, anhydrous DMAc (1.0 mL) was added under an argon atmosphere. Then, $(MeO)_3SiH$ (0.60 mmol, 3.0 equiv) (if the alkene or alkyl halide was liquid, it was also added at this time) was added under an argon atmosphere. The mixture was stirred at 100 °C for 12 h, diluted with H_2O followed by extraction with ethyl acetate, dried with anhydrous Na_2SO_4, and concentrated in vacuo. The residue was purified by column chromatography to produce the target product.

Regioisomeric ratios (r.r.) were determined by GC analysis.

Examples Described in Figure 4f

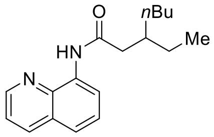

3-ethyl-*N*-(quinolin-8-yl)heptanamide (**6aa**)

Following general procedure 2. The product was isolated by column chromatography, as colorless oil (23.9 mg, 42%, r.r. = 19:1).

Rf (petroleum ether : ethyl acetate = 5:1) = 0.50

^{1}H NMR (500 MHz, Chloroform-*d*) δ 9.82 (s, 1H), 8.97 – 8.62 (m, 2H), 8.16 (d, *J* = 8.2 Hz, 1H), 7.64 – 7.39 (m, 3H), 2.49 (d, *J* = 7.0 Hz, 2H), 2.10 – 1.95 (m, 1H), 1.53 – 1.37 (m, 4H), 1.37 – 1.22 (m, 4H), 0.94 (t, *J* = 7.4 Hz, 3H), 0.88 (t, *J* = 6.8 Hz, 3H).

^{13}C NMR (126 MHz, Chloroform-*d*) δ 171.9, 148.1, 138.3, 136.6, 134.7, 128.1, 127.6, 121.7, 121.4, 116.6, 43.0, 37.0, 33.1, 29.0, 26.4, 23.1, 14.2, 11.0.

HRMS (ESI) calculated for $C_{18}H_{25}N_2O^+$ $[M+H]^+$: 285.1962, found: 285.1970.

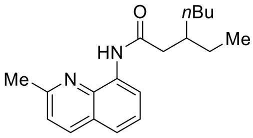

3-ethyl-*N*-(2-methylquinolin-8-yl)heptanamide (**6ra**)

Following general procedure 2. The product was isolated by column chromatography, as colorless oil (26.9 mg, 45%, r.r. > 20:1).

Rf (petroleum ether : ethyl acetate = 5:1) = 0.50

^{1}H NMR (500 MHz, Chloroform-*d*) δ 9.87 (s, 1H), 8.75 (dd, *J* = 6.7, 2.2 Hz, 1H), 8.02 (d, *J* = 8.4 Hz, 1H), 7.60 – 7.38 (m, 2H), 7.31 (d, *J* = 8.4 Hz, 1H), 2.74 (s, 3H), 2.49 (d, *J* = 6.9 Hz, 2H), 2.07 – 1.95 (m, 1H), 1.54 – 1.43 (m, 4H), 1.39 – 1.26 (m, 4H), 0.96 (t, *J* = 7.4 Hz, 3H), 0.89 (t, *J* = 6.9 Hz, 3H).

^{13}C NMR (126 MHz, Chloroform-*d*) δ 171.8, 157.2, 137.8, 136.5, 134.1, 126.5, 126.1, 122.5, 121.2, 116.4, 43.0, 37.0, 33.2, 29.0, 26.4, 25.4, 23.1, 14.2, 11.0.

HRMS (ESI) calculated for $C_{19}H_{27}N_2O^+$ $[M+H]^+$: 299.2118, found: 299.2130.

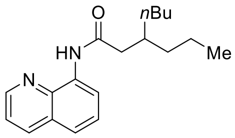

3-propyl-*N*-(quinolin-8-yl)heptanamide (**6qa**)

Following general procedure 2. The product was isolated by column chromatography, as colorless oil (20.9 mg, 35%, r.r. = 14:1).

Rf (petroleum ether : ethyl acetate = 5:1) = 0.50

^{1}H NMR (500 MHz, Chloroform-*d*) δ 9.81 (s, 1H), 8.98 – 8.62 (m, 2H), 8.16 (dd, *J* = 8.3, 1.7 Hz, 1H), 7.67 – 7.38 (m, 3H), 2.49 (d, *J* = 6.9 Hz, 2H), 2.14 – 2.00 (m, 1H), 1.50 – 1.28 (m, 10H), 1.00 – 0.81 (m, 6H).

^{13}C NMR (126 MHz, Chloroform-*d*) δ 171.9, 148.2, 138.4, 136.5, 134.7, 128.1, 127.6, 121.7, 121.4, 116.6, 43.4, 36.3, 35.4, 33.7, 28.9, 23.1, 19.9, 14.5, 14.2.

HRMS (ESI) calculated for $C_{19}H_{27}N_2O^+$ $[M+H]^+$: 299.2118, found: 299.2131.

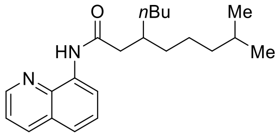

3-butyl-7-methyl-*N*-(quinolin-8-yl)octanamide (**6ca**)

Following general procedure 2. The product was isolated by column chromatography, as colorless oil (24.5 mg, 36%, r.r. = 15:1).

Rf (petroleum ether : ethyl acetate = 5:1) = 0.45

^{1}H NMR (400 MHz, Chloroform-*d*) δ 9.81 (s, 1H), 8.93 – 8.68 (m, 2H), 8.16 (dd, *J* = 8.3, 1.7 Hz, 1H), 7.69 – 7.34 (m, 3H), 2.49 (d, *J* = 6.9 Hz, 2H), 2.18 – 1.99 (m, 1H), 1.63 – 1.47 (m, 1H), 1.44 – 1.28 (m, 10H), 1.21 – 1.07 (m, 2H), 0.93 – 0.81 (m, 9H).

^{13}C NMR (126 MHz, Chloroform-*d*) δ 171.9, 148.2, 138.4, 136.5, 134.7, 128.1, 127.6, 121.7, 121.4, 116.6, 43.5, 39.4, 35.6, 34.2, 33.7, 29.0, 28.1, 24.5, 23.1, 22.8, 22.7, 14.2.

HRMS (ESI) calculated for $C_{22}H_{33}N_2O^+$ $[M+H]^+$: 341.2588, found: 341.2591.

Synthetic Applications

Late-stage Functionalization of Biorelevant Molecules

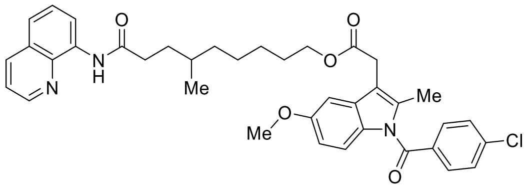

6-methyl-9-oxo-9-(quinolin-8-ylamino)nonyl 2-(1-(4-chlorobenzoyl)-5-methoxy-2-methyl-1*H*-indol-3-yl)acetate (**4au**)

Following general procedure 1. The product was isolated by column chromatography, as colorless oil (75.9 mg, 58%, r.r. = 9.5:1).

Rf (petroleum ether : ethyl acetate = 2:1) = 0.60

^{1}H NMR (400 MHz, Chloroform-*d*) δ 9.82 (s, 1H), 8.91 – 8.67 (m, 2H), 8.16 (dd, *J* = 8.3, 1.7 Hz, 1H), 7.70 – 7.60 (m, 2H), 7.57 – 7.47 (m, 2H), 7.45 (dt, *J* = 8.6, 2.1 Hz, 3H), 6.96 (d, *J* = 2.6 Hz, 1H), 6.87 (d, *J* = 9.0 Hz, 1H), 6.67 (dd, *J* = 9.0, 2.5 Hz, 1H), 4.09 (t, *J* = 6.7 Hz, 2H), 3.83 (s, 3H), 3.65 (s, 2H), 2.65 – 2.47 (m, 2H), 2.38 (s, 3H), 1.89 – 1.78 (m, 1H), 1.73 – 1.57 (m, 4H), 1.55 – 1.47 (m, 1H), 1.39 – 1.22 (m, 4H), 1.19 – 1.06 (m, 1H), 0.94 (d, *J* = 6.5 Hz, 3H).

^{13}C NMR (101 MHz, Chloroform-*d*) δ 172.2, 171.1, 168.4, 156.2, 148.1, 139.3, 138.3, 136.7, 136.0, 134.6, 134.1, 131.3, 130.9, 130.8, 129.2, 128.1, 127.6, 121.7, 121.5, 116.7, 115.1, 112.9, 111.8, 101.4, 65.3, 55.8, 36.8, 36.0, 32.6, 32.6, 30.6, 28.8, 26.7, 26.3, 19.5, 13.5.

HRMS (ESI) calculated for $C_{38}H_{41}ClN_3O_4^+$ $[M+H]^+$: 654.2729, found: 654.2743.

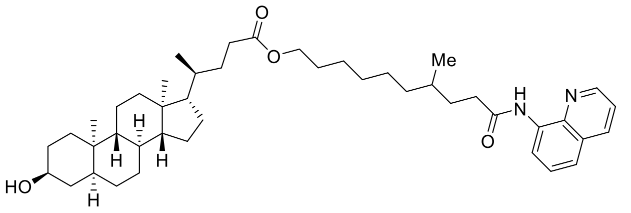

7-methyl-10-oxo-10-(quinolin-8-ylamino)decyl (4*S*)-4-((3*S*,5*S*,8*S*,9*R*,10*R*,13*S*,14*R*,17*S*)-3-hydroxy-10,13-dimethylhexadecahydro-1*H*-cyclopenta[*a*]phenanthren-17-yl)pentanoate (**4av**)

Following general procedure 1. The product was isolated by column chromatography, as colorless oil (75.6 mg, 55%, r.r. > 20:1, 1.0:1 d.r.).

Rf (petroleum ether : ethyl acetate = 3:1) = 0.30

^{1}H NMR (400 MHz, Chloroform-*d*) δ 9.81 (s, 1H), 8.89 – 8.70 (m, 2H), 8.16 (dd, *J* = 8.3, 1.8 Hz, 1H), 7.66 – 7.38 (m, 3H), 4.03 (t, *J* = 6.7 Hz, 2H), 3.66 – 3.53 (m, 1H), 2.66 – 2.44 (m, 2H), 2.40 – 2.26 (m, 1H), 2.25 – 2.13 (m, 1H), 1.89 – 1.70 (m, 8H), 1.68 – 1.45 (m, 8H), 1.45 – 1.19 (m, 20H), 1.13 – 0.99 (m, 4H), 0.95 (d, *J* = 6.4 Hz, 3H), 0.89 (t, *J* = 3.3 Hz, 6H), 0.62 (s, 3H).

^{13}C NMR (126 MHz, Chloroform-*d*) δ 174.6, 172.3, 148.2, 138.4, 136.6, 134.7, 128.1, 127.6, 121.7, 121.5, 116.7, 71.9, 64.5, 56.6, 56.1, 42.8, 42.2, 40.5, 40.3, 36.8, 36.6, 36.0, 35.9, 35.5, 35.5, 34.7, 32.7, 32.7, 31.5, 31.2, 30.7, 29.7, 28.8, 28.3, 27.3, 27.0, 26.5, 26.1, 24.3, 23.5, 20.9, 19.6, 18.4, 12.1.

HRMS (ESI) calculated for $C_{44}H_{67}N_2O_4^+$ $[M+H]^+$: 687.5096, found: 687.5093.

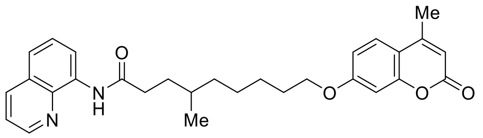

4-methyl-9-((4-methyl-2-oxo-2*H*-chromen-7-yl)oxy)-*N*-(quinolin-8-yl)nonanamide (**4aw**)

Following general procedure 1. The product was isolated by column chromatography, as white solid (78.4 mg, 83%, r.r. > 20:1).

Rf (petroleum ether : ethyl acetate = 3:1) = 0.40

^{1}H NMR (400 MHz, Chloroform-*d*) δ 9.81 (s, 1H), 8.89 – 8.63 (m, 2H), 8.15 (dt, *J* = 8.2, 1.9 Hz, 1H), 7.59 – 7.37 (m, 4H), 6.82 (ddd, *J* = 8.8, 2.6, 1.4 Hz, 1H), 6.77 (q, *J* = 2.6 Hz, 1H), 6.10 (t, *J* = 1.5 Hz, 1H), 3.97 (td, *J* = 6.5, 1.4 Hz, 2H), 2.66 – 2.47 (m, 2H), 2.36 (t, *J* = 1.5 Hz, 3H), 1.93 – 1.84 (m, 1H), 1.84 – 1.73 (m, 2H), 1.70 – 1.50 (m, 2H), 1.50 – 1.39 (m, 4H), 1.37 – 1.32 (m, 1H), 1.30 – 1.16 (m, 1H), 0.96 (d, *J* = 6.4 Hz, 3H).

^{13}C NMR (101 MHz, Chloroform-*d*) δ 172.2, 162.3, 161.5, 155.4, 152.7, 148.2, 138.3, 136.6, 134.6, 128.0, 127.6, 125.5, 121.7, 121.5, 116.5, 113.5, 112.7, 111.9, 101.4, 68.6, 36.7, 36.0, 32.6, 32.5, 29.1, 26.8, 26.3, 19.6, 18.8.

HRMS (ESI) calculated for $C_{29}H_{33}N_2O_4^+$ [M+H]$^+$: 473.2435, found: 473.2449.

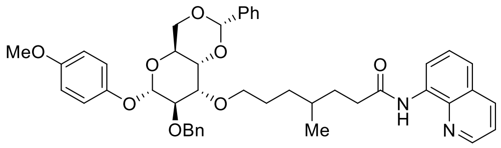

7-(((2*S*,4a*S*,6*S*,7*R*,8*S*,8a*S*)-7-(benzyloxy)-6-(4-methoxyphenoxy)-2-phenylhexahydropyrano[3,2-*d*][1,3]dioxin-8-yl)oxy)-4-methyl-*N*-(quinolin-8-yl)heptanamide (**4ax**)

Following general procedure 1. The product was isolated by column chromatography, as white solid (70.3 mg, 48%, r.r. > 20:1, 1.1:1 d.r.).

Rf (petroleum ether : ethyl acetate = 2:1) = 0.25

^{1}H NMR (400 MHz, Chloroform-*d*) δ 9.78 (s, 1H), 8.78 (ddt, *J* = 7.6, 4.2, 1.8 Hz, 2H), 8.15 (dd, *J* = 8.3, 1.7 Hz, 1H), 7.58 – 7.48 (m, 4H), 7.43 (dd, *J* = 8.3, 4.2 Hz, 1H), 7.39 – 7.26 (m, 8H), 7.10 – 7.01 (m, 2H), 6.84 – 6.76 (m, 2H), 5.56 (d, *J* = 9.2 Hz, 1H), 4.94 (dd, *J* = 10.8, 2.1 Hz, 1H), 4.88 (dd, *J* = 7.8, 3.1 Hz, 1H), 4.83 (dd, *J* = 10.9, 2.7 Hz, 1H), 4.37 – 4.24 (m, 2H), 4.12 – 3.96 (m, 2H), 3.76 (s, 3H), 3.71 (dtd, *J* = 9.2, 6.6, 4.6 Hz, 1H), 3.65 – 3.55 (m, 1H), 3.50 (dt, *J* = 9.7, 3.5 Hz, 1H), 3.47 – 3.40 (m, 1H), 2.58 – 2.39 (m, 2H), 1.88 – 1.78 (m, 1H), 1.76 – 1.51 (m, 5H), 1.49 – 1.35 (m, 1H), 0.93 (d, *J* = 6.3 Hz, 3H).

¹³C NMR (126 MHz, Chloroform-*d*) δ 172.1, 155.4, 151.8, 148.2, 138.9, 138.4, 137.9, 136.5, 134.7, 129.0, 128.4, 128.2, 128.1, 127.6, 127.6, 126.6, 121.7, 121.5, 119.1, 116.6, 114.5, 103.3, 101.5, 80.3, 78.0, 75.5, 73.5, 73.5, 70.5, 70.5, 69.4, 66.8, 55.7, 35.9, 35.8, 33.1, 33.1, 32.5, 32.4, 32.4, 27.5, 27.4, 19.6, 19.5.

HRMS (ESI) calculated for $C_{44}H_{49}N_2O_8^+$ $[M+H]^+$: 733.3484, found: 733.3484.

Gram-scale Reaction and Removal of the Directing Group

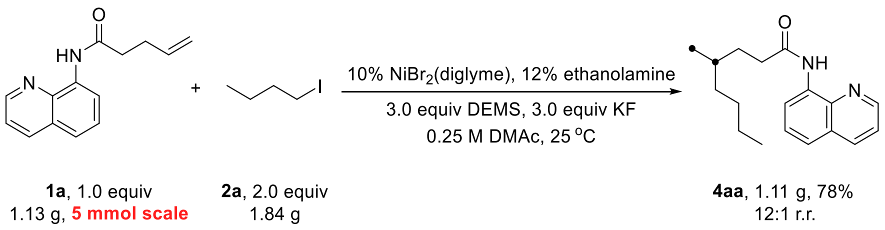

Supplementary Figure 9. Gram scale synthesis of **4aa**

Following general procedure 1, the product **4aa** was synthesized on a 5 mmol scale. The product **4aa** was isolated by column chromatography as colorless oil (1.11 g, 78%, r.r. = 12:1).

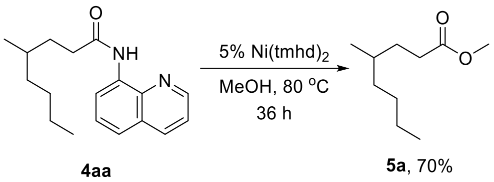

Supplementary Figure 10. Transformation of the directing group

According to the reported literature,[17] **5a** could be readily achieved. Ni(tmhd)$_2$ (5 mol%, 4.3 mg) was added to a Schlenk tube equipped with a stir bar. The Schlenk tube was evacuated and filled with argon (three cycles). To the solids, **4aa** (0.2 mmol, 1.0 equiv, 56.9 mg) and MeOH (1 mL) were added. The reaction mixture was stirred at 80 °C for 36 hours. After cooling to room temperature, the crude mixture was directly purified by flash silica gel column chromatography to provide pure products **5a** as colorless oil (24.1 mg, 70%).

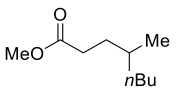

methyl 4-methyloctanoate (**5a**)

¹H NMR (400 MHz, Chloroform-*d*) δ 3.66 (s, 3H), 2.40 – 2.17 (m, 2H), 1.71 – 1.57 (m, 1H), 1.49 – 1.35 (m, 1H), 1.34 – 1.21 (m, 7H), 0.95 – 0.76 (m, 6H).

^{13}C NMR (126 MHz, Chloroform-*d*) δ 174.7, 51.6, 36.5, 32.5, 32.1, 32.0, 29.3, 23.1, 19.4, 14.2.

HRMS (ESI) calculated for $C_{10}H_{21}O_2^+$ $[M+H]^+$: 173.1536, found: 173.1543.

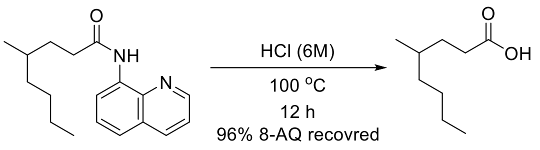

4aa **5b**, 73%

Supplementary Figure 11. Removal of the directing group

A 10 mL Schlenk tube equipped with a stir bar was evacuated and filled with argon (three cycles). **4aa** (0.2 mmol, 1.0 equiv, 56.9 mg) and 6M HCl (4 mL) were added. The reaction mixture was stirred at 100 °C for 12 hours. To the mixture was added dichloromethane (2 mL), the organic phase was separated and the aqueous phase was extracted twice with dichloromethane. The combined organic phase was concentrated to provide pure products **5b** as colorless oil (23.1 mg, 73%).

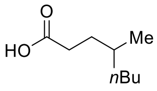

4-methyloctanoic acid (**5b**)

^{1}H NMR (400 MHz, Chloroform-*d*) δ 2.43 – 2.23 (m, 2H), 1.74 – 1.57 (m, 1H), 1.50 – 1.39 (m, 1H), 1.36 – 1.21 (m, 7H), 0.93 – 0.79 (m, 6H).

^{13}C NMR (126 MHz, Chloroform-*d*) δ 180.6, 36.4, 32.4, 32.0, 31.8, 29.3, 23.1, 19.4, 14.2.

HRMS (ESI) calculated for $C_9H_{19}O_2^+$ $[M+H]^+$: 159.1380, found: 159.1387.

Supplementary Discussions

Radical Clock Experiments

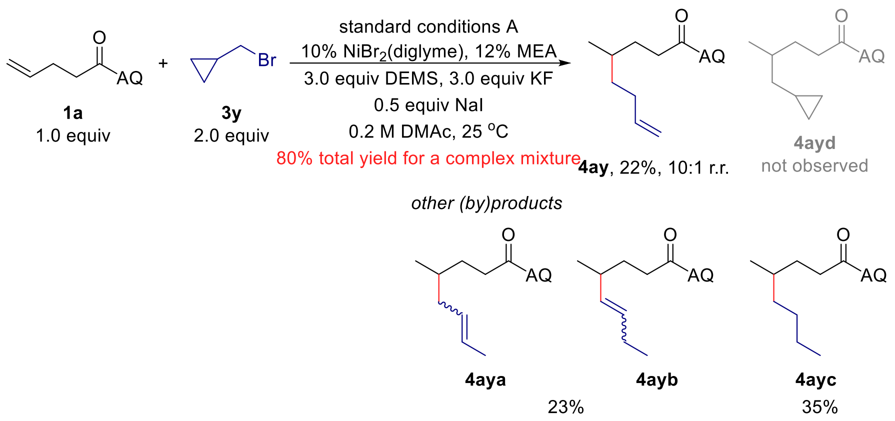

For the reaction using (bromomethyl)cyclopropane (**3y**) as an electrophile, we obtained **4ay** and other ring-opened products (**4aya**, **4ayb**, and **4ayc**) with the migration or hydrogenation of alkenyl double bond.

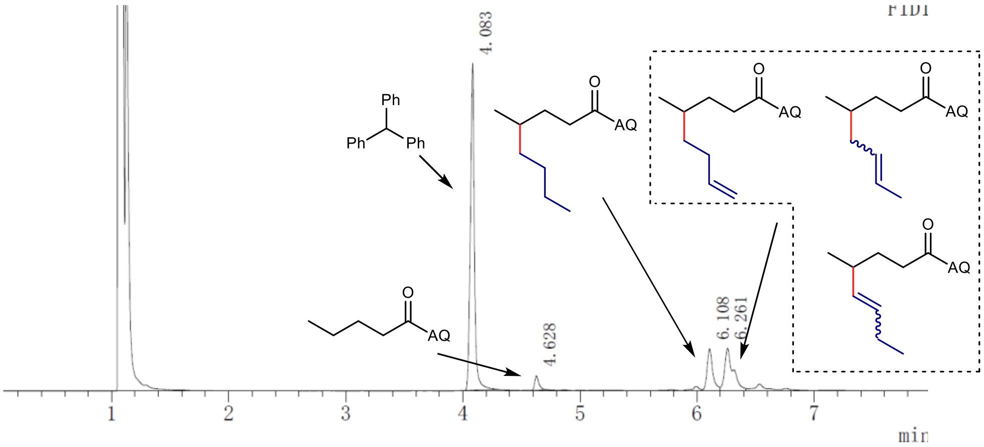

Supplementary Figure 12. GC spectra of radical clock experiment using (bromomethyl)cyclopropane

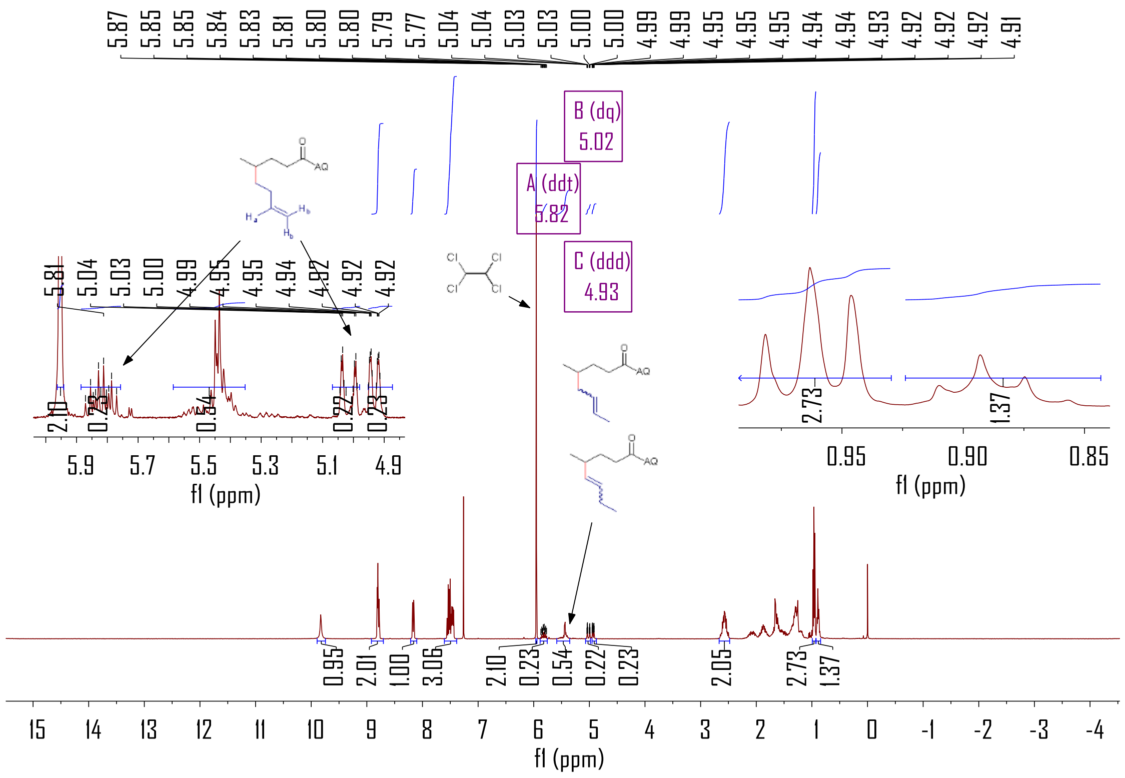

Supplementary Figure 13. ^{1}H NMR spectra of mixed products

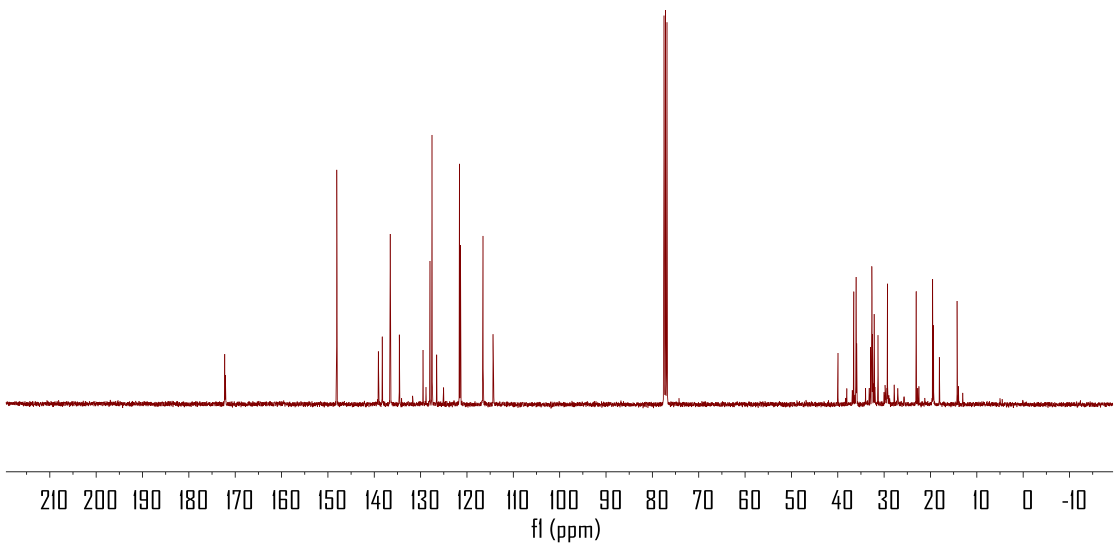

Supplementary Figure 14. ^{13}C NMR spectra of mixed products

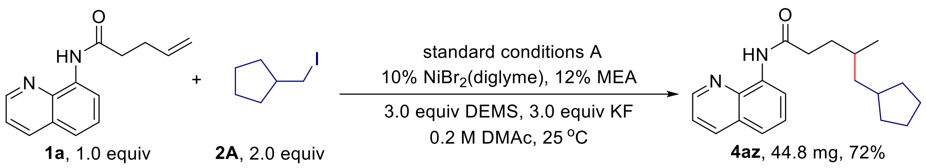

Supplementary Figure 15. Synthesis of **4az**

Following general procedure 1, *N*-(quinolin-8-yl)pent-4-enamide (**1a**, 45.2 mg, 0.2 mmol) with (iodomethyl)cyclopentane (**2A**, 84 mg, 0.4 mmol) were used. The product **4az** was isolated by column chromatography as colorless oil (44.8 mg, 72%, r.r. > 20:1).

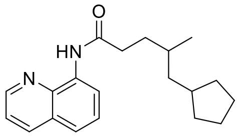

5-cyclopentyl-4-methyl-*N*-(quinolin-8-yl)pentanamide (**4az**)

Rf (petroleum ether : ethyl acetate = 5:1) = 0.45

^{1}H NMR (500 MHz, Chloroform-*d*) δ 9.82 (s, 1H), 8.99 – 8.63 (m, 2H), 8.16 (dd, *J* = 8.2, 1.7 Hz, 1H), 7.73 – 7.38 (m, 3H), 2.66 – 2.48 (m, 2H), 1.94 – 1.82 (m, 2H), 1.80 – 1.69 (m, 2H), 1.68 – 1.54 (m, 4H), 1.53 – 1.42 (m, 2H), 1.36 (ddd, *J* = 13.5, 8.2, 5.5 Hz, 1H), 1.28 – 1.15 (m, 1H), 1.12 – 0.99 (m, 2H), 0.96 (d, *J* = 6.4 Hz, 3H).

^{13}C NMR (126 MHz, Chloroform-*d*) δ 172.3, 148.1, 138.4, 136.6, 134.7, 128.1, 127.6, 121.7, 121.4, 116.6, 43.6, 37.7, 36.0, 33.4, 33.0, 32.7, 31.7, 25.2, 19.8.

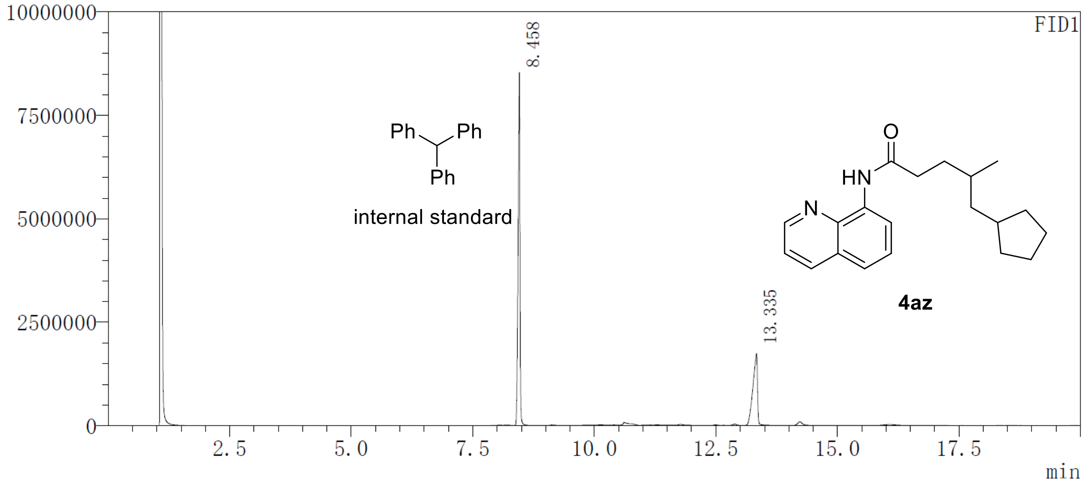

Supplementary Figure 16. GC spectra of **4az**

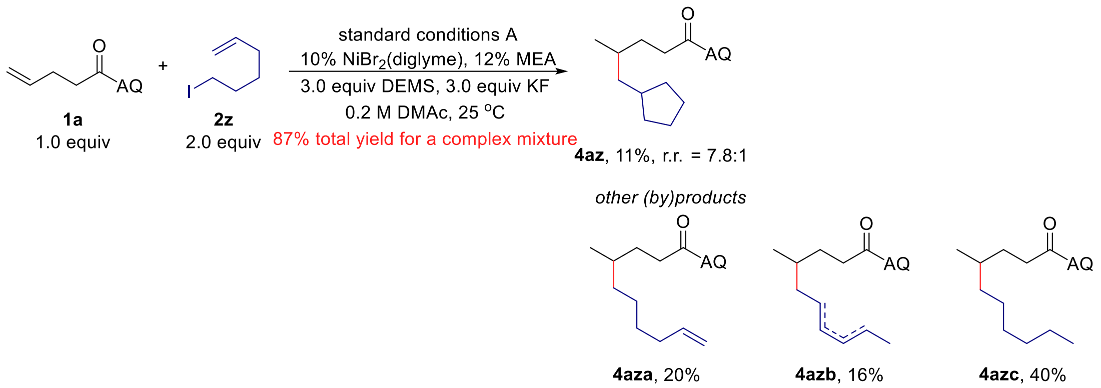

For the reaction using 6-iodohex-1-ene (**2z**) as an electrophile, we obtained ring-cyclized product **4az** and other uncyclized products (**4aza**, **4azb**, and **4azc**) with the migration or hydrogenation of alkenyl double bond.

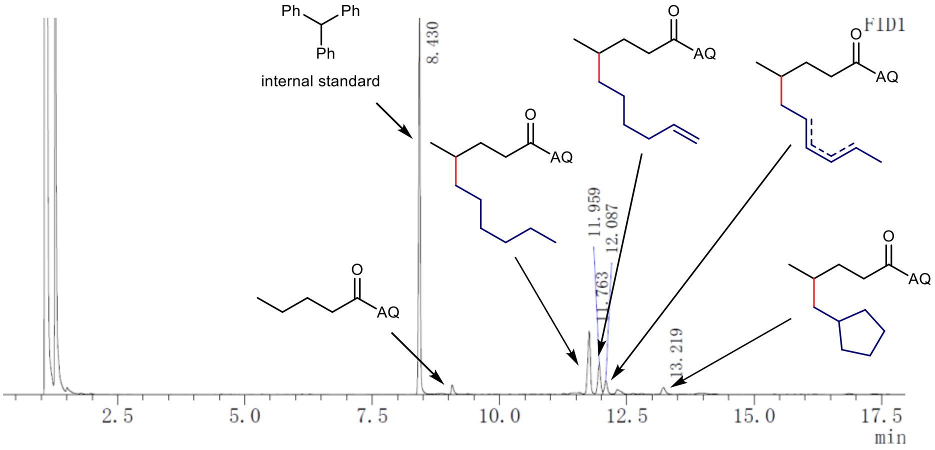

Supplementary Figure 17. GC spectra of radical clock experiment using 6-iodohex-1-ene

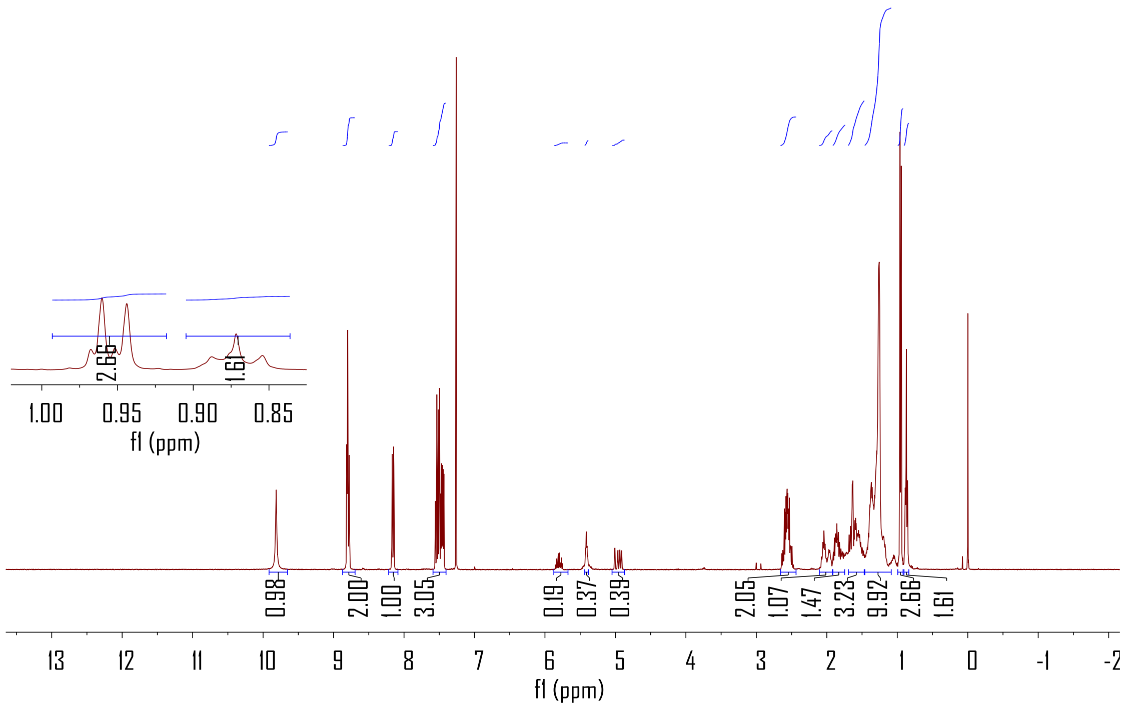

Supplementary Figure 18. ^{1}H NMR spectra of radical clock experiment using 6-iodohex-1-ene

Deuterium-labelling Experiments

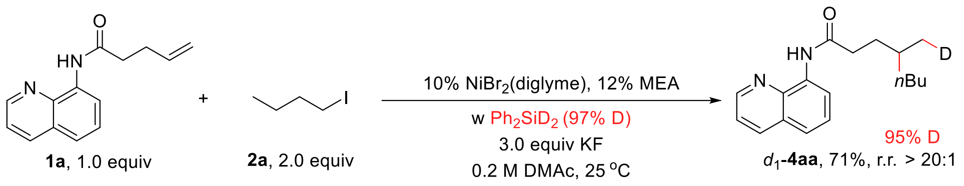

Following general procedure 1, Ph_2SiD_2 (97% deuterium content) was used. The product d_1-**4aa** was isolated by column chromatography as colorless oil (40.5 mg, 71%, r.r. > 20:1).

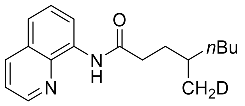

4-(methyl-*d*)-*N*-(quinolin-8-yl)octanamide (d_1-**4aa**)

Rf (petroleum ether : ethyl acetate = 5:1) = 0.50

^{1}H NMR (400 MHz, Chloroform-*d*) δ 9.83 (s, 1H), 8.92 – 8.63 (m, 2H), 8.16 (dd, *J* =

8.3, 1.7 Hz, 1H), 7.66 – 7.38 (m, 3H), 2.68 – 2.44 (m, 2H), 1.93 – 1.78 (m, 1H), 1.70 – 1.57 (m, 1H), 1.59 – 1.46 (m, 1H), 1.41 – 1.15 (m, 6H), 0.97 – 0.90 (m, 2H), 0.91 – 0.87 (m, 3H).

^{2}H NMR (61 MHz, Chloroform) δ 0.96 (s).

^{13}C NMR (126 MHz, Chloroform-*d*) δ 172.3, 148.1, 138.4, 136.7, 134.7, 128.1, 127.7, 121.7, 121.4, 116.7, 36.6, 36.1, 32.7, 32.6, 29.4, 23.1, 19.5 – 19.0 (m), 14.3.

HRMS (ESI) calculated for $C_{18}H_{24}DN_2O^+$ $[M+H]^+$: 286.2024, found: 286.2035.

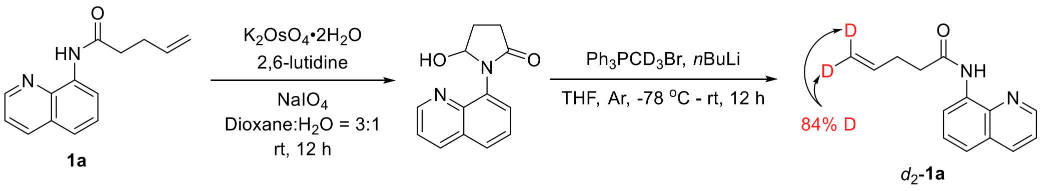

Supplementary Figure 19. Synthesis of *d*2-**1a**

According to the previously reported procedure.[1] To a solution of *N*-(quinolin-8-yl)pent-4-enamide (**1a**, 1.0 equiv) in Dioxane:H_2O = 3:1 (0.1 M) were added 2,6-lutidine (3.0 equiv), $K_2OsO_4(2H_2O)$ (4 mol%) and $NaIO_4$ (4.0 equiv). The reaction mixture was stirred at room temperature for 12 h. After the disappearance of starting material, the reaction mixture was diluted with water, extracted with CH_2Cl_2, dried with anhydrous Na_2SO_4, and concentrated in vacuo. The residue was purified by column chromatography to afford 5-hydroxy-1-(quinolin-8-yl)pyrrolidin-2-one.

To a solution of Ph_3PCD_3Br (1.2 equiv) in THF was added ^{n}BuLi (2.4 M in hexane, 2.2 equiv) at -78 °C under an argon atmosphere. The mixture was stirred at -78 °C for 30 min, 5-hydroxy-1-(quinolin-8-yl)pyrrolidin-2-one (1.0 equiv) in THF was added to the bright yellow solution dropwise. The mixture was allowed to warm to room temperature gradually and stirred for 12 h. The mixture was quenched by aqueous NH_4Cl at 0 °C and extracted with EA (3 × 50 mL). The combined organic layer was dried with Na_2SO_4. After removal of solvent, the residue was purified by flash chromatography on silica gel to give *d*2-**1a** with 84% deuterium incorporation at the terminal position.

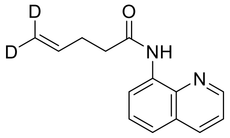

*d*2-*N*-(quinolin-8-yl)pent-4-enamide (*d*2-**1a**, 84%D)

^{1}**H NMR (400 MHz, Chloroform-*d*)** δ 9.82 (s, 1H), 8.84 – 8.55 (m, 2H), 8.14 (dt, *J* = 8.3, 1.5 Hz, 1H), 7.79 – 7.36 (m, 3H), 6.05 – 5.70 (m, 1H), 5.23 – 5.09 (m, 0.16H), 5.09 – 4.96 (m, 0.16H), 2.72 – 2.63 (m, 2H), 2.62 – 2.50 (m, 2H).
^{13}**C NMR (126 MHz, Chloroform-*d*)** δ 171.1, 148.2, 138.5, 136.9, 136.8, 136.5, 134.6, 128.1, 127.5, 121.7, 121.6, 116.6, 37.4, 29.5.

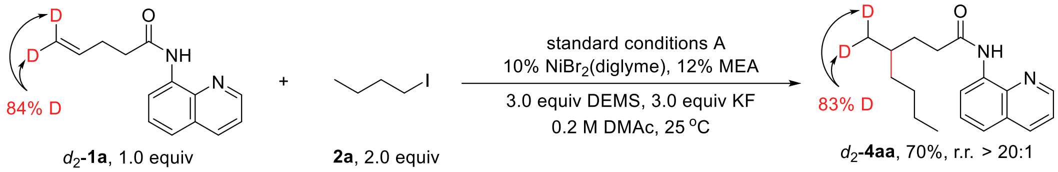

As shown above, we tested the reaction of d_2-*N*-(quinolin-8-yl)pent-4-enamide (d_2-**1a**, 45.7 mg, 0.2 mmol) with 1-iodobutane (**2a**, 73.6 mg, 0.4 mmol). The product d_2-**4aa** was obtained in 70% isolated yield (40.0 mg, r.r. > 20:1, 83% D). The *δ*-position deuterium atoms in the dideuterated substrate remained intact, and no H/D exchange was observed along the alkyl chain determined by ^{1}H NMR and ^{2}H NMR.

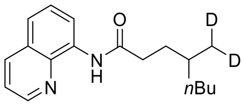

4-(methyl-d_2)-*N*-(quinolin-8-yl)octanamide (d_2-**4aa**)
Rf (petroleum ether : ethyl acetate = 5:1) = 0.50
^{1}**H NMR (400 MHz, Chloroform-*d*)** δ 9.81 (s, 1H), 8.96 – 8.61 (m, 2H), 8.15 (dd, *J* = 8.3, 1.7 Hz, 1H), 7.66 – 7.37 (m, 3H), 2.69 – 2.43 (m, 2H), 1.92 – 1.80 (m, 1H), 1.69 – 1.59 (m, 1H), 1.58 – 1.47 (m, 1H), 1.34 – 1.27 (m, 6H), 0.95 – 0.91 (m, 1.33H), 0.91 – 0.87 (m, 3H).
^{2}**H NMR (61 MHz, Chloroform)** δ 0.94 (s).
^{13}**C NMR (126 MHz, Chloroform-*d*)** δ 172.3, 148.2, 138.5, 136.6, 134.7, 128.1, 127.6, 121.7, 121.4, 116.6, 36.6, 36.1, 32.7, 32.5, 29.4, 23.1, 19.6 – 18.6 (m), 14.3.
HRMS (ESI) calculated for $C_{18}H_{23}D_2N_2O^+$ $[M+H]^+$: 287.2087, found: 287.2091.

Limitations of Substrates in Standard Conditions A

Supplementary Table 5. Proximal-selective Hydroalkylation of Unactivated Alkenes with Iodocyclohexane.

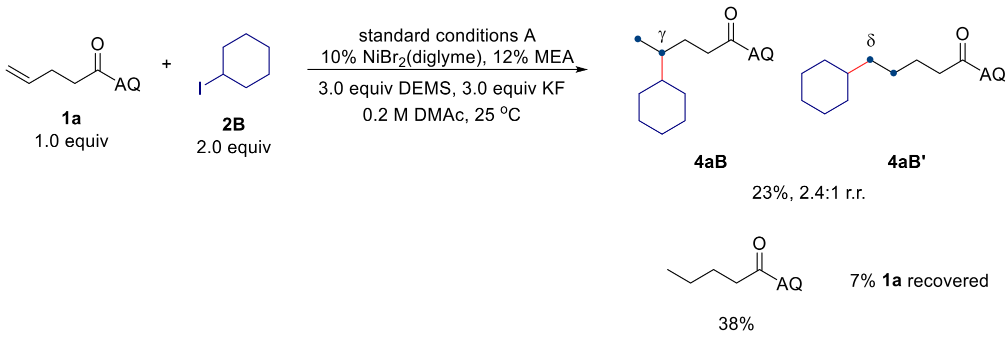

entry	variation from standard conditions A	yield/%[a]	r.r.[a]
1	none	26 (23[b])	2.4:1
2	CsF instead of KF	11	1:2.1
3	$K_3PO_4(H_2O)$ instead of KF	13	1:1.5
4	NaF instead of KF	11	3.9:1
5	$(EtO)_3SiH$ instead of DEMS	27	1.2:1
6	$(MeO)_3SiH$ instead of DEMS	40 (35[b])	1.9:1
7	80 °C	32	1.4:1

[a]Yields and regioisomeric ratios were determined by GC analysis with triphenylmethane as an internal standard. Total yield for the mixture of all regioisomers. r.r. refers to the regioisomeric ratio, that of the major product to the sum of all other isomers. In most cases, a mixture of γ- and δ-selective products was obtained; we could hardly observe other isomers. [b]Isolated yield in parentheses.

For the reaction using iodocyclohexane (**2B**) as an electrophile, the hydroalkylation product (23% yield, 2.4:1 r.r.) and the alkene protonation product (38% yield) were obtained. In addition, the starting material **1a** was recovered in a 7% yield. We examined 3 different bases and 2 different silanes to improve the coupling efficiency and regioselectivity. Using $(MeO)_3SiH$ as hydride source, **4aB** was obtained in a 35% total yield (1.9:1 r.r.)

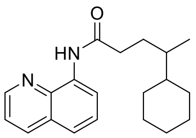

4-cyclohexyl-*N*-(quinolin-8-yl)pentanamide (**4aB**)

Following general procedure 1. The product was isolated by column chromatography, as colorless oil (21.7 mg, 35%, r.r. = 1.9:1).

^{1}H NMR (400 MHz, Chloroform-*d*) δ 9.84 (s, 1H), 9.03 – 8.54 (m, 2H), 8.33 – 8.07 (m, 1H), 7.67 – 7.39 (m, 3H), 2.70 – 2.44 (m, 2H), 2.00 – 1.87 (m, 0.67H), 1.85 – 1.57

(m, 7H), 1.48 – 1.37 (m, 1.45H), 1.33 – 1.17 (m, 4H), 1.15 – 0.97 (m, 2H), 0.91 (d, *J* = 6.8 Hz, 2H).

^{13}C NMR (126 MHz, Chloroform-*d*) δ 172.4, 172.2, 148.1, 138.2, 136.8, 134.7, 128.1, 127.7, 121.7, 121.5, 121.5, 121.5, 116.8, 42.8, 38.5, 37.9, 37.7, 37.4, 36.5, 33.5, 30.8, 30.0, 28.8, 27.0, 27.0, 26.9, 26.9, 26.7, 26.6, 26.1, 16.1.

HRMS (ESI) calculated for $C_{20}H_{26}N_2NaO^+$ $[M+Na]^+$: 333.1937, found: 333.1943.

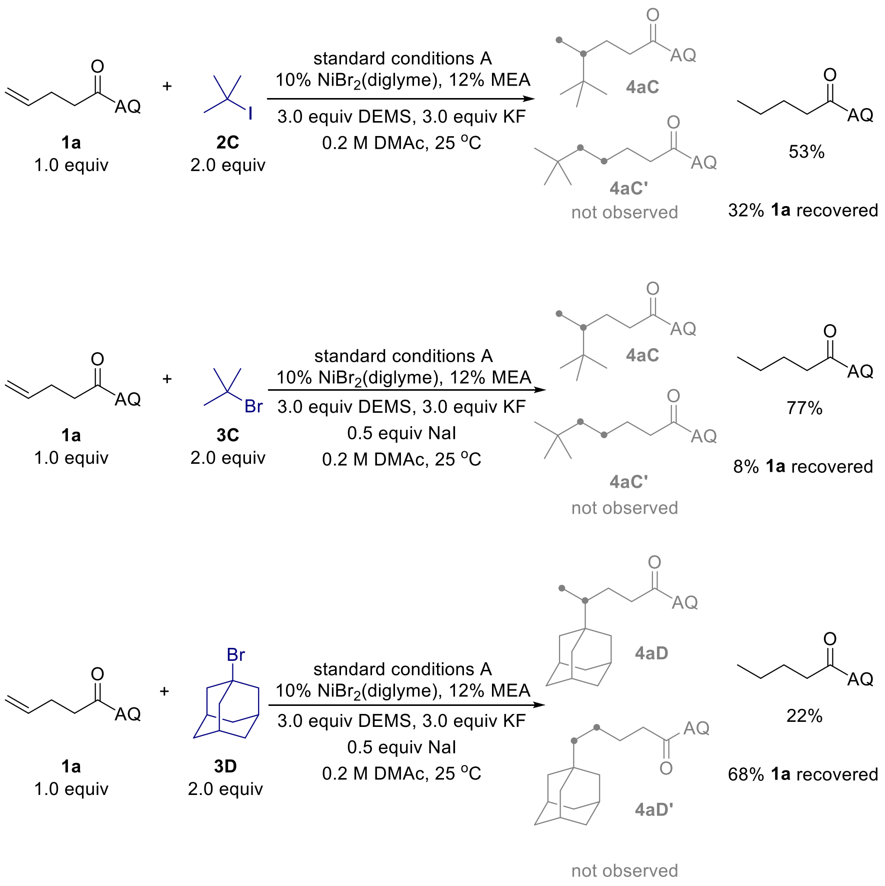

Supplementary Figure 20. Proximal-selective hydroalkylation of unactivated alkenes with tertiary electrophiles

For the reaction using tertiary electrophiles, such as 2-iodo-2-methylpropane (**2C**), 2-bromo-2-methylpropane (**3C**), and 1-bromoadamantane (**3D**), the desired hydroalkylation products were not observed. The alkene protonation product was observed as the main by-product with a large amount of starting material **1a** recovered.

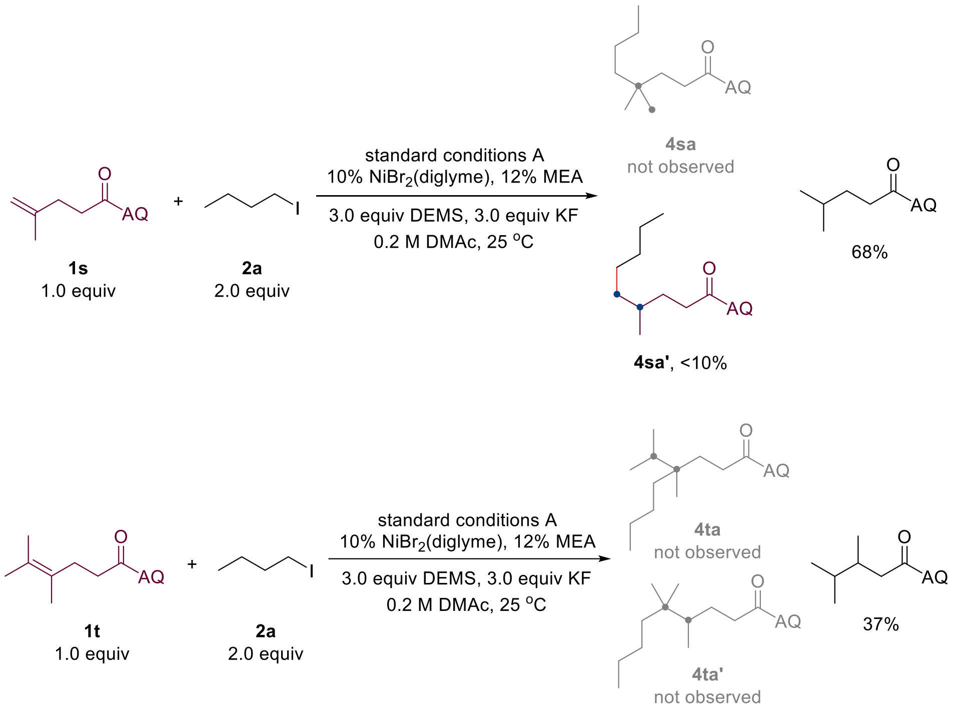

Supplementary Figure 21. Proximal-selective hydroalkylation of multi-substituted alkenes

For the reaction using 1,1-disubstituted alkene 4-methyl-*N*-(quinolin-8-yl)pent-4-enamide (**1s**), we obtained a small amount (< 10%) of linear-selective product **4sa'**. But γ-functionalized product **4sa** were not observed. In addition, the alkene protonation product was observed as the main by-product in a 68% yield.

The results for tetrasubstituted alkene 4,5-dimethyl-*N*-(quinolin-8-yl)hex-4-enamide (**1t**) were similar to 1,1-disubstituted alkene 4-methyl-*N*-(quinolin-8-yl)pent-4-enamide.

Proposed Mechanism and DFT Calculations

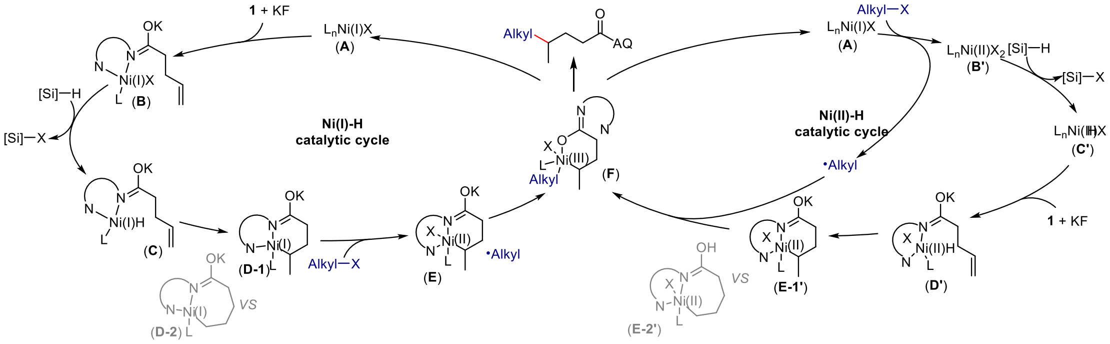

Supplementary Figure 22. Proposed mechanism of Ni(I)-H (left) and Ni(II)-H (right) catalytic cycle

DFT Calculations

All calculations were performed with Gaussian 16 package[18]. B3LYP density functional theory,[19,20] and a mixed basis set, pseudopotential of SDD[21] for Ni, and the total electron basis set of 6-31G(d) for the other atoms, were used in the unrestricted geometry optimization. The SMD solvation model[22,23] (with DMA solvent, in accordance with our experiment) was used for all of the calculations to incorporate the solvent effect. The frequency analysis was calculated at the same level of theory with geometry optimization to confirm the nature of the saddle points (local minimum with 0 imaginary frequency and transition state with 1 imaginary frequency) and to obtain the thermodynamic corrections of Gibbs free energy. For each transition state, an intrinsic reaction coordinate (IRC) analysis was conducted to verify its connection between the right reactant and product.[24,25] On the basis of the optimized structures, Truhlar's M06L functional[26] (with a mixed basis set, pseudopotential of SDD[21] for Ni and the total electron basis set of 6-311+G(d,p) for the other atoms) and the same solvation model were used for single-point energy calculations.

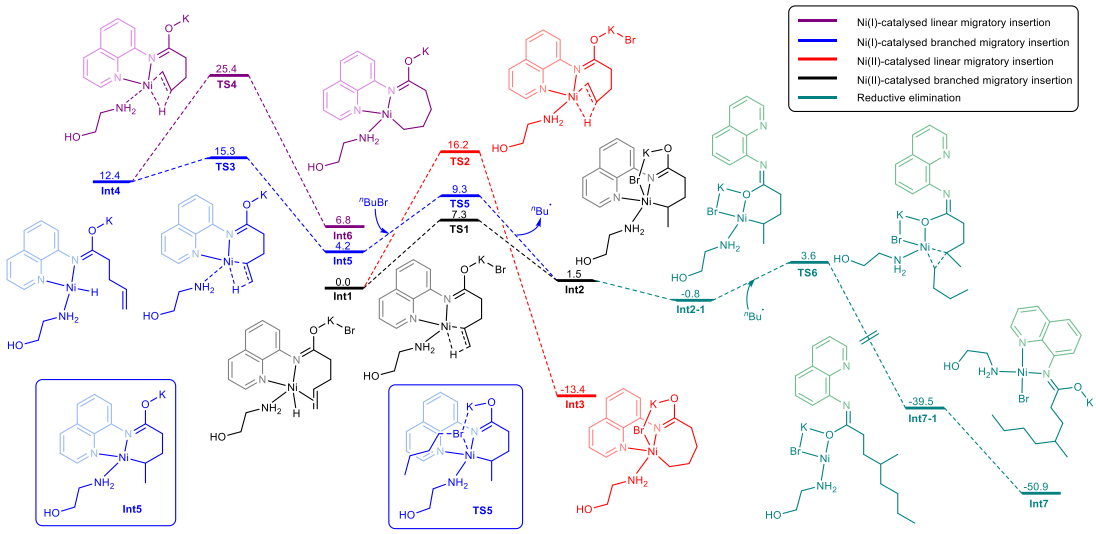

Supplementary Figure 23. DFT calculations at the M06L-D3/6-311+G(d,p)-SDD-SMD(DMA)//B3LYP-D3/6-31G(d)-SMD(DMA) level of theory. Free energies are given in kcal/mol.

In the proposed Ni(I)-catalysed branched migratory insertion, nickel(I)-alkyl species **Int5** abstracts halogen from an alkyl halide to form nickel(II)-alkyl species **Int2** and an alkyl radical, passing over the transition state **TS5** with an energy barrier of 5.1 kcal/mol.

In the reductive elimination step, the alkyl radical is trapped by nickel(II)-alkyl species **Int2-1** to give Ni(III) species **TS6** and undergo rapid reductive elimination to complete

the catalytic cycle.

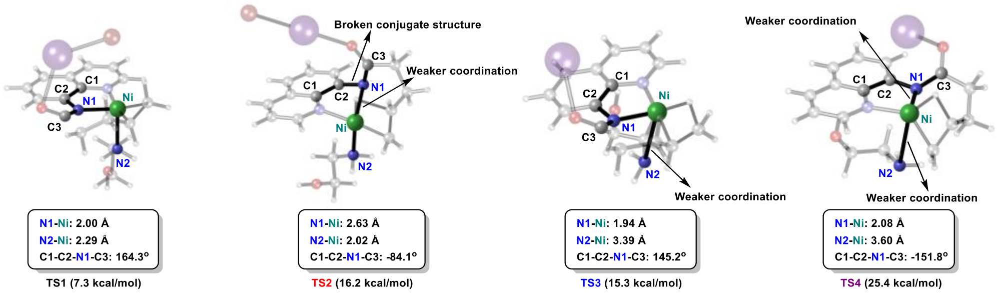

Supplementary Figure 24. Optimized 3D structures of **TS1, TS2, TS3** and **TS4.**

3D structures of four transition states, **TS1**, **TS2**, **TS3** and **TS4**, are provided to explain the reaction regioselectivity clearly. The bifacial angle of C1-C2-N1-C3 in **TS2** appears to be -84.1°. Compared to **TS1** **(**the bifacial angle of C1-C2-N1-C3 is 164.3°**)**, the twisted seven-membered ring in **TS2** breaks the conjugate structure of the alkene ligand. The bifacial angle in **TS2** almost changes from plane to horizontal, which is a state with significant steric hindrance. N1-Ni in **TS2** has a much longer bond length of 2.63 Å compared to 2.00 Å in **TS1**, indicating that this distorted structure weakens the N1-Ni coordination. Therefore, **TS2** has higher relative energy than **TS1**.

N2-Ni bonds in **TS3** and **TS4** are much longer than the sum of van der Waals radii of nickel and nitrogen (3.39 Å>3.25 Å, 3.60 Å>3.25 Å), suggesting weak coordinations close to being ignored. Moreover, a similar twisted structure of **TS4** also causes the destabilization of the N1-Ni bond (2.08 Å in **TS4** vs 1.94 Å in **TS3**), making it less stable than **TS3**.

Supplementary Figures

NMR spectra of substrates

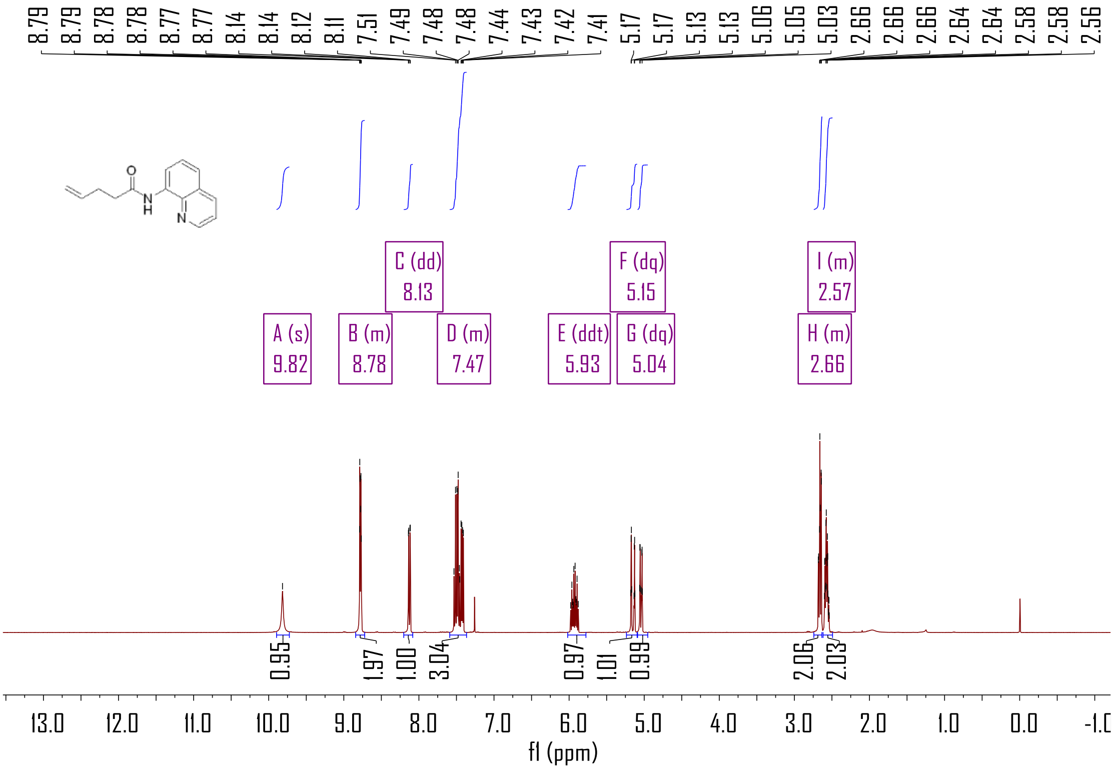

Supplementary Figure 25. ^{1}H NMR spectra of **1a**

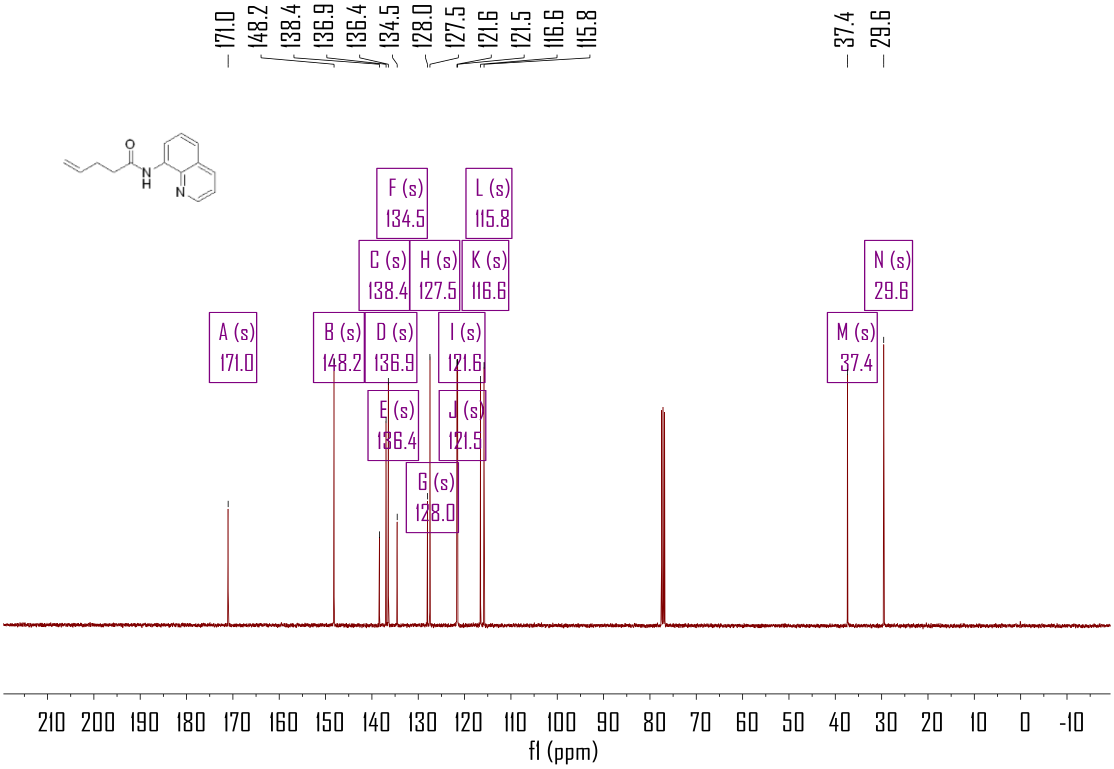

Supplementary Figure 26. ^{13}C NMR spectra of **1a**

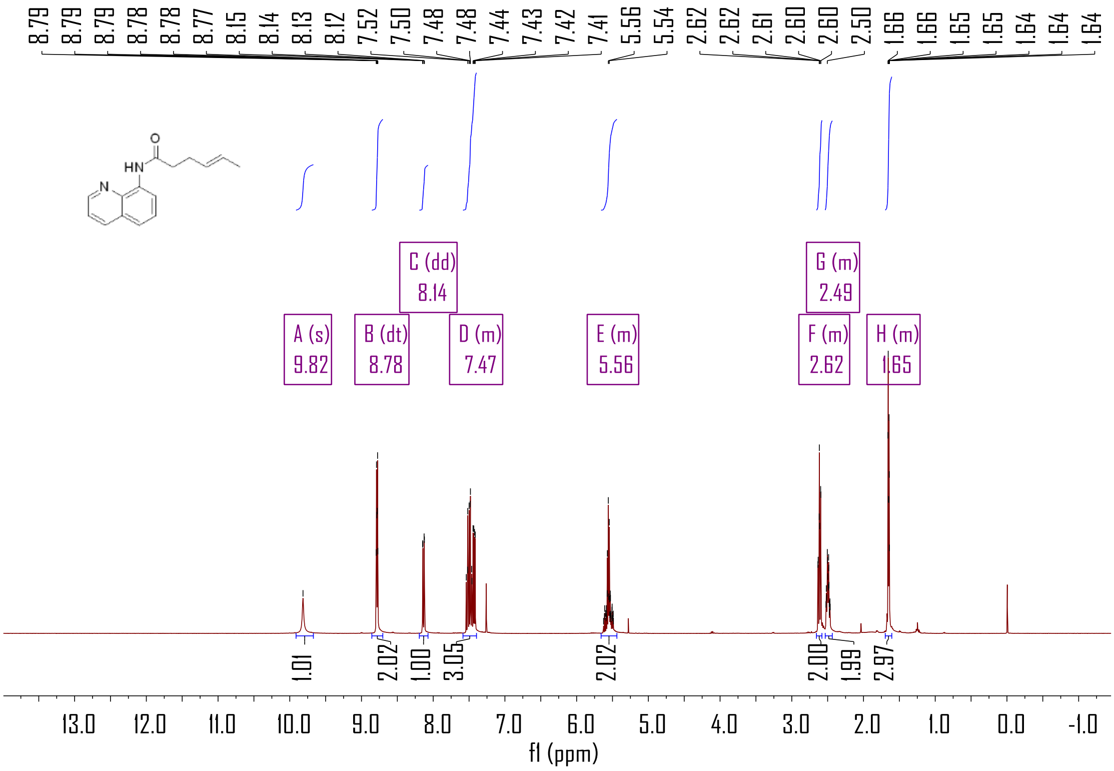

Supplementary Figure 27. ^{1}H NMR spectra of *E*-**1b**

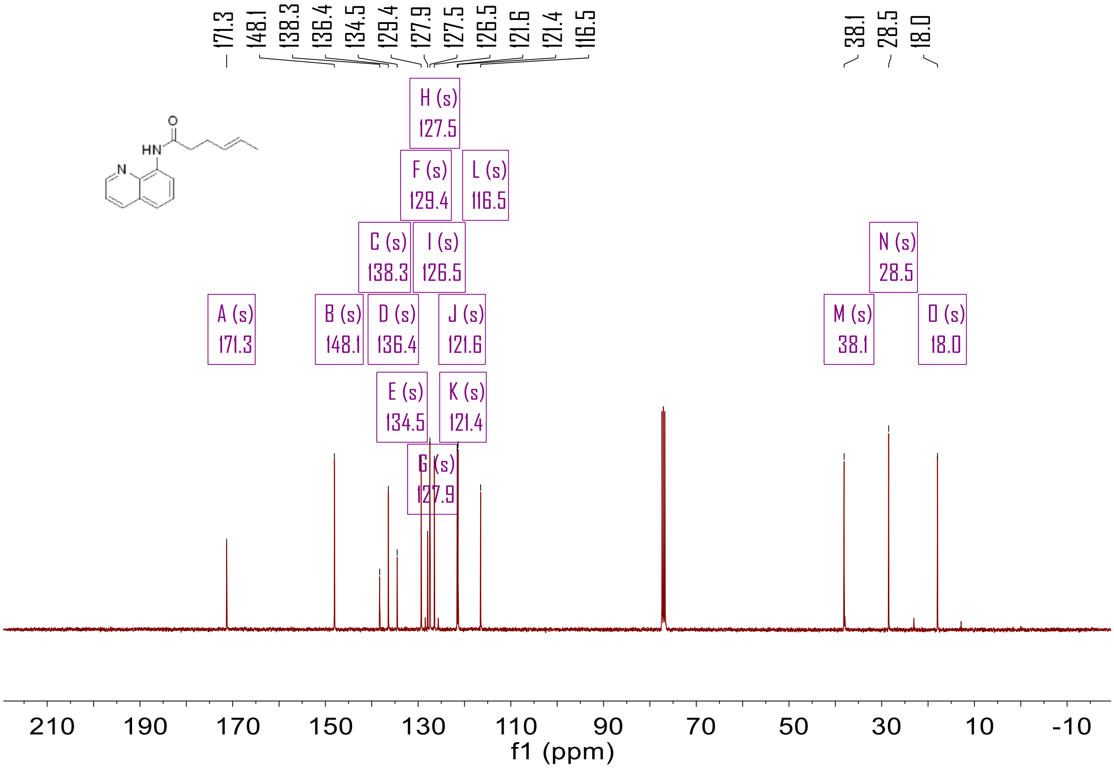

Supplementary Figure 28. ^{13}C NMR spectra of *E*-**1b**

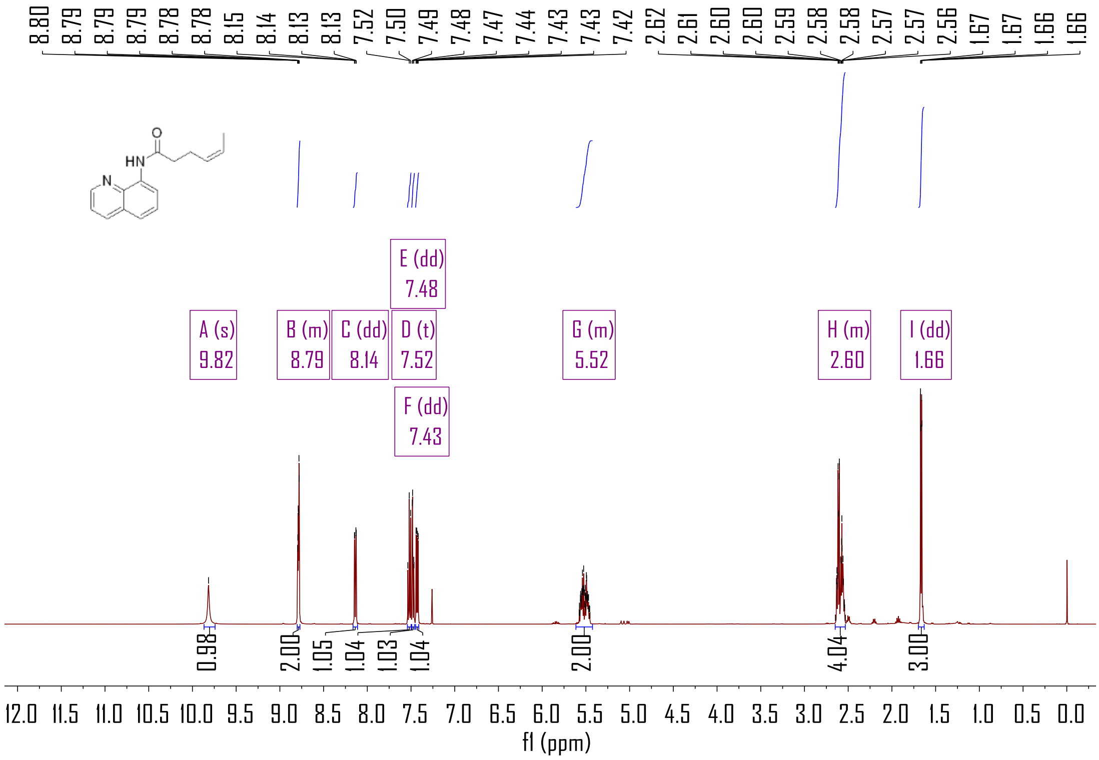

Supplementary Figure 29. ^{1}H NMR spectra of *Z*-**1b**

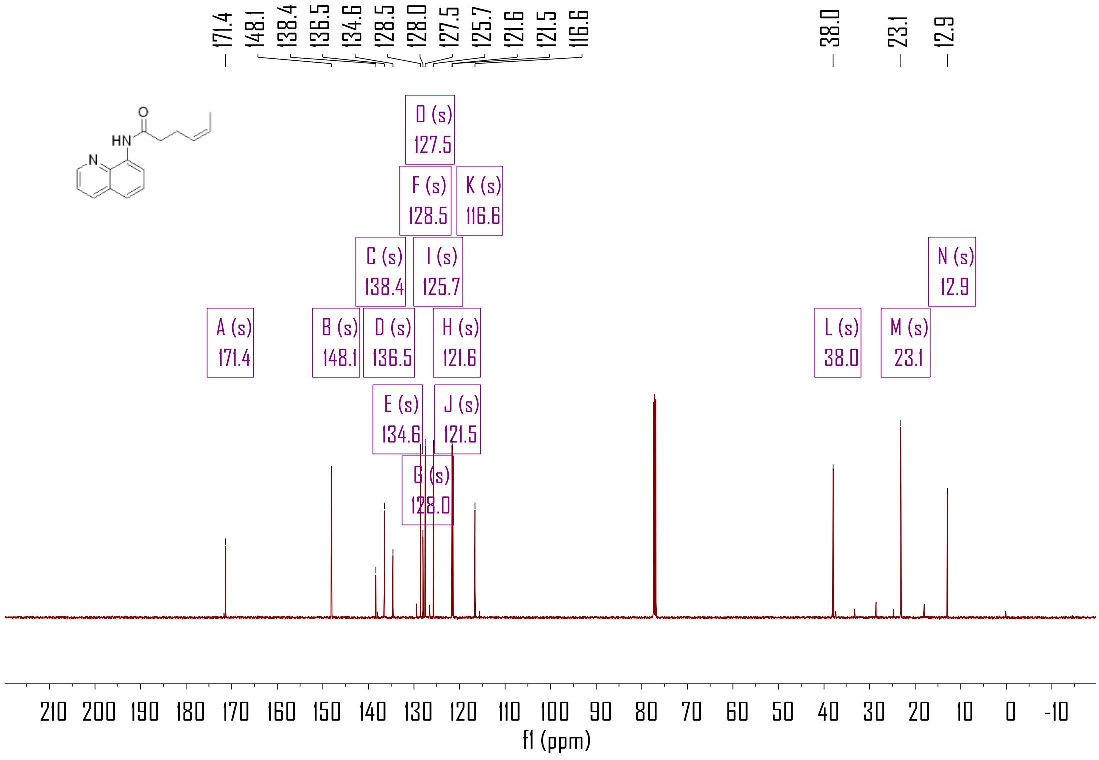

Supplementary Figure 30. ^{13}C NMR spectra of *Z*-**1b**

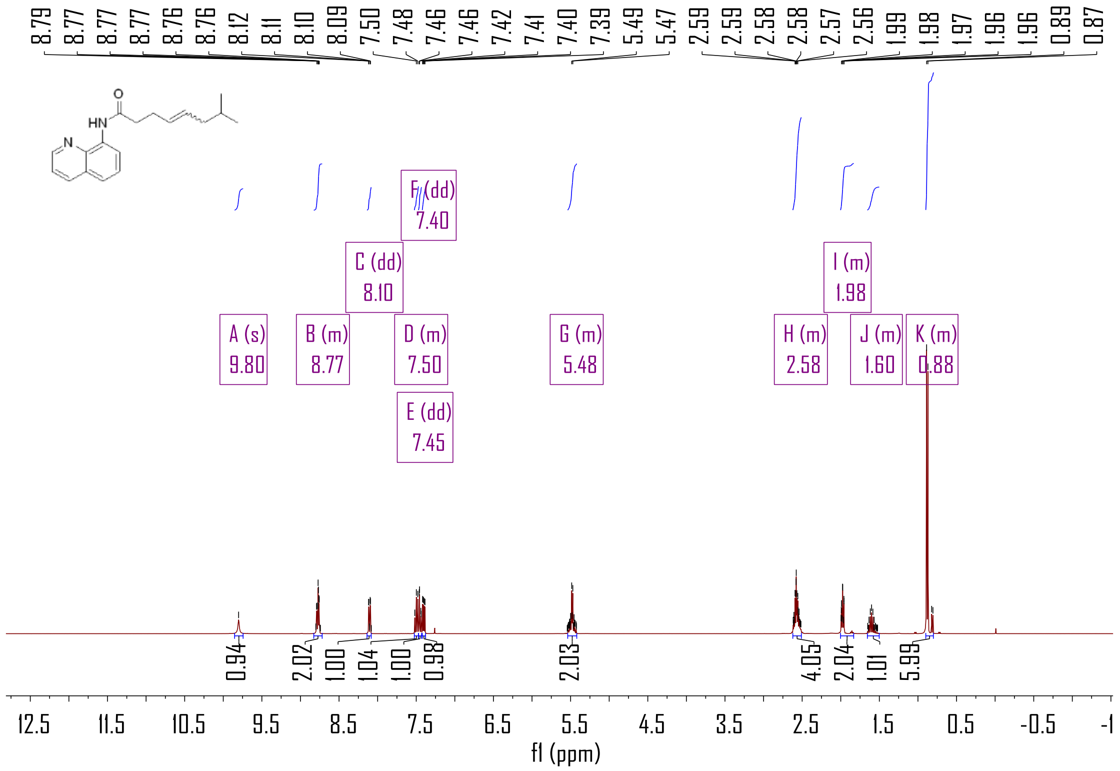

Supplementary Figure 31. ^{1}H NMR spectra of **1c**

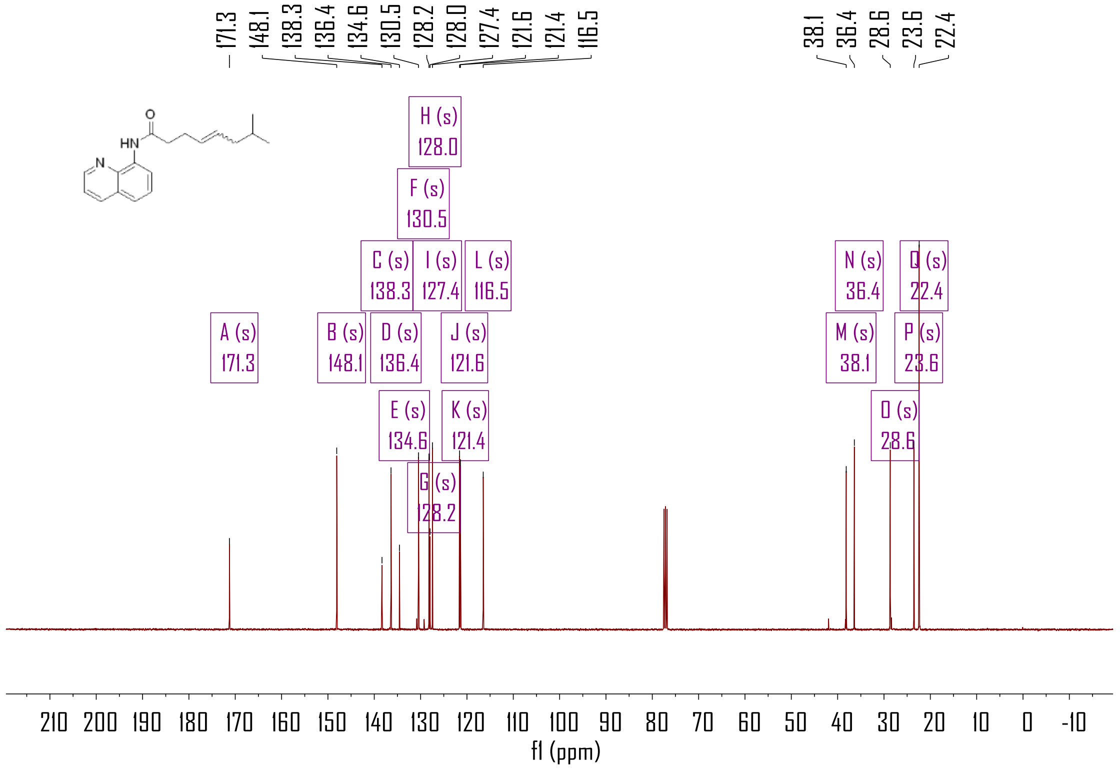

Supplementary Figure 32. ^{13}C NMR spectra of **1c**

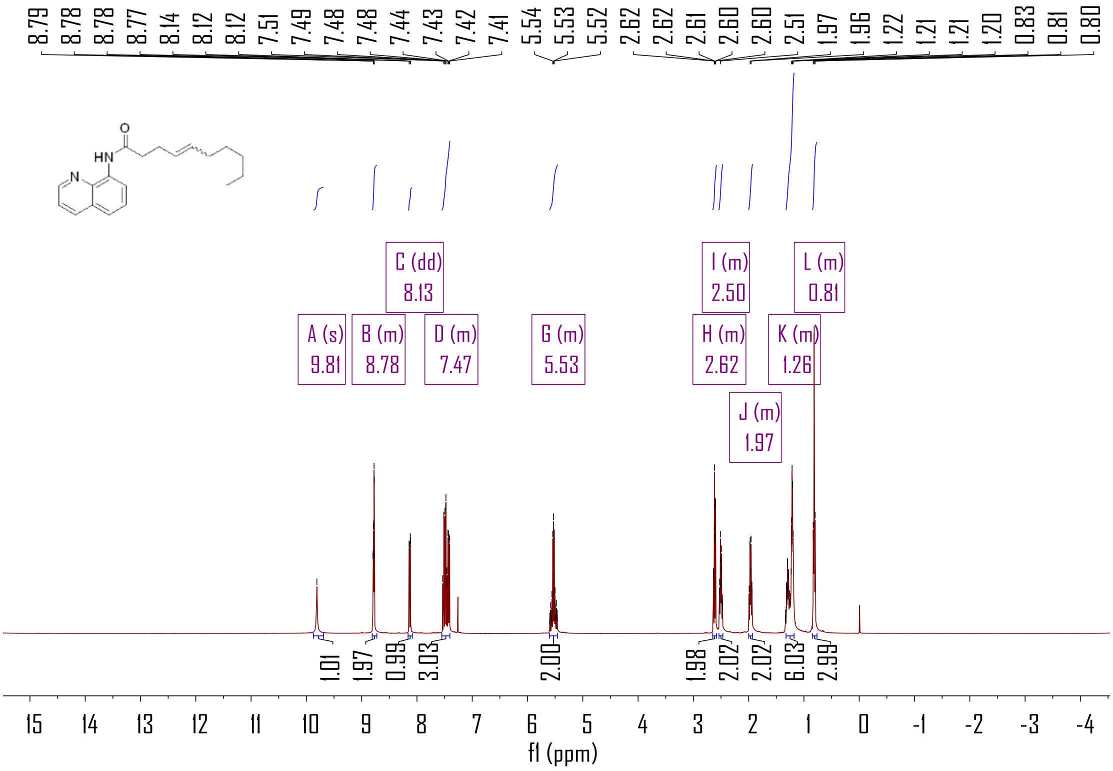

Supplementary Figure 33. ^{1}H NMR spectra of **1d**

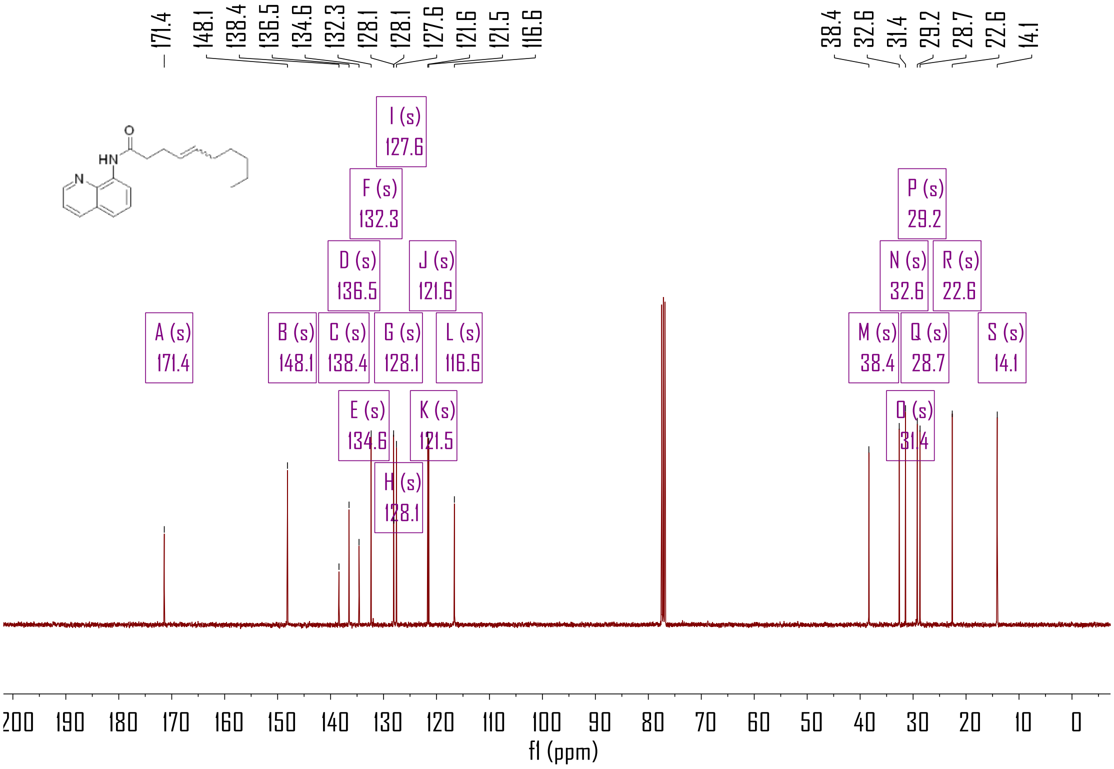

Supplementary Figure 34. ^{13}C NMR spectra of **1d**

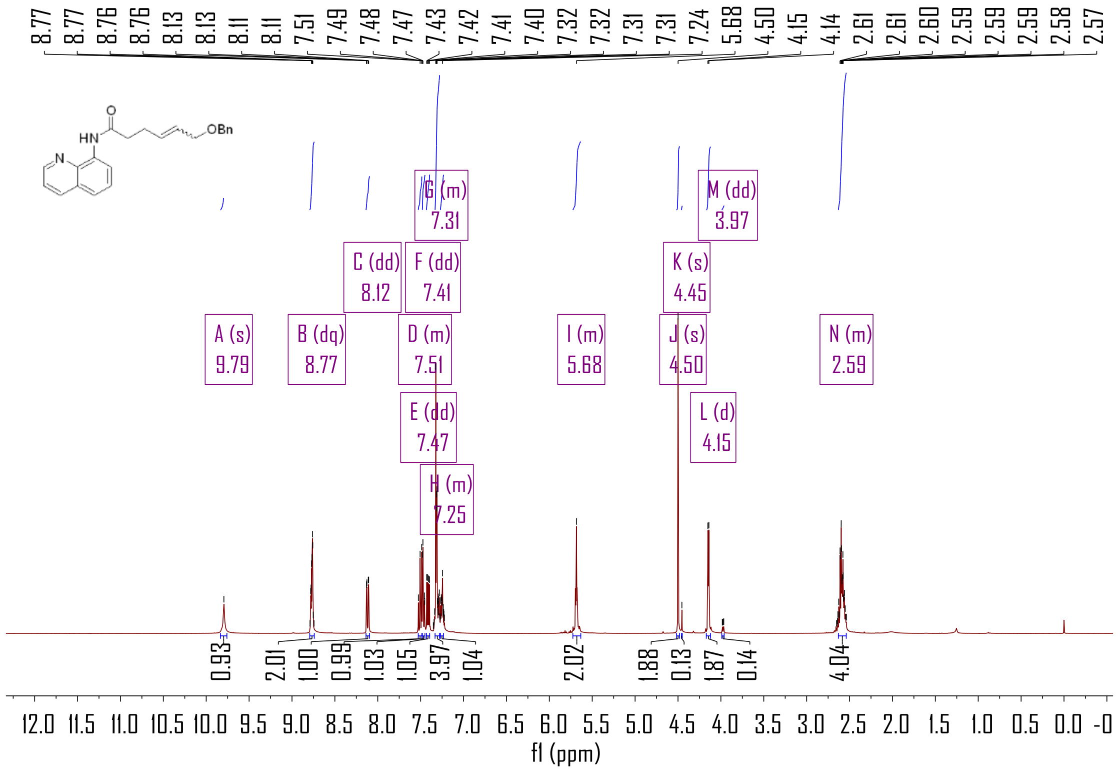

Supplementary Figure 35. ^{1}H NMR spectra of **1e**

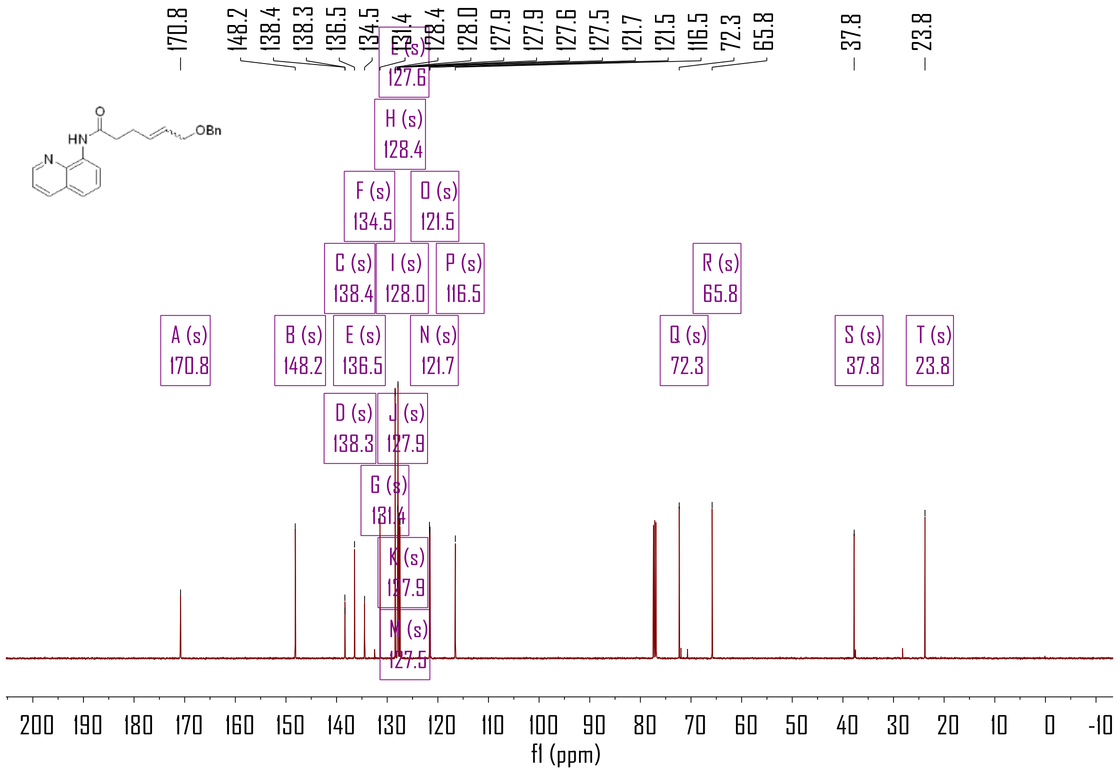

Supplementary Figure 36. ^{13}C NMR spectra of **1e**

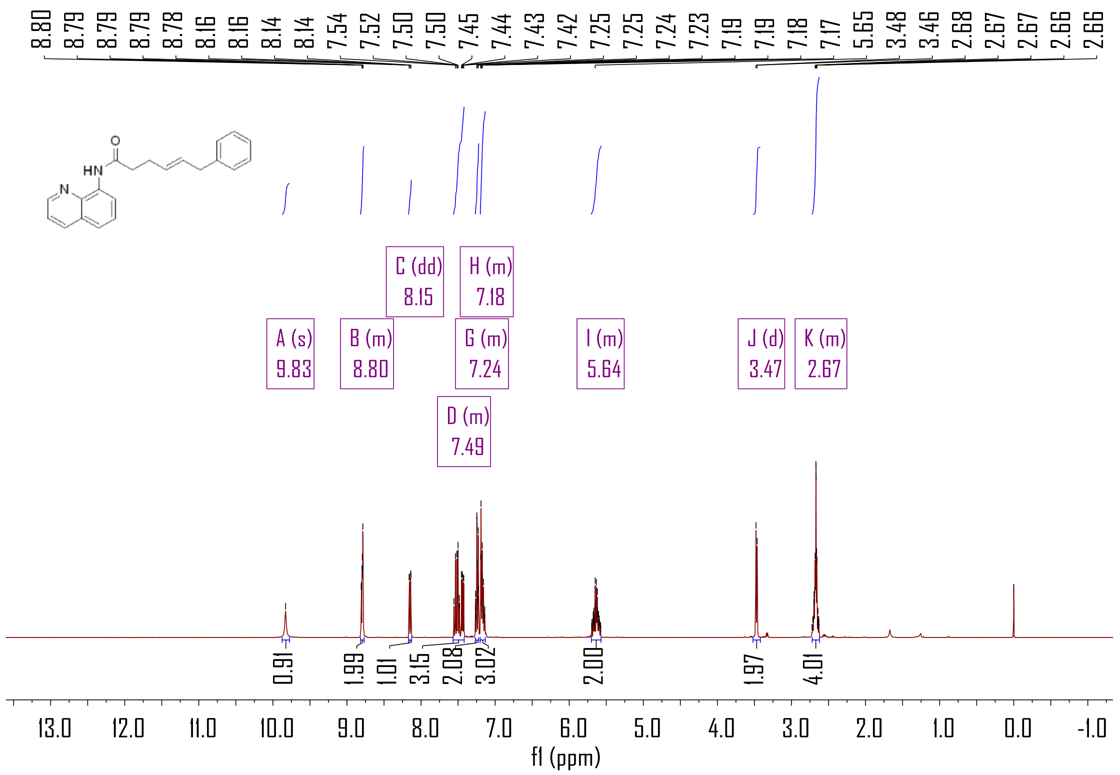

Supplementary Figure 37. ^{1}H NMR spectra of **1f**

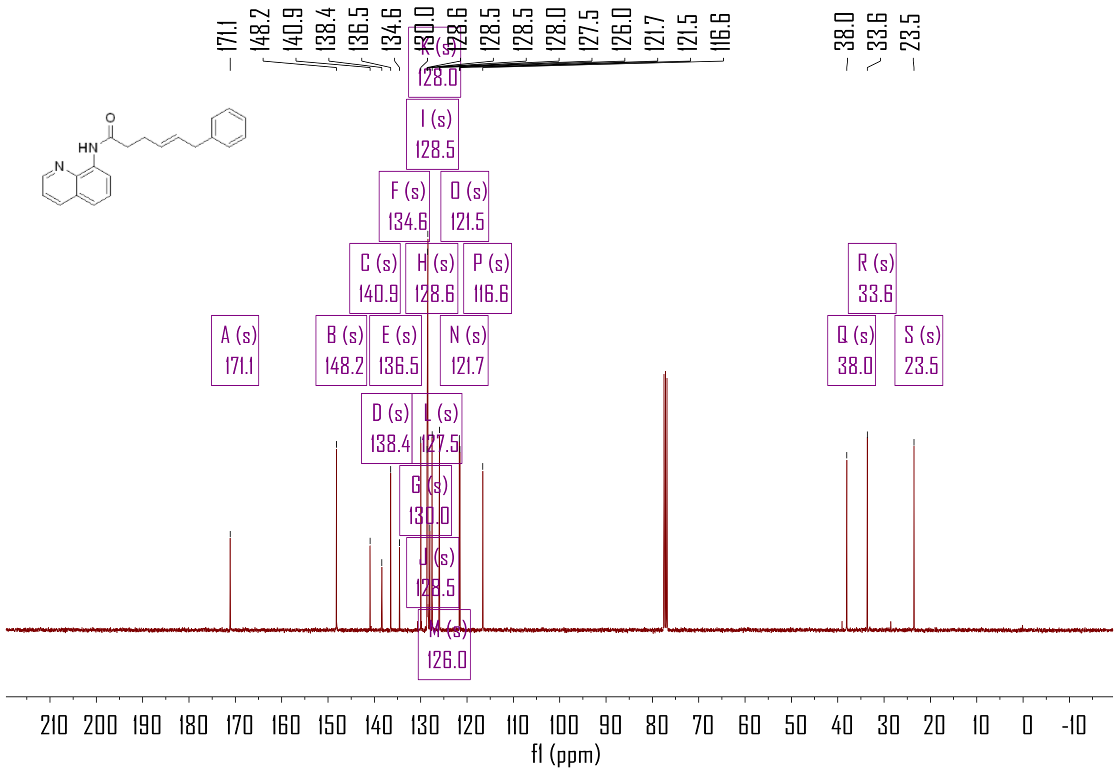

Supplementary Figure 38. ^{13}C NMR spectra of **1f**

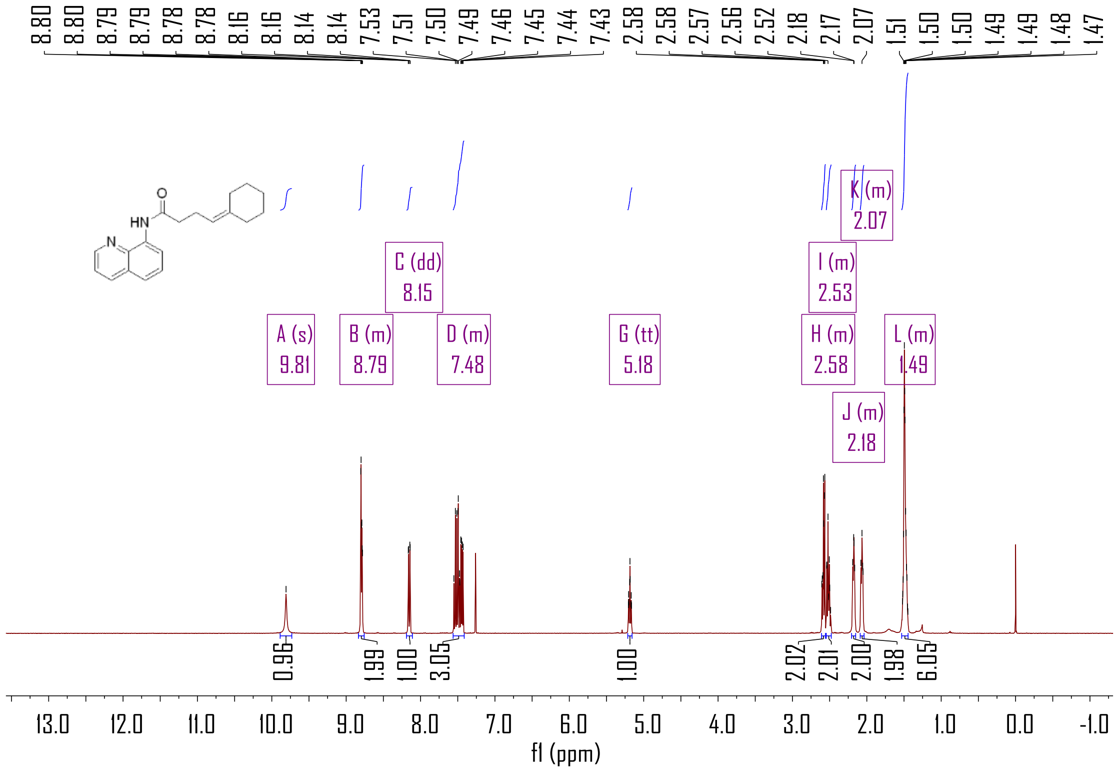

Supplementary Figure 39. ^{1}H NMR spectra of **1g**

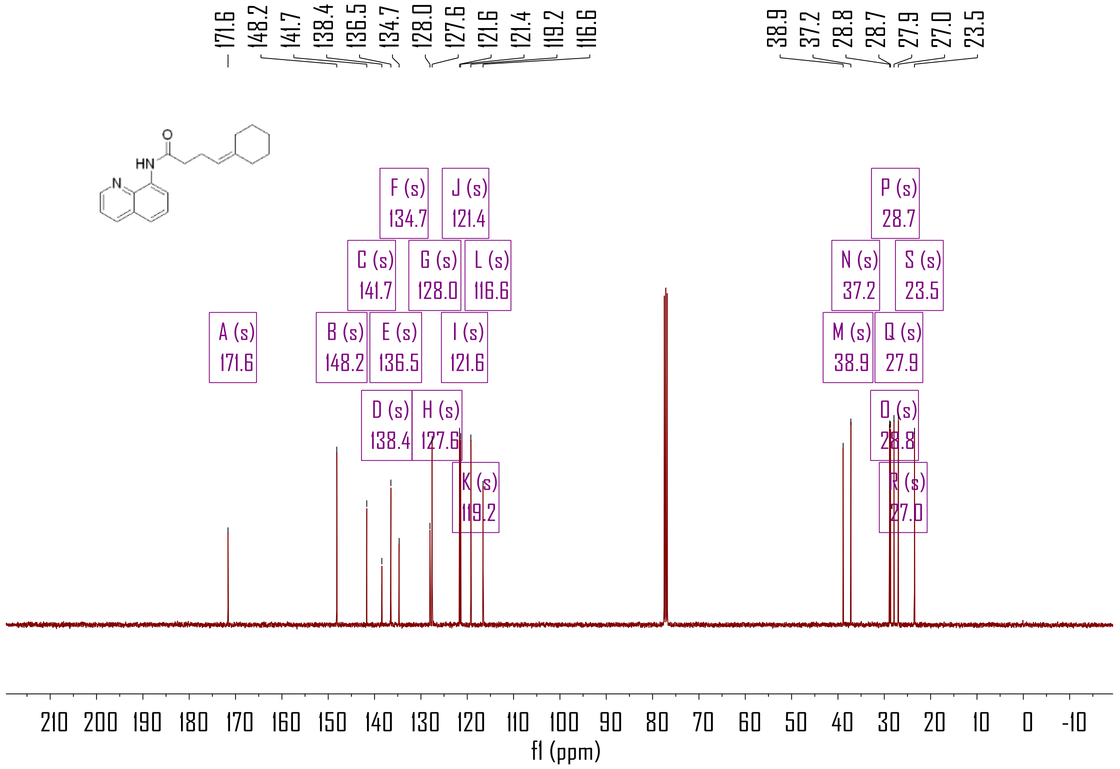

Supplementary Figure 40. ^{13}C NMR spectra of **1g**

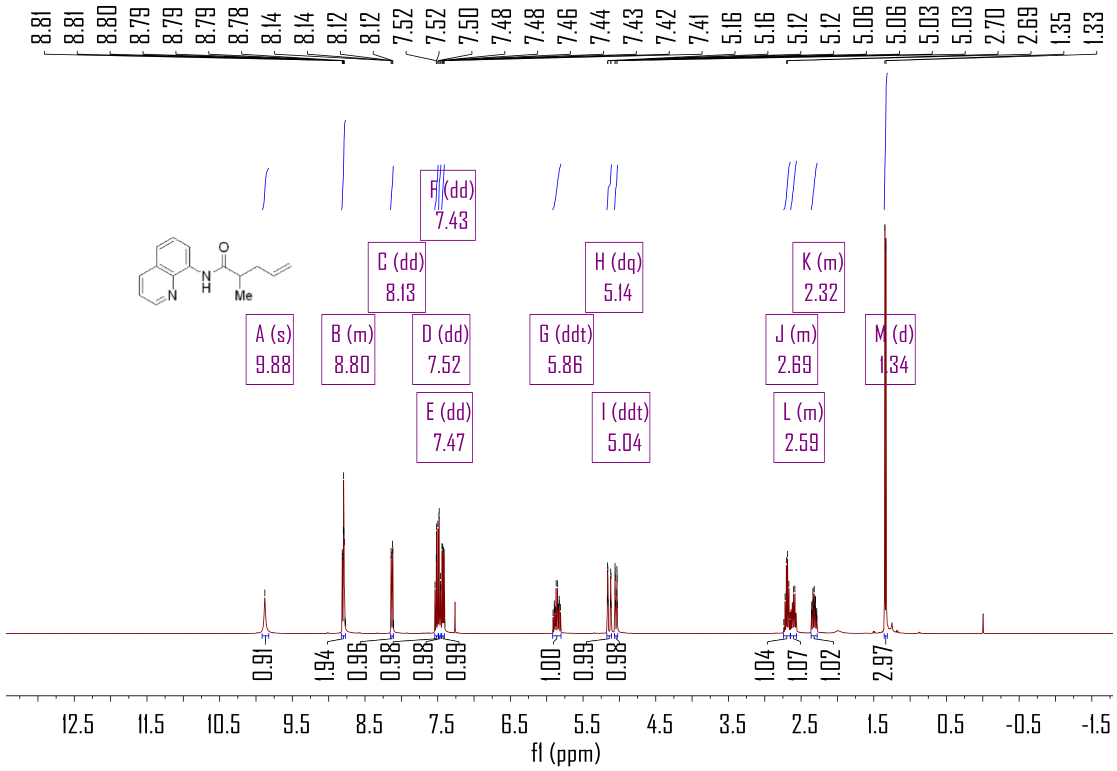

Supplementary Figure 41. ^{1}H NMR spectra of **1h**

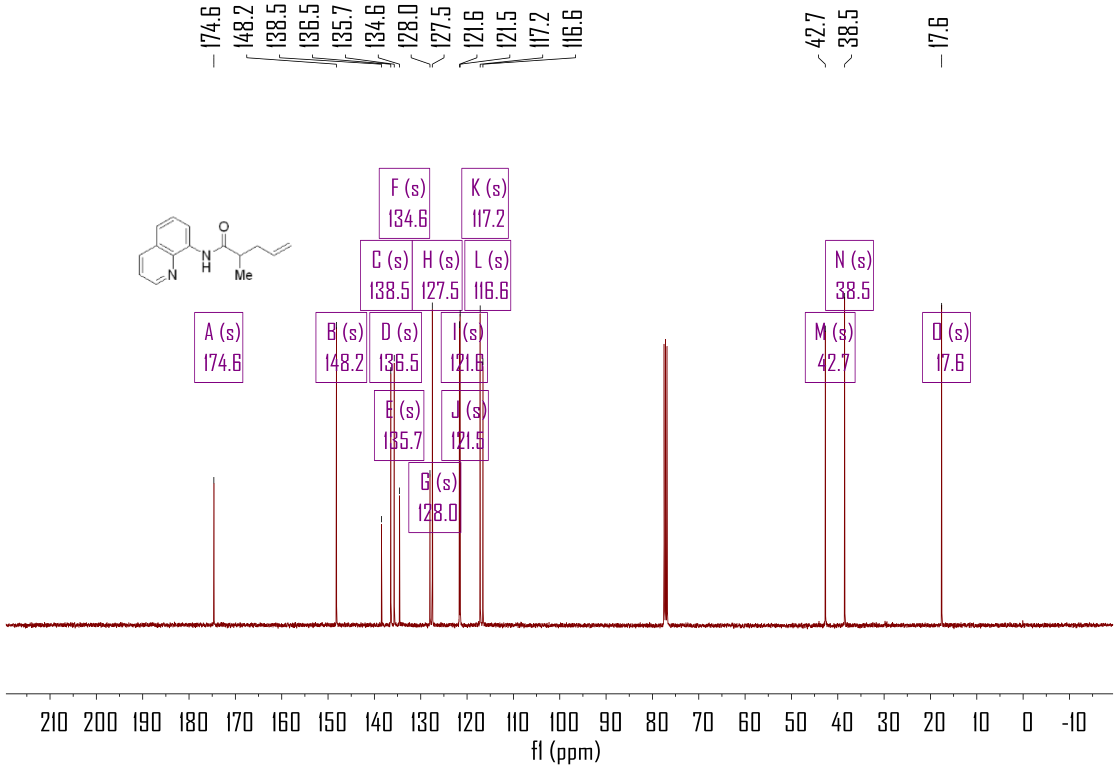

Supplementary Figure 42. ^{13}C NMR spectra of **1h**

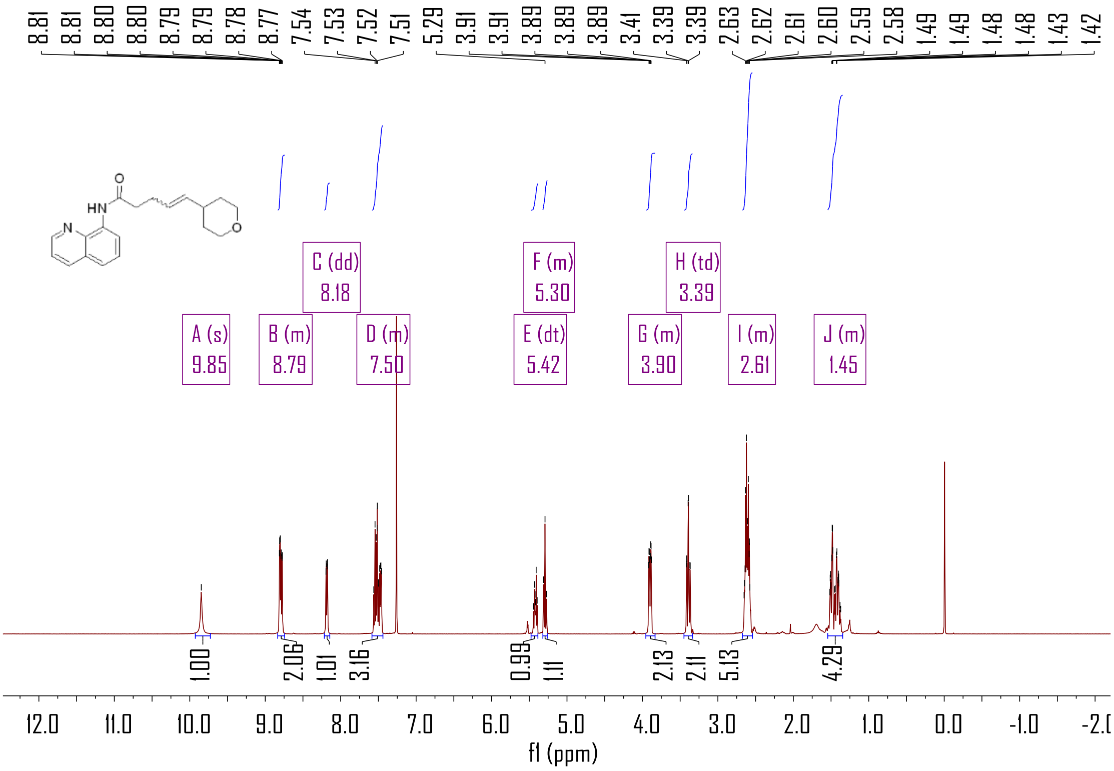

Supplementary Figure 43. ^{1}H NMR spectra of **1i**

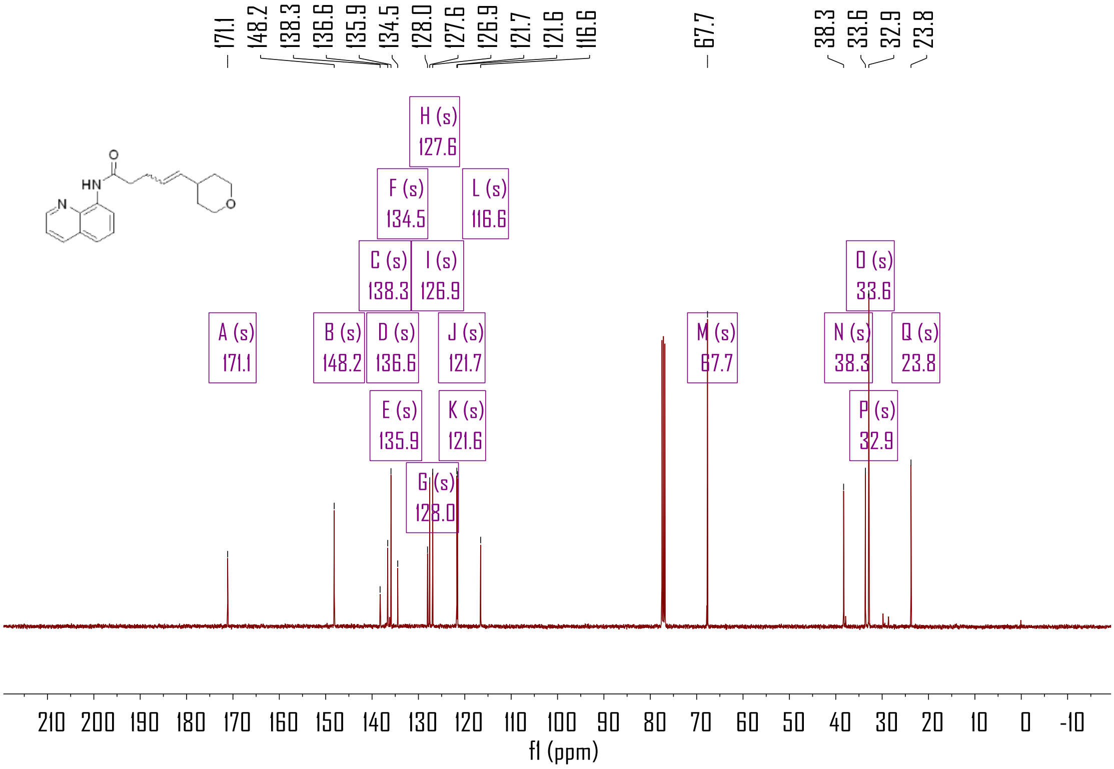

Supplementary Figure 44. ^{13}C NMR spectra of **1i**

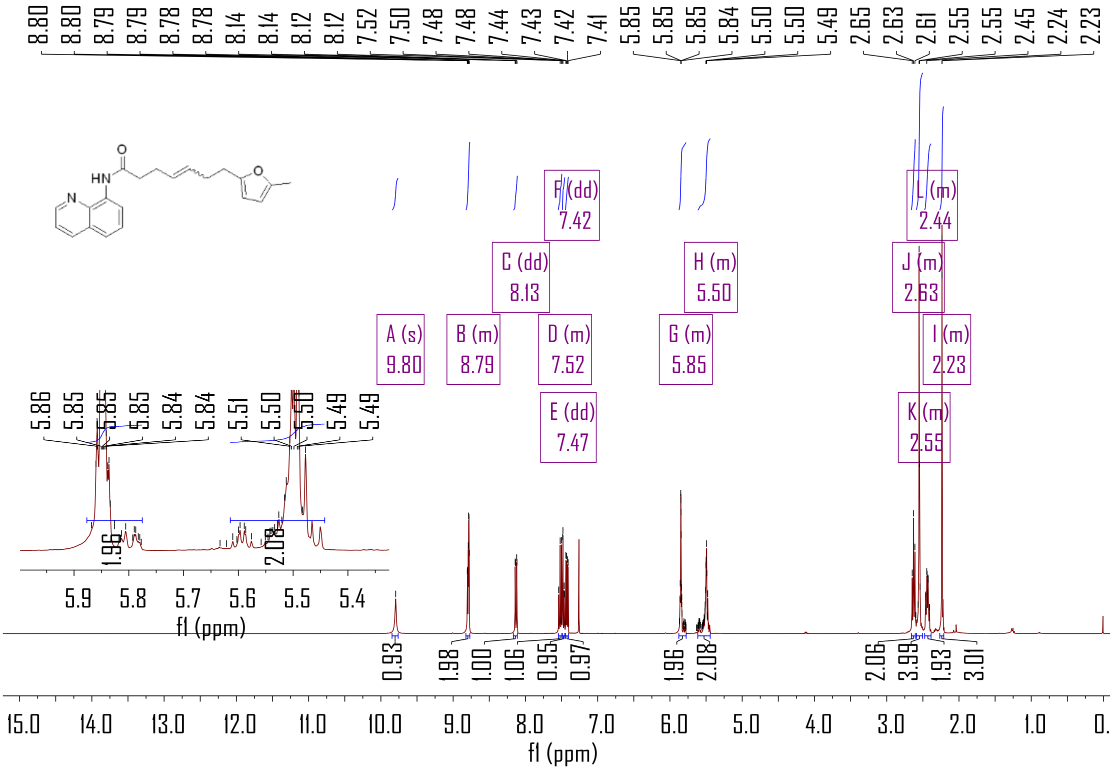

Supplementary Figure 45. ^{1}H NMR spectra of **1j**

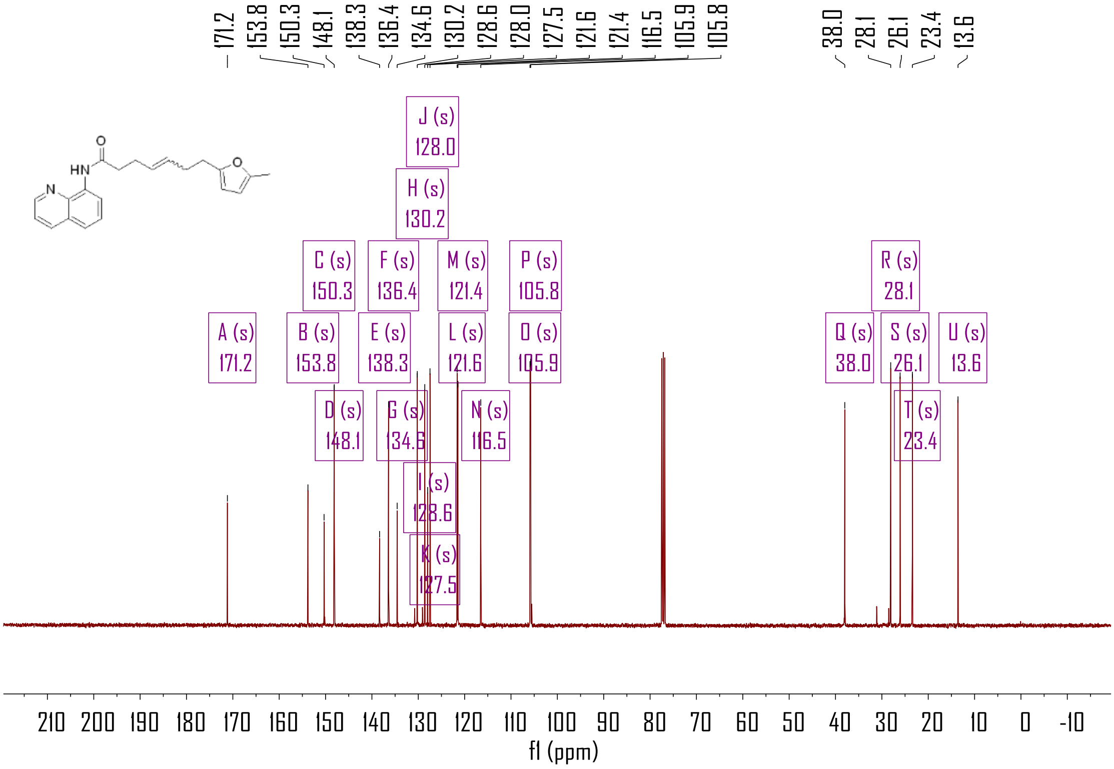

Supplementary Figure 46. ^{13}C NMR spectra of **1j**

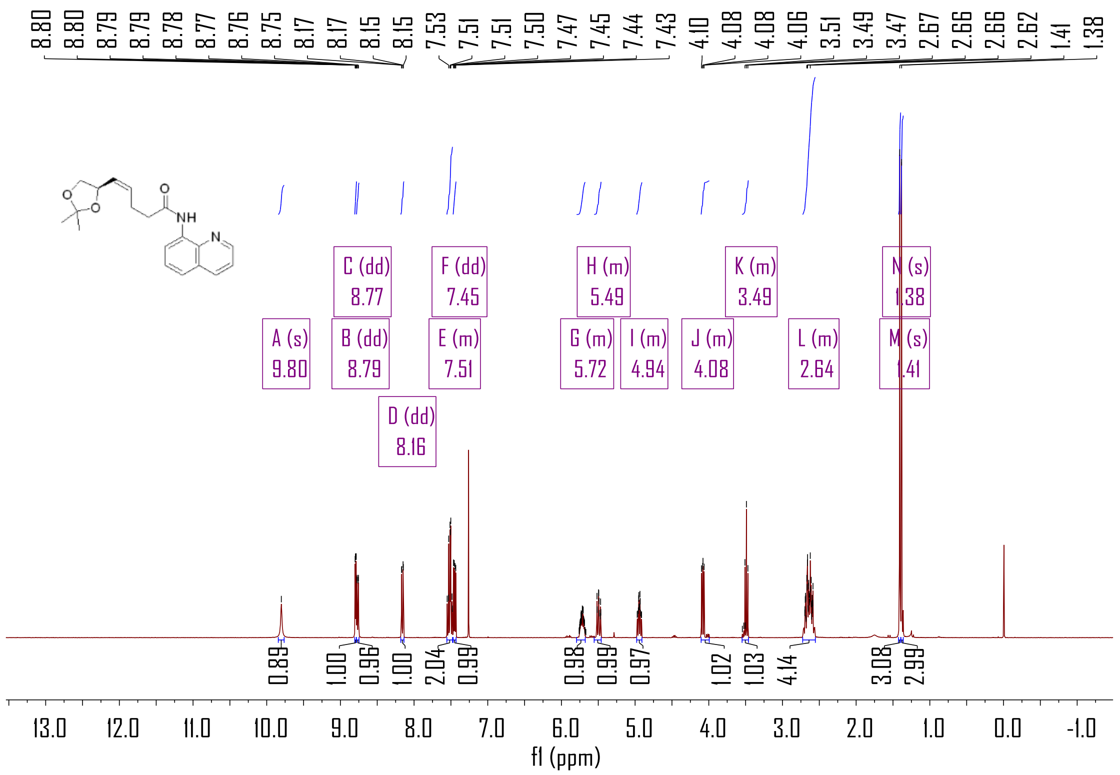

Supplementary Figure 47. ^{1}H NMR spectra of **1k**

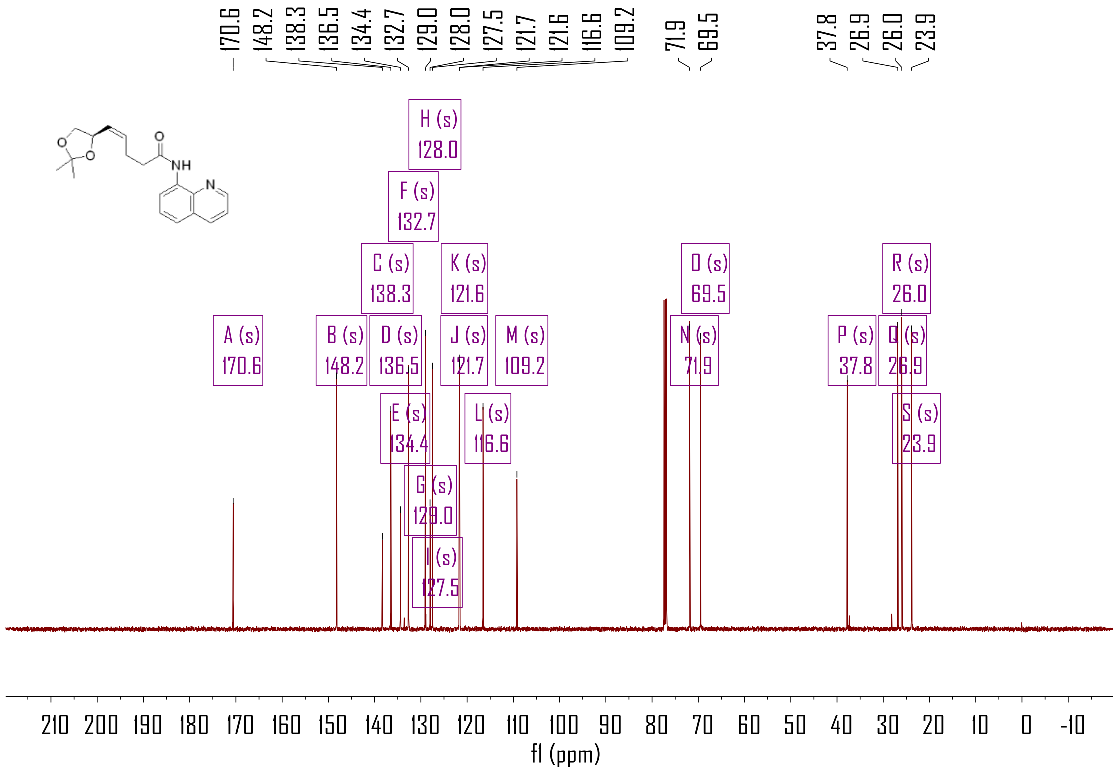

Supplementary Figure 48. ^{13}C NMR spectra of **1k**

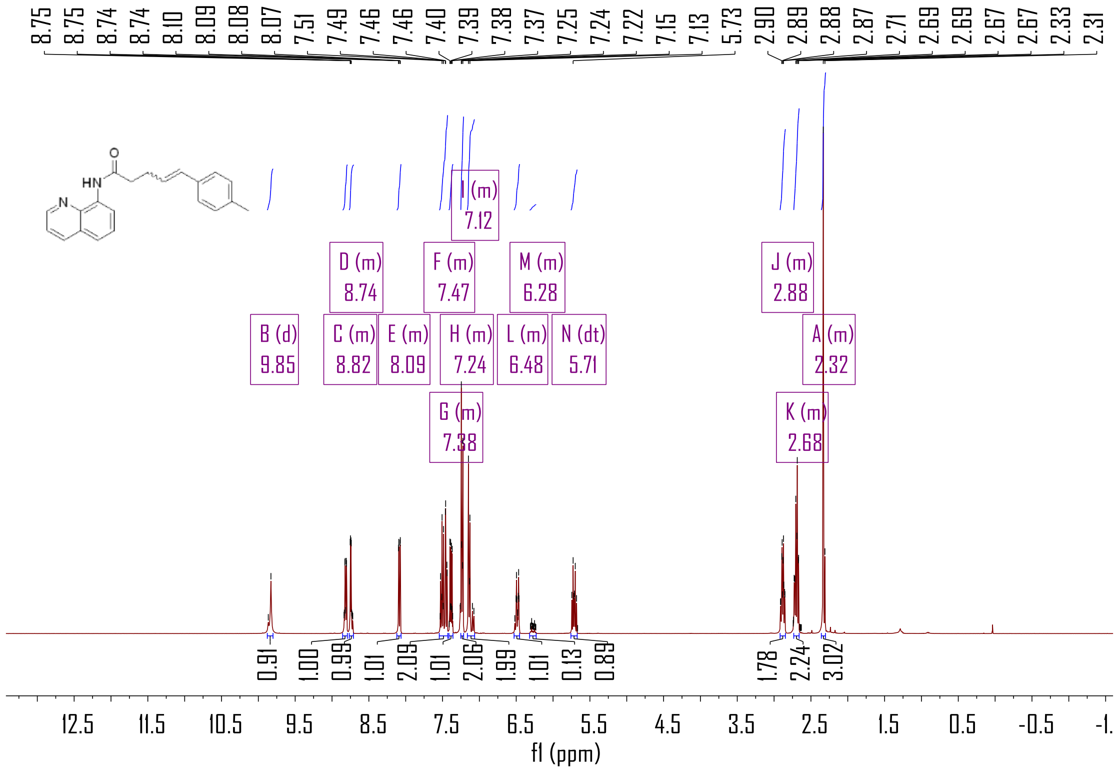

Supplementary Figure 49. ^{1}H NMR spectra of **1l**

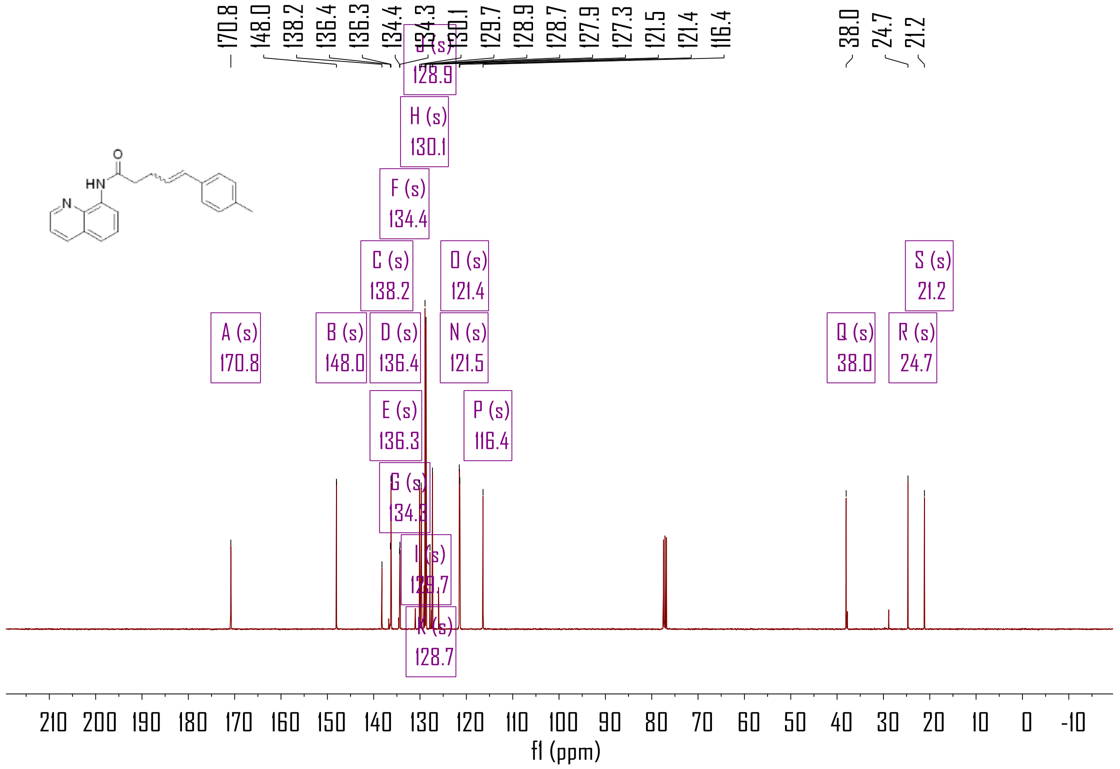

Supplementary Figure 50. ^{13}C NMR spectra of **1l**

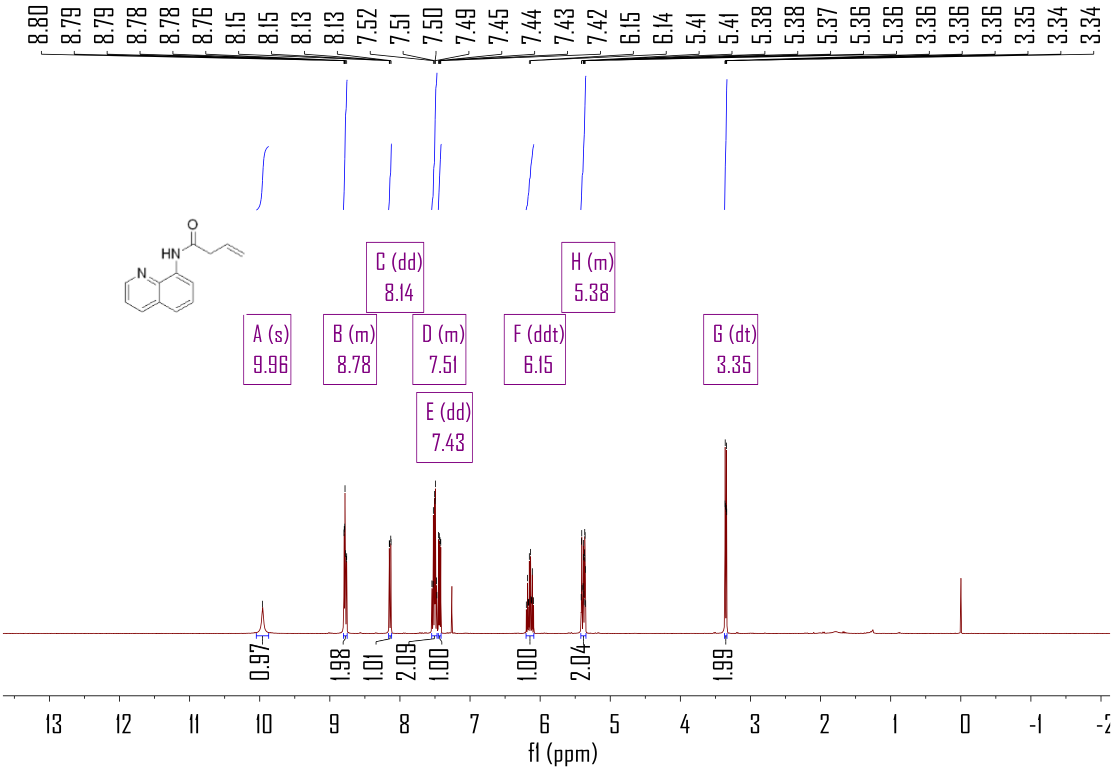

Supplementary Figure 51. ^{1}H NMR spectra of **1m**

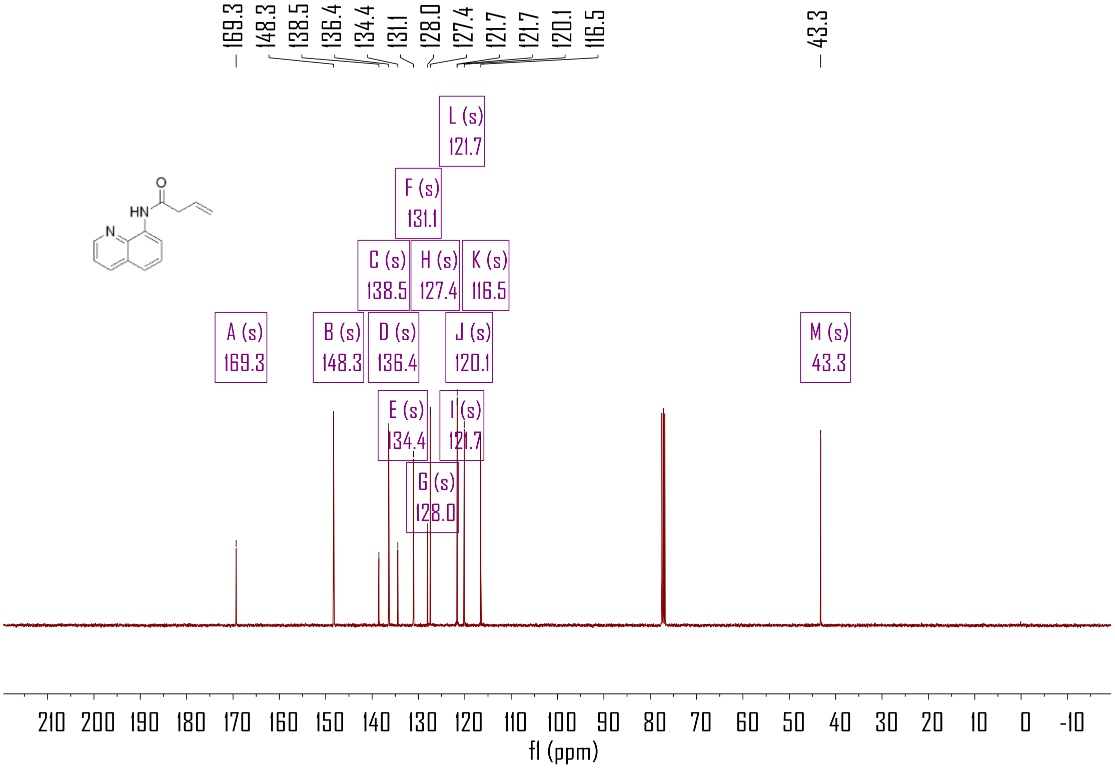

Supplementary Figure 52. ^{13}C NMR spectra of **1m**

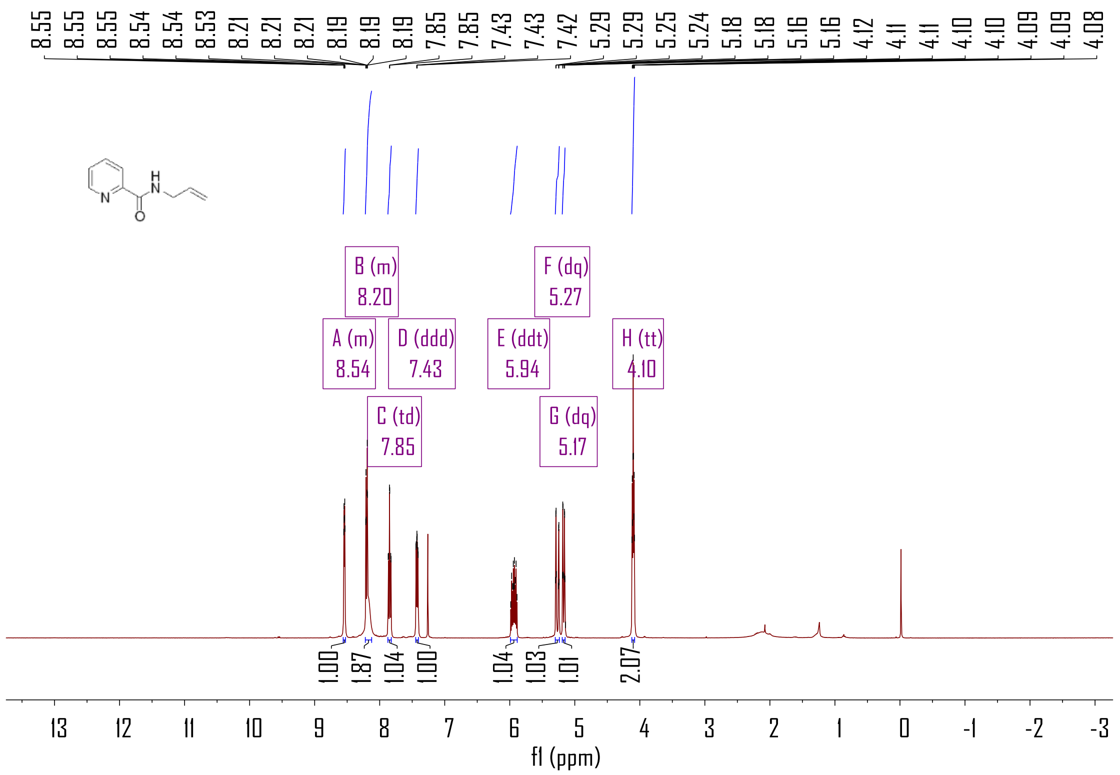

Supplementary Figure 53. ^{1}H NMR spectra of **1n**

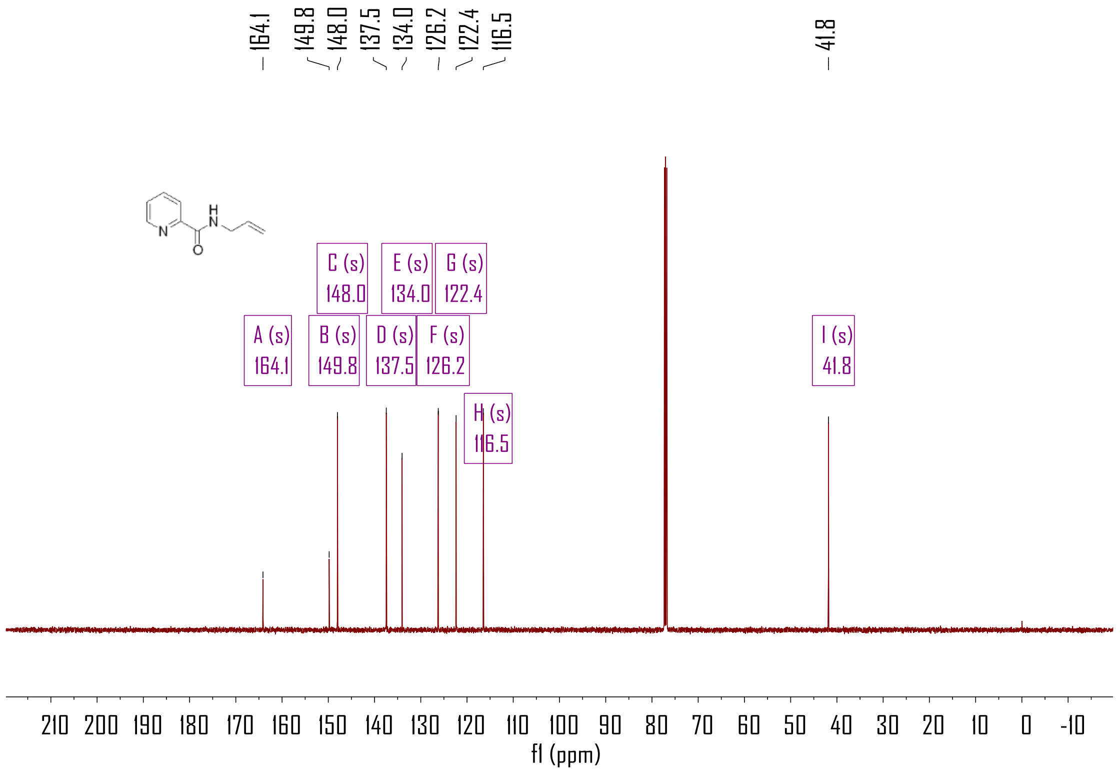

Supplementary Figure 54. ^{13}C NMR spectra of **1n**

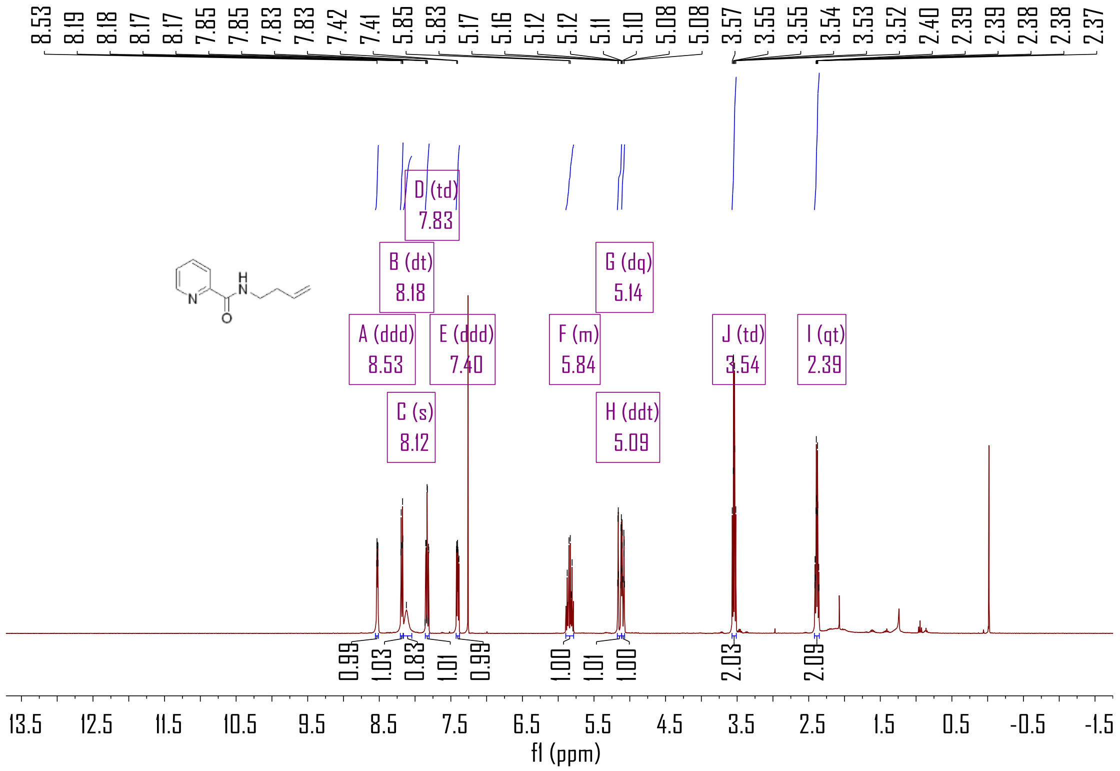

Supplementary Figure 55. ^{1}H NMR spectra of **1o**

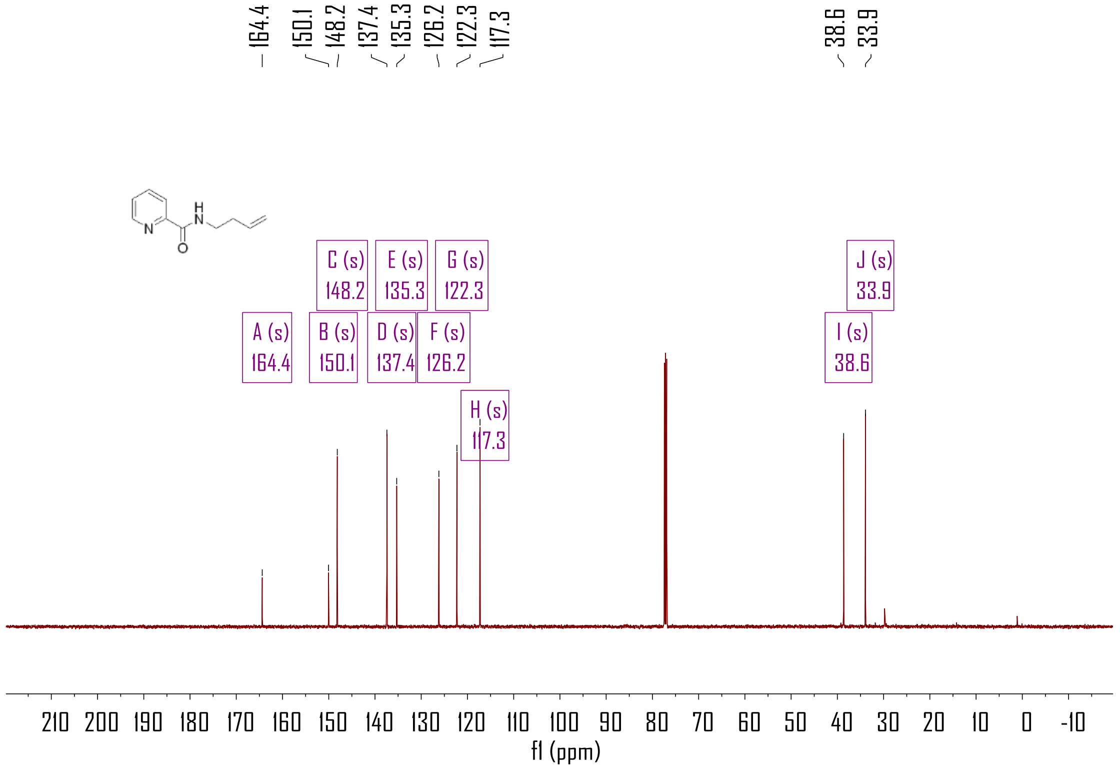

Supplementary Figure 56. ^{13}C NMR spectra of **1o**

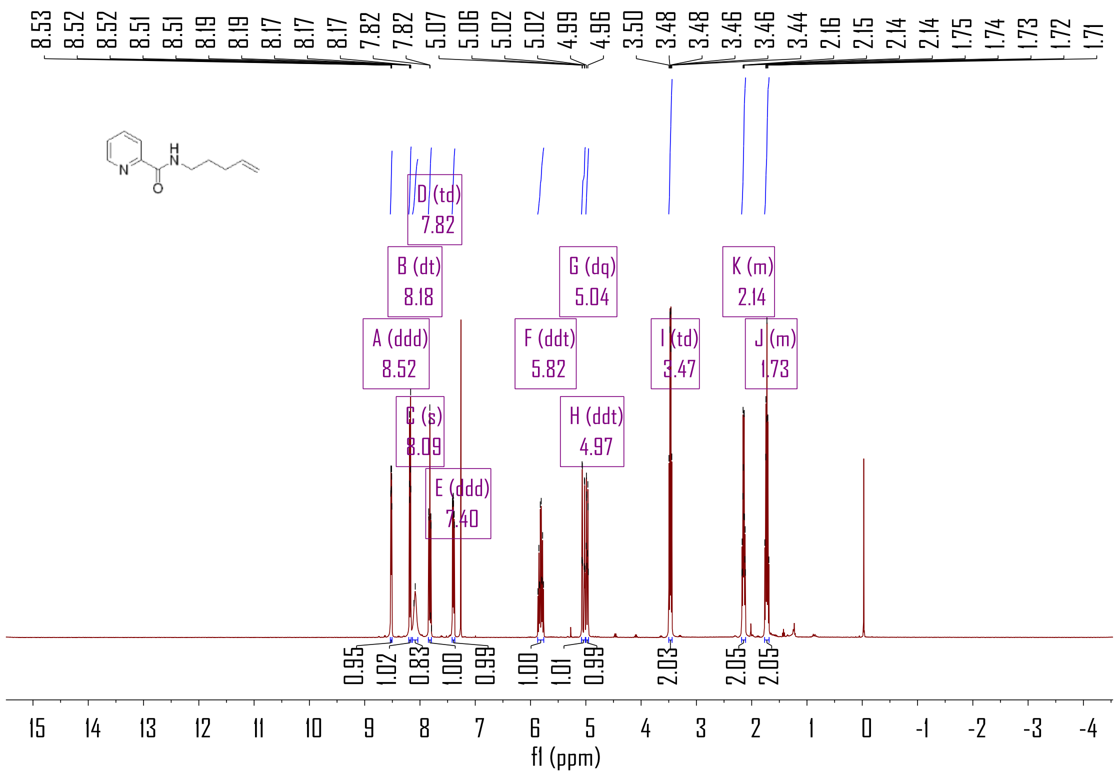

Supplementary Figure 57. ^{1}H NMR spectra of **1p**

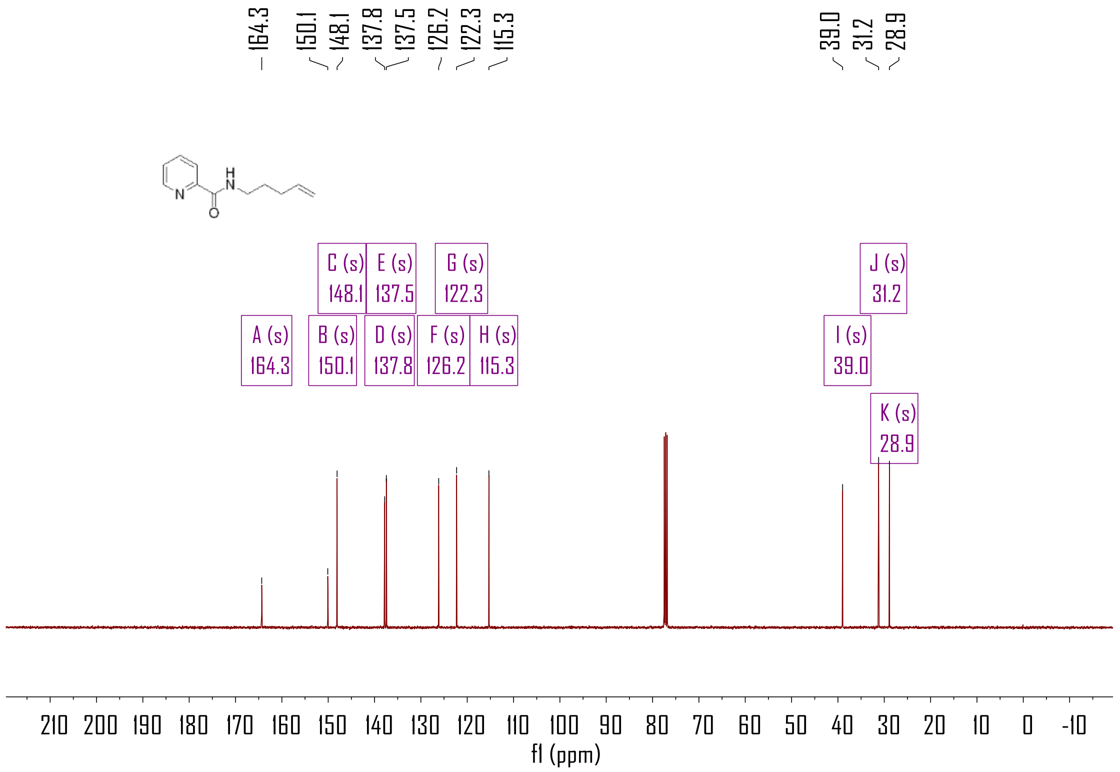

Supplementary Figure 58. ^{13}C NMR spectra of **1p**

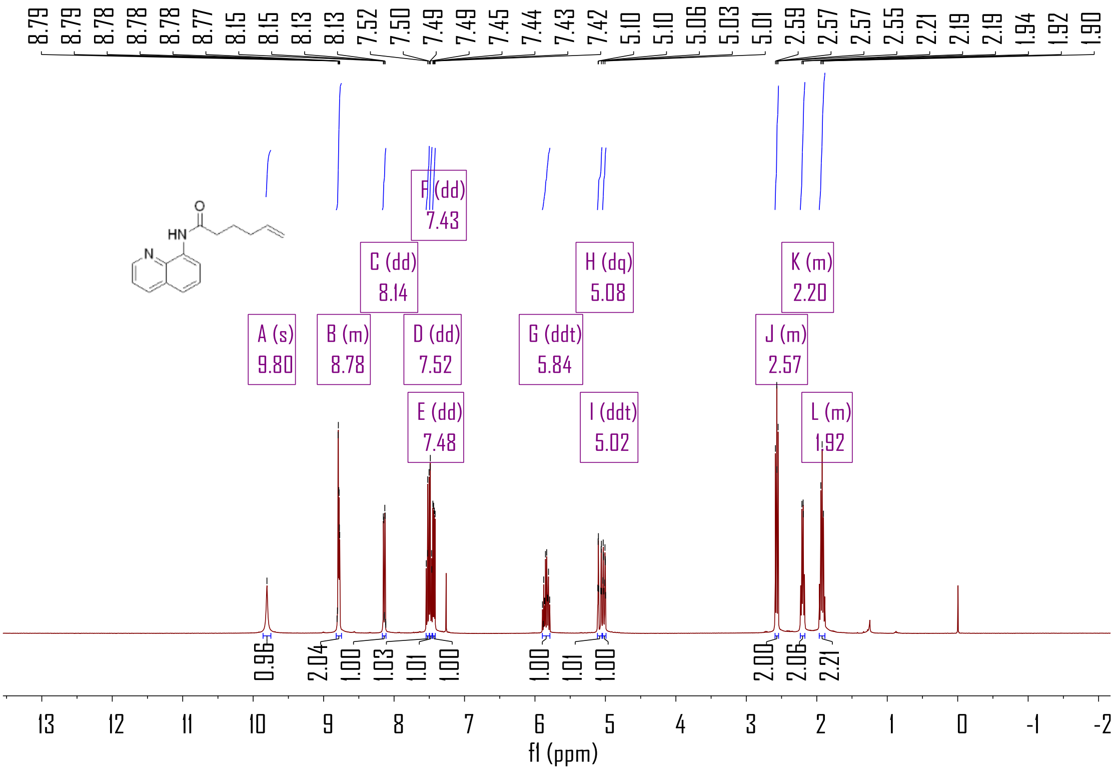

Supplementary Figure 59. ^{1}H NMR spectra of **1q**

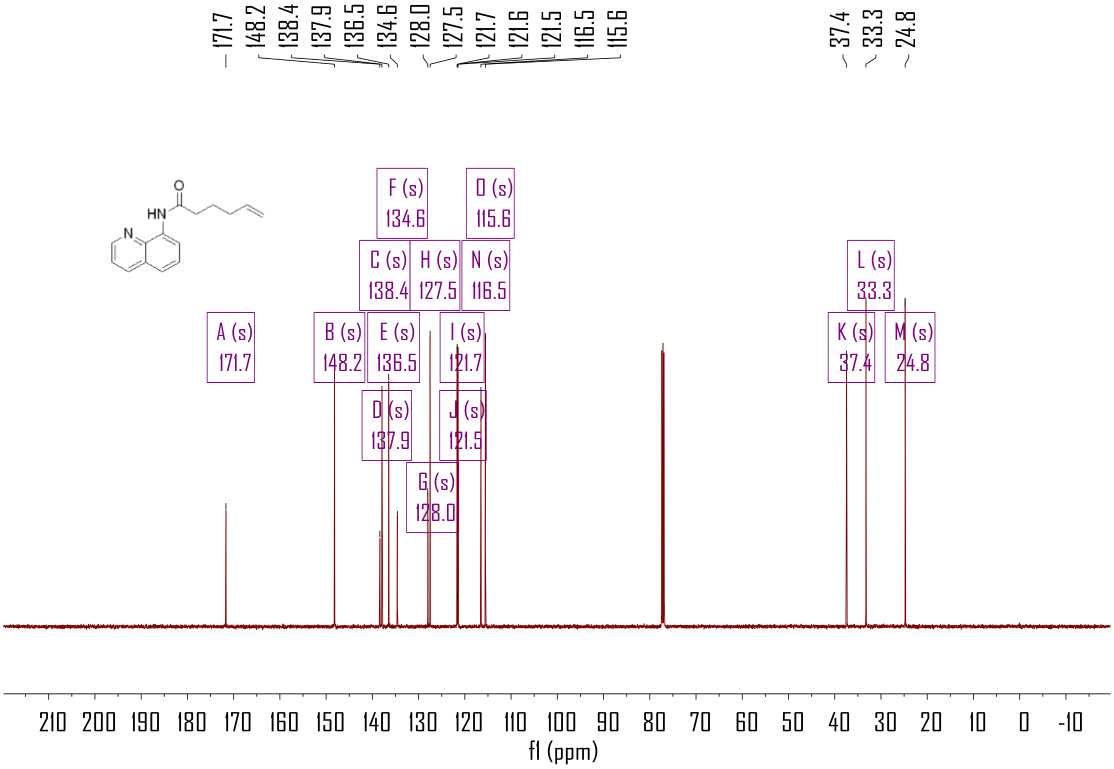

Supplementary Figure 60. ^{13}C NMR spectra of **1q**

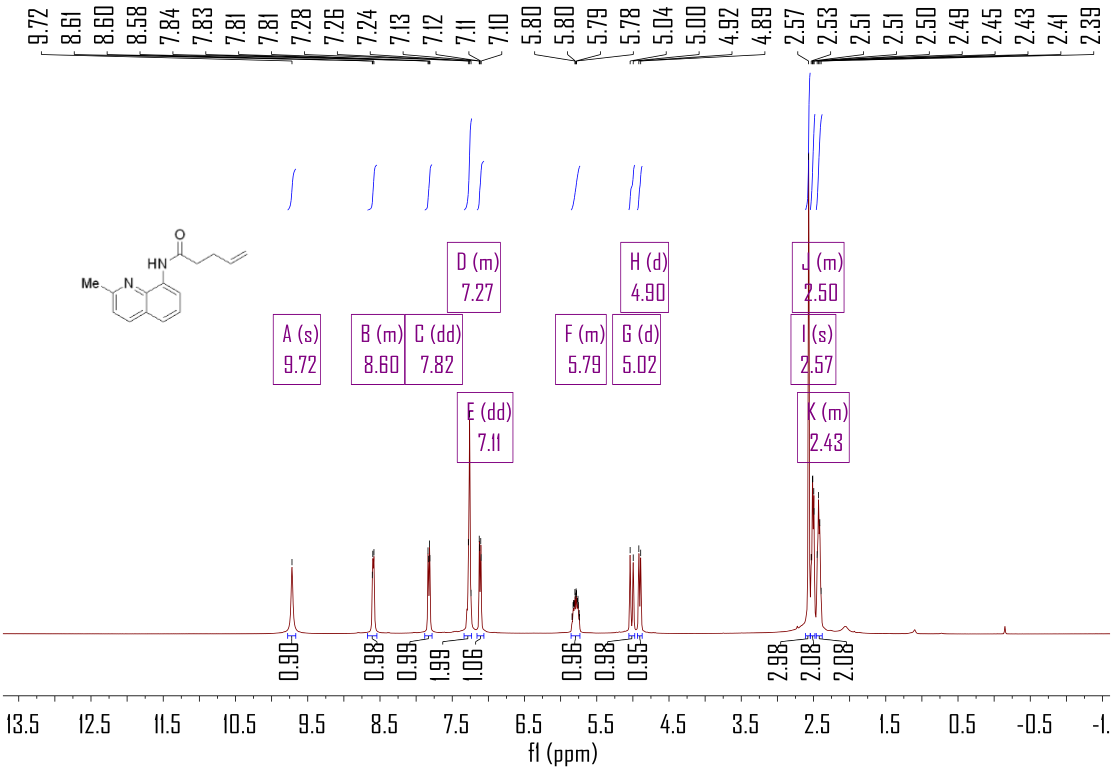

Supplementary Figure 61. ^{1}H NMR spectra of **1r**

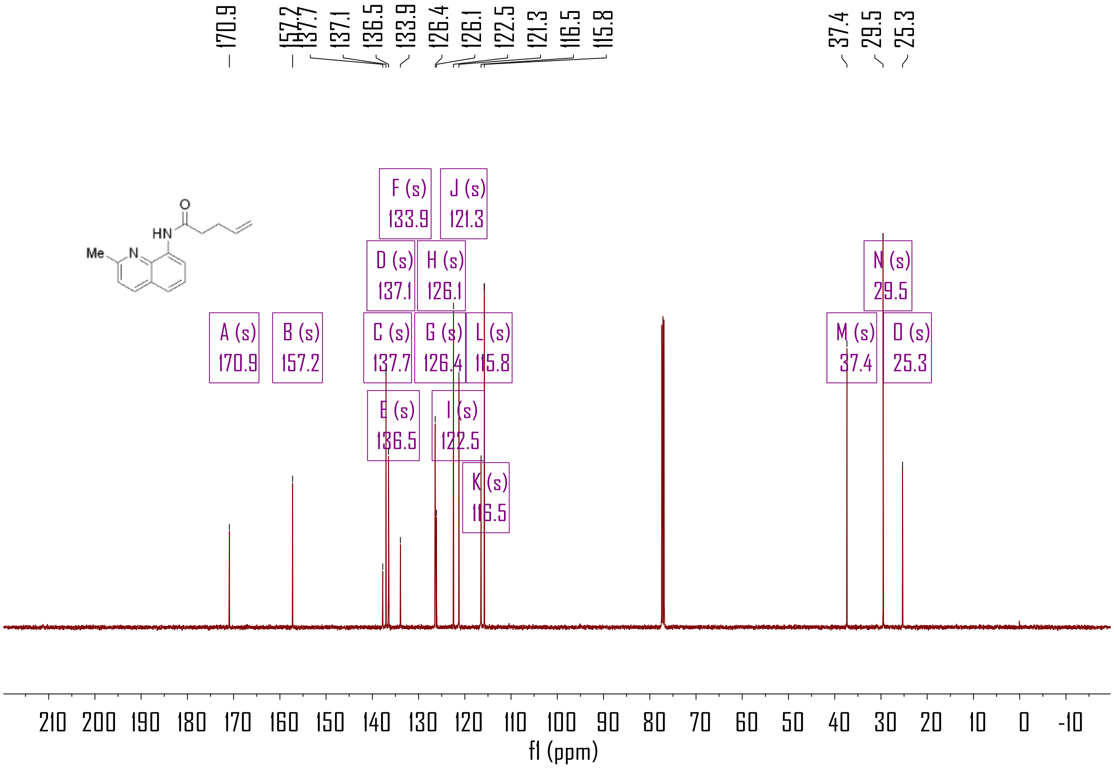

Supplementary Figure 62. ^{13}C NMR spectra of **1r**

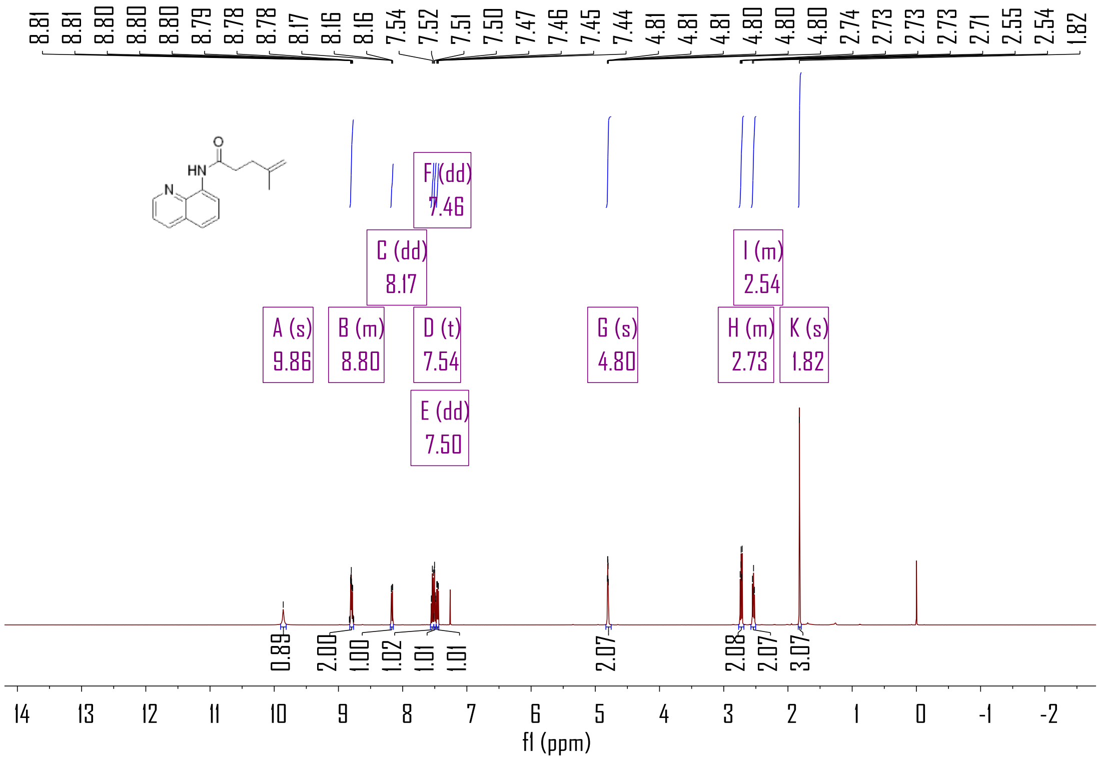

Supplementary Figure 63. ^{1}H NMR spectra of **1s**

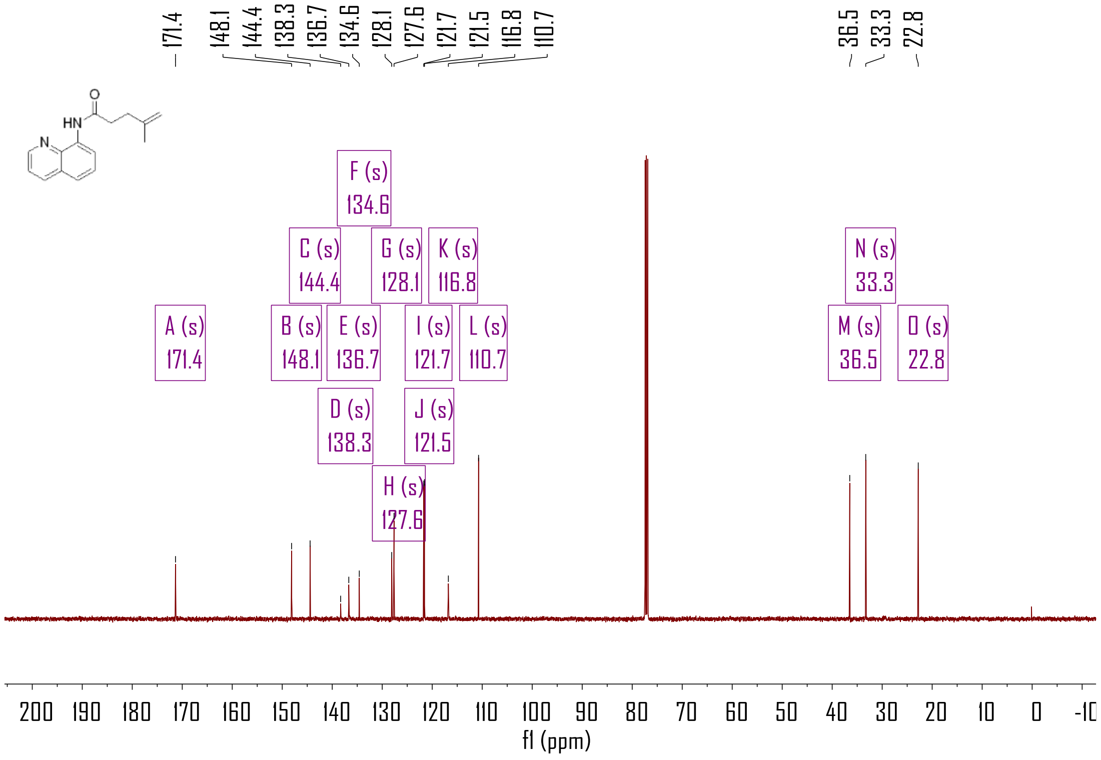

Supplementary Figure 64. ^{13}C NMR spectra of **1s**

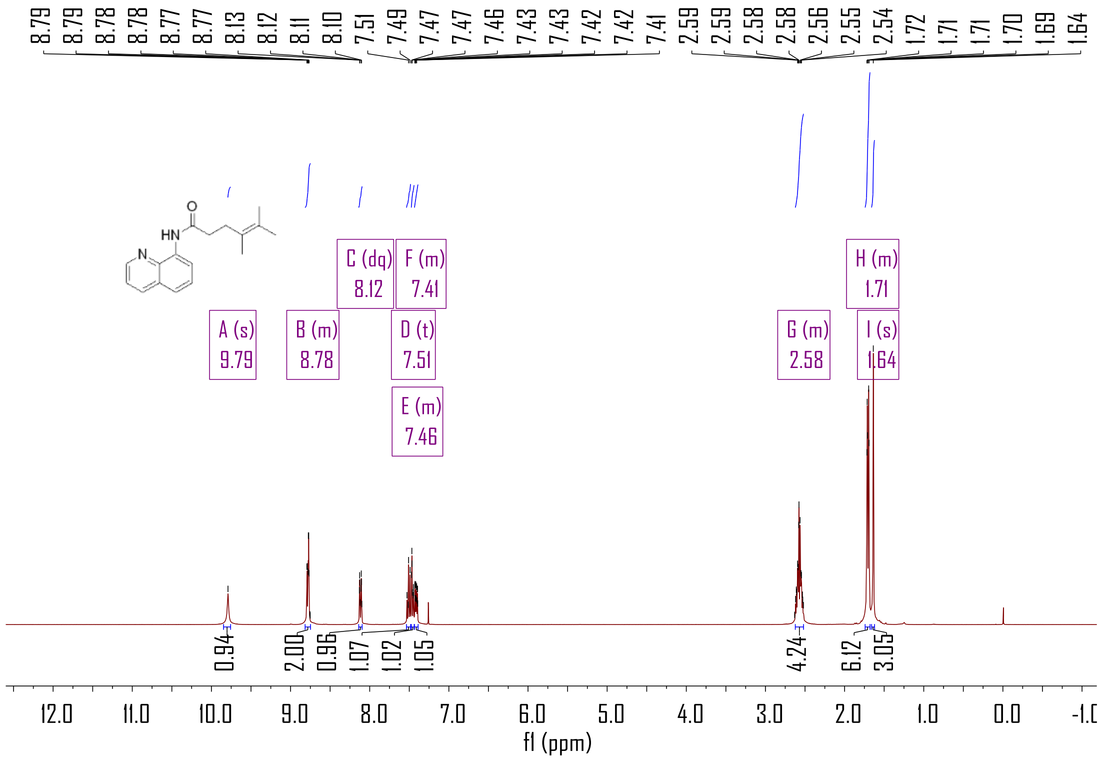

Supplementary Figure 65. ^{1}H NMR spectra of **1t**

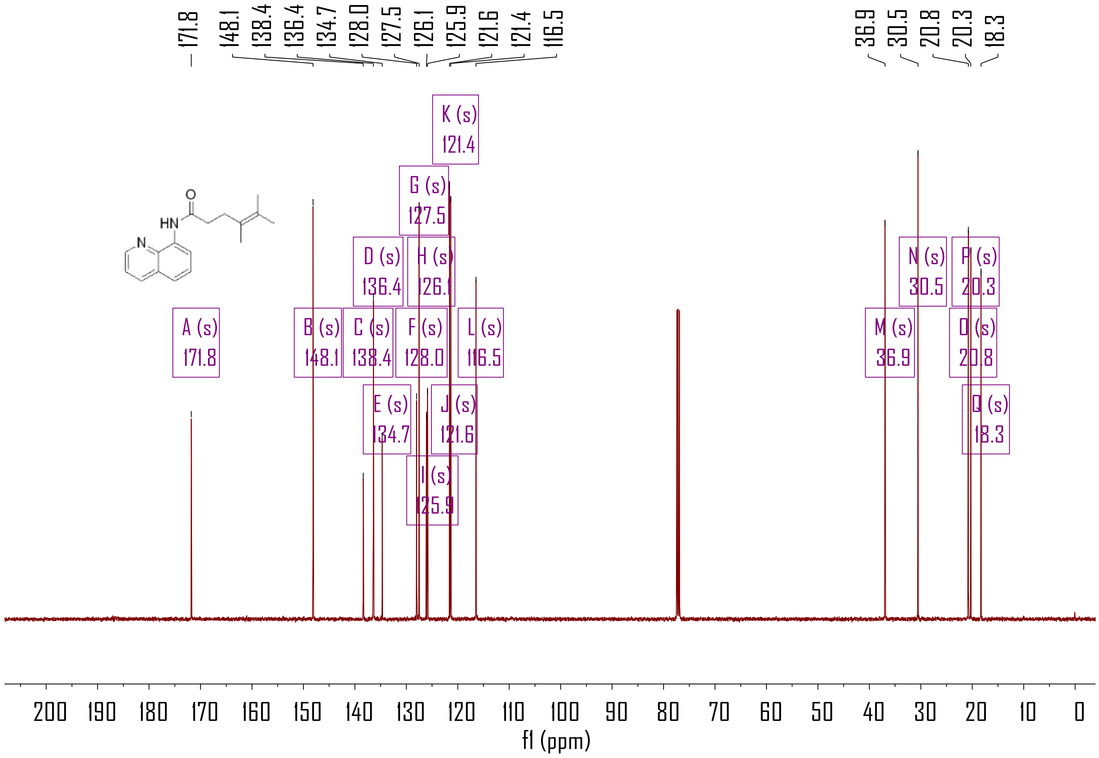

Supplementary Figure 66. ^{13}C NMR spectra of **1t**

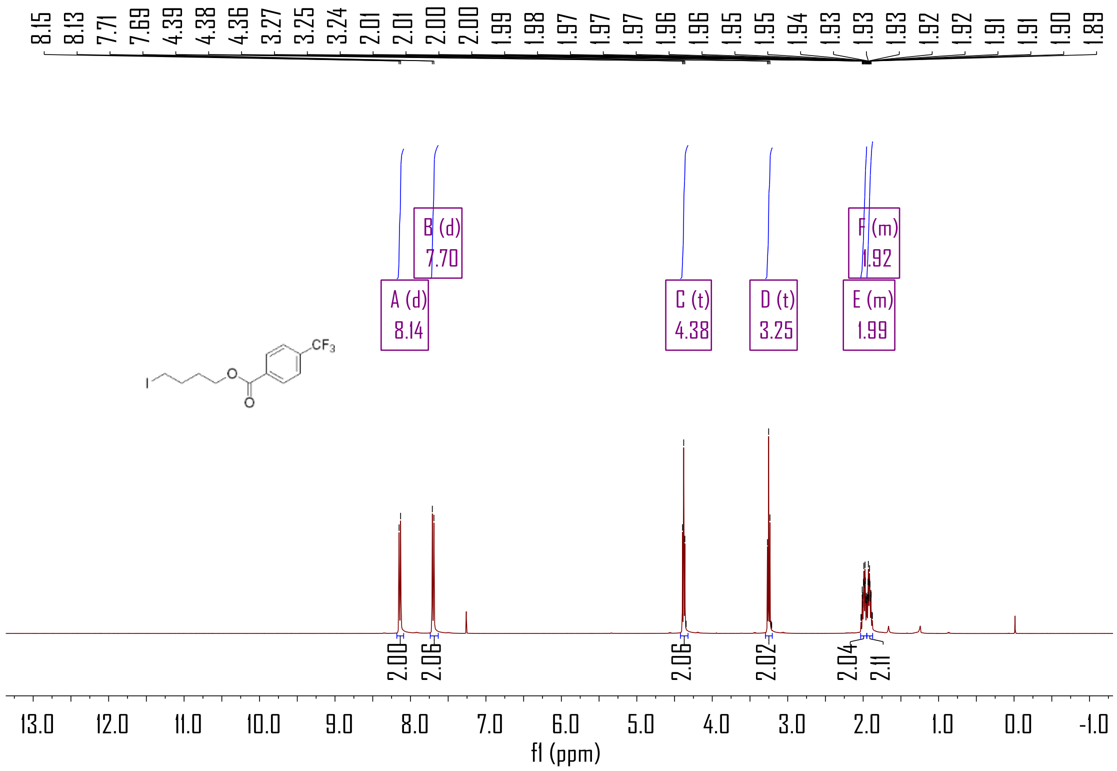

Supplementary Figure 67. ^{1}H NMR spectra of **2d**

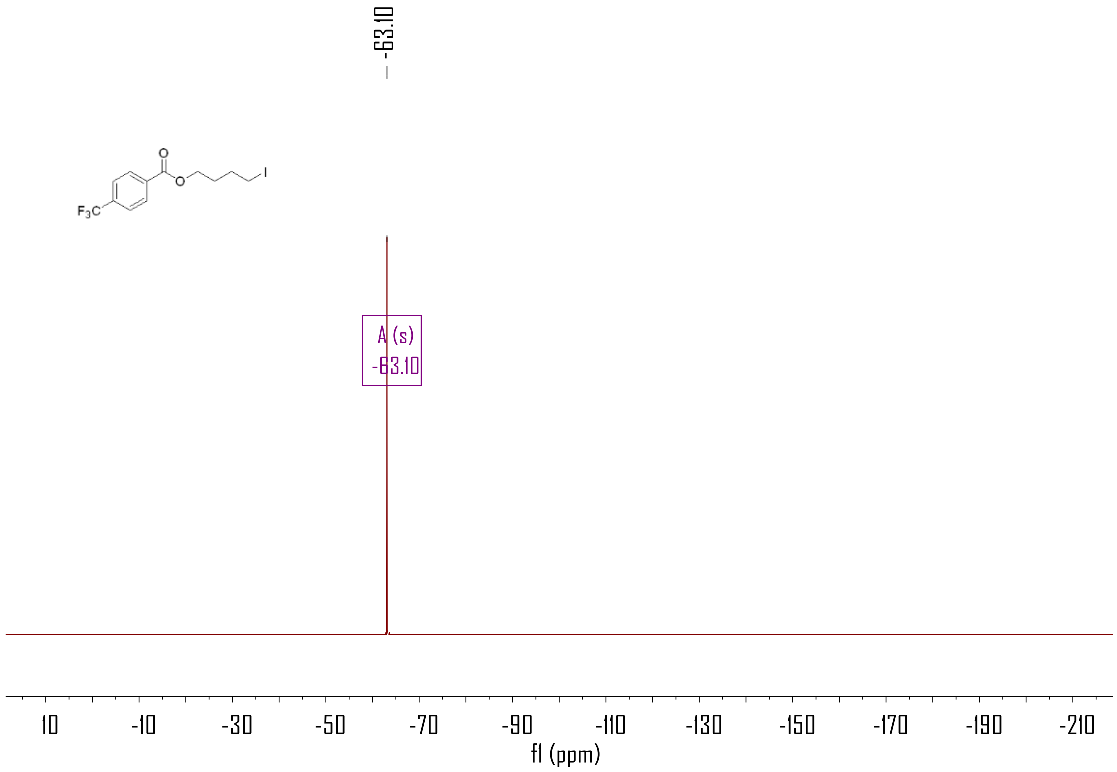

Supplementary Figure 68. ^{19}F NMR spectra of **2d**

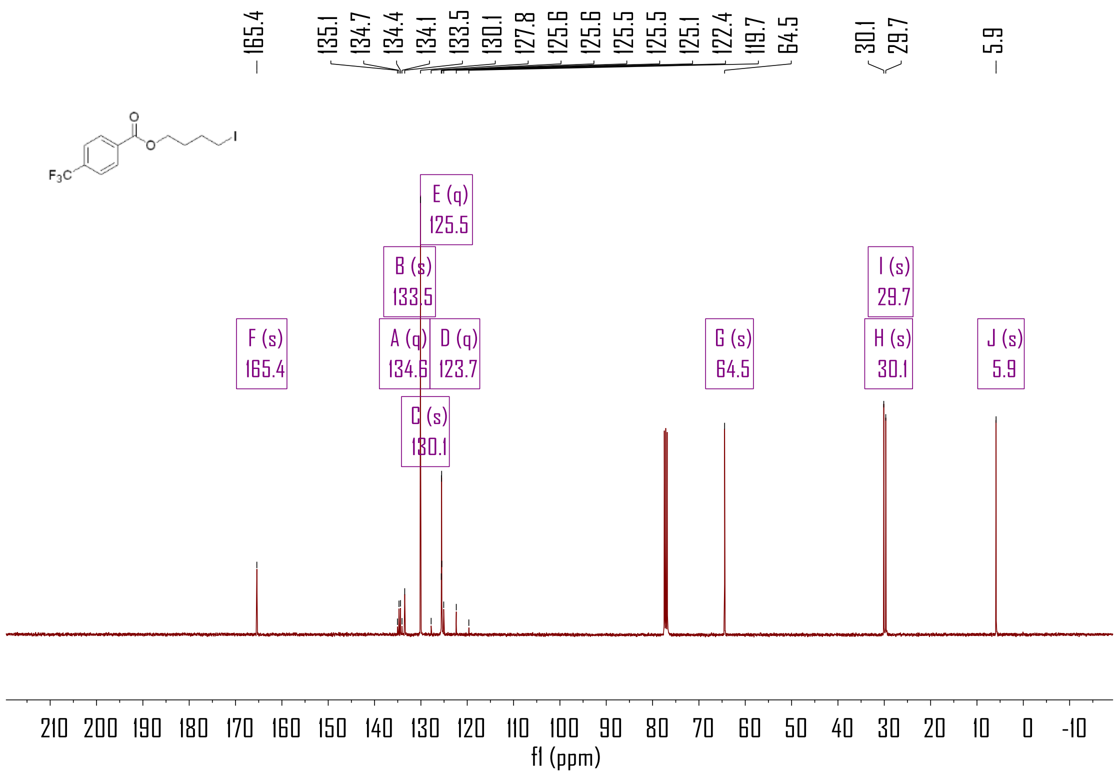

Supplementary Figure 69. ^{13}C NMR spectra of **2d**

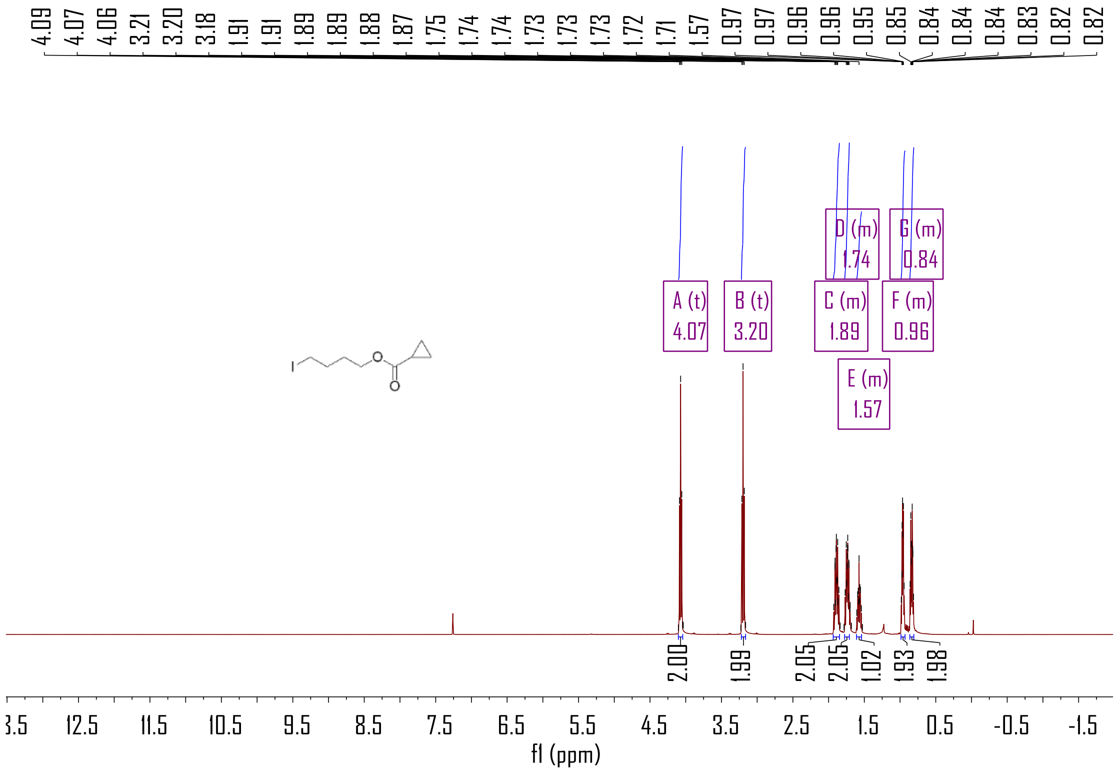

Supplementary Figure 70. ^{1}H NMR spectra of **2e**

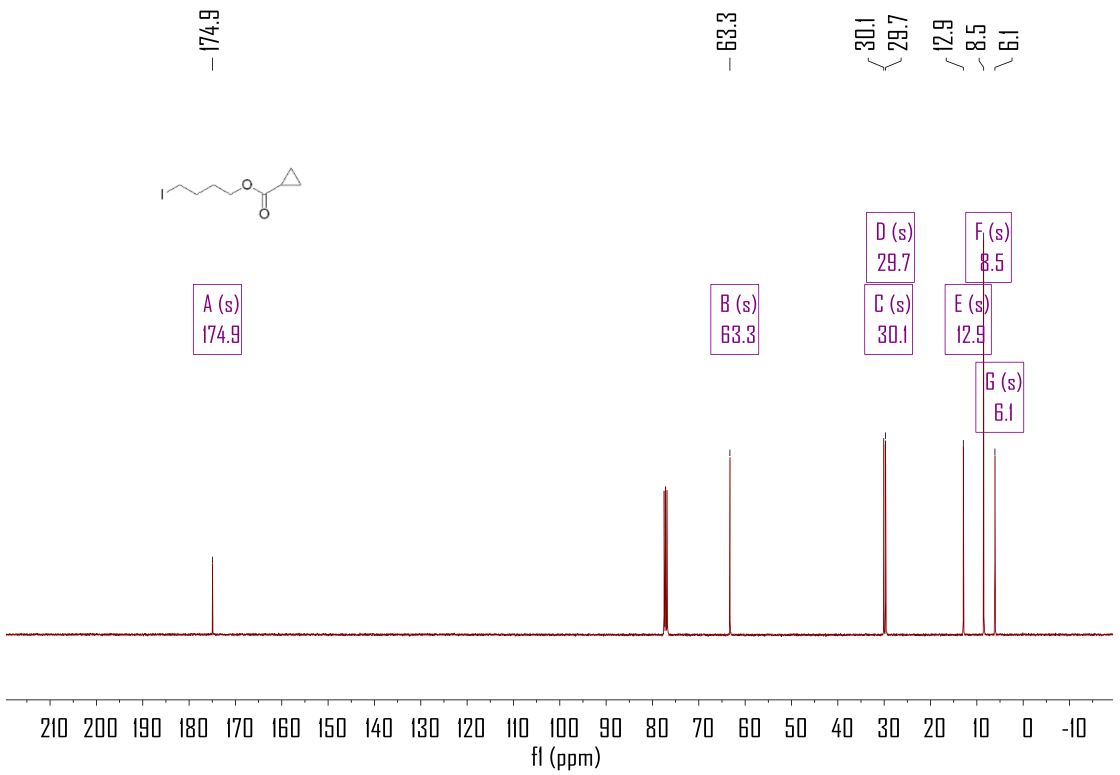

Supplementary Figure 71. ^{13}C NMR spectra of **2e**

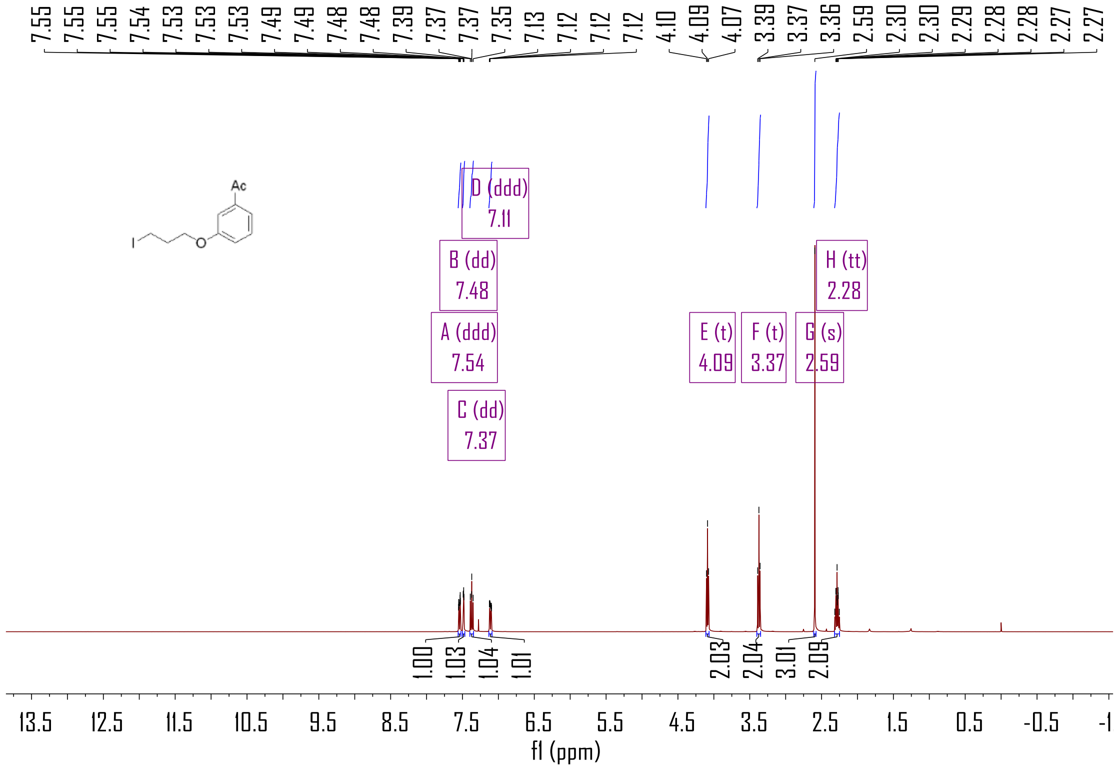

Supplementary Figure 72. ^{1}H NMR spectra of **2k**

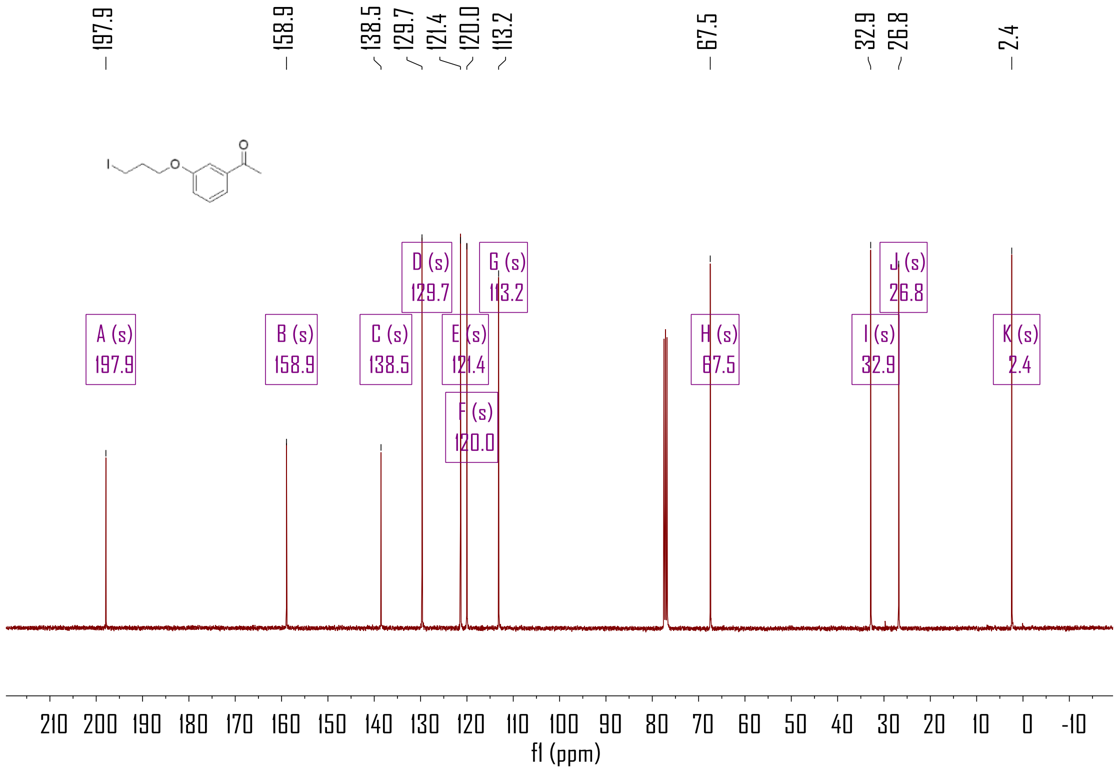

Supplementary Figure 73. ^{13}C NMR spectra of **2k**

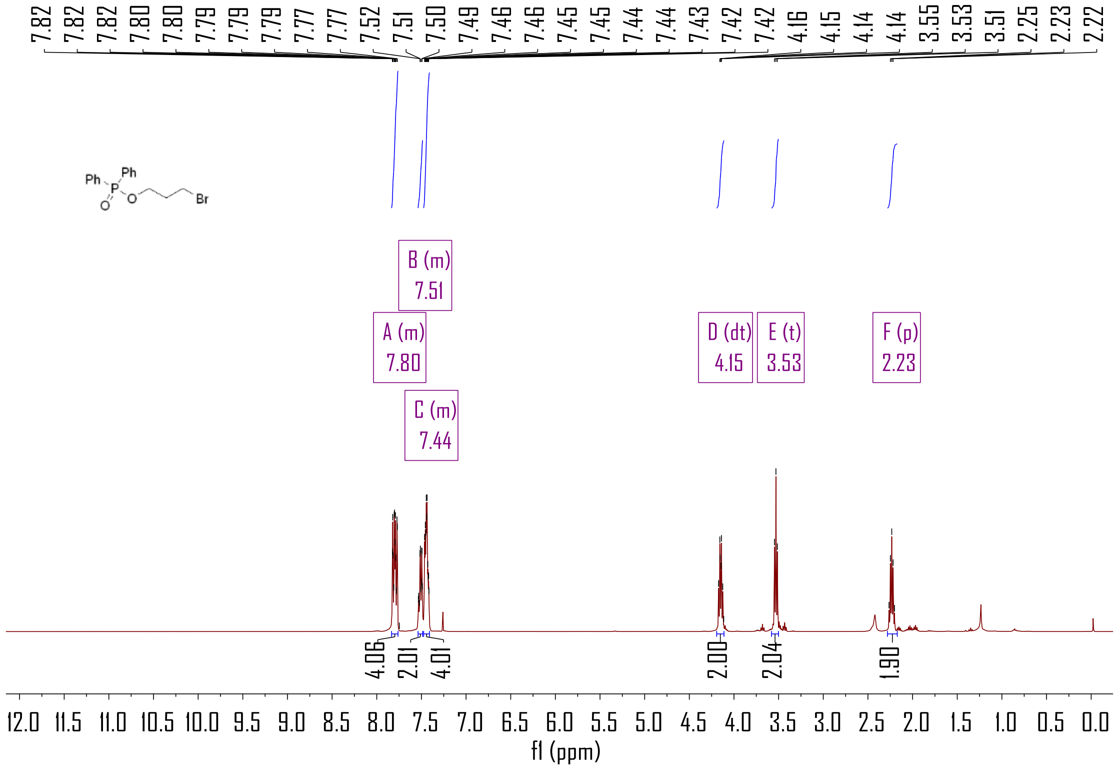

Supplementary Figure 74. ^{1}H NMR spectra of **3m**

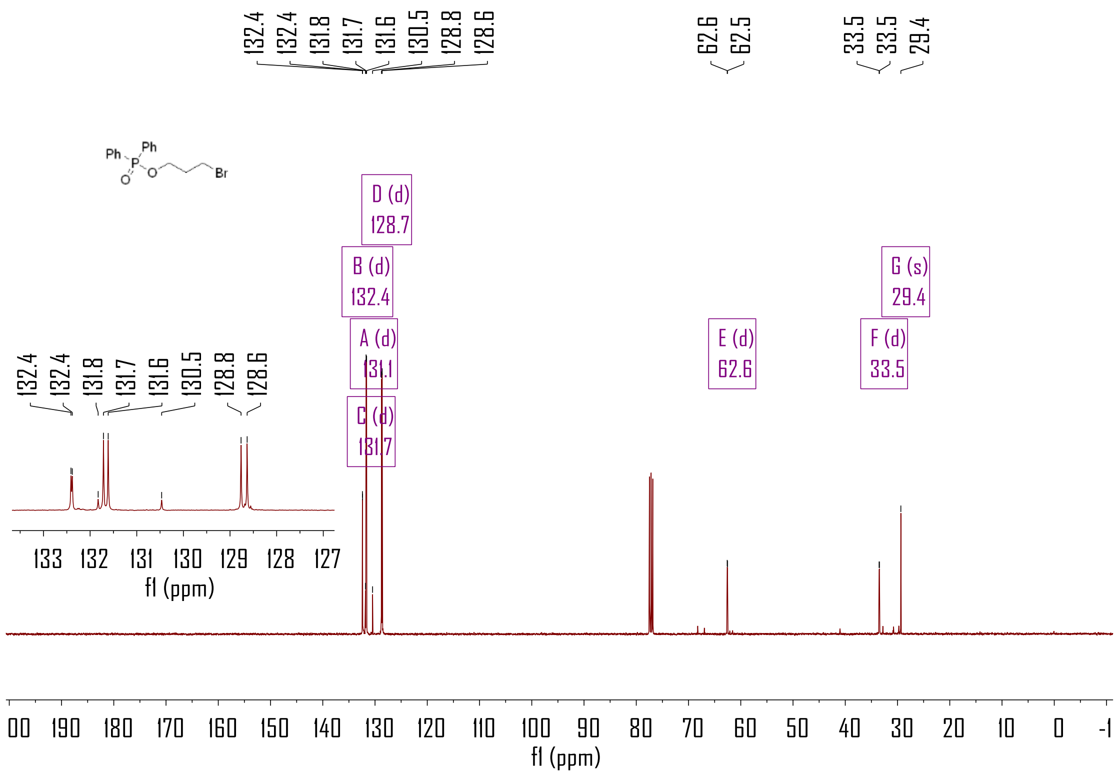

Supplementary Figure 75. ^{13}C NMR spectra of **3m**

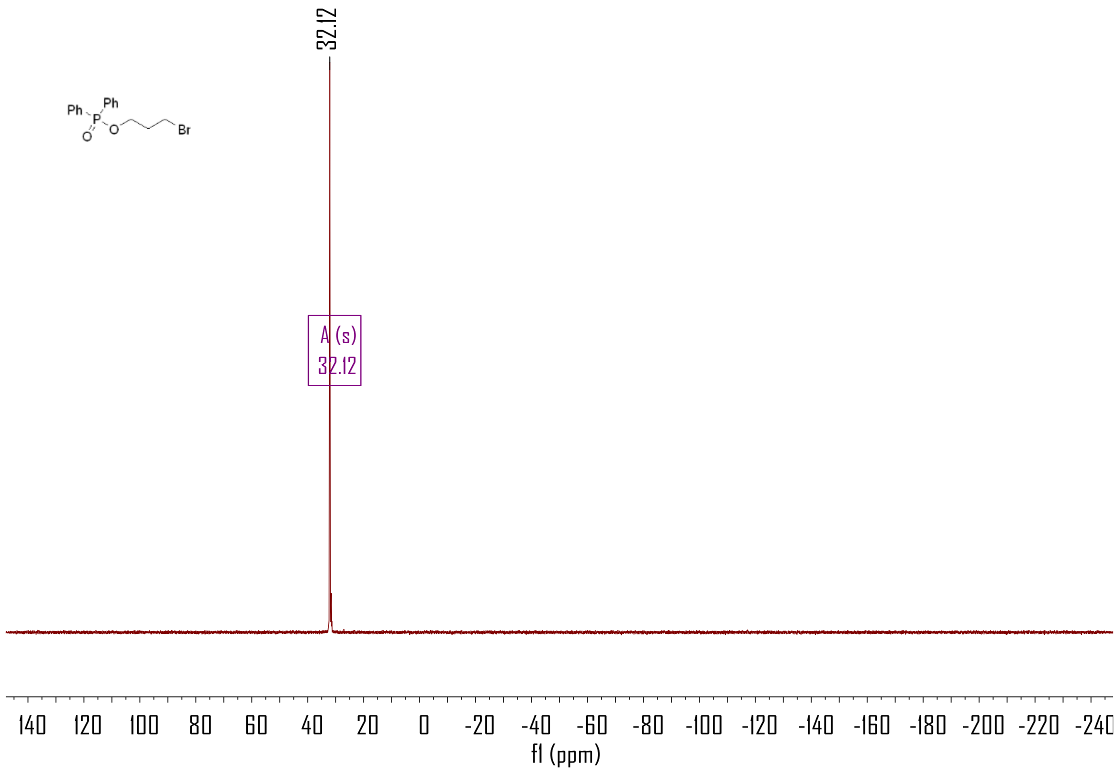

Supplementary Figure 76. ^{31}P NMR spectra of **3m**

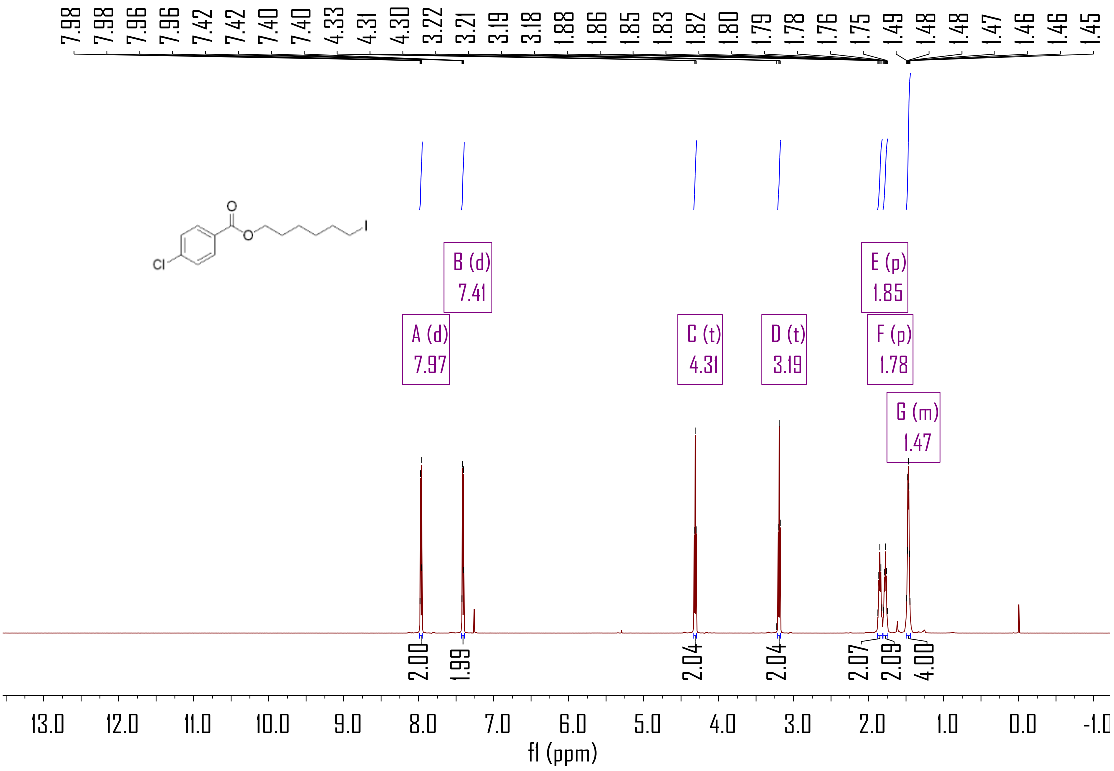

Supplementary Figure 77. ^{1}H NMR spectra of **2q**

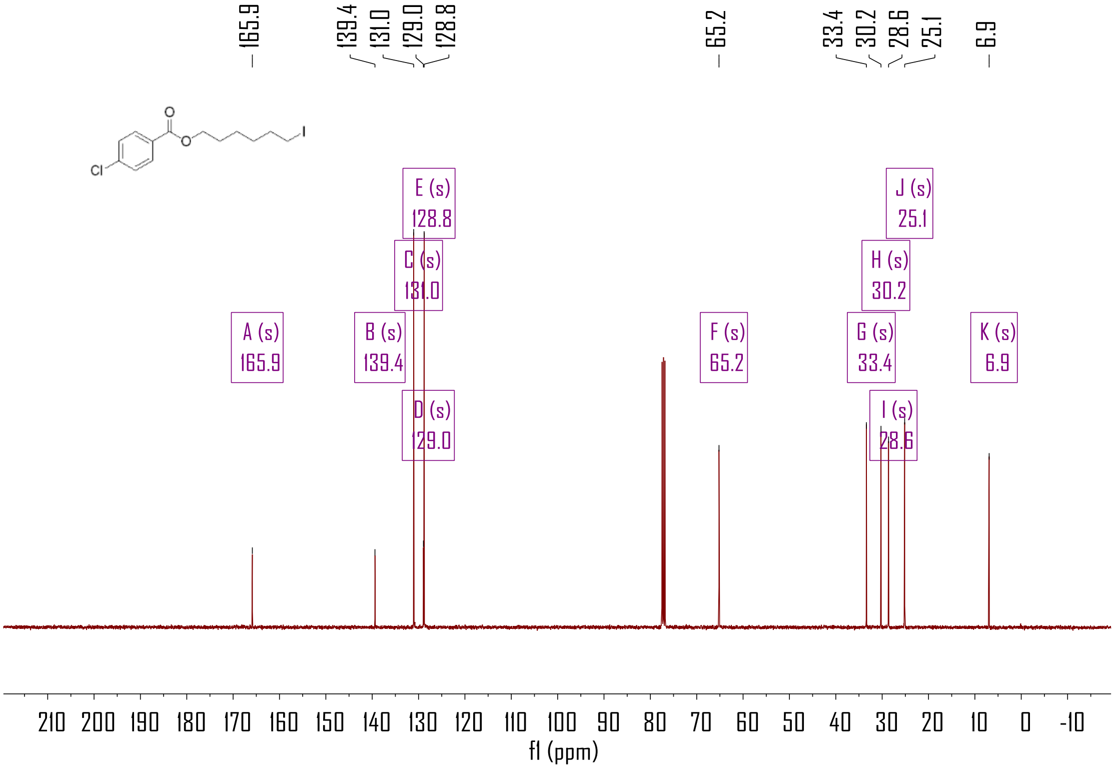

Supplementary Figure 78. ^{13}C NMR spectra of **2q**

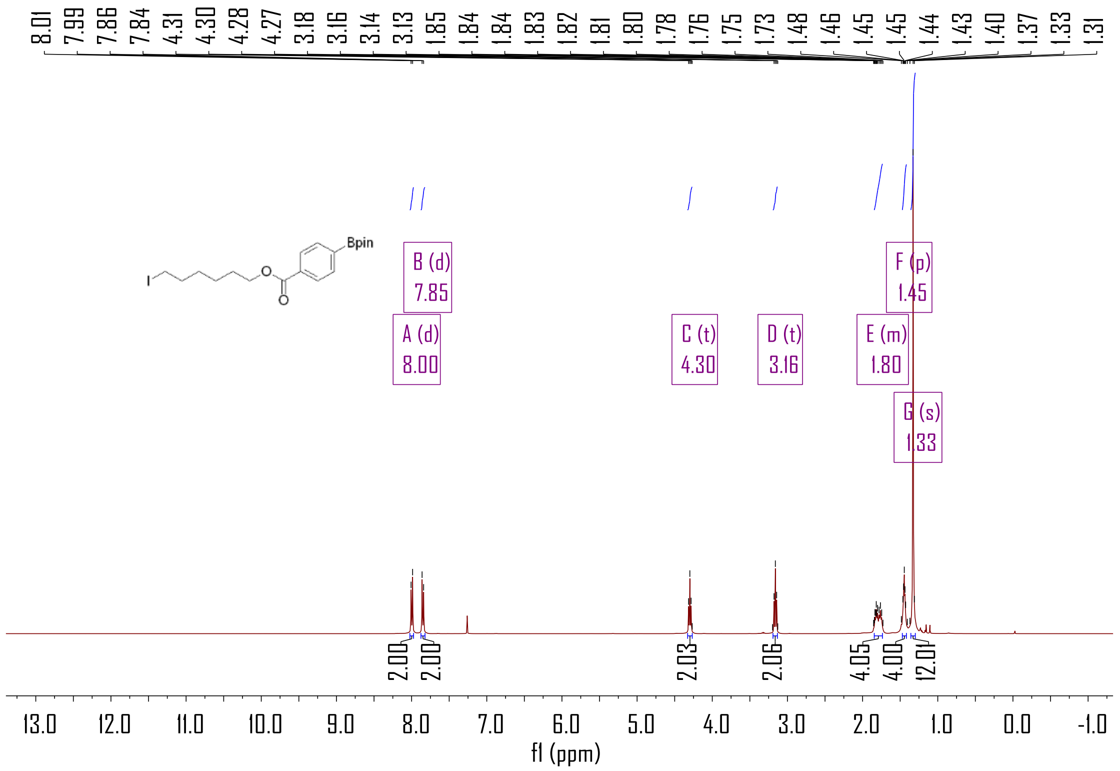

Supplementary Figure 79. ^{1}H NMR spectra of **2s**

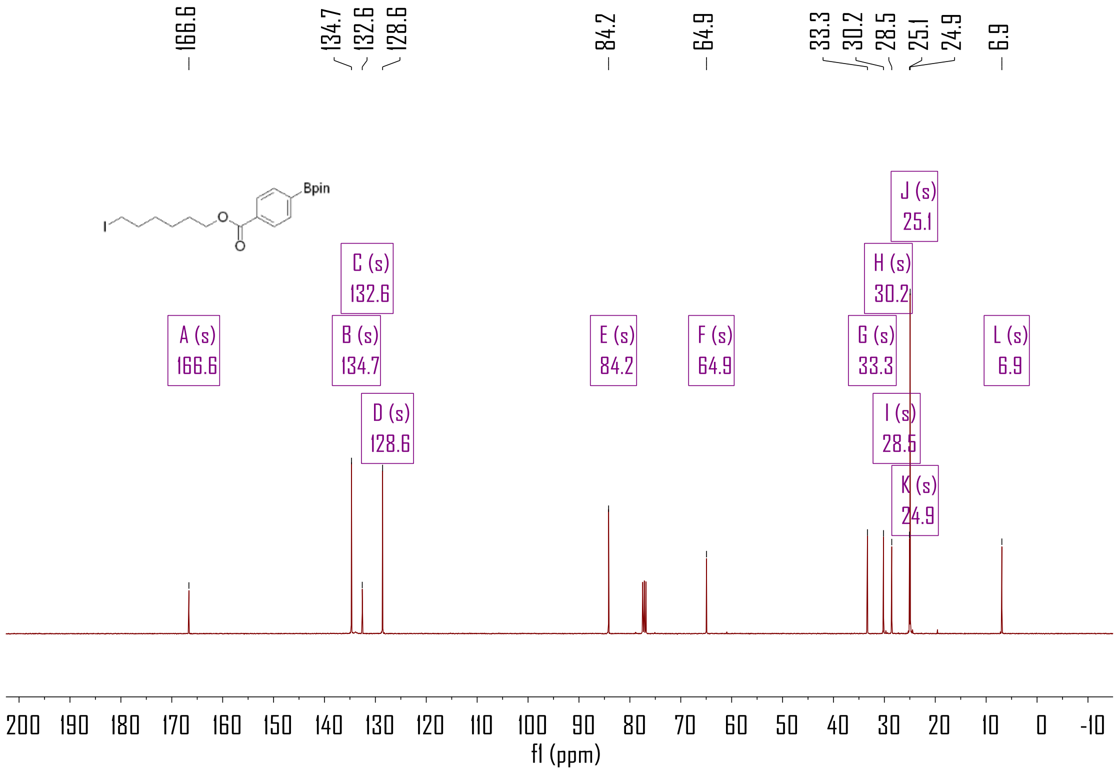

Supplementary Figure 80. ^{13}C NMR spectra of **2s**

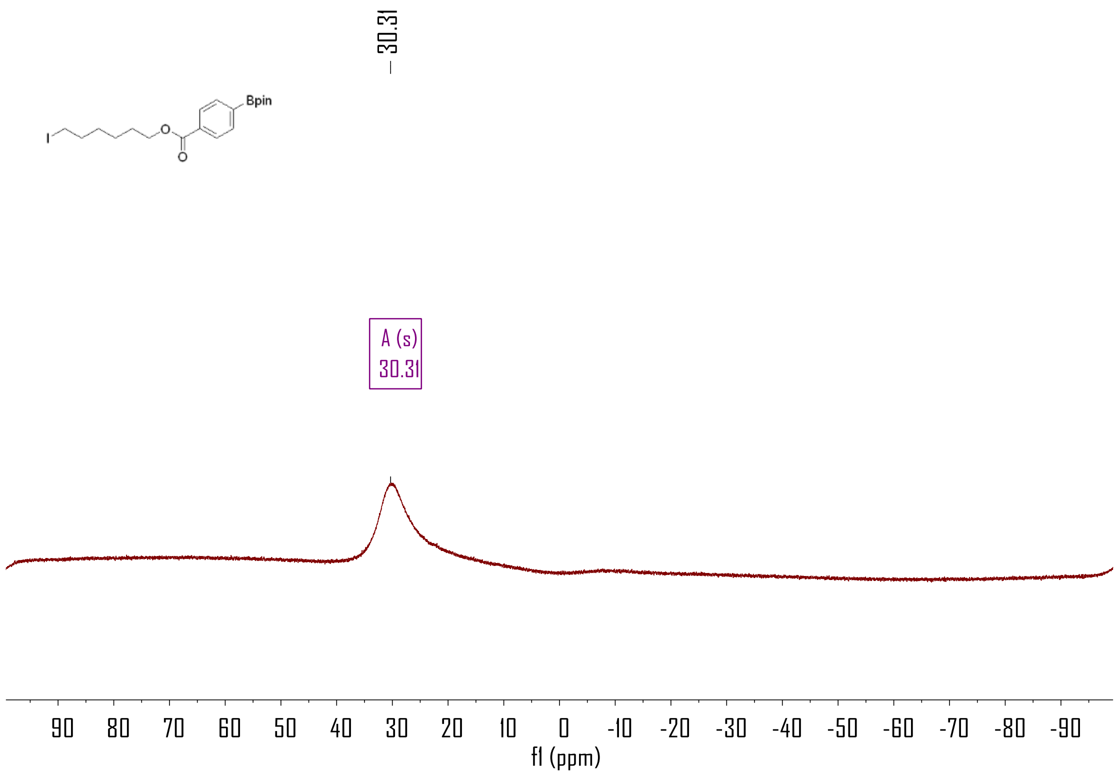

Supplementary Figure 81. ^{11}B NMR spectra of **2s**

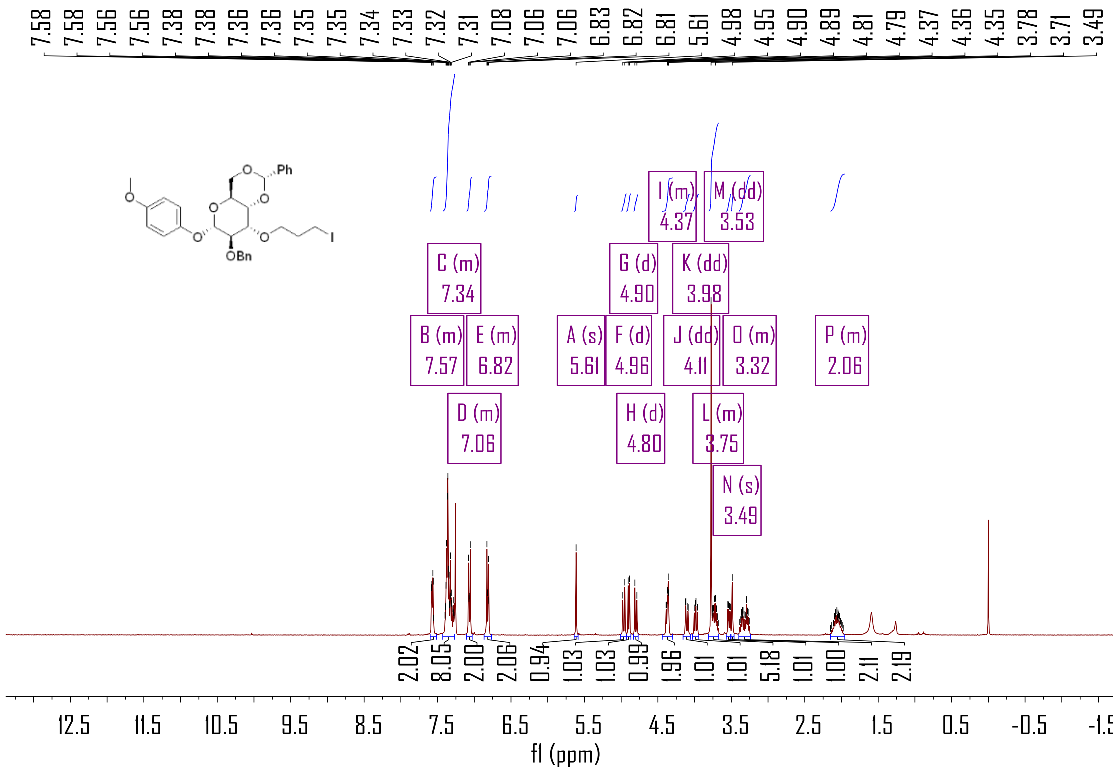

Supplementary Figure 82. ^{1}H NMR spectra of **2x**

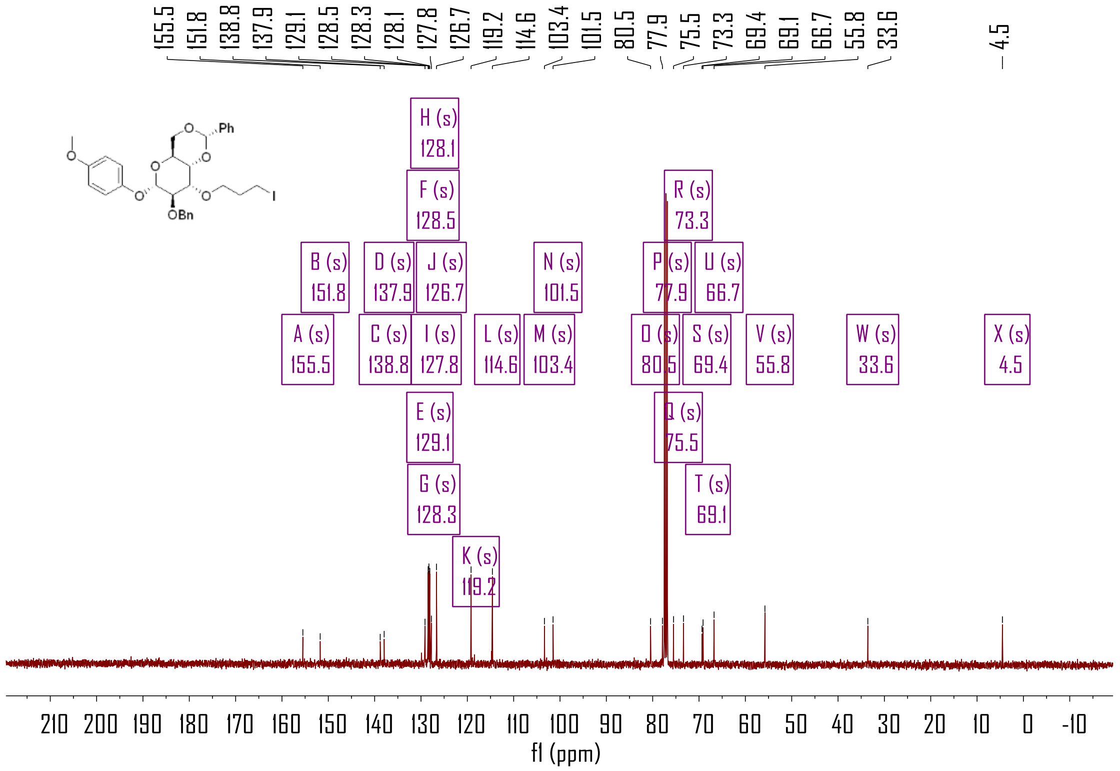

Supplementary Figure 83. ^{13}C NMR spectra of **2x**

NMR spectra of products

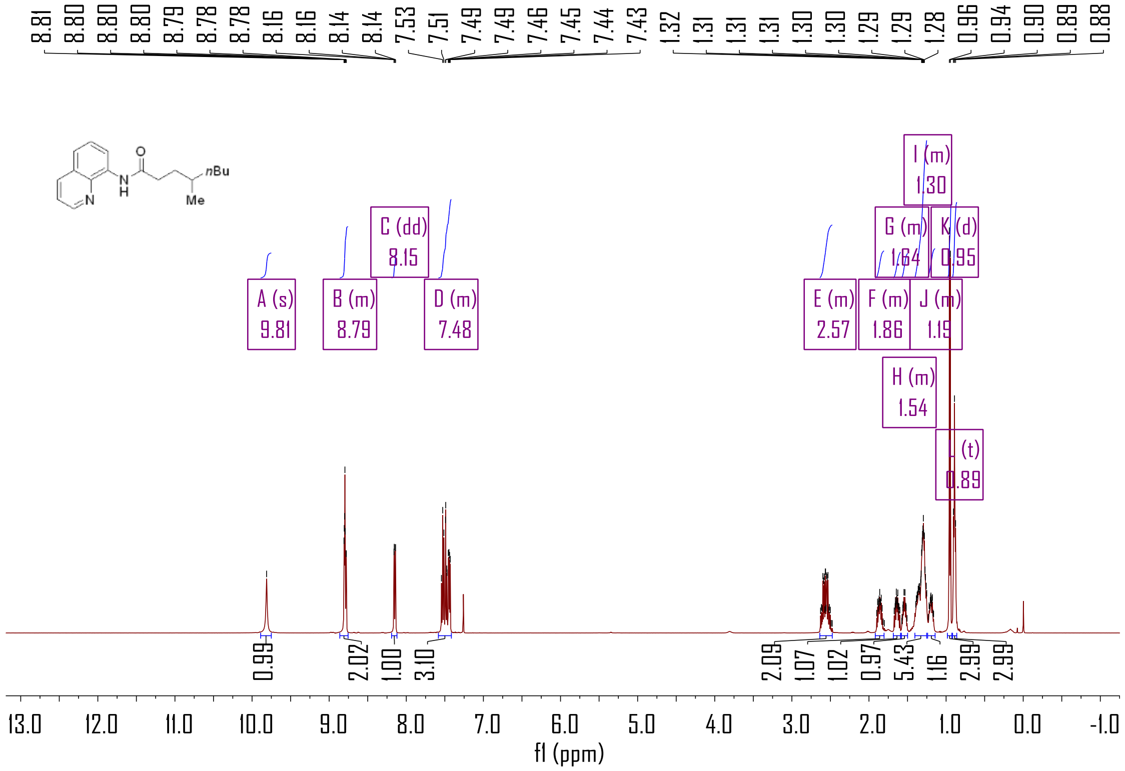

Supplementary Figure 84. ^{1}H NMR spectra of **4aa**

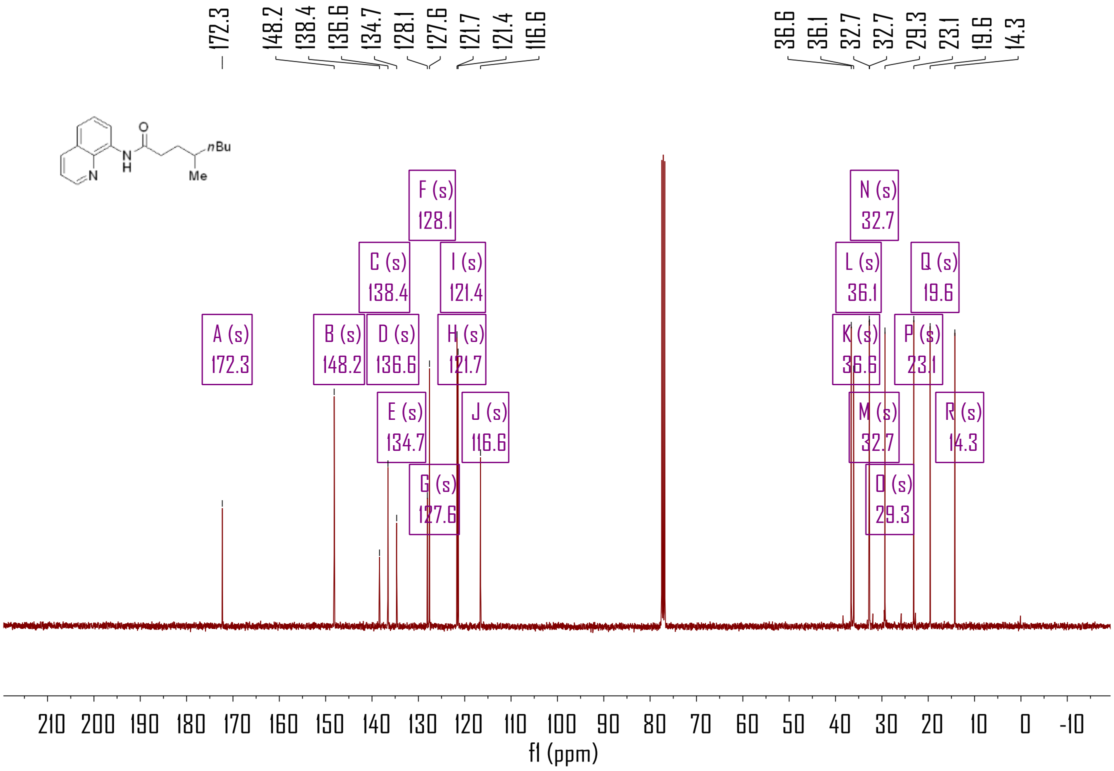

Supplementary Figure 85. ^{13}C NMR spectra of **4aa**

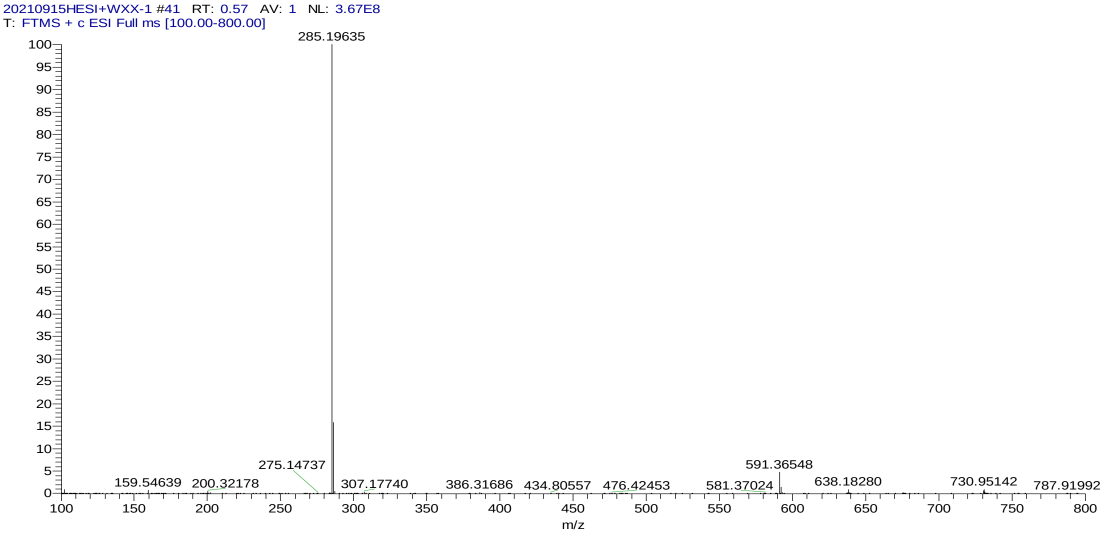

Supplementary Figure 86. HRMS spectra of **4aa**

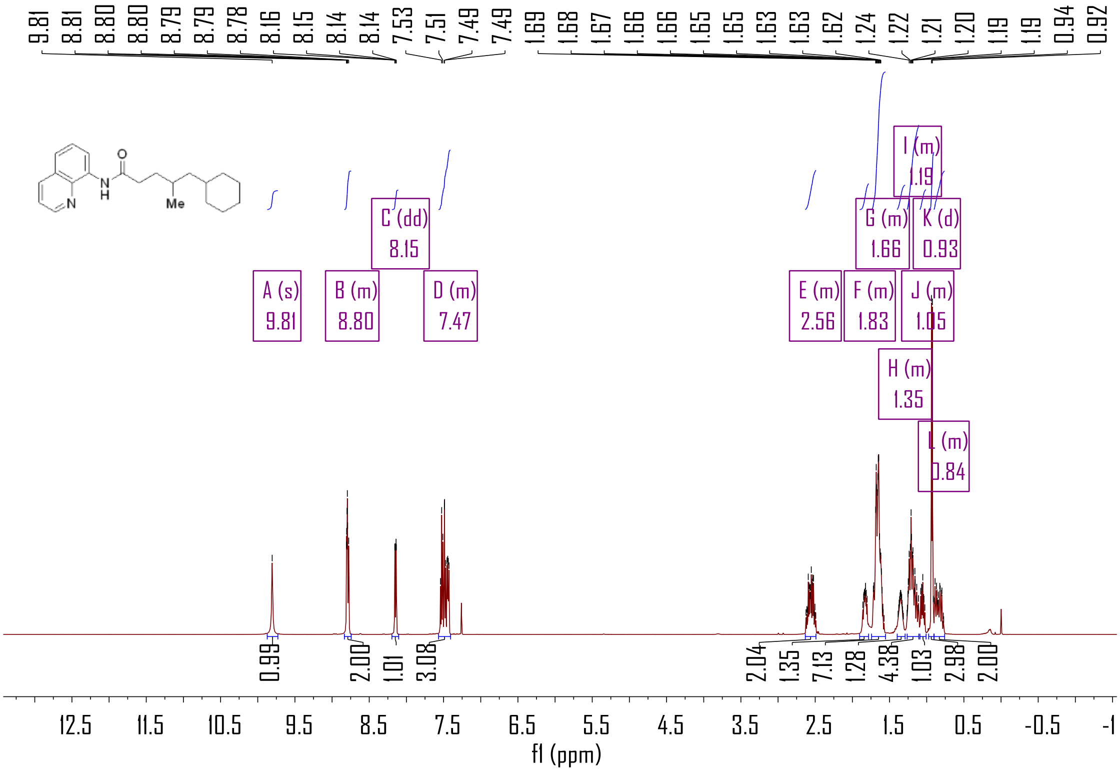

Supplementary Figure 87. ^{1}H NMR spectra of **4ab**

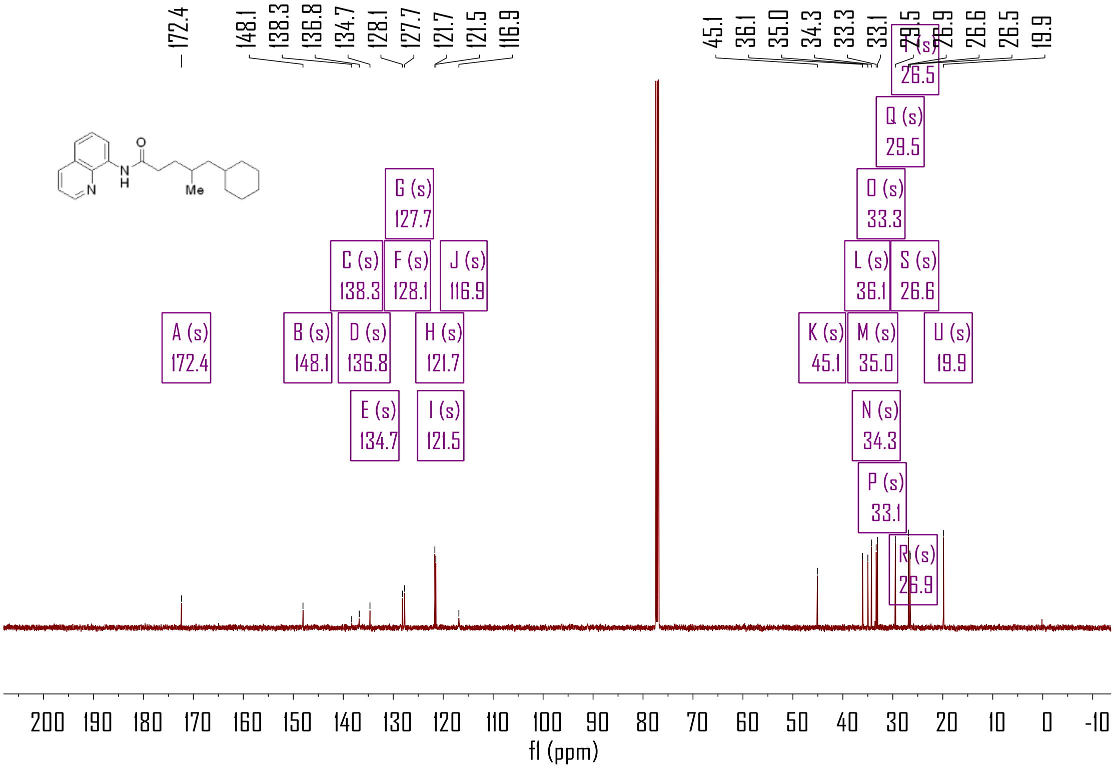

Supplementary Figure 88. ^{13}C NMR spectra of **4ab**

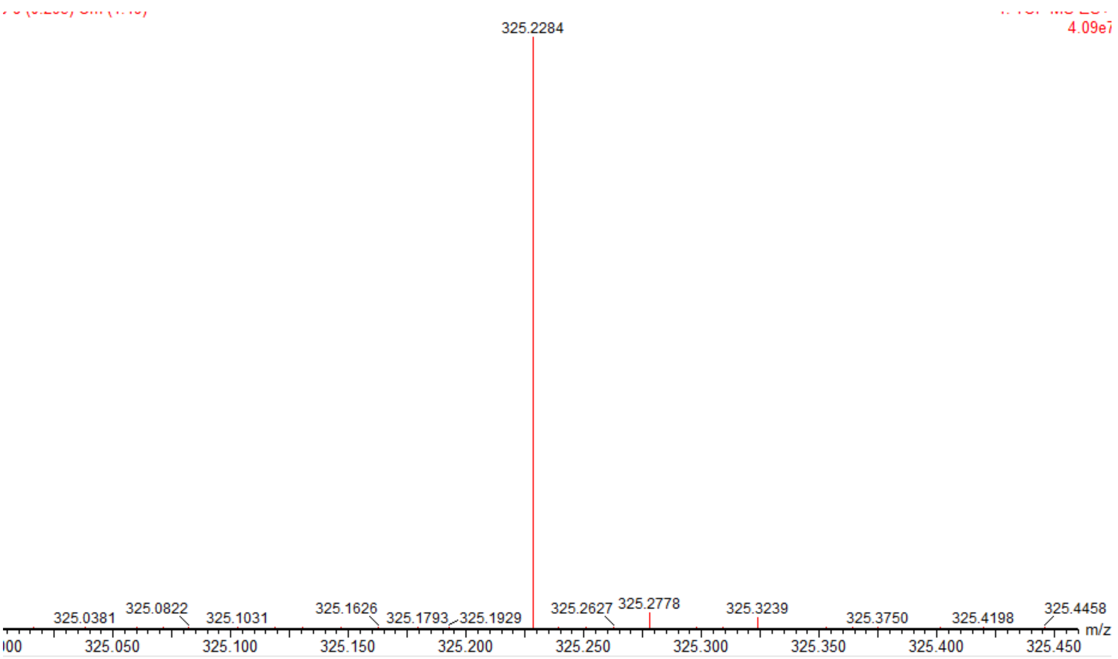

Supplementary Figure 89. HRMS spectra of **4ab**

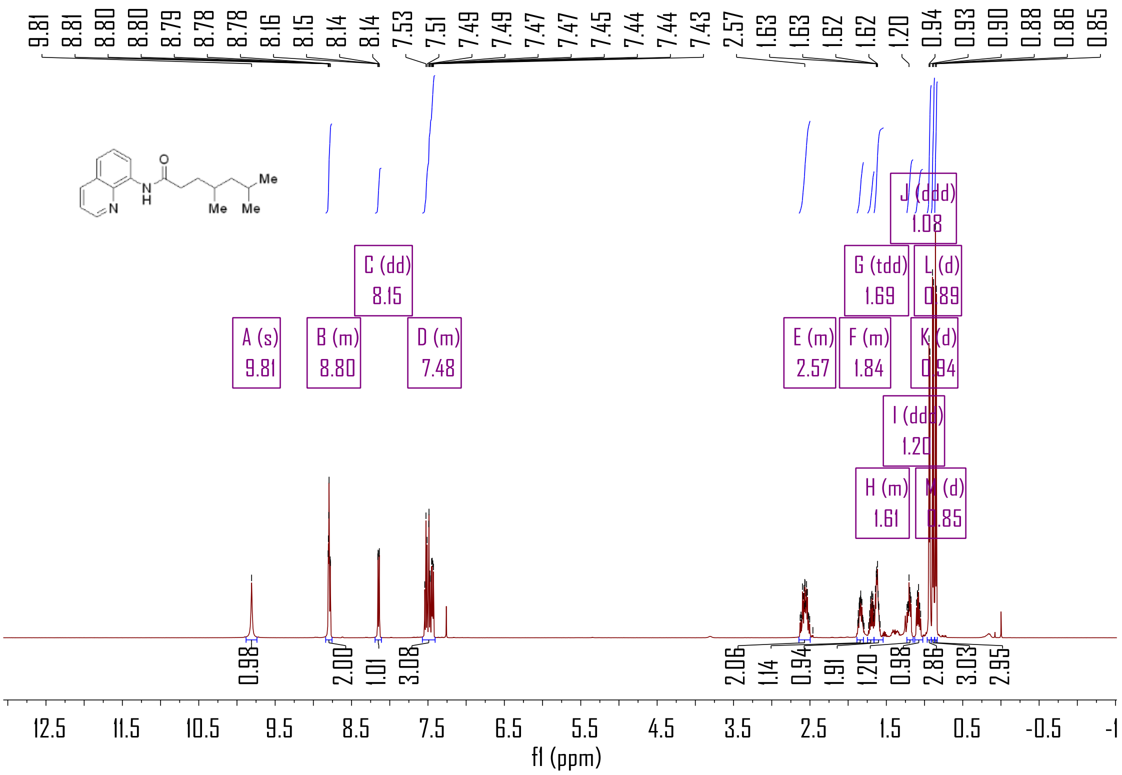

Supplementary Figure 90. ^{1}H NMR spectra of **4ac**

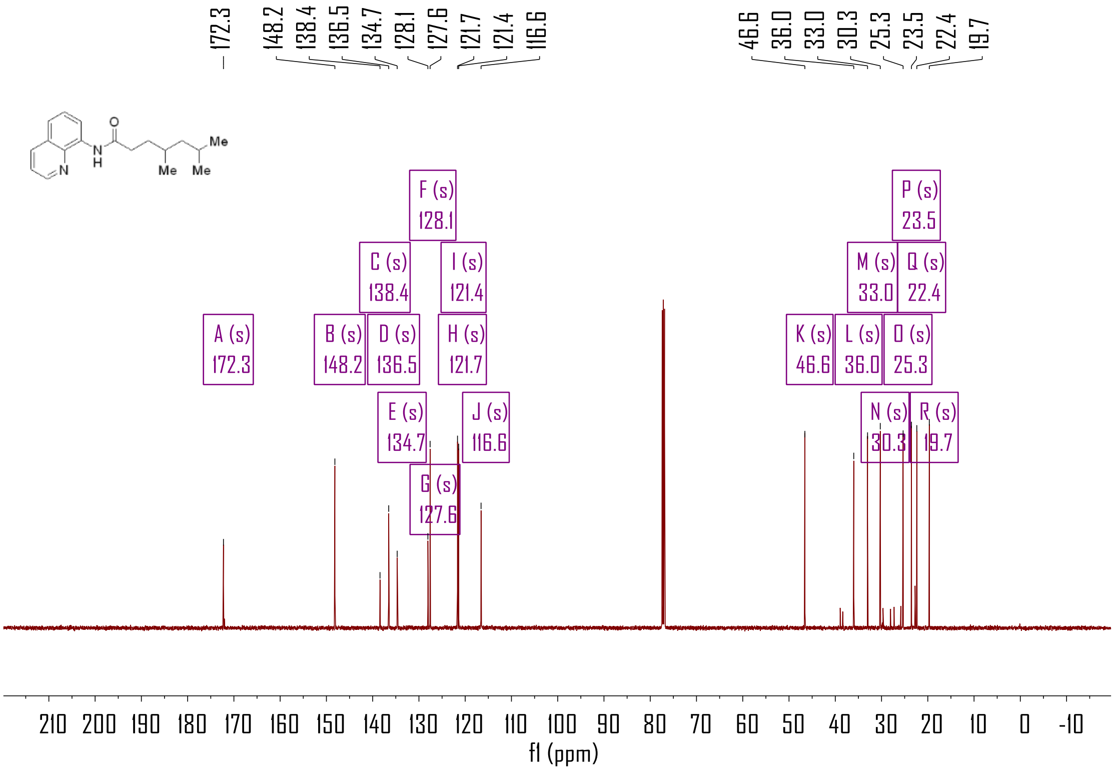

Supplementary Figure 91. ^{13}C NMR spectra of **4ac**

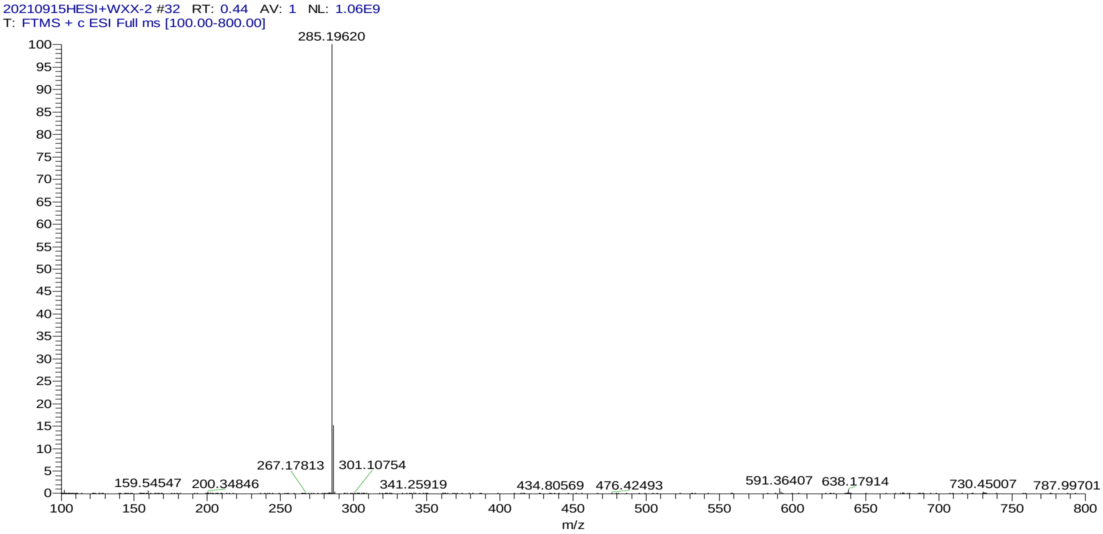

Supplementary Figure 92. HRMS spectra of **4ac**

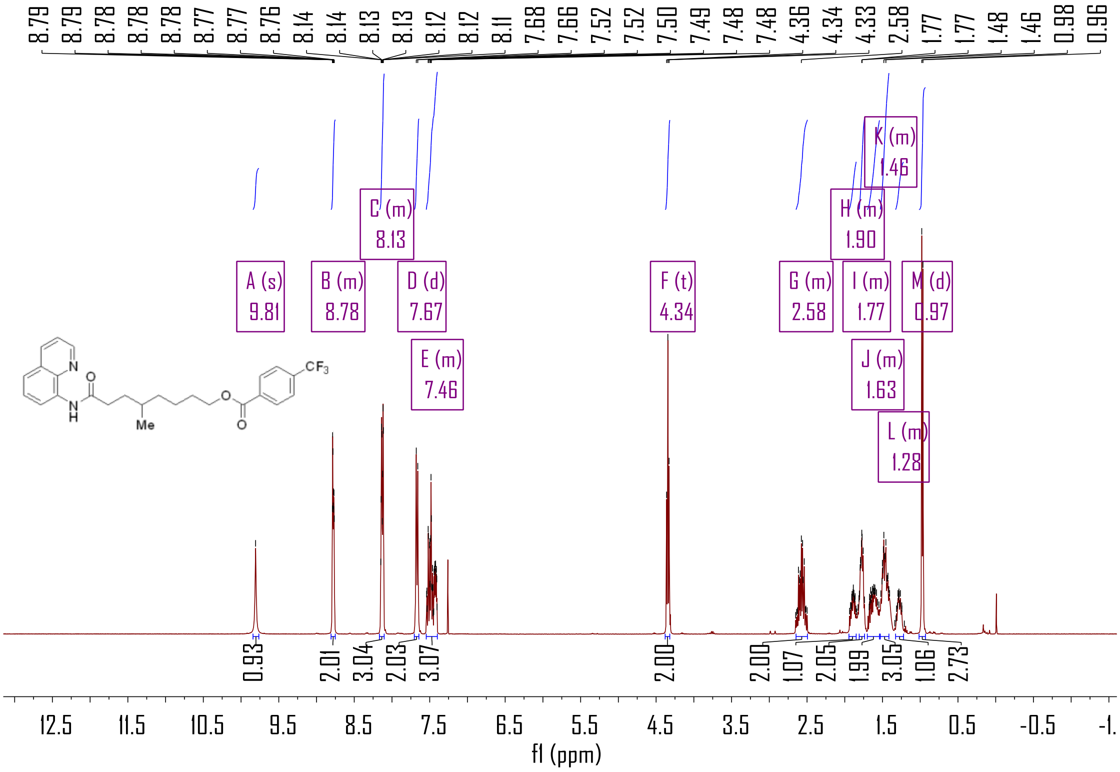

Supplementary Figure 93. ^{1}H NMR spectra of **4ad**

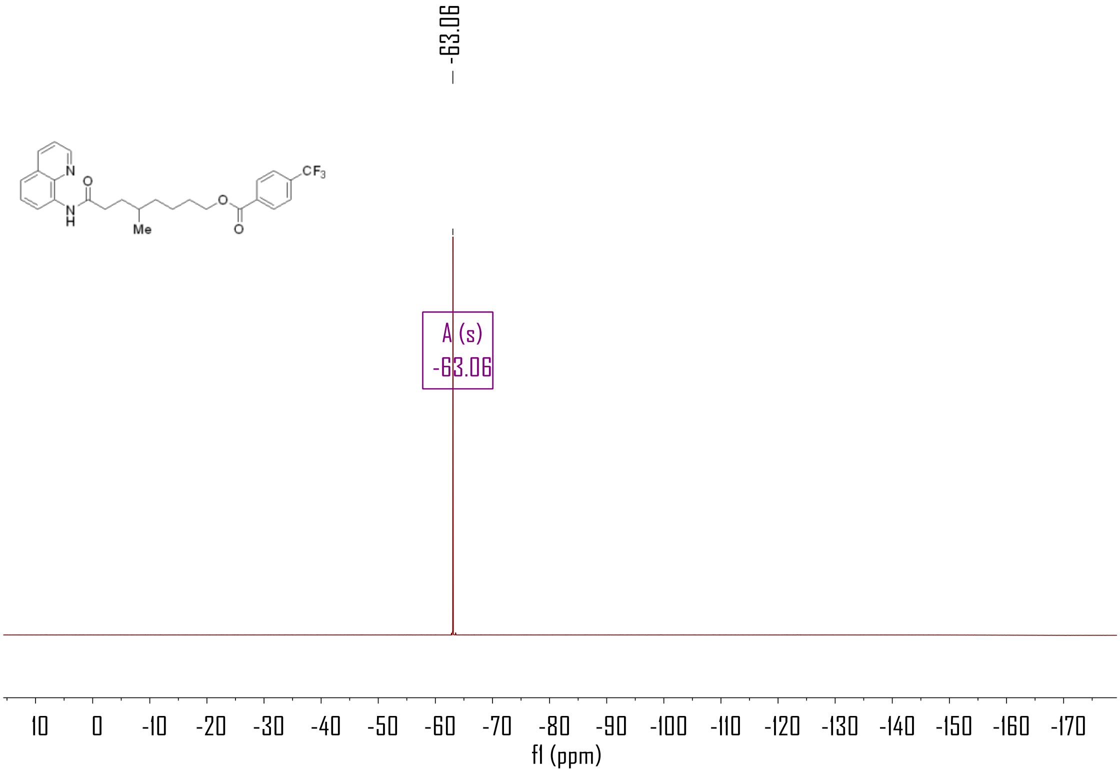

Supplementary Figure 94. ^{19}F NMR spectra of **4ad**

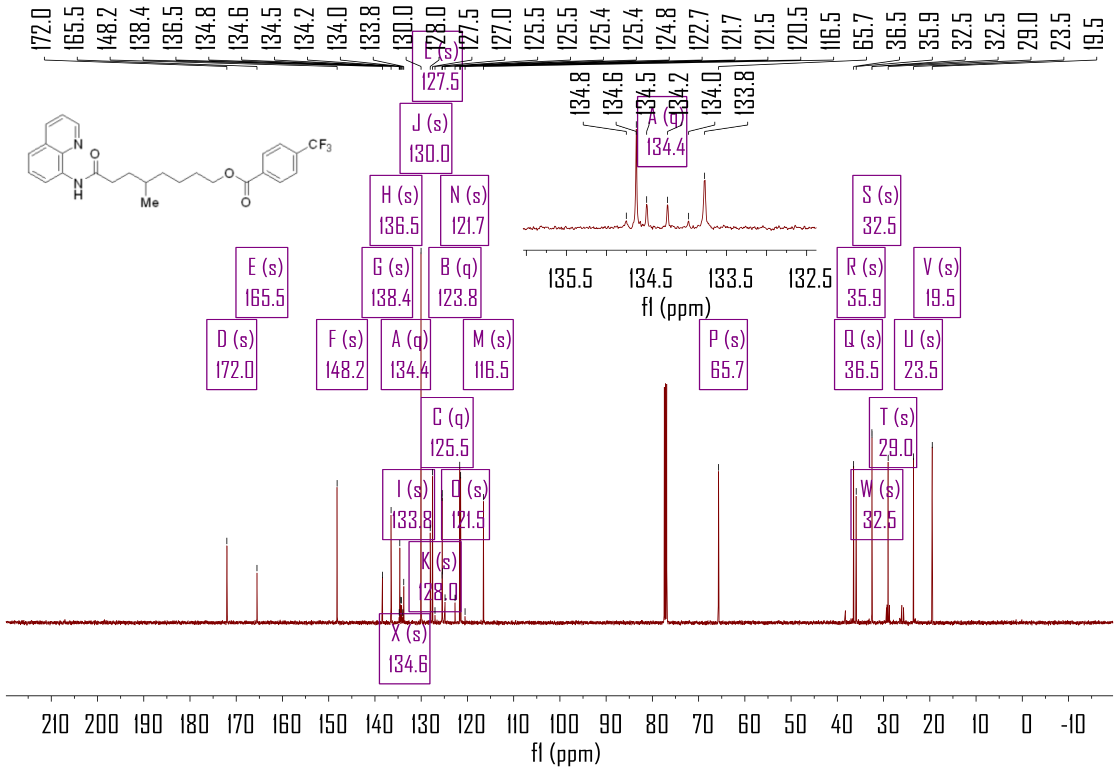

Supplementary Figure 95. ^{13}C NMR spectra of **4ad**

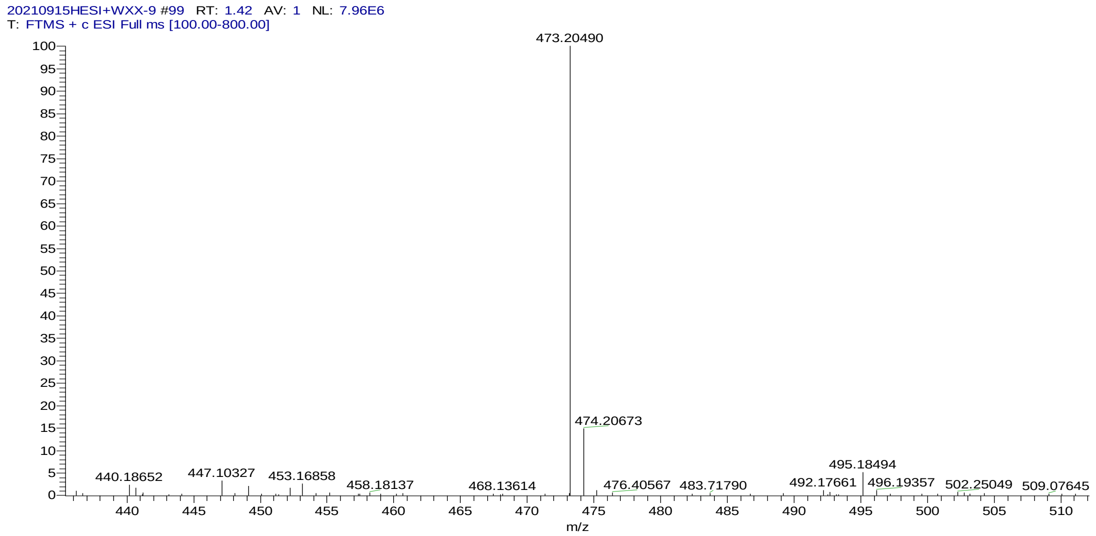

Supplementary Figure 96. HRMS spectra of **4ad**

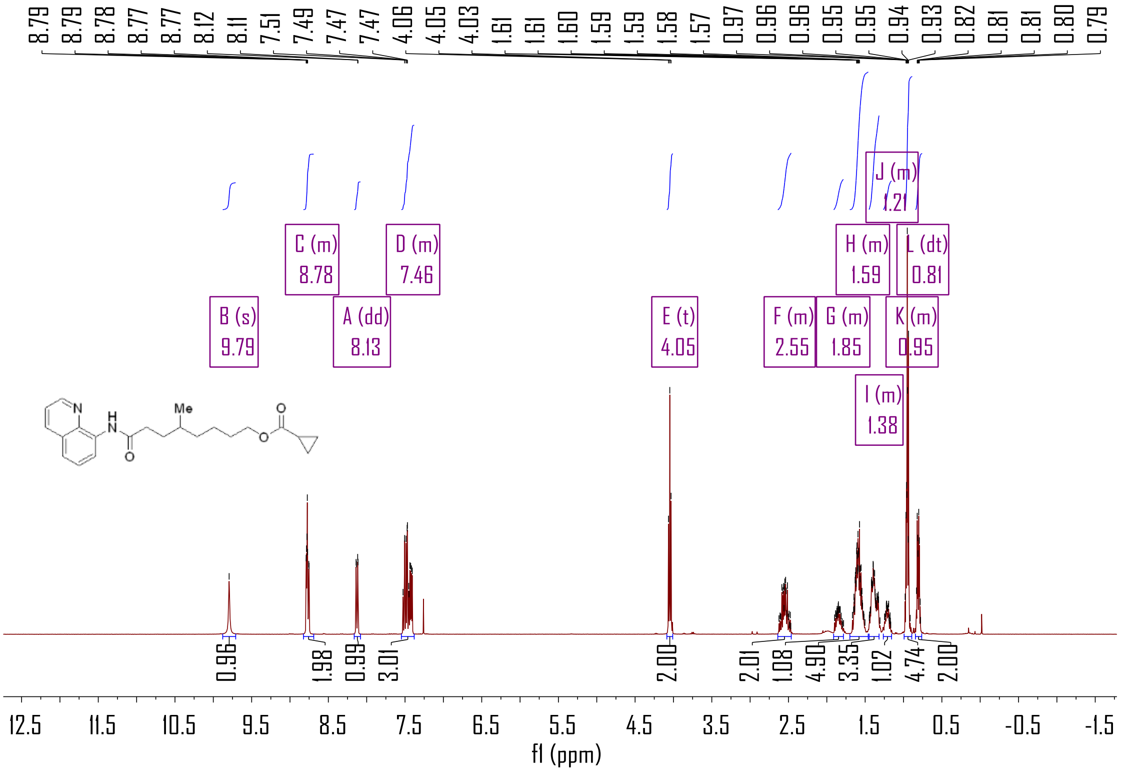

Supplementary Figure 97. ^{1}H NMR spectra of **4ae**

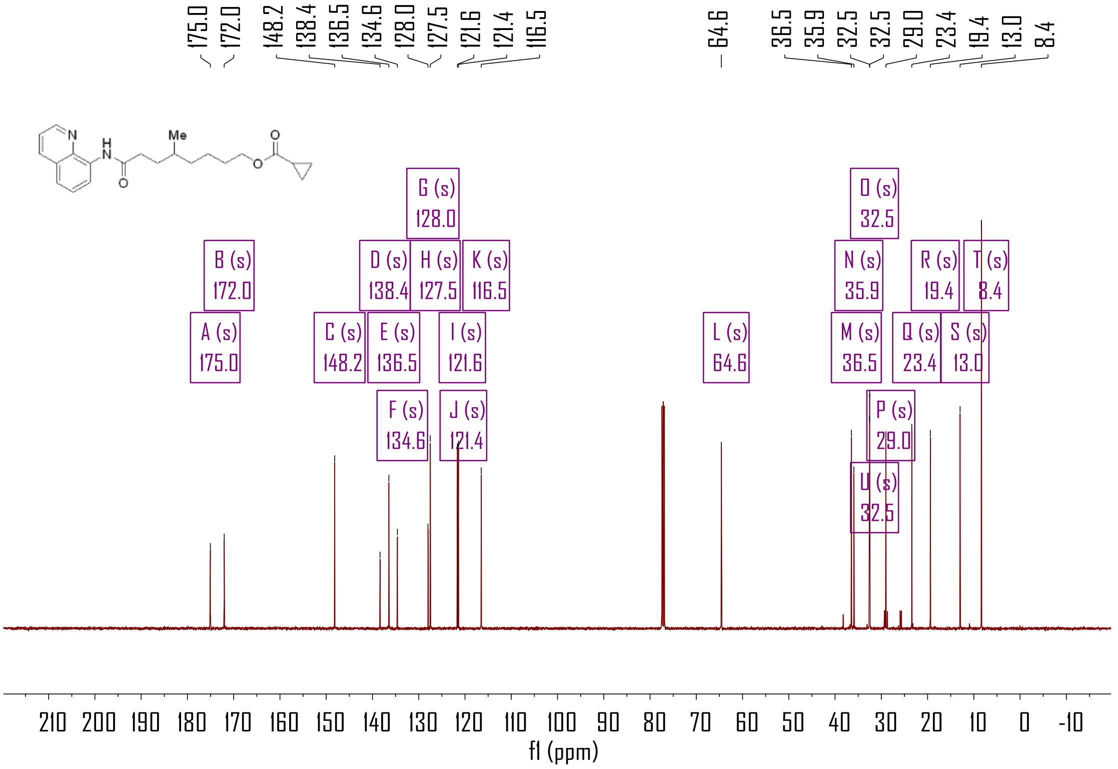

Supplementary Figure 98. ^{13}C NMR spectra of **4ae**

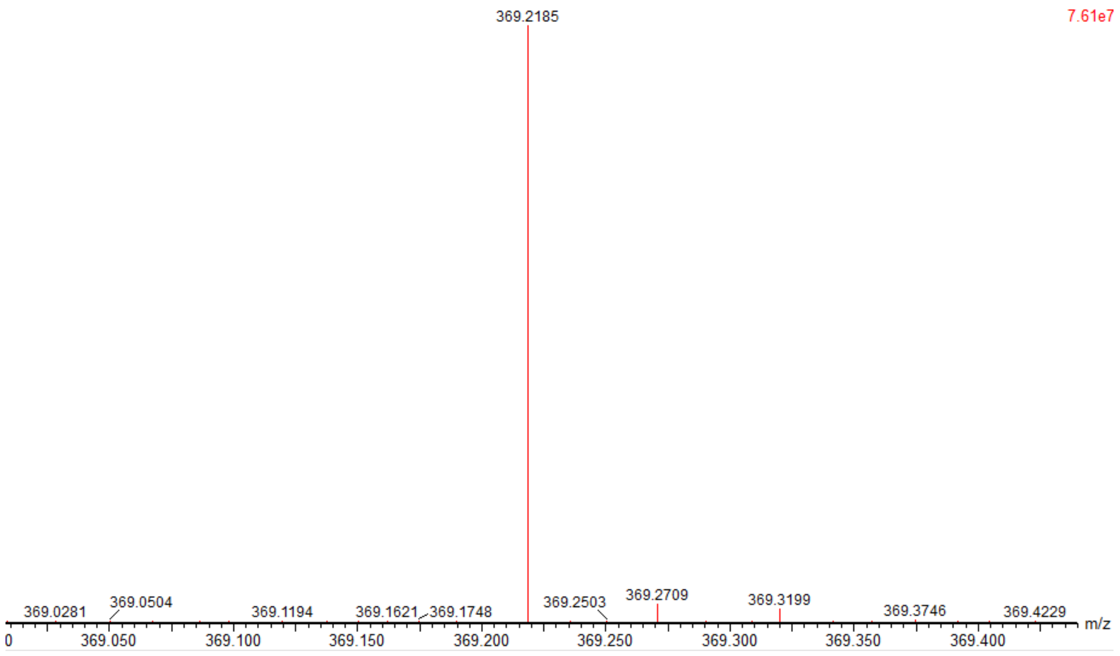

Supplementary Figure 99. HRMS spectra of **4ae**

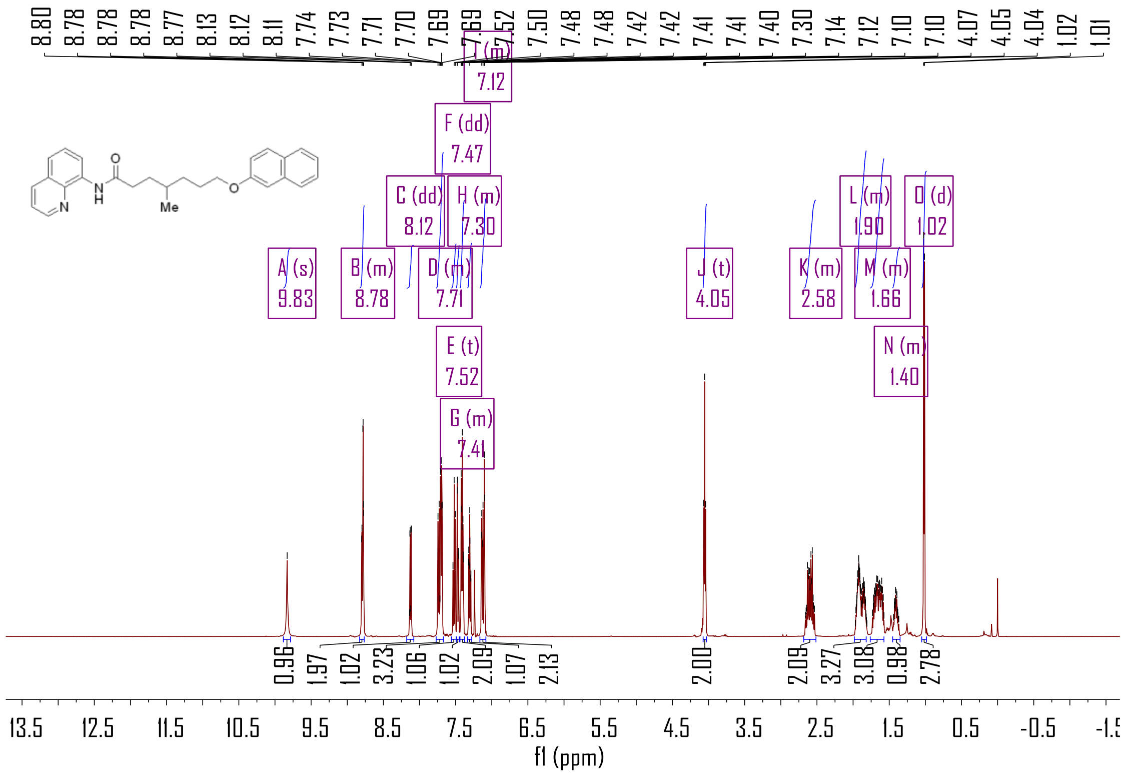

Supplementary Figure 100. ^{1}H NMR spectra of **4af**

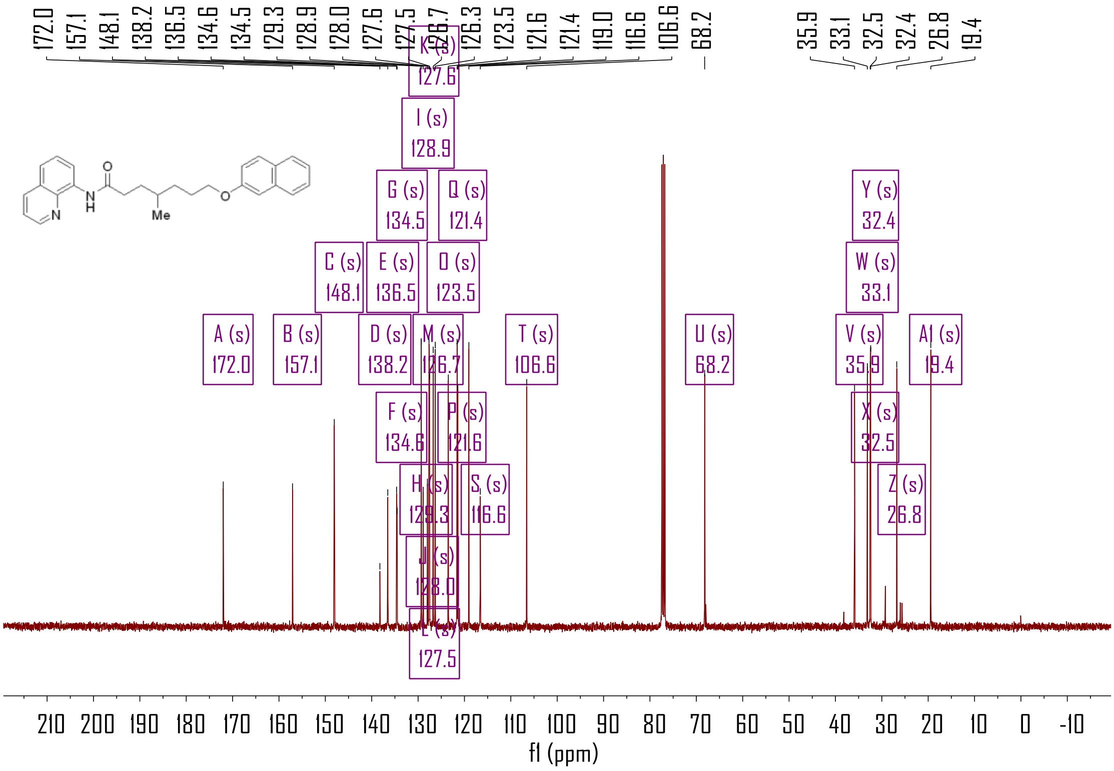

Supplementary Figure 101. ^{13}C NMR spectra of **4af**

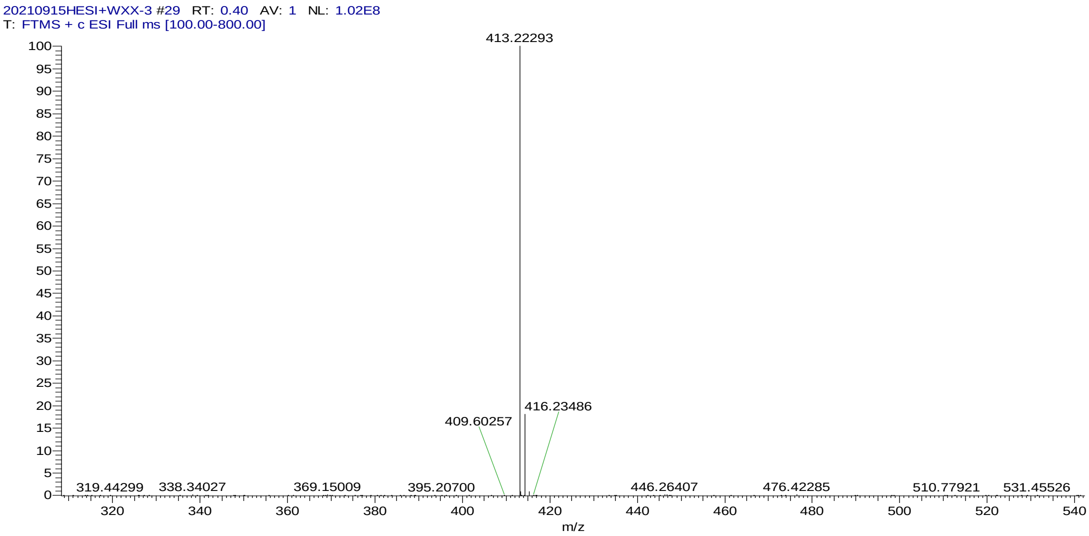

Supplementary Figure 102. HRMS spectra of **4af**

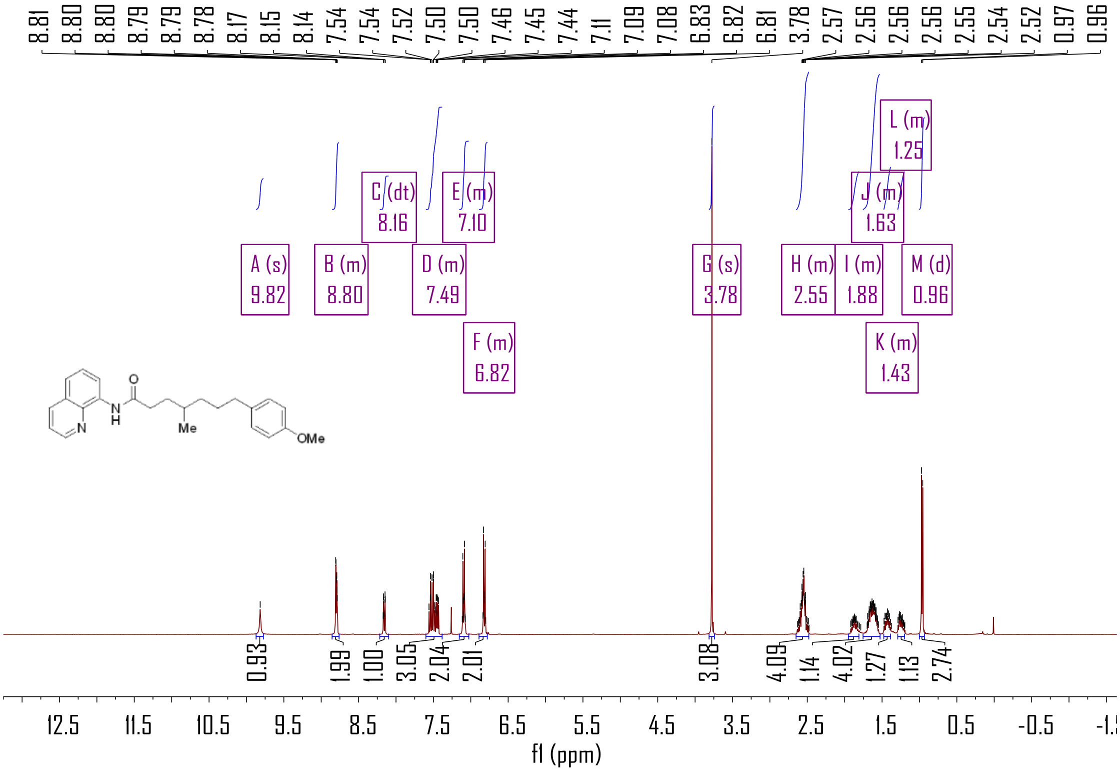

Supplementary Figure 103. ^{1}H NMR spectra of **4ag**

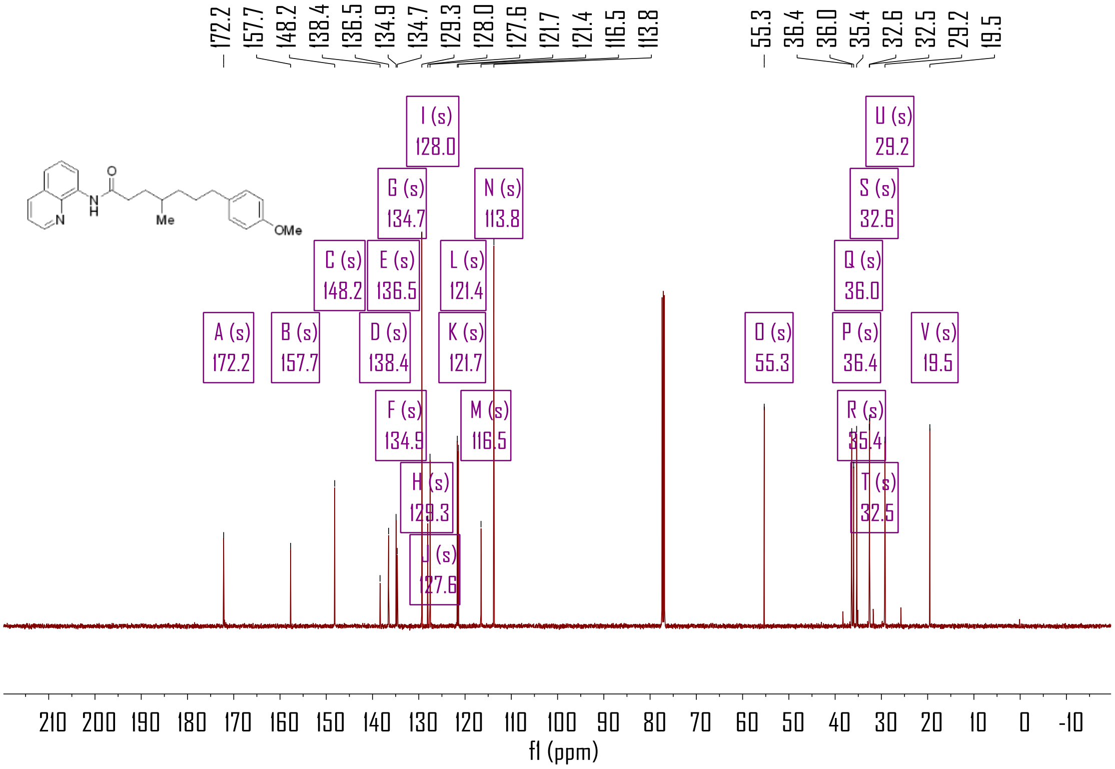

Supplementary Figure 104. ^{13}C NMR spectra of **4ag**

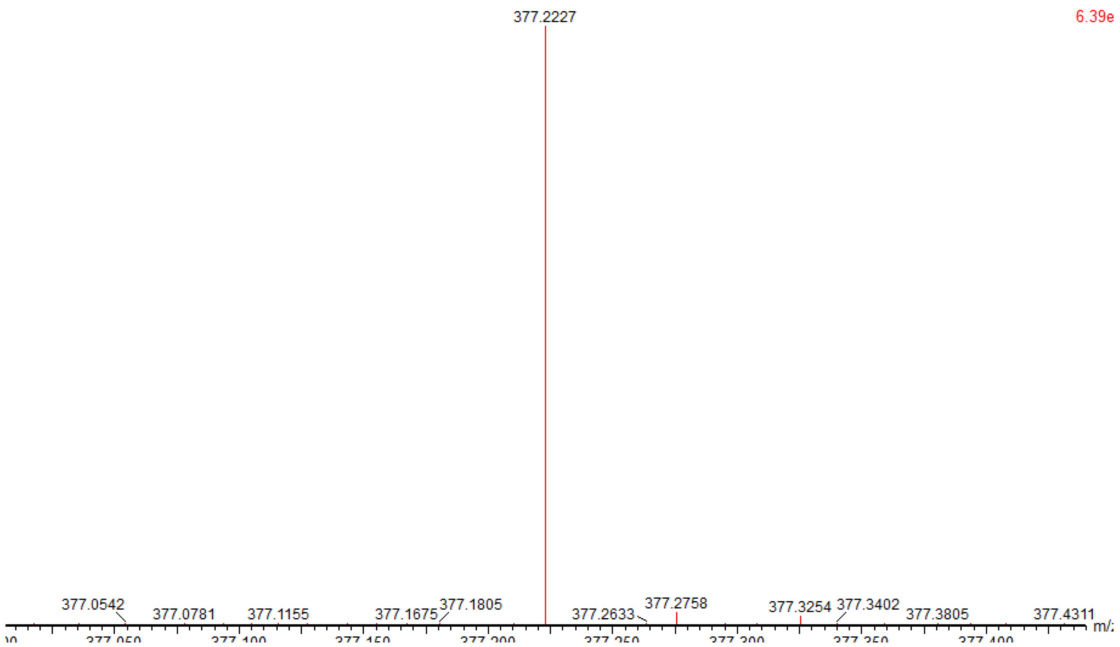

Supplementary Figure 105. HRMS spectra of **4ag**

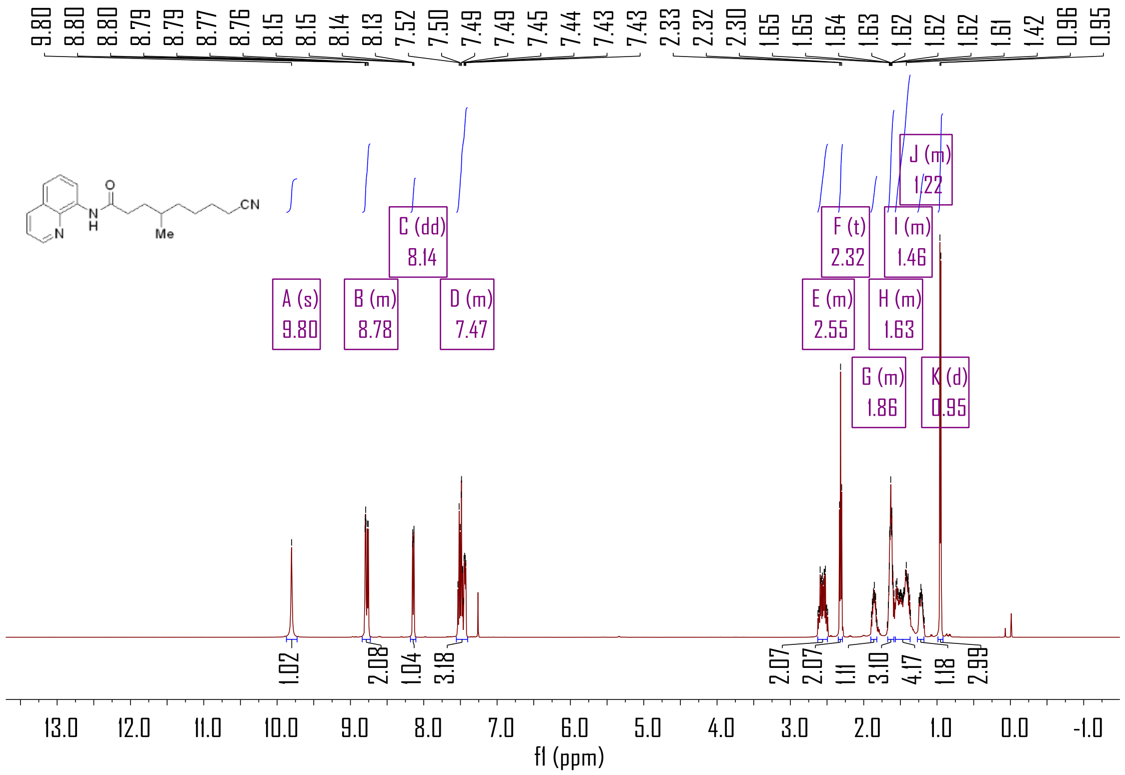

Supplementary Figure 106. ^{1}H NMR spectra of **4ah**

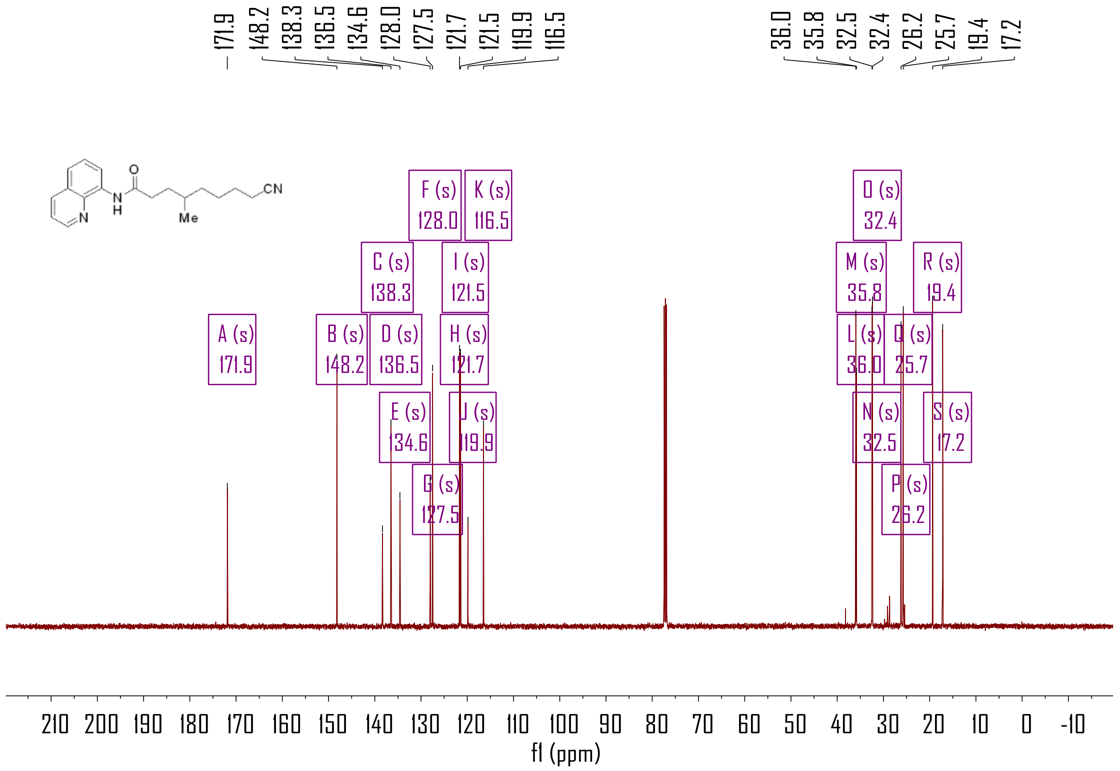

Supplementary Figure 107. ^{13}C NMR spectra of **4ah**

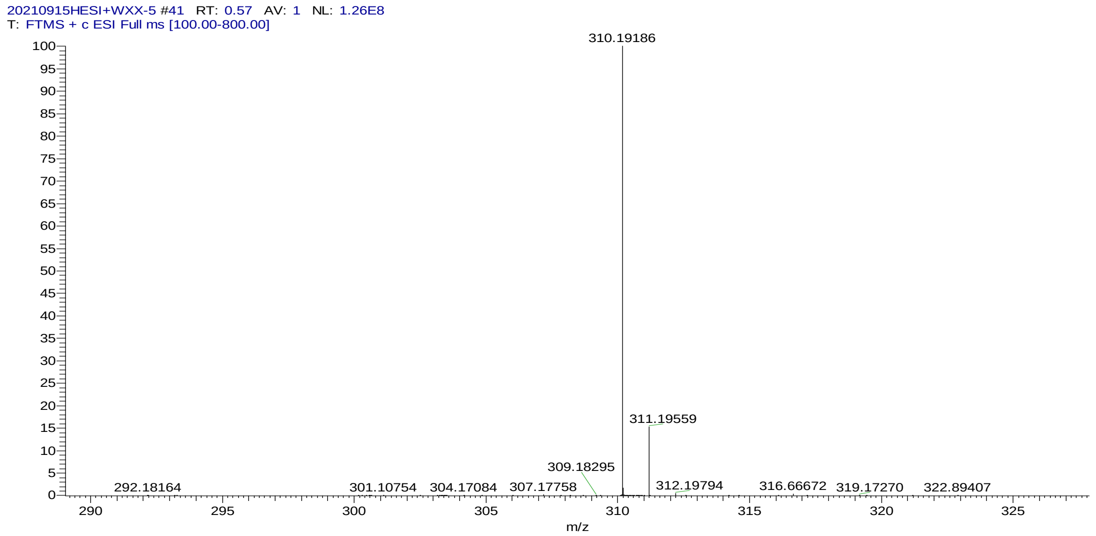

Supplementary Figure 108. HRMS spectra of **4ah**

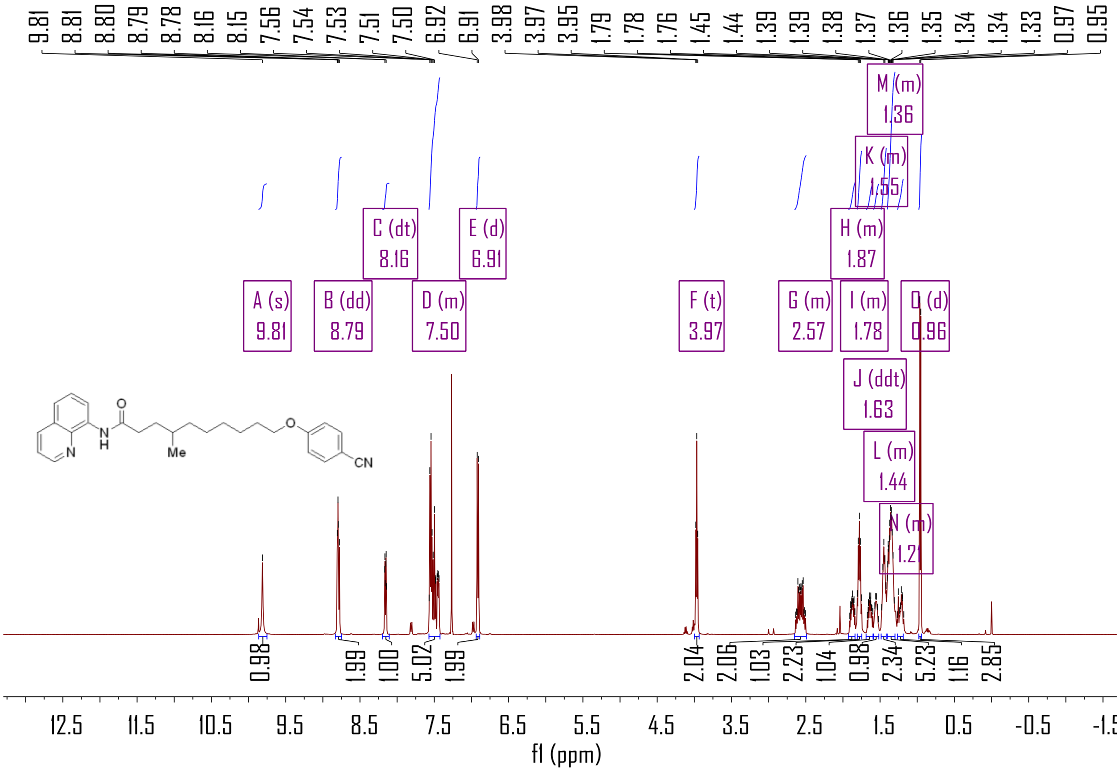

Supplementary Figure 109. ^{1}H NMR spectra of **4ai**

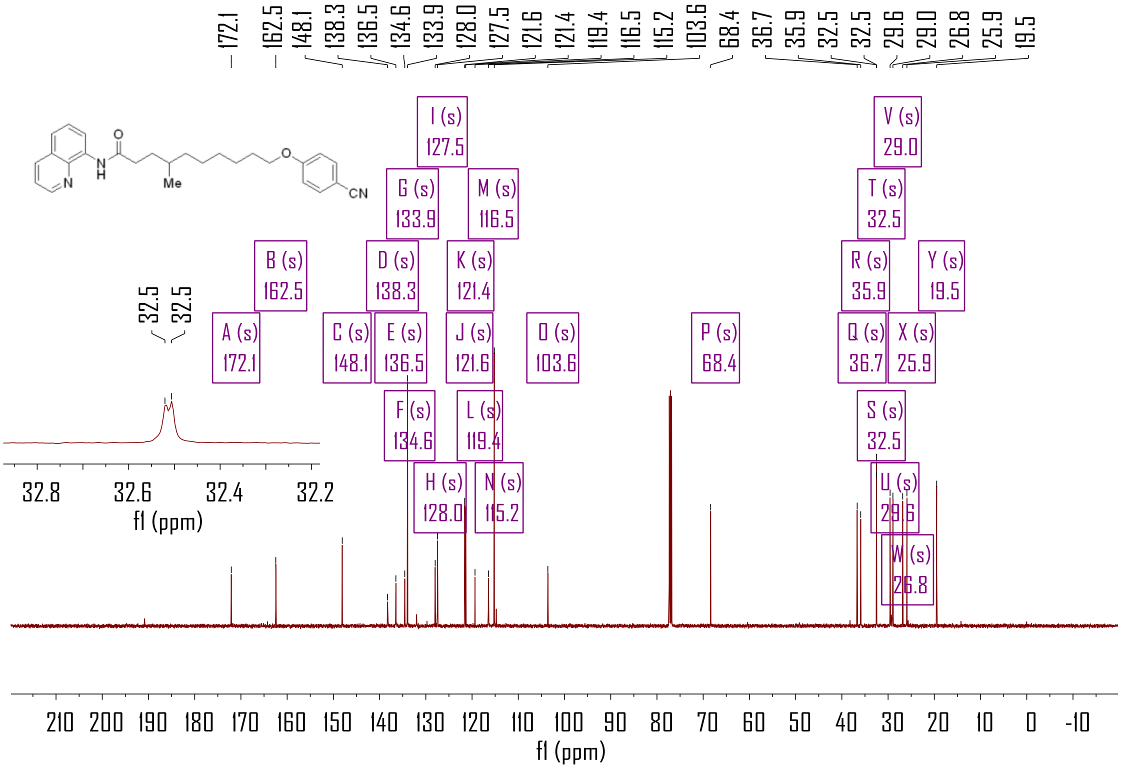

Supplementary Figure 110. ^{13}C NMR spectra of **4ai**

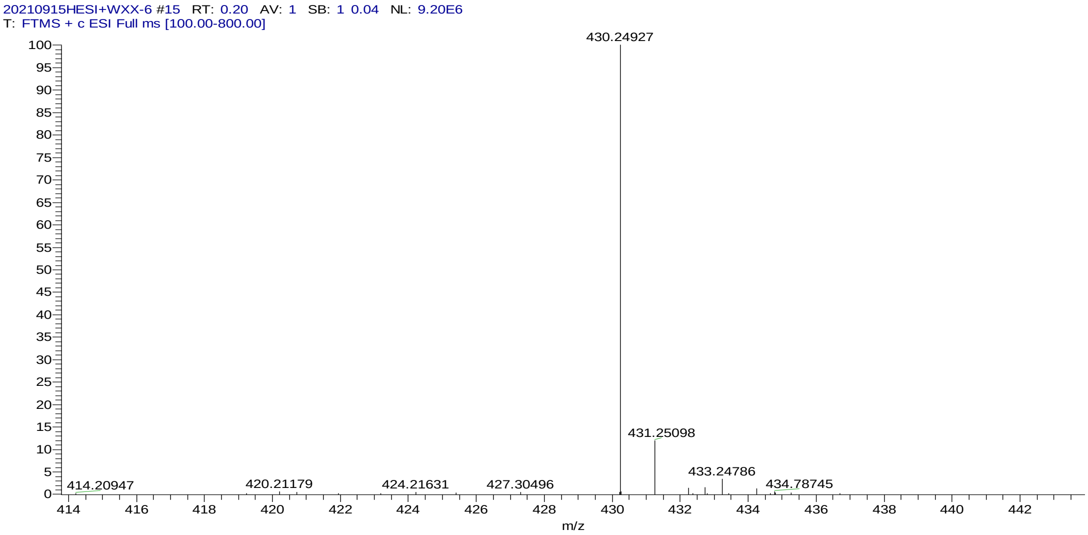

Supplementary Figure 111. HRMS spectra of **4ai**

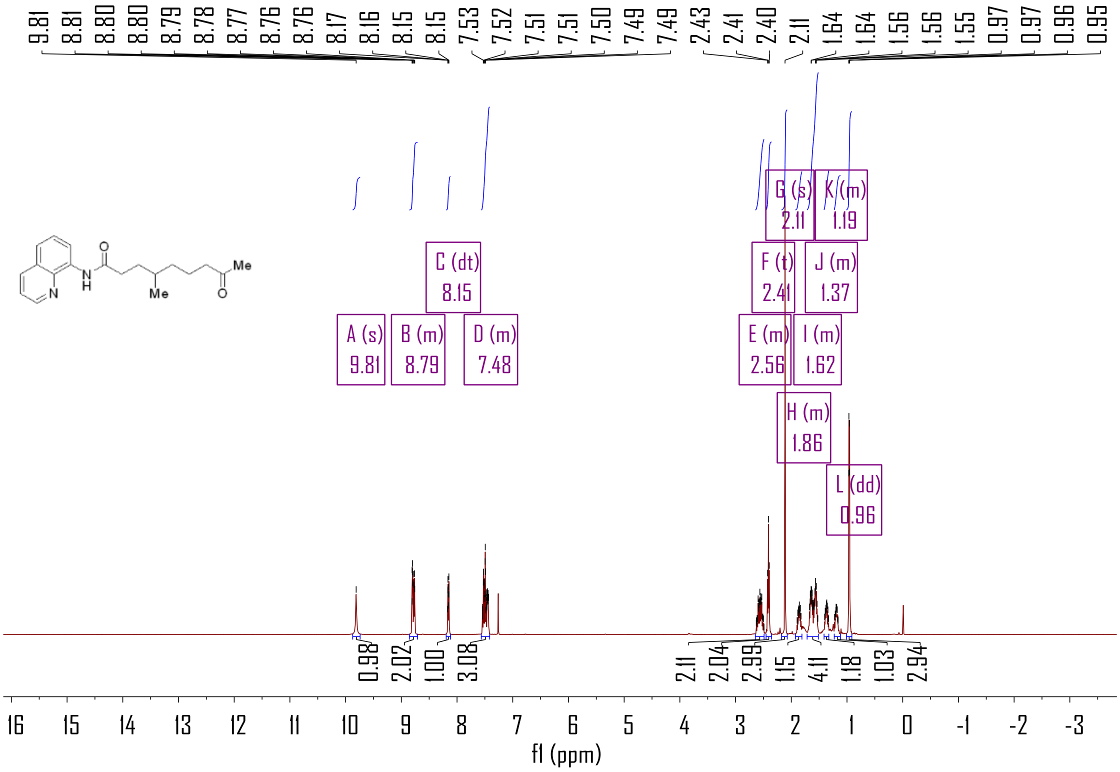

Supplementary Figure 112. ^{1}H NMR spectra of **4aj**

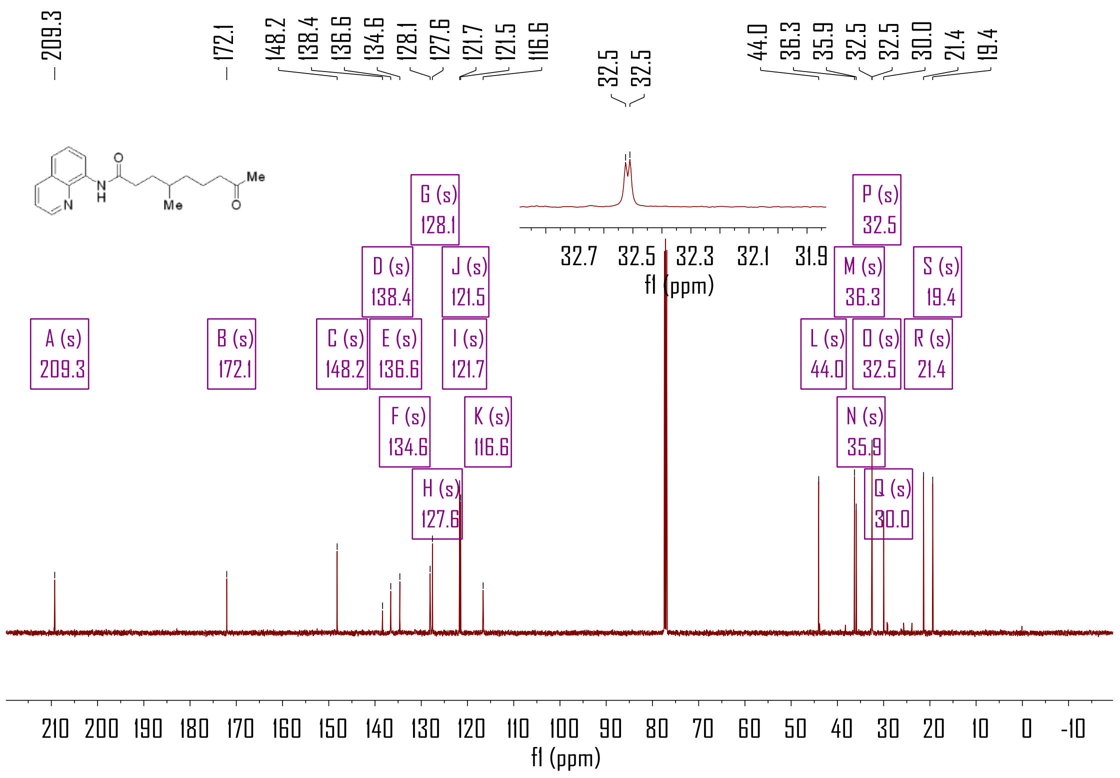

Supplementary Figure 113. ^{13}C NMR spectra of **4aj**

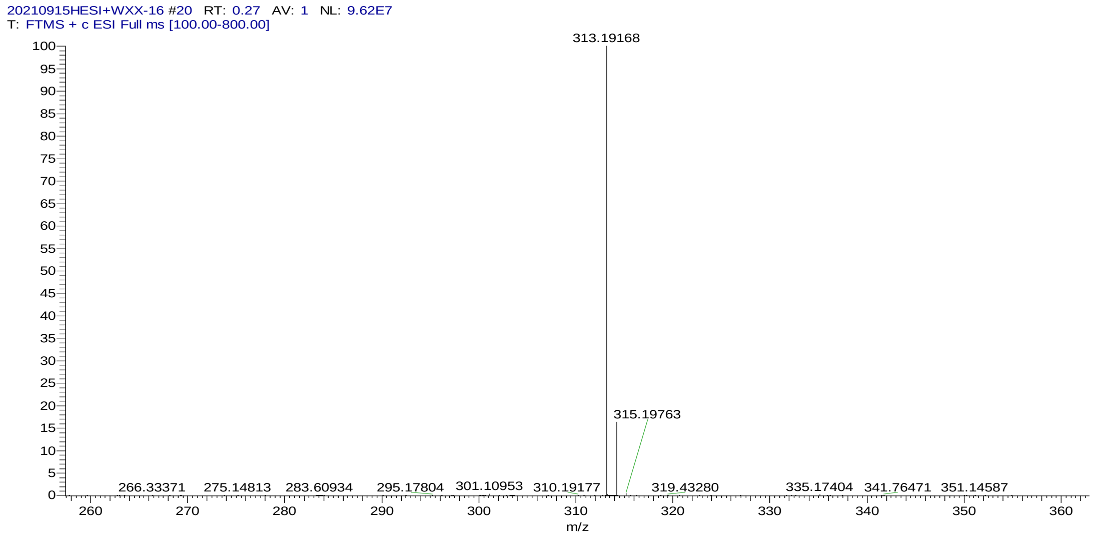

Supplementary Figure 114. HRMS spectra of **4aj**

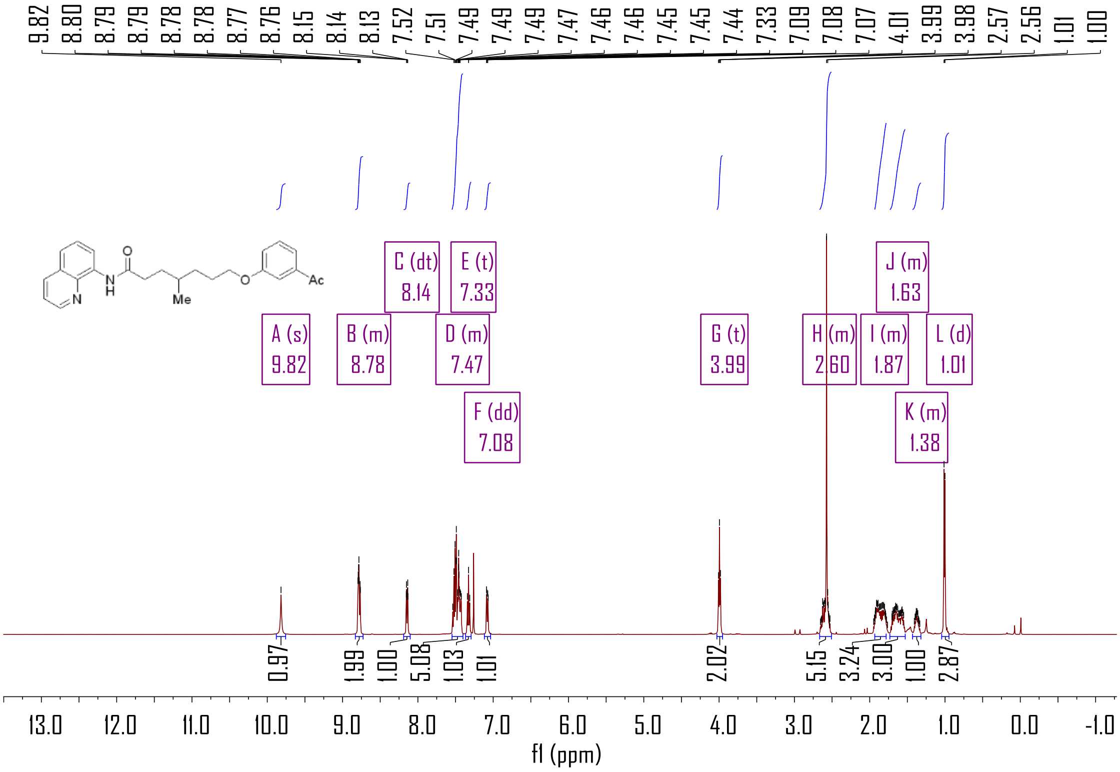

Supplementary Figure 115. ^{1}H NMR spectra of **4ak**

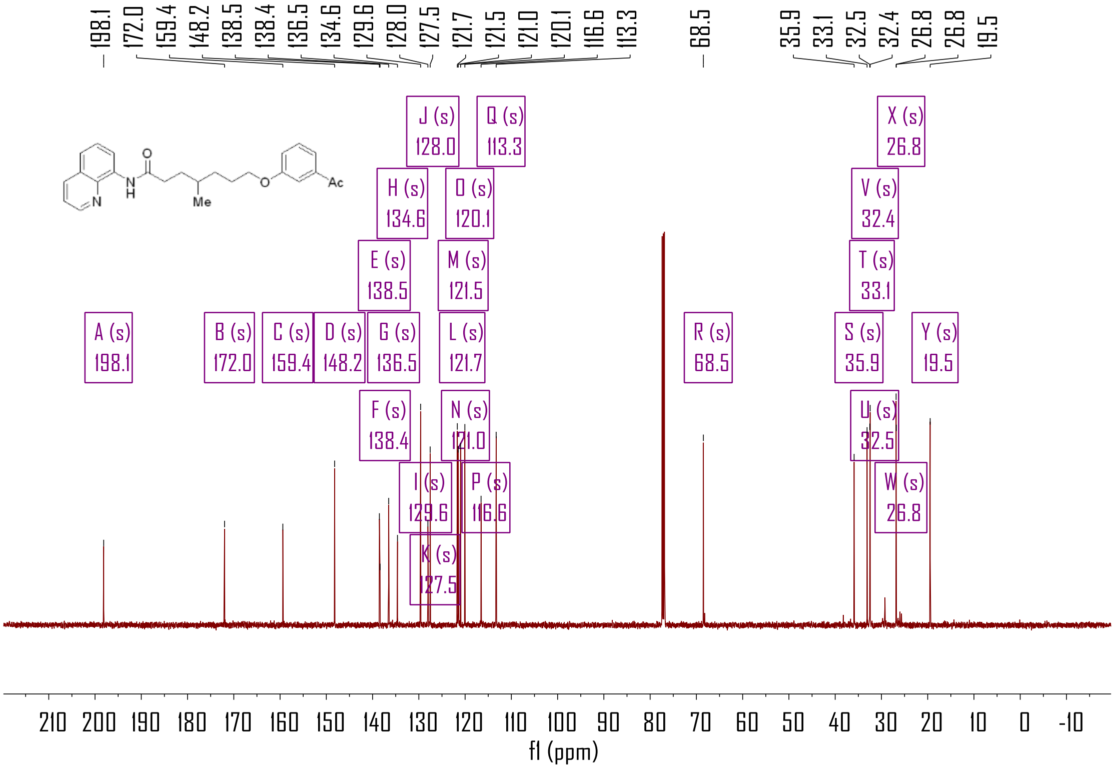

Supplementary Figure 116. ^{13}C NMR spectra of **4ak**

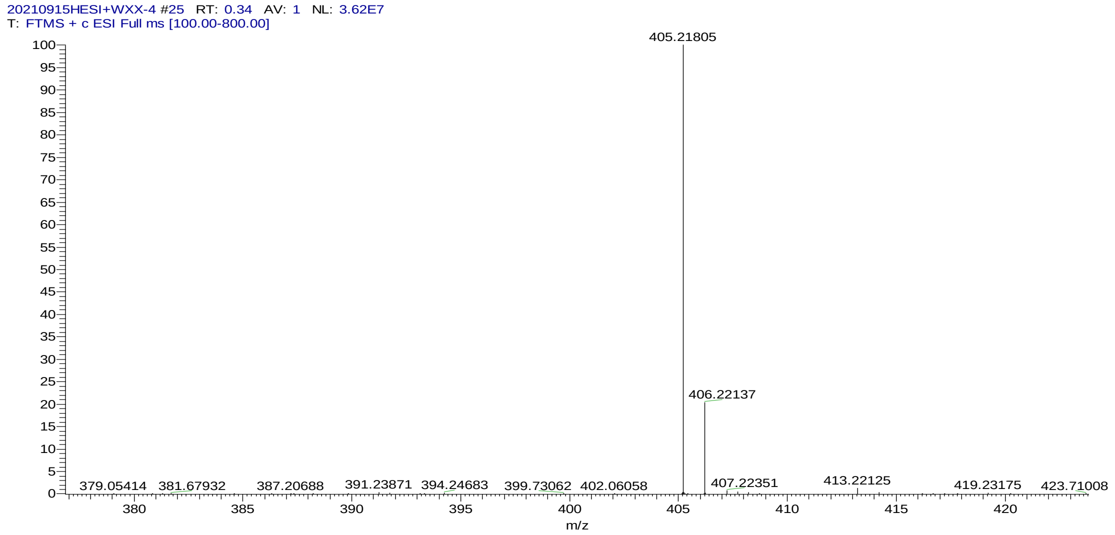

Supplementary Figure 117. HRMS spectra of **4ak**

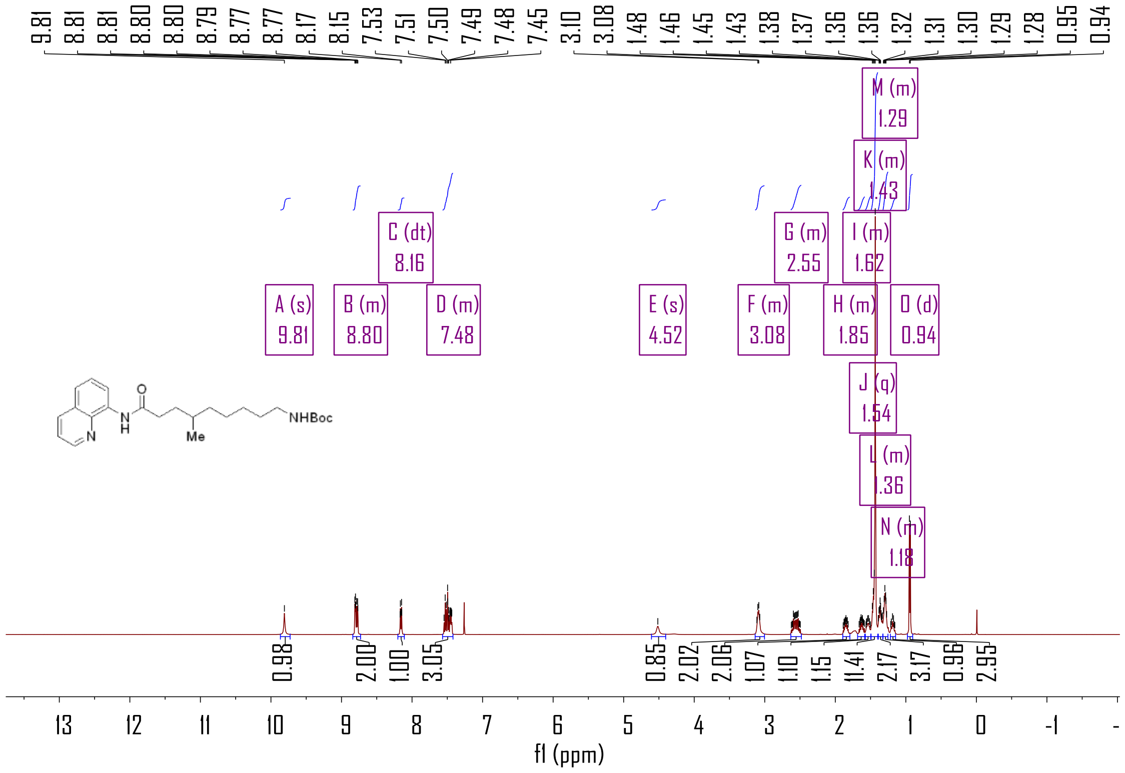

Supplementary Figure 118. ^{1}H NMR spectra of **4al**

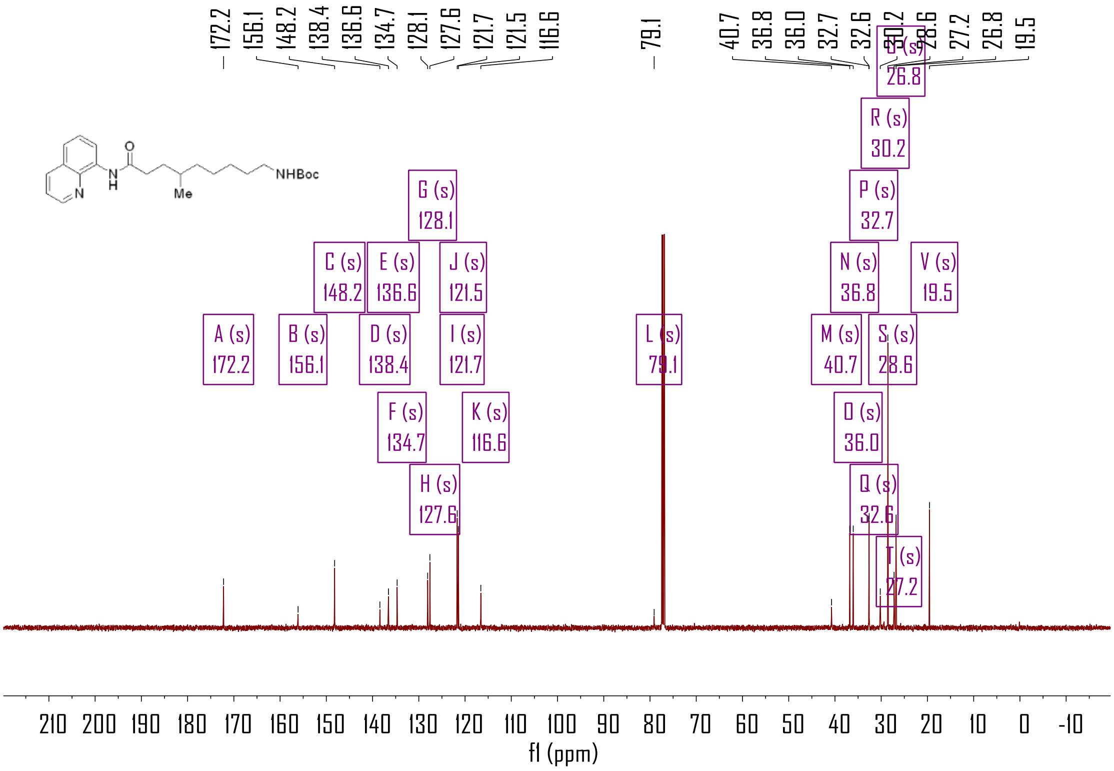

Supplementary Figure 119. ^{13}C NMR spectra of **4al**

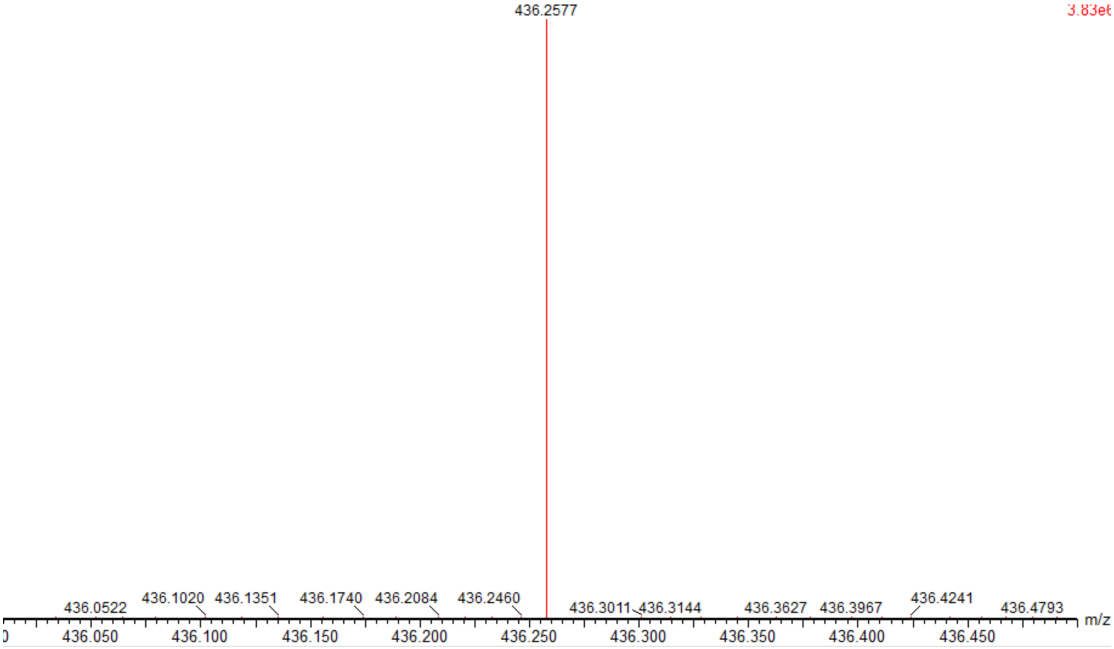

Supplementary Figure 120. HRMS spectra of **4al**

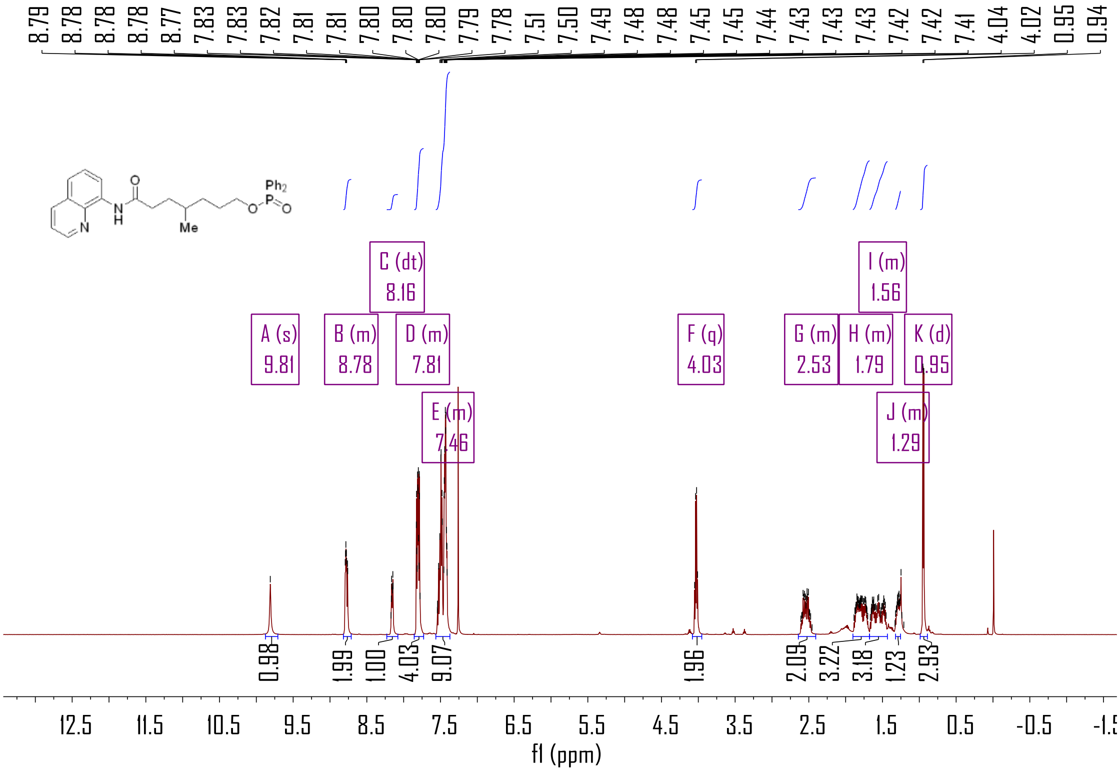

Supplementary Figure 121. ^{1}H NMR spectra of **4am**

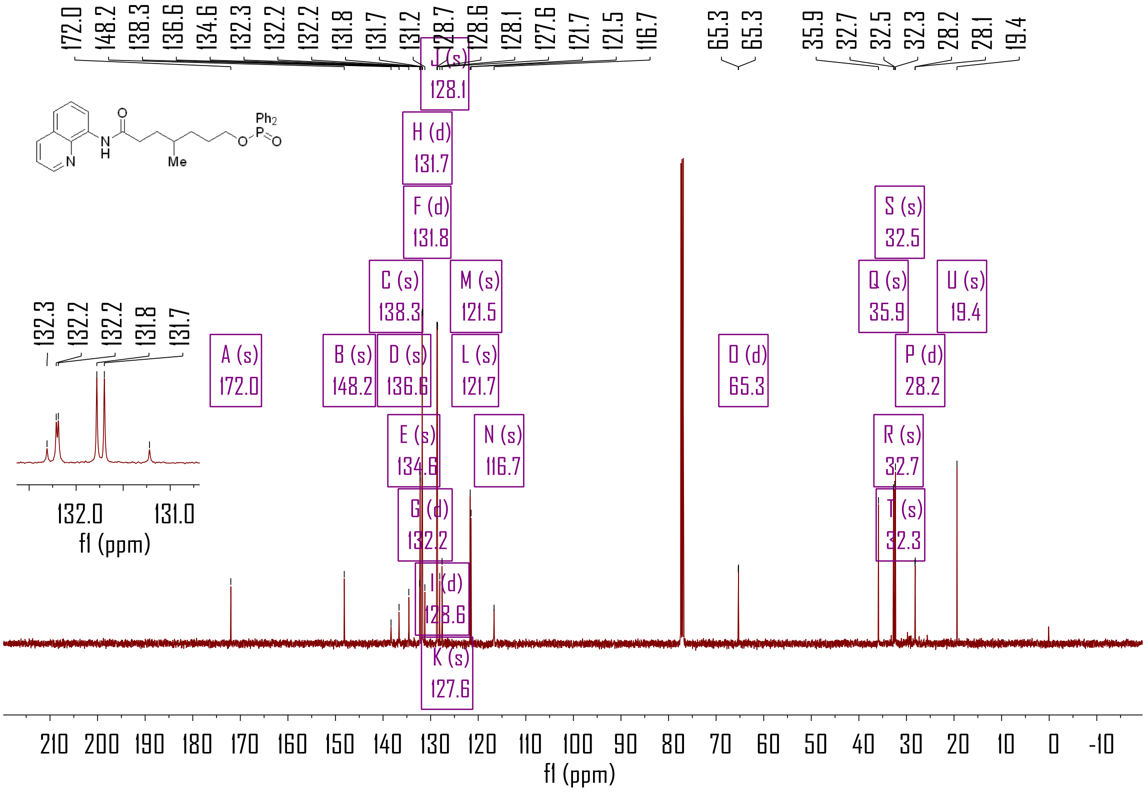

Supplementary Figure 122. ^{13}C NMR spectra of **4am**

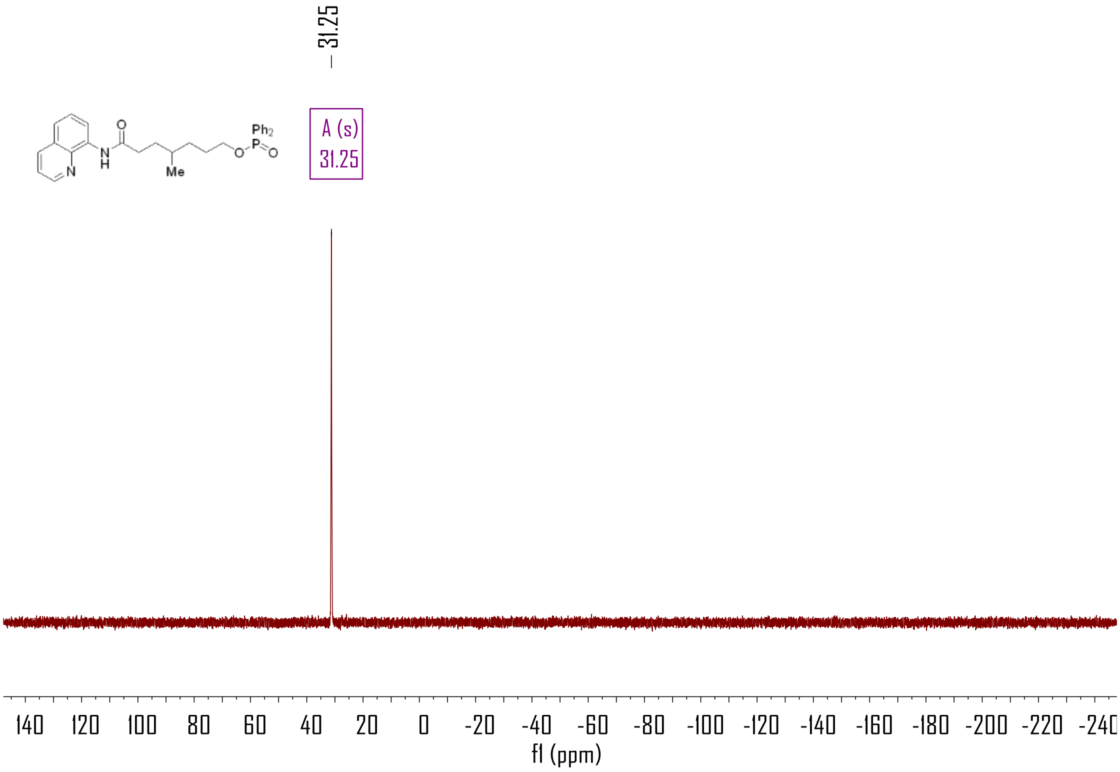

Supplementary Figure 123. ^{31}P NMR spectra of **4am**

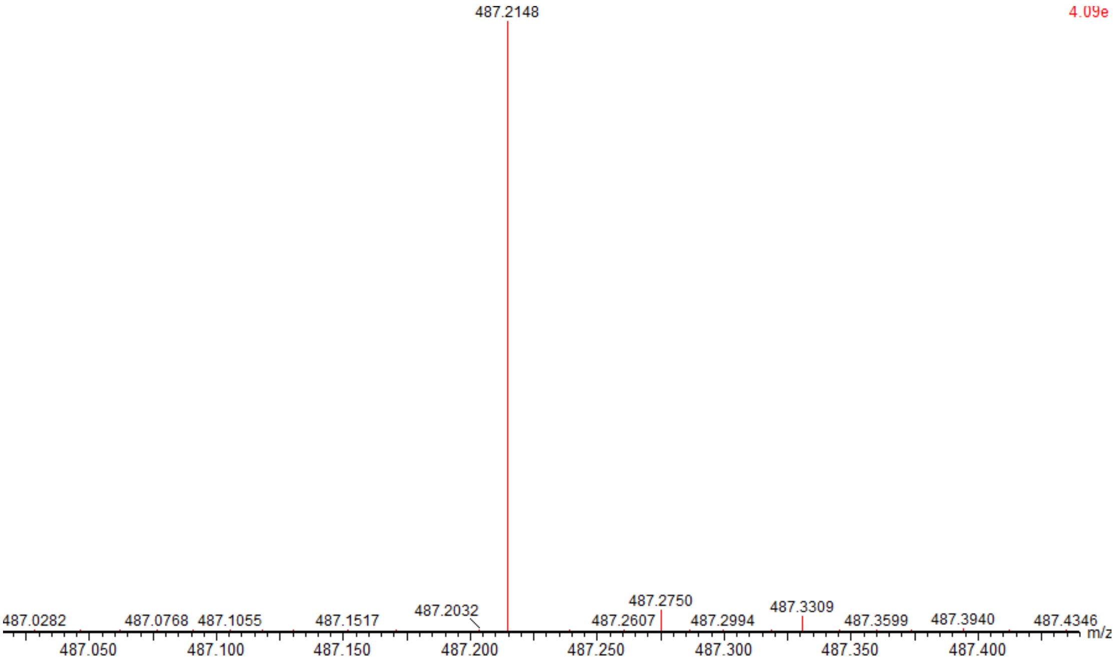

Supplementary Figure 124. HRMS spectra of **4am**

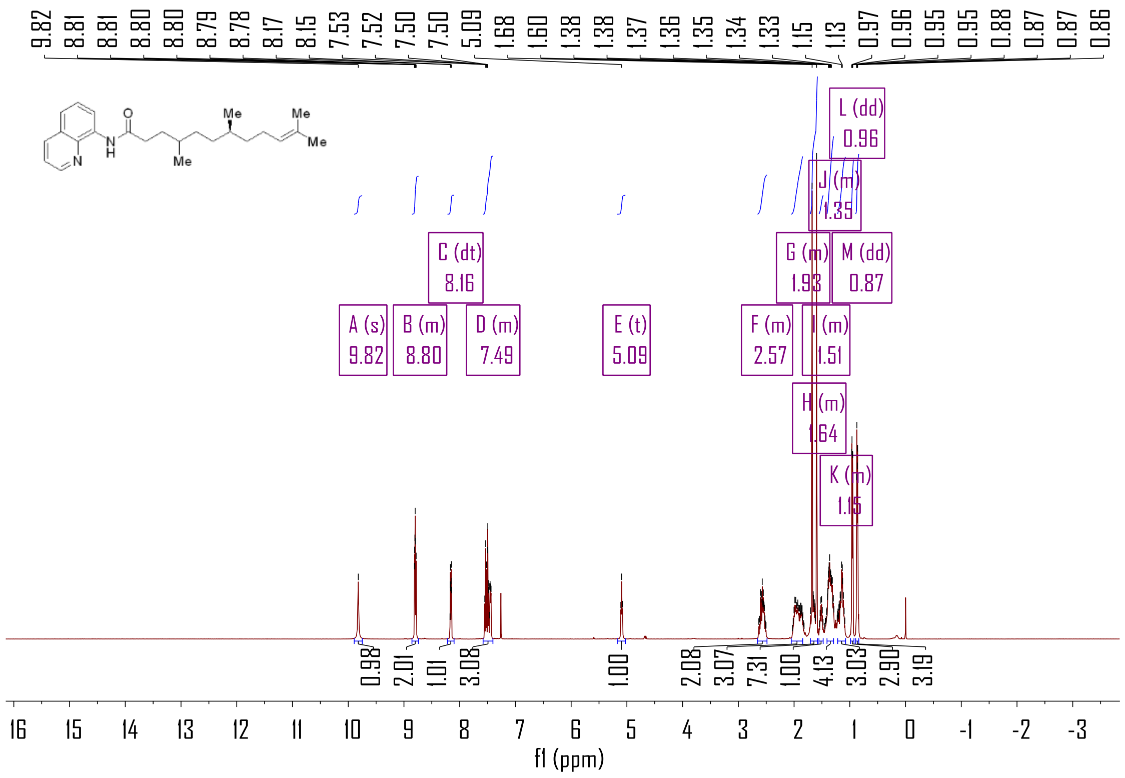

Supplementary Figure 125. ^{1}H NMR spectra of **4an**

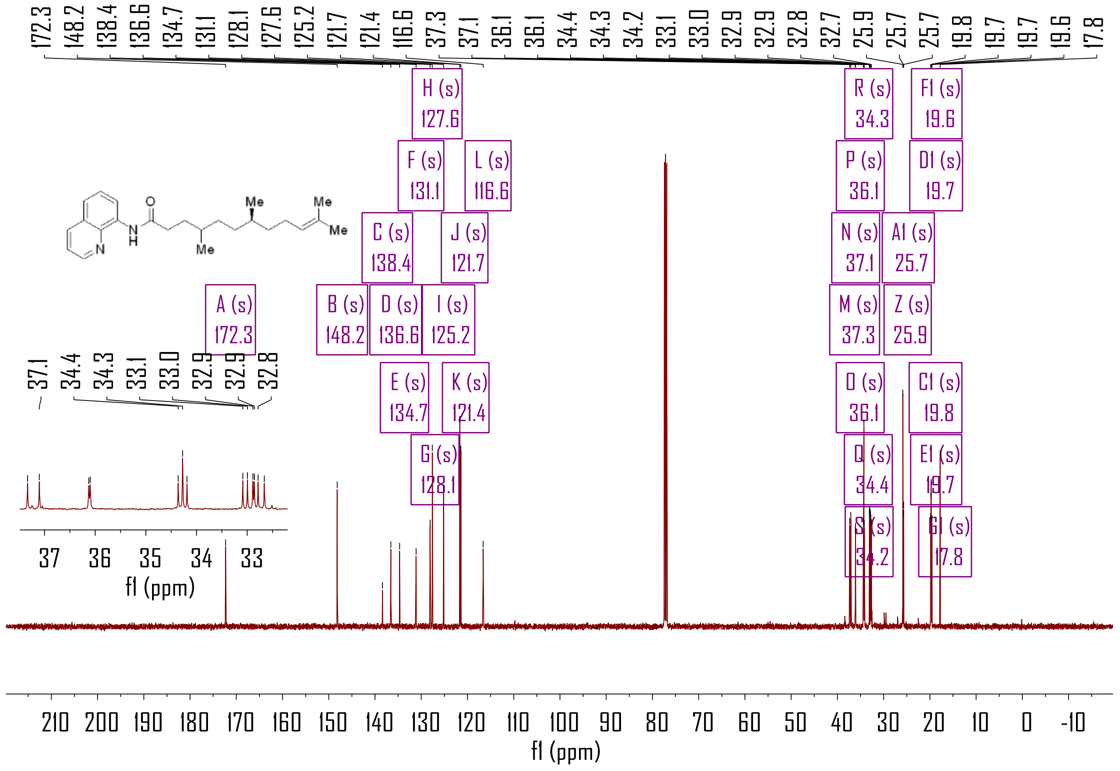

Supplementary Figure 126. ^{13}C NMR spectra of **4an**

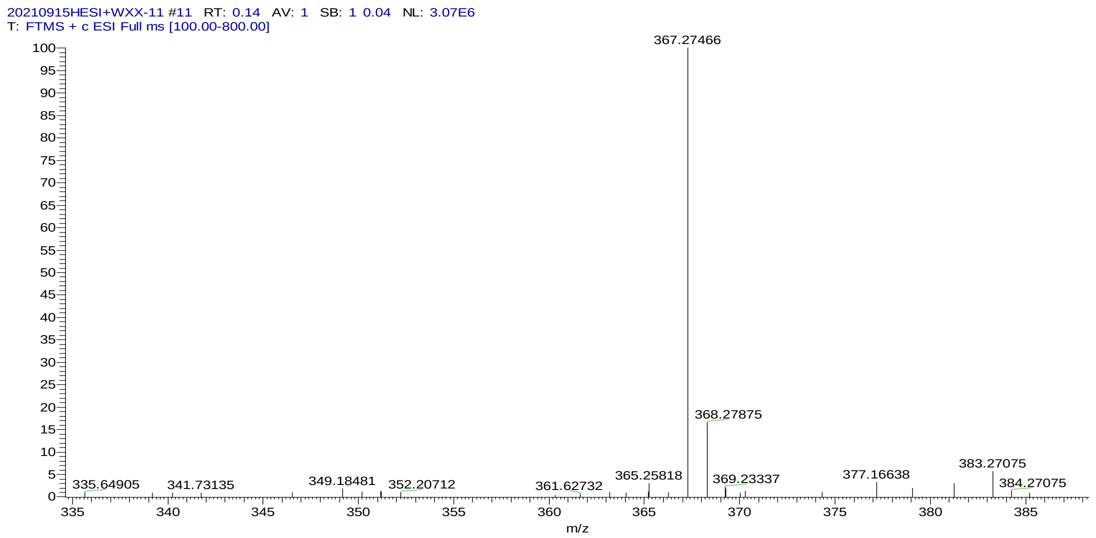

Supplementary Figure 127. HRMS spectra of **4an**

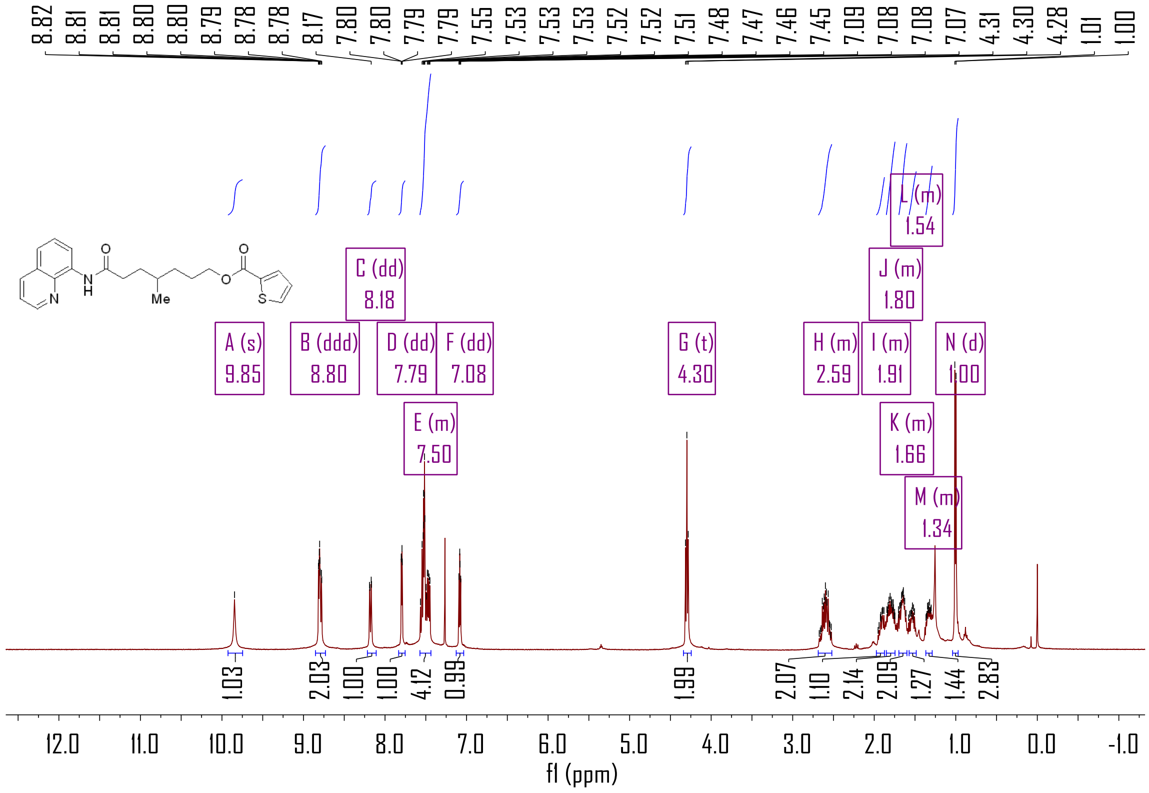

Supplementary Figure 128. ^{1}H NMR spectra of **4ao**

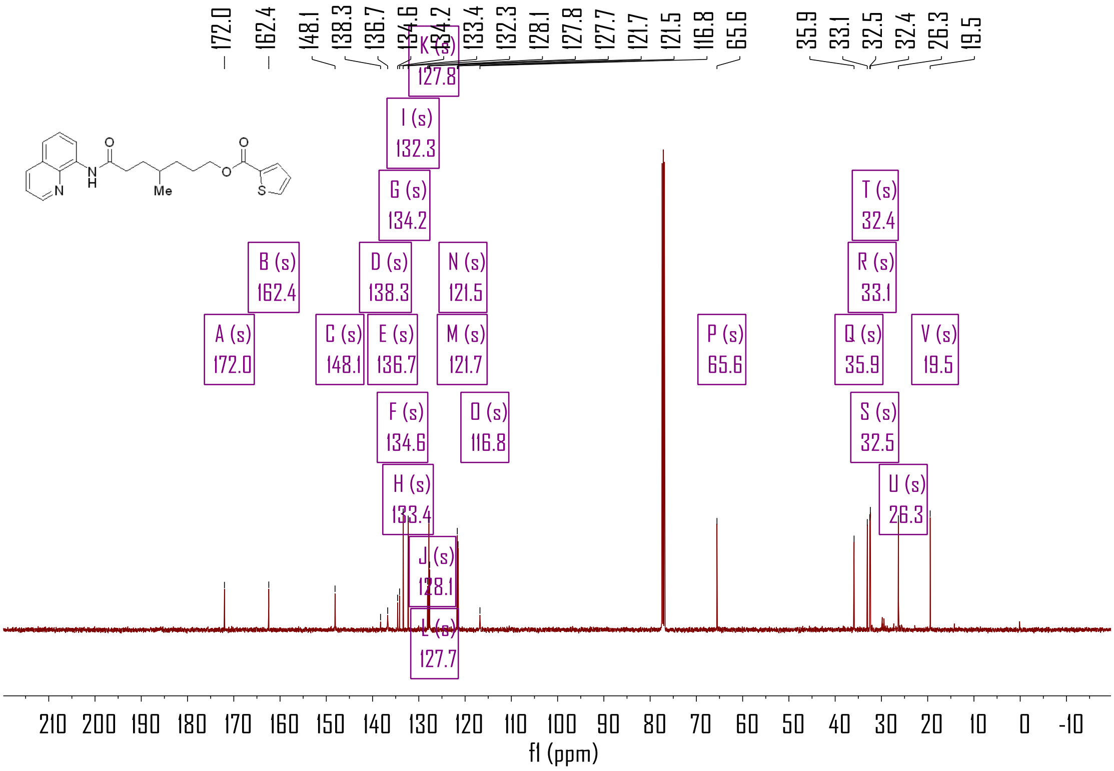

Supplementary Figure 129. ^{13}C NMR spectra of **4ao**

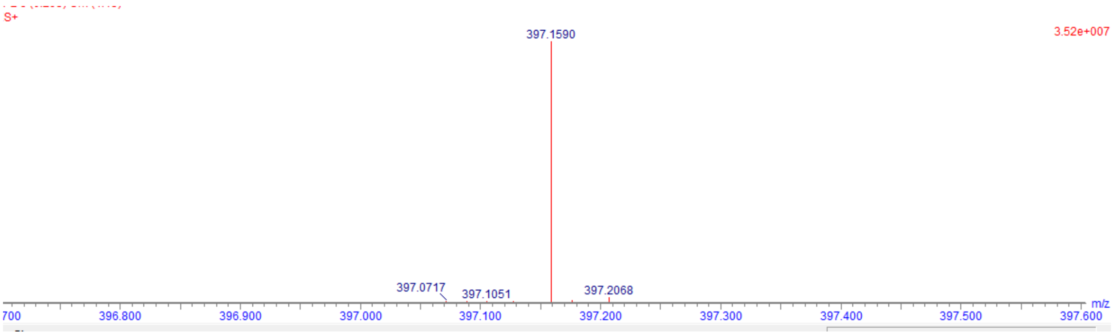

Supplementary Figure 130. HRMS spectra of **4ao**

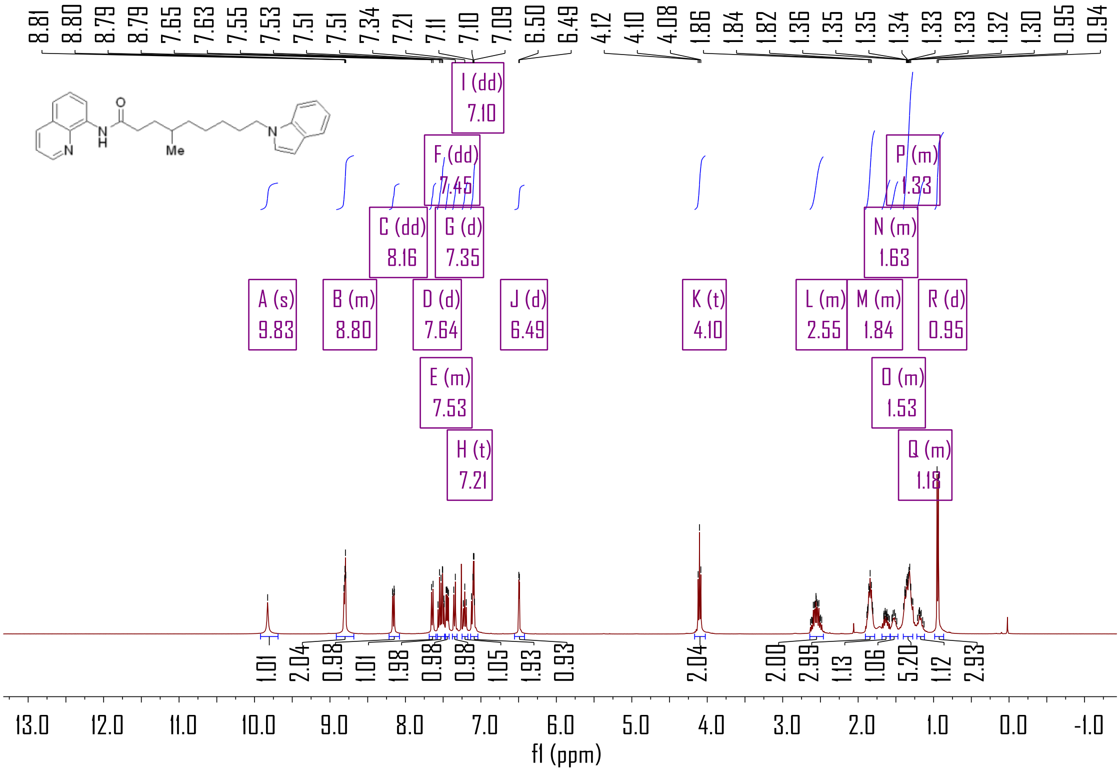

Supplementary Figure 131. ^{1}H NMR spectra of **4ap**

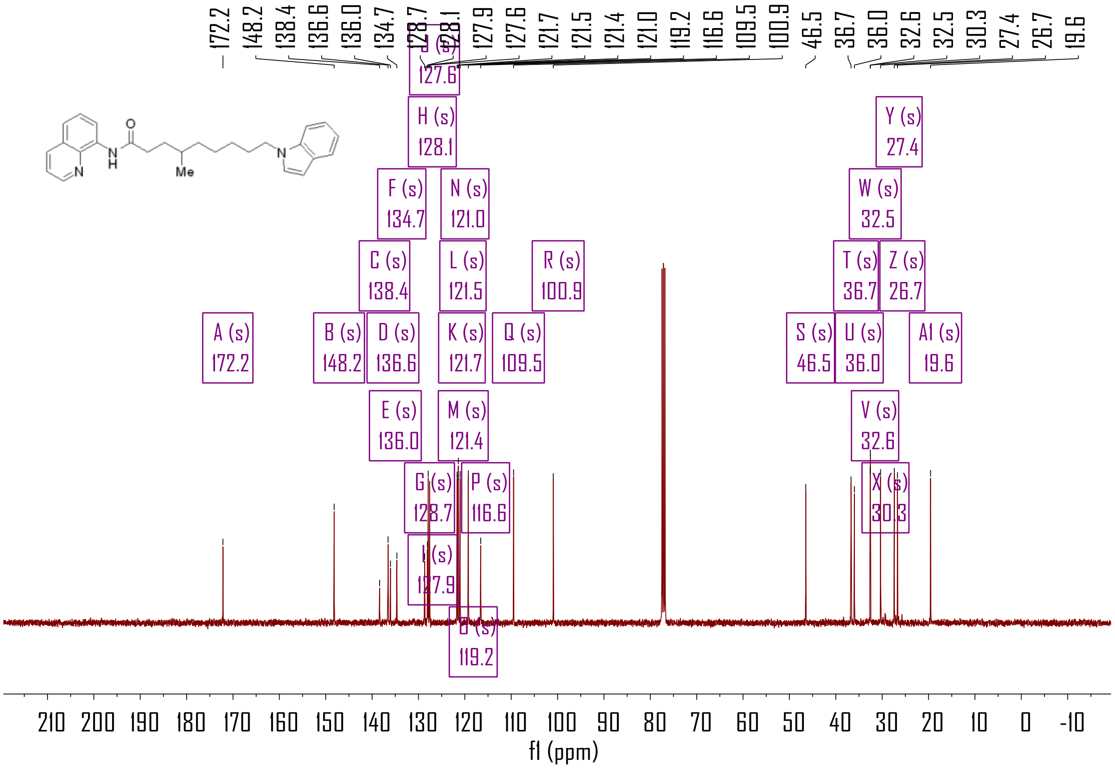

Supplementary Figure 132. ^{13}C NMR spectra of **4ap**

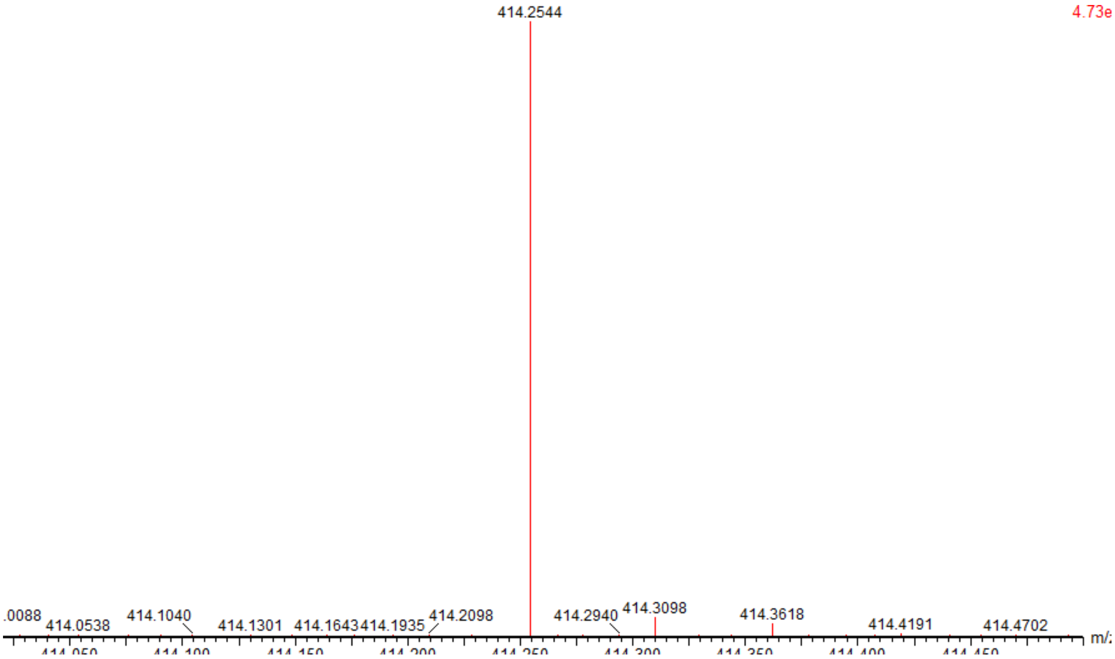

Supplementary Figure 133. HRMS spectra of **4ae**

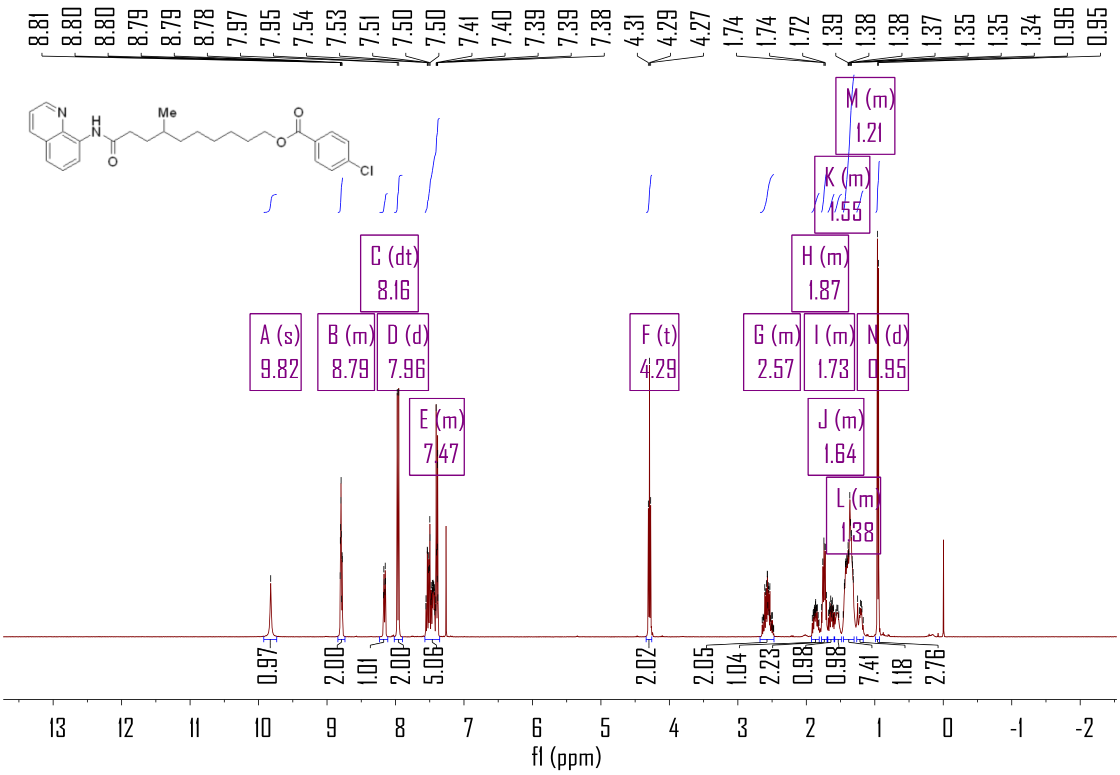

Supplementary Figure 134. ^{1}H NMR spectra of **4aq**

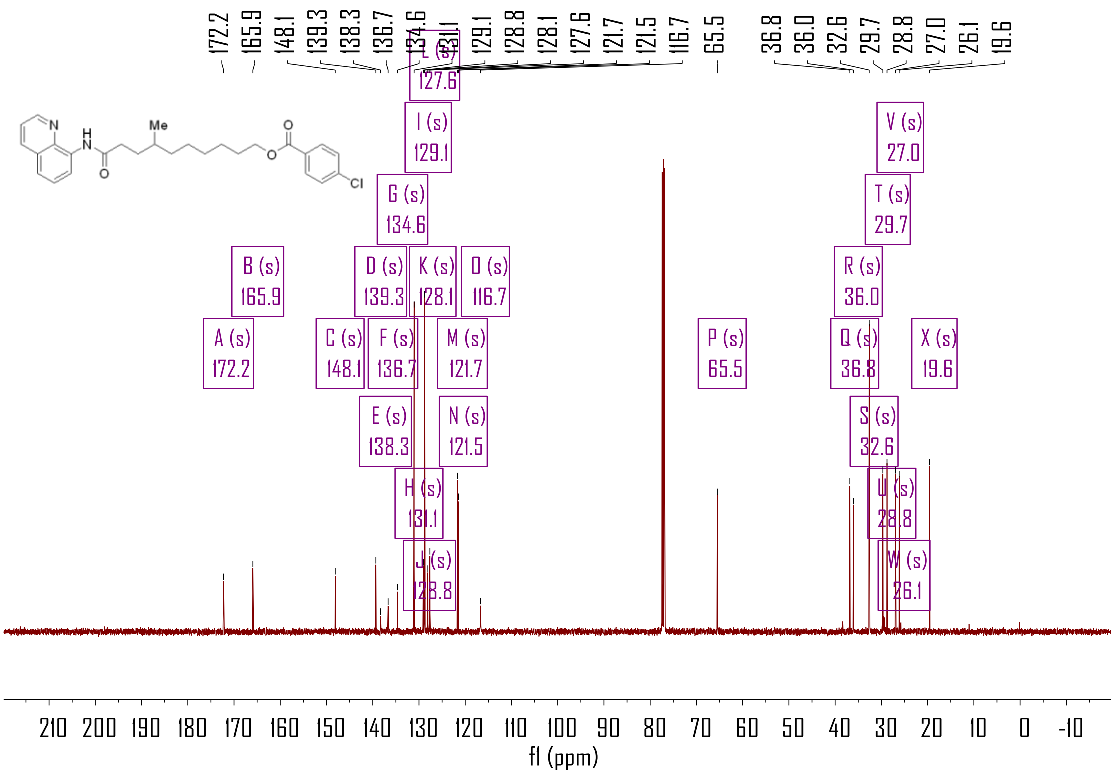

Supplementary Figure 135. ^{13}C NMR spectra of **4aq**

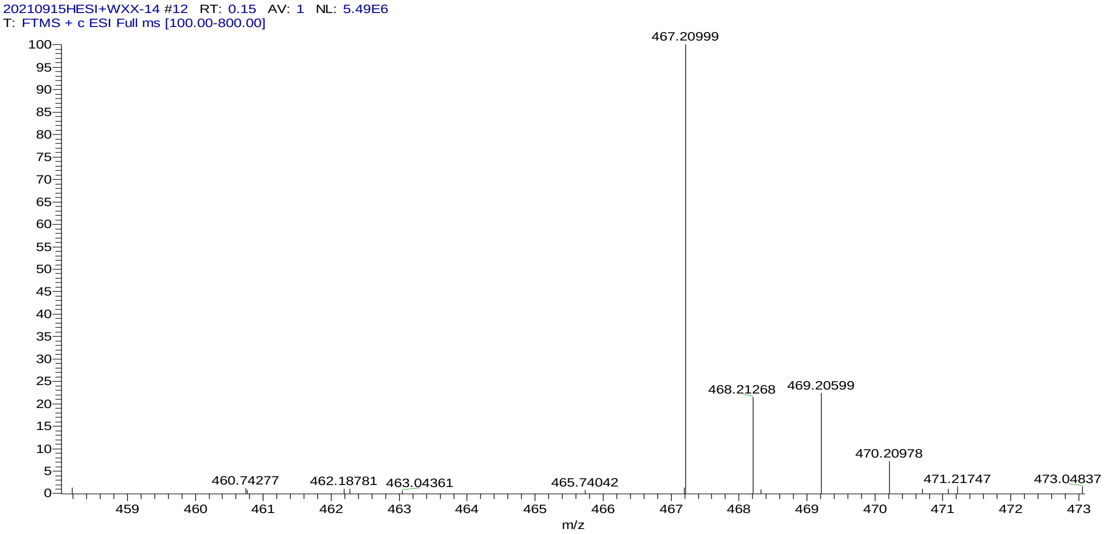

Supplementary Figure 136. HRMS spectra of **4aq**

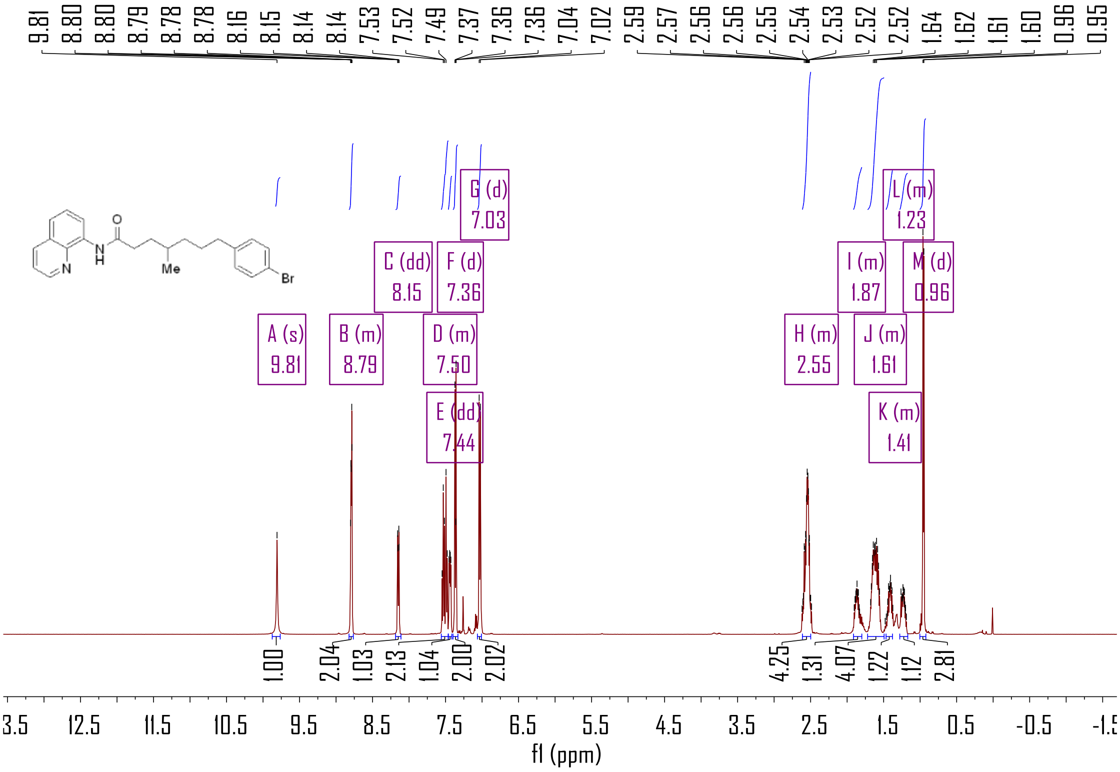

Supplementary Figure 137. ^{1}H NMR spectra of **4ar**

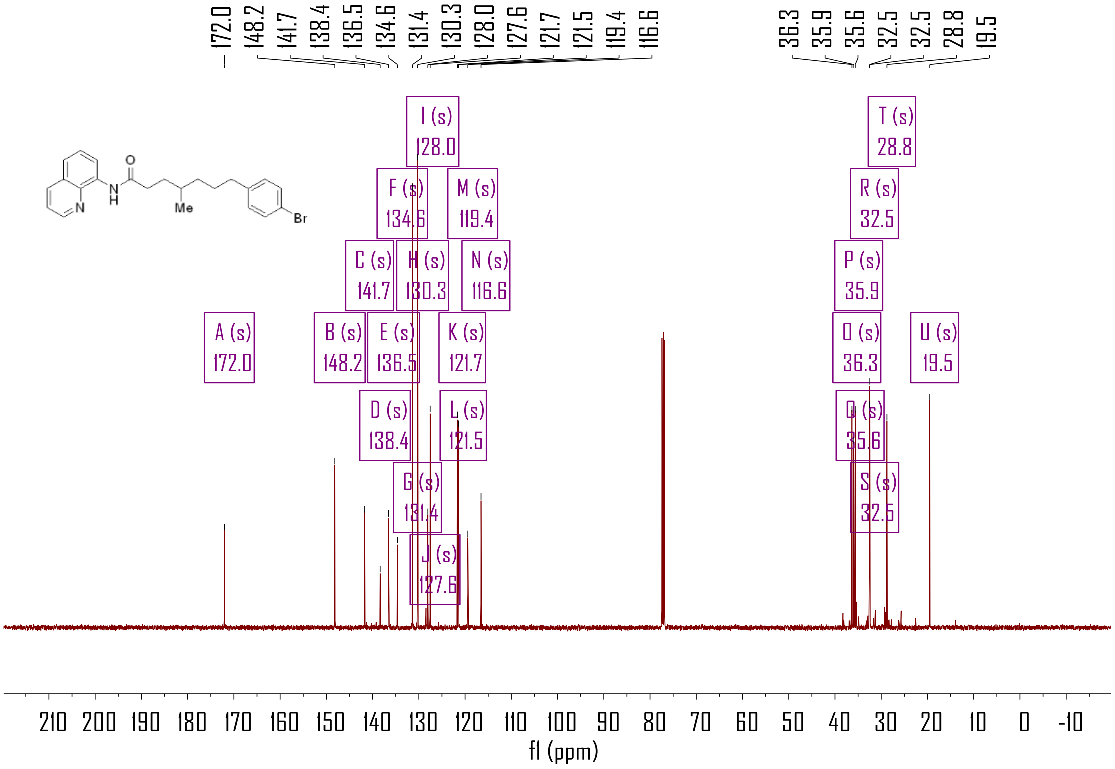

Supplementary Figure 138. ^{13}C NMR spectra of **4ar**

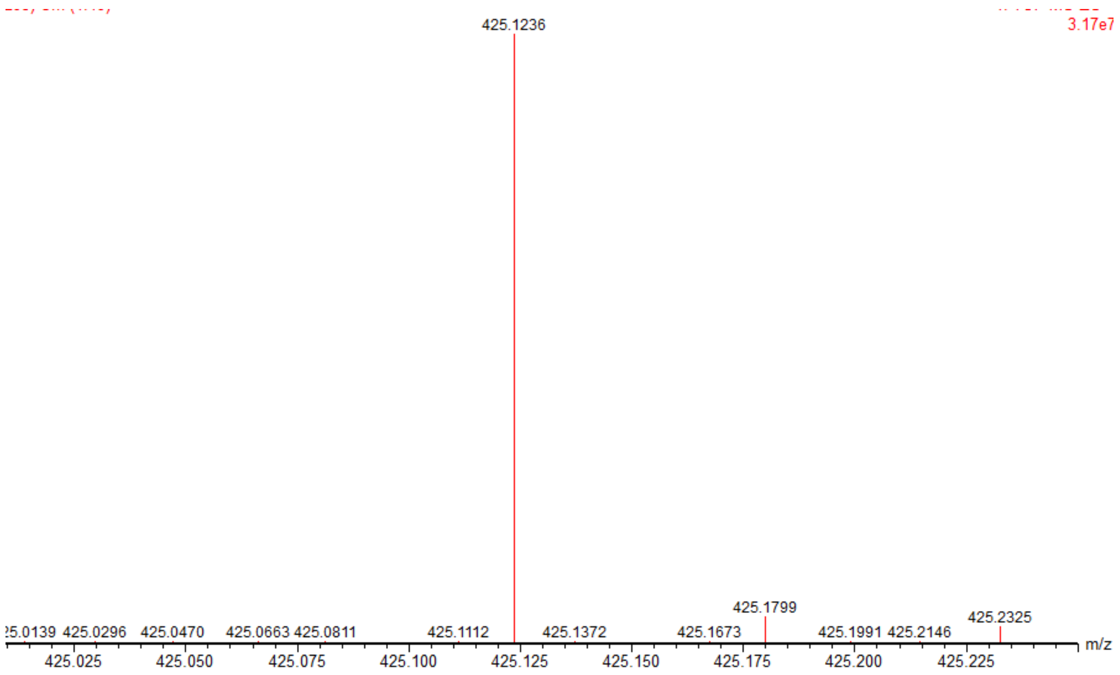

Supplementary Figure 139. HRMS spectra of **4ar**

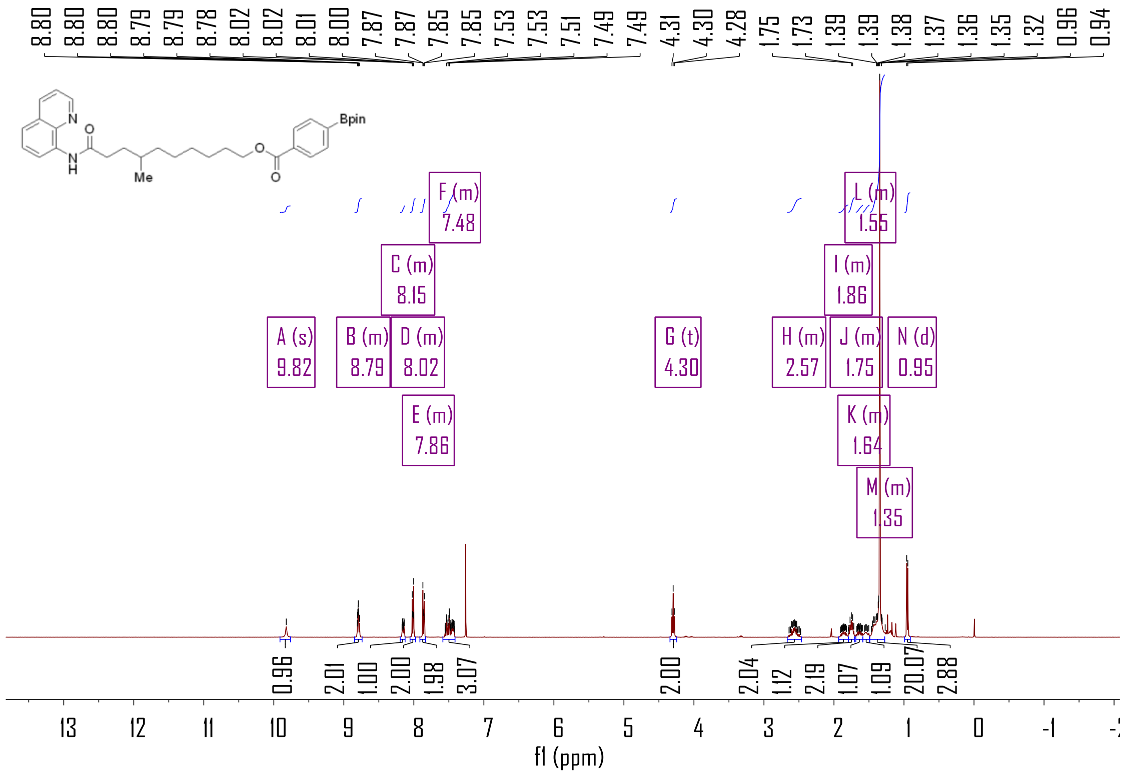

Supplementary Figure 140. ^{1}H NMR spectra of **4as**

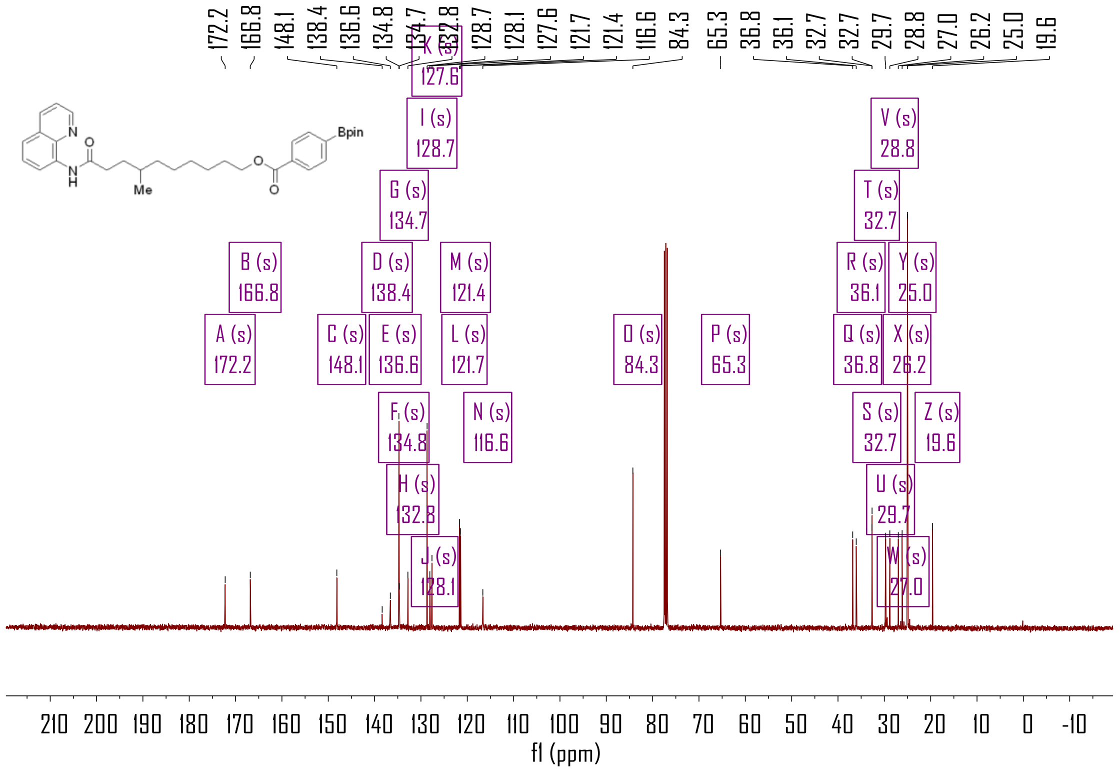

Supplementary Figure 141. ^{13}C NMR spectra of **4as**

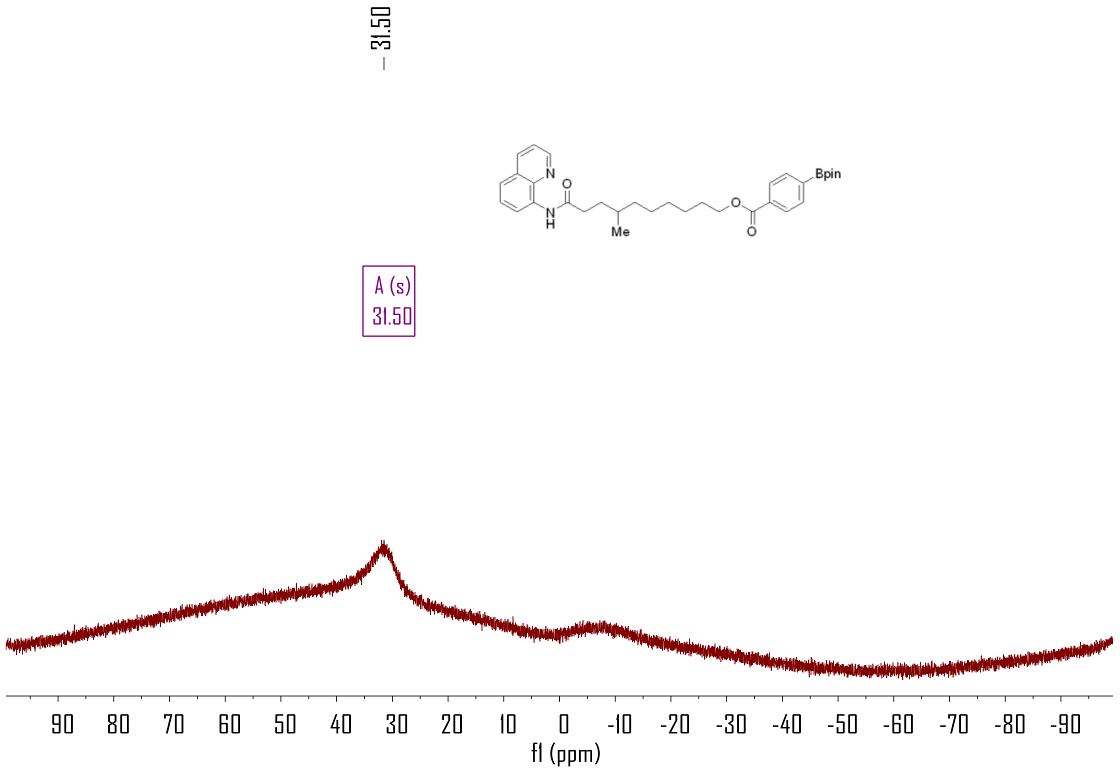

Supplementary Figure 142. ^{11}B NMR spectra of **4as**

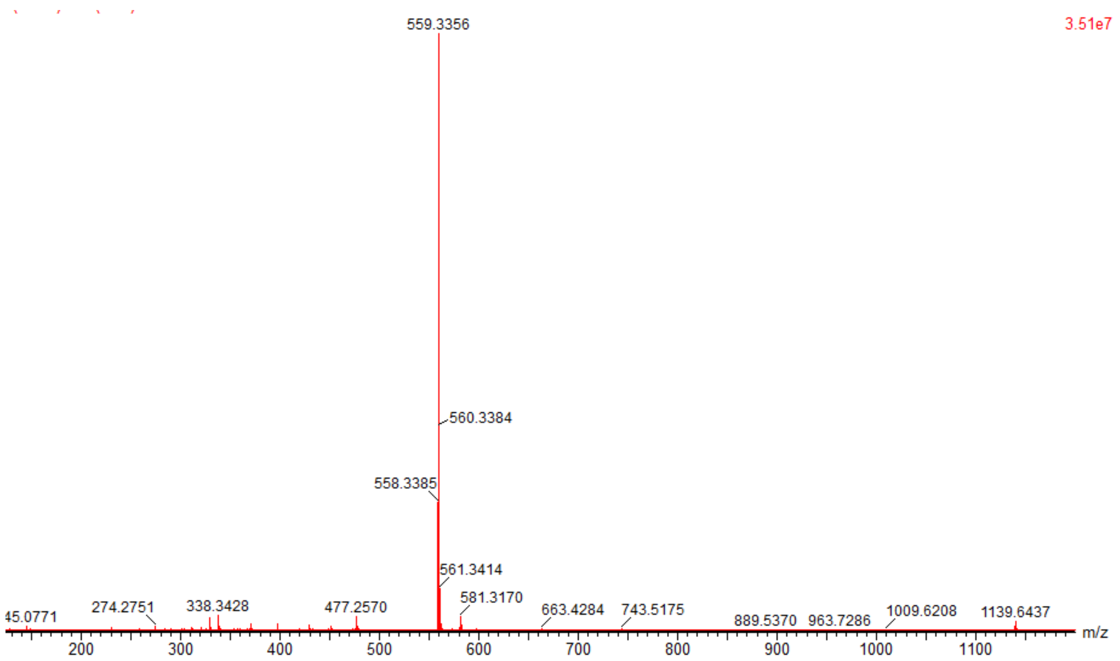

Supplementary Figure 143. HRMS spectra of **4as**

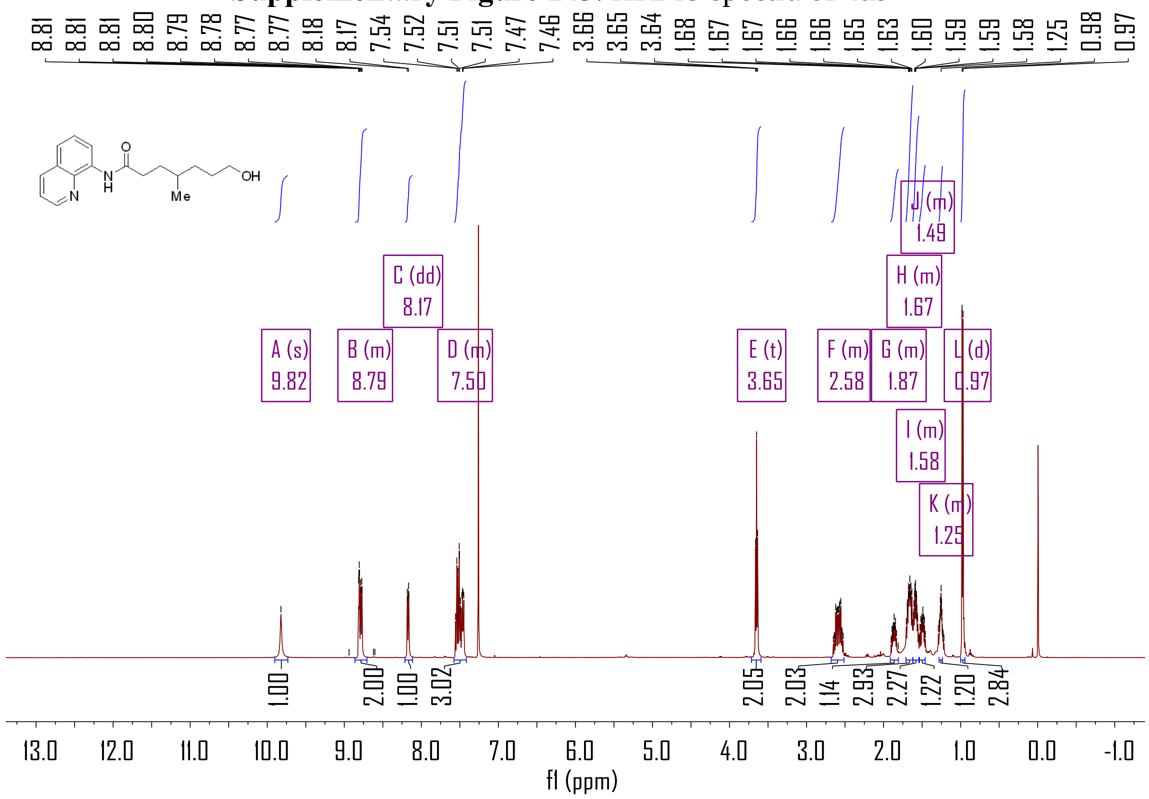

Supplementary Figure 144. ^{1}H NMR spectra of **4at**

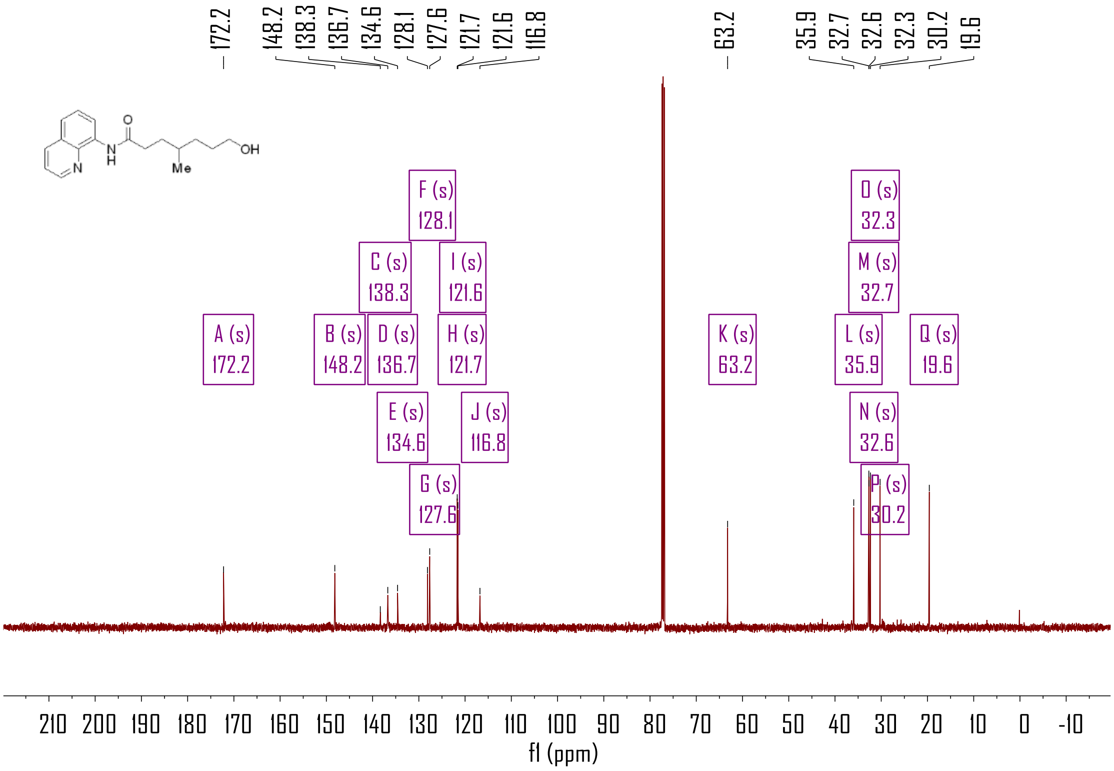

Supplementary Figure 145. ^{13}C NMR spectra of **4at**

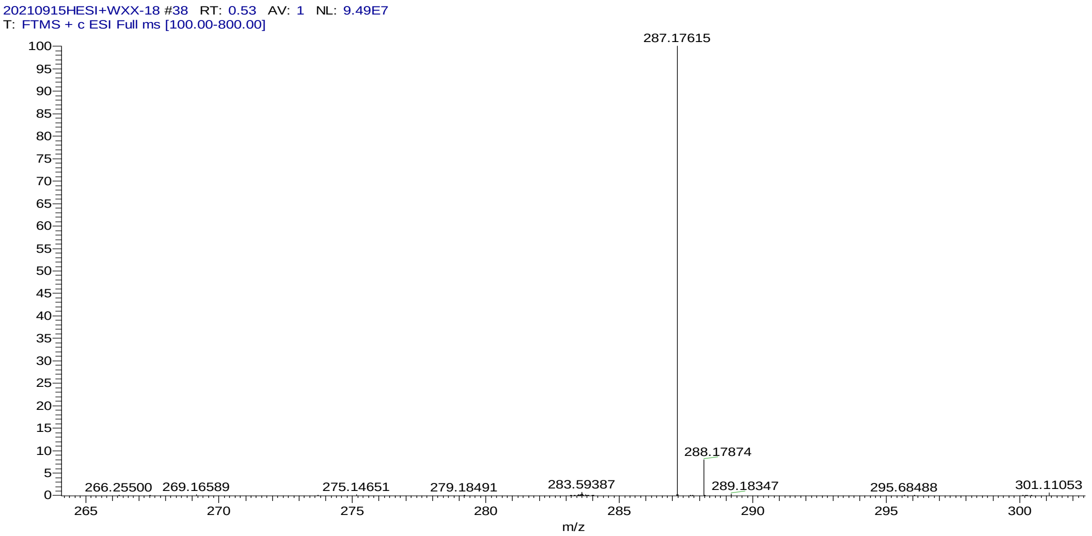

Supplementary Figure 146. HRMS spectra of **4at**

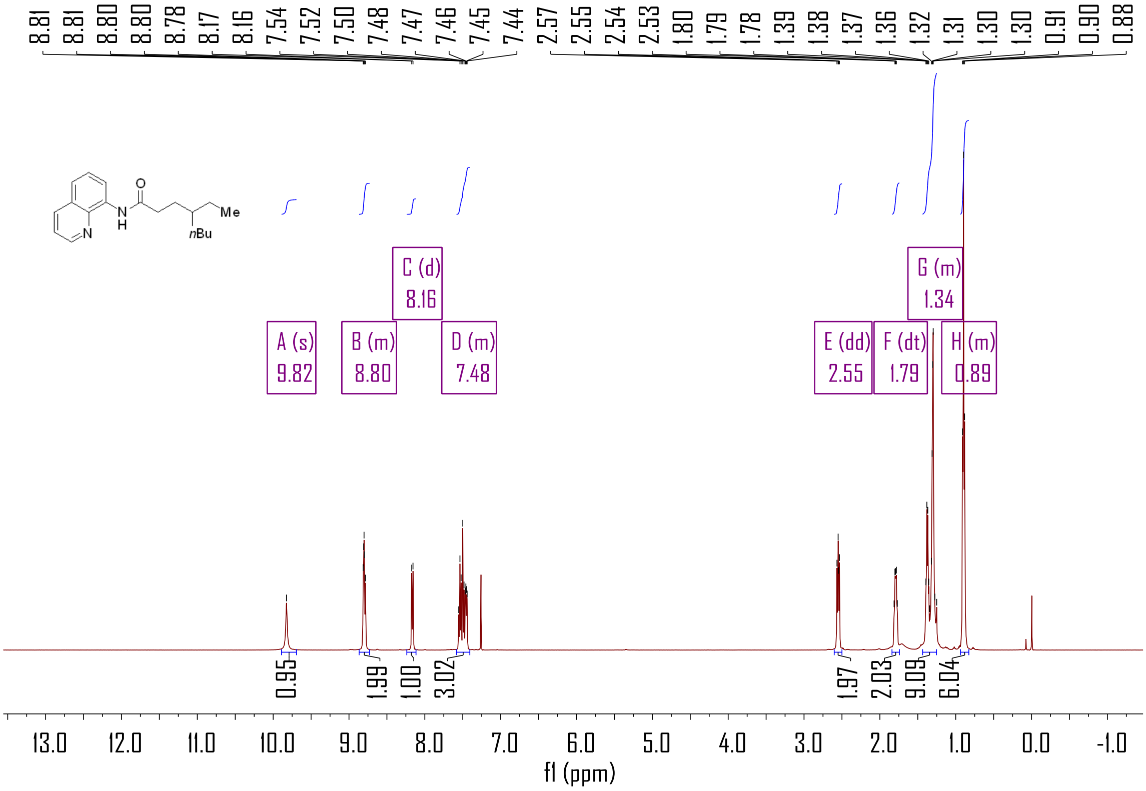

Supplementary Figure 147. ^{1}H NMR spectra of **4ba**

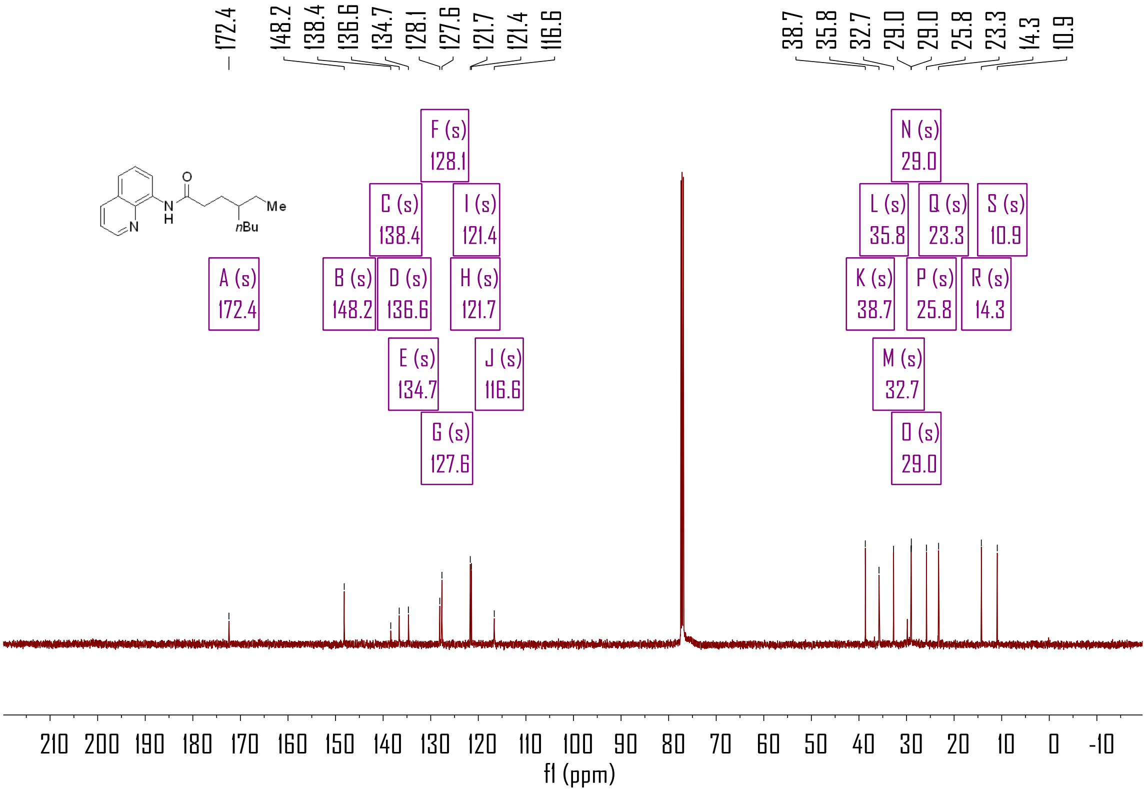

Supplementary Figure 148. ^{13}C NMR spectra of **4ba**

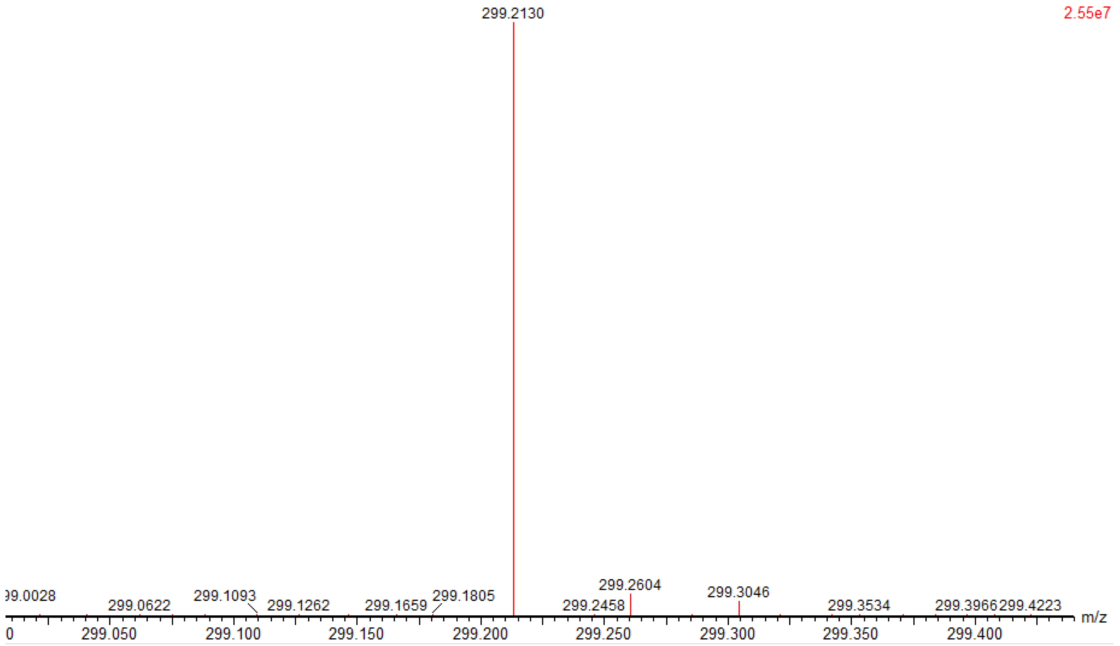

Supplementary Figure 149. HRMS spectra of **4ba**

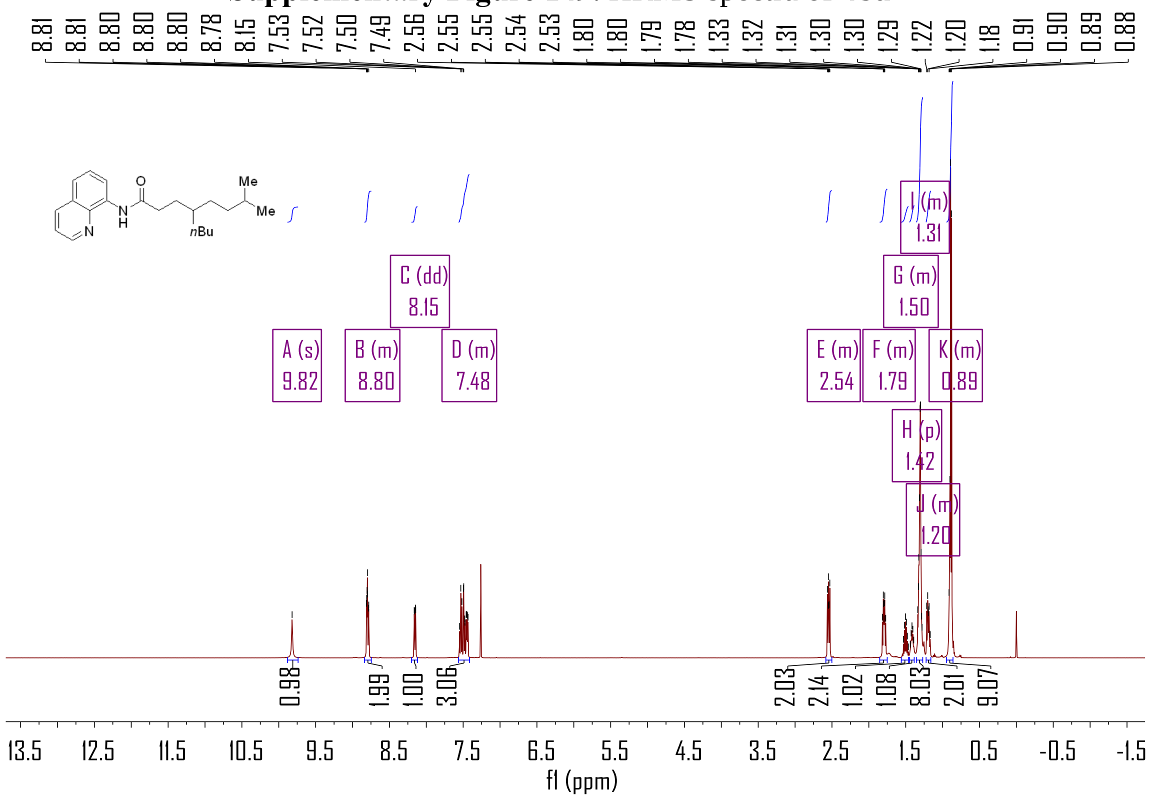

Supplementary Figure 150. ^{1}H NMR spectra of **4ca**

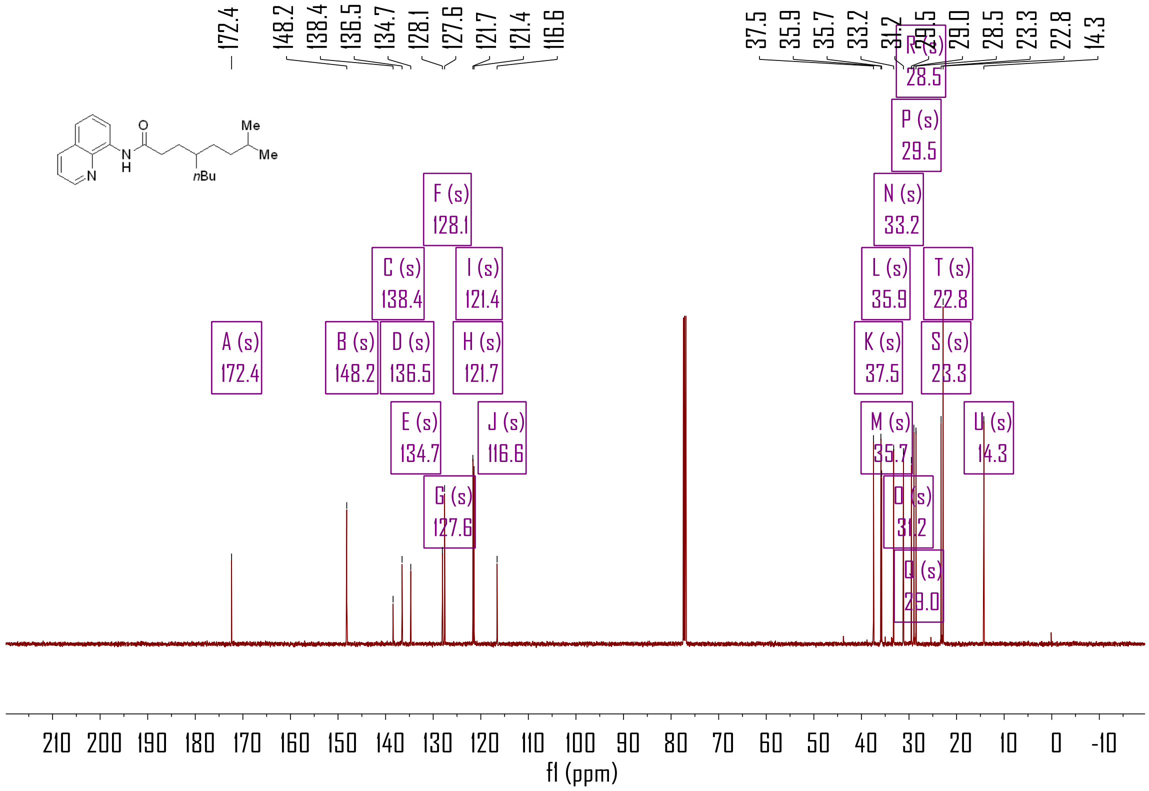

Supplementary Figure 151. ^{13}C NMR spectra of **4ca**

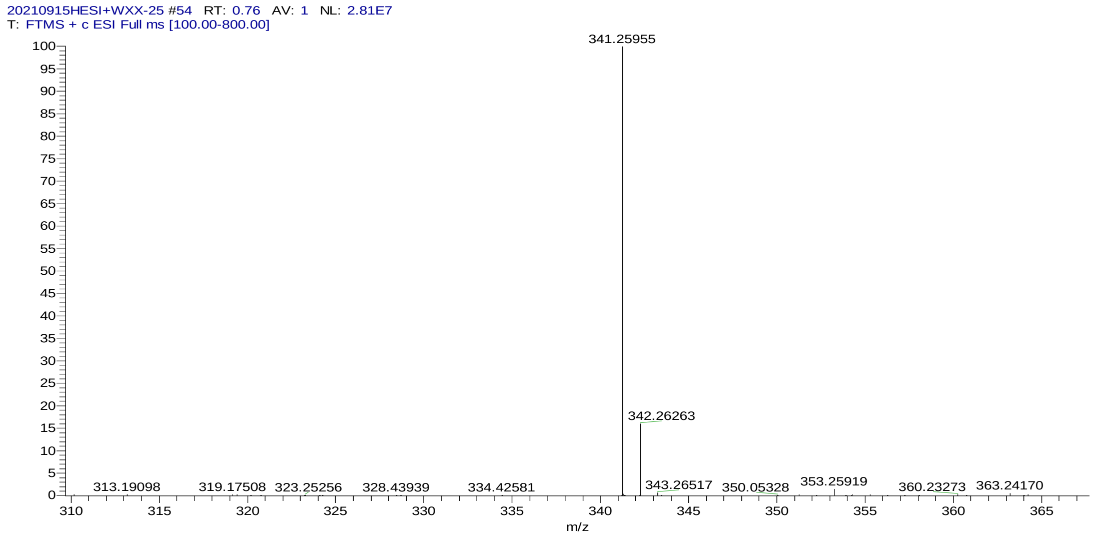

Supplementary Figure 152. HRMS spectra of **4ca**

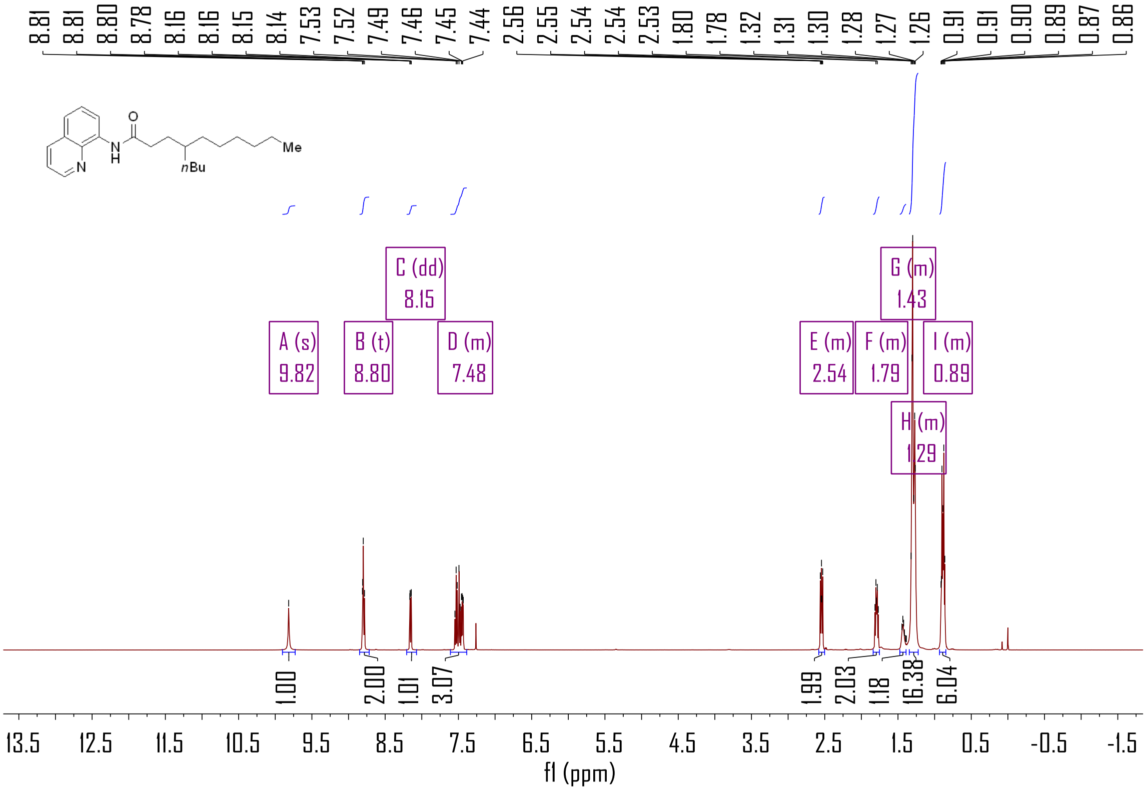

Supplementary Figure 153. ^{1}H NMR spectra of **4da**

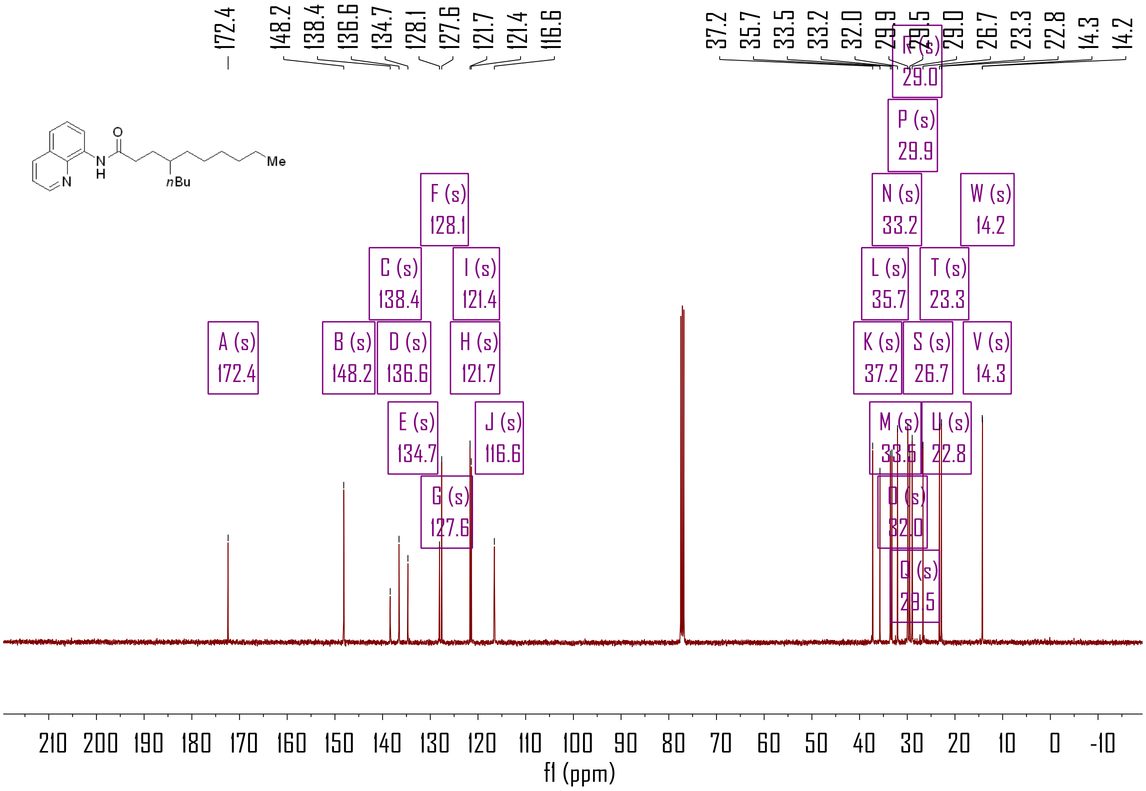

Supplementary Figure 154. ^{13}C NMR spectra of **4da**

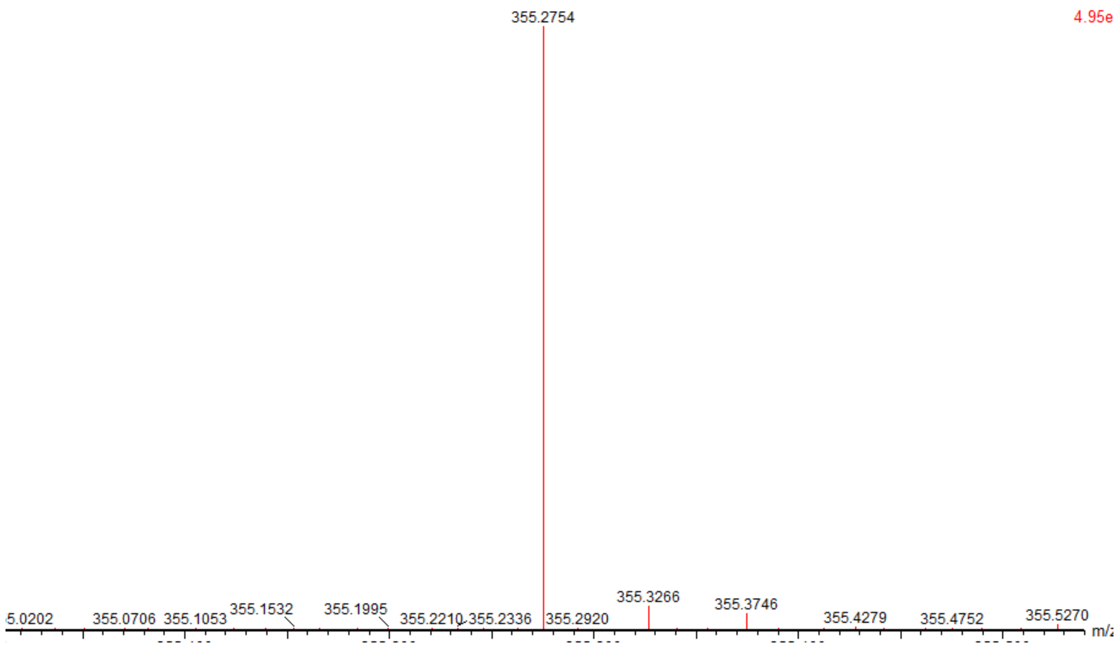

Supplementary Figure 155. HRMS spectra of **4da**

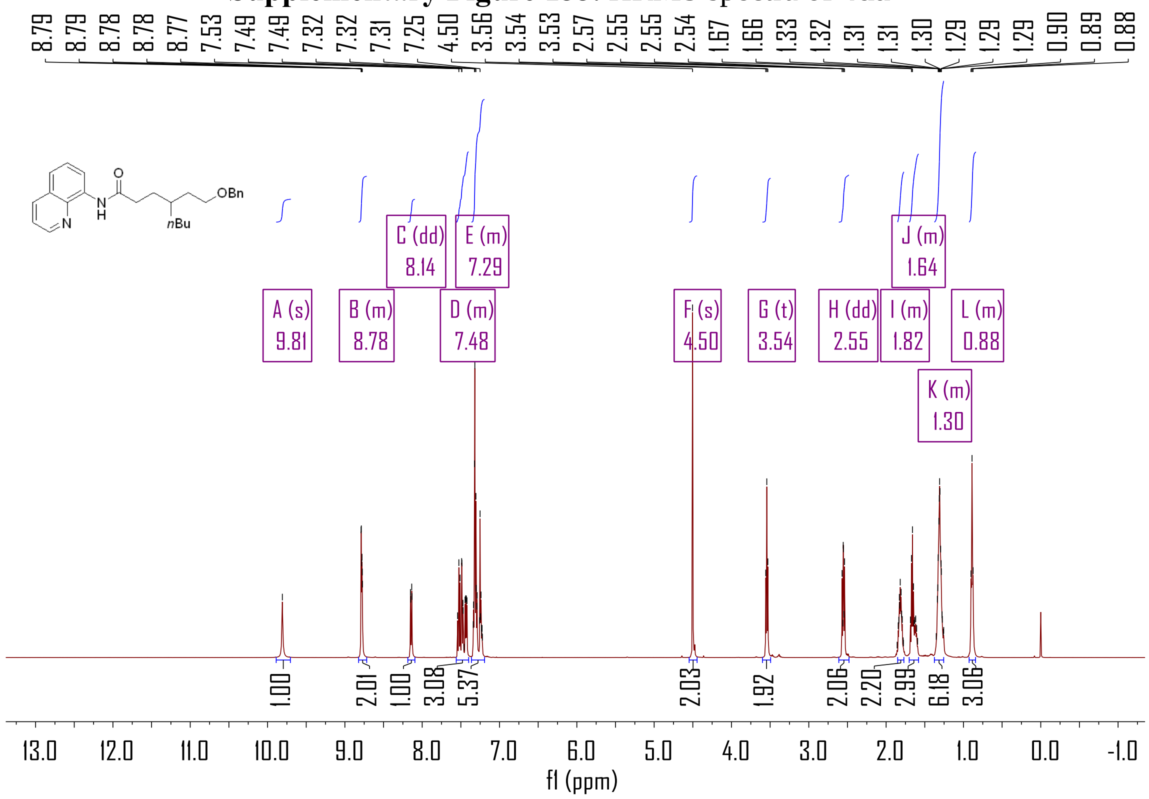

Supplementary Figure 156. ^{1}H NMR spectra of **4ea**

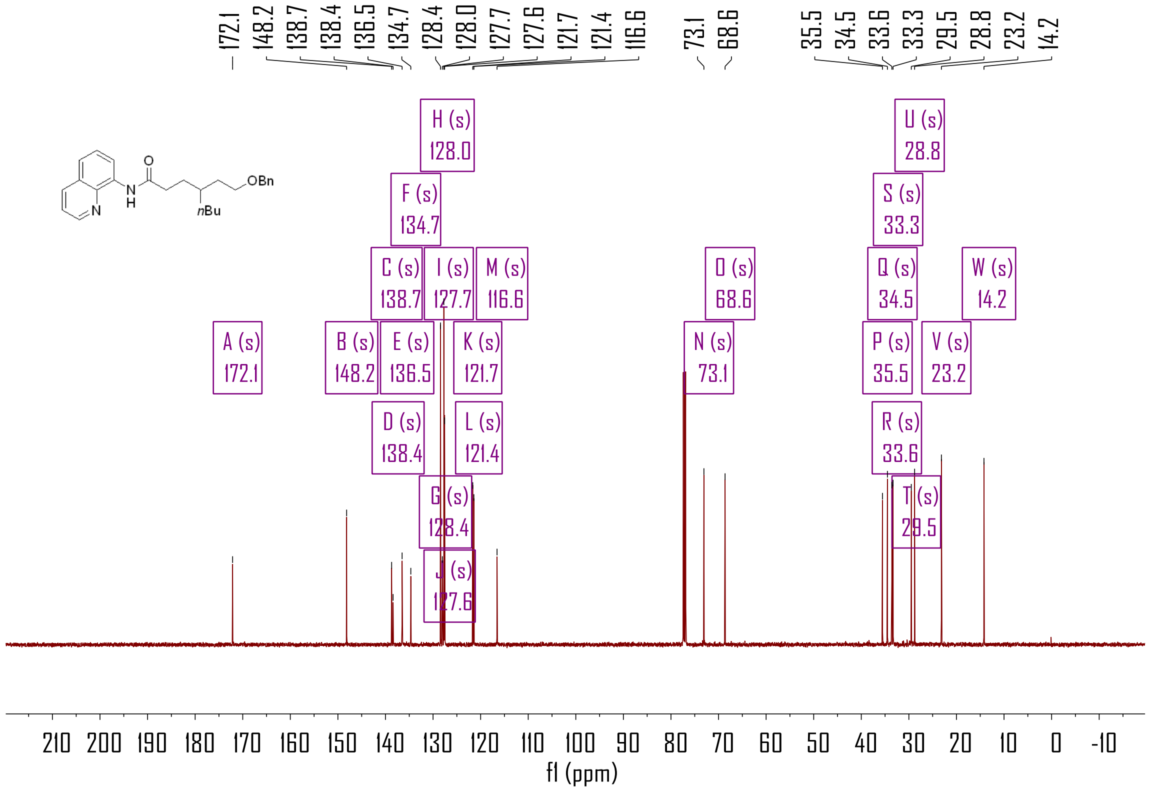

Supplementary Figure 157. ^{13}C NMR spectra of **4ea**

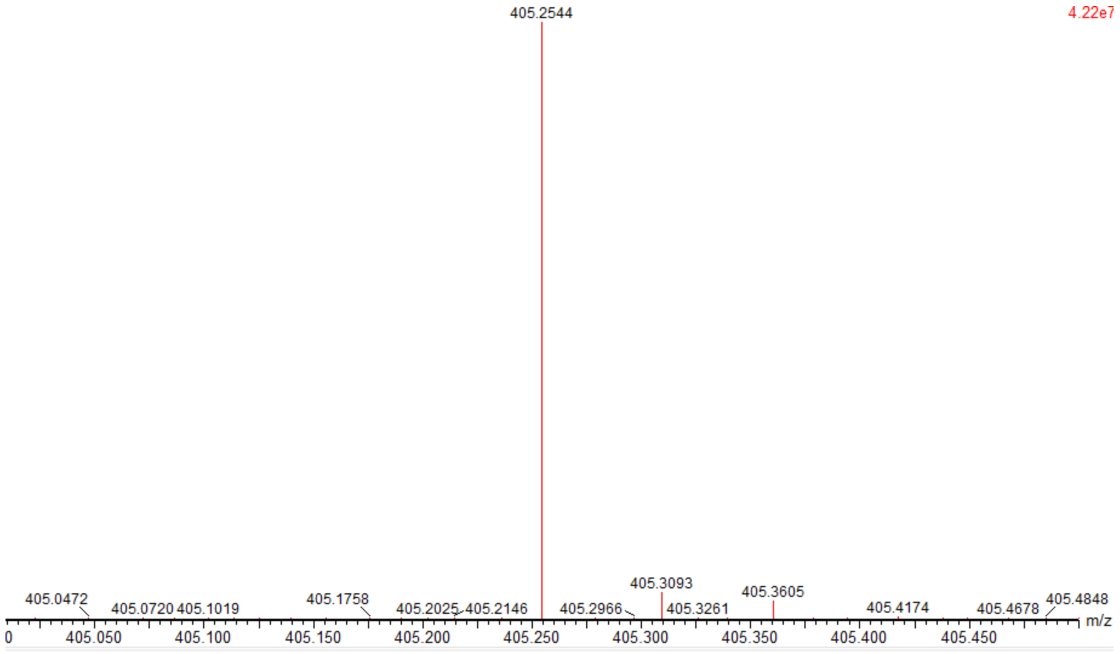

Supplementary Figure 158. HRMS spectra of **4ea**

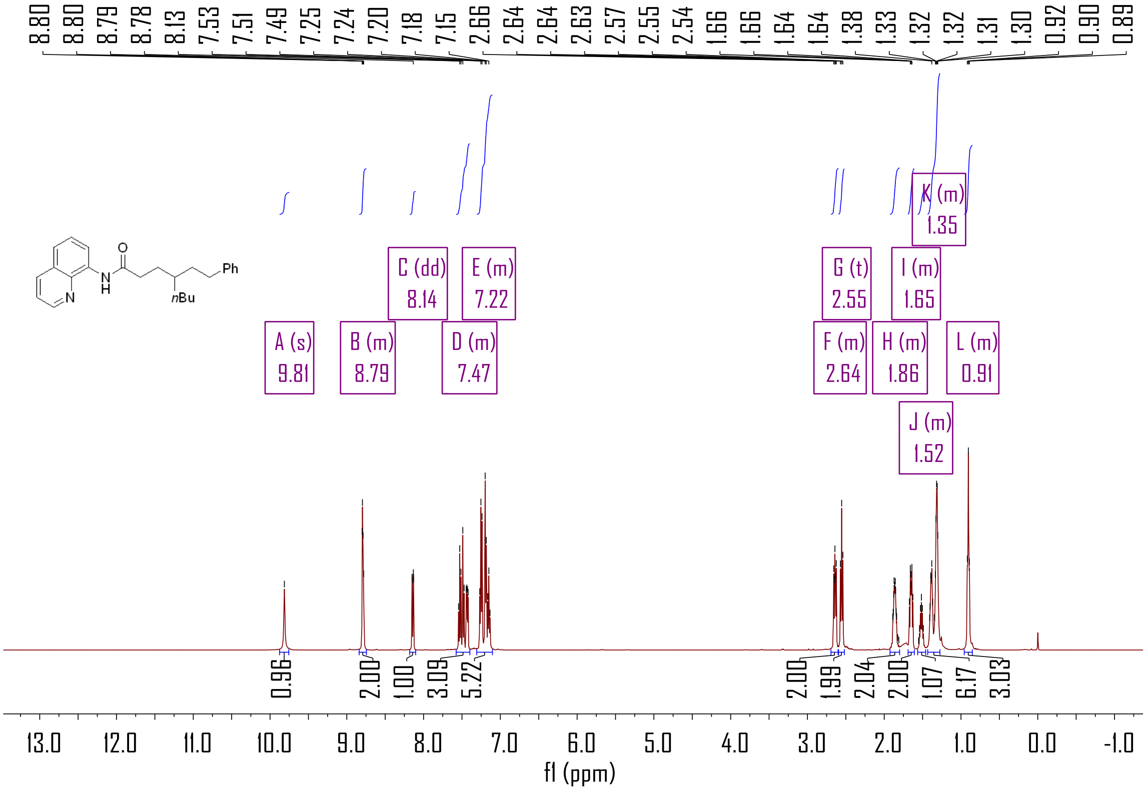

Supplementary Figure 159. ^{1}H NMR spectra of **4fa**

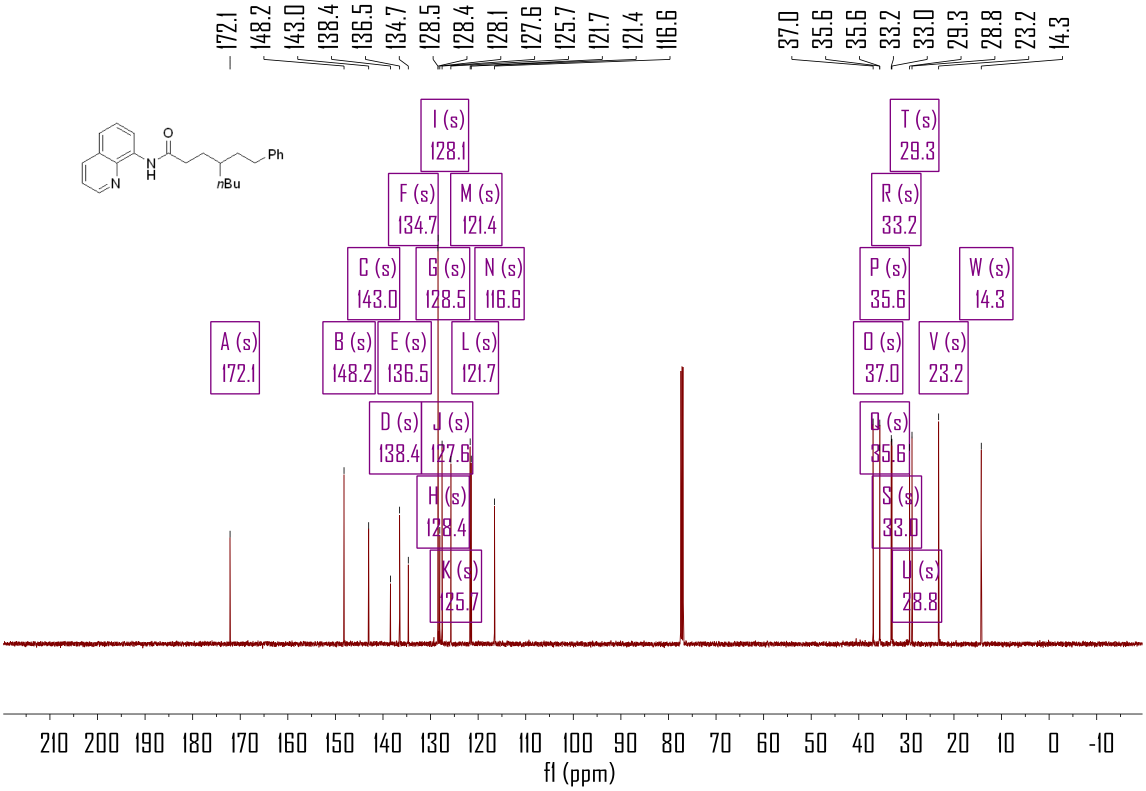

Supplementary Figure 160. ^{13}C NMR spectra of **4fa**

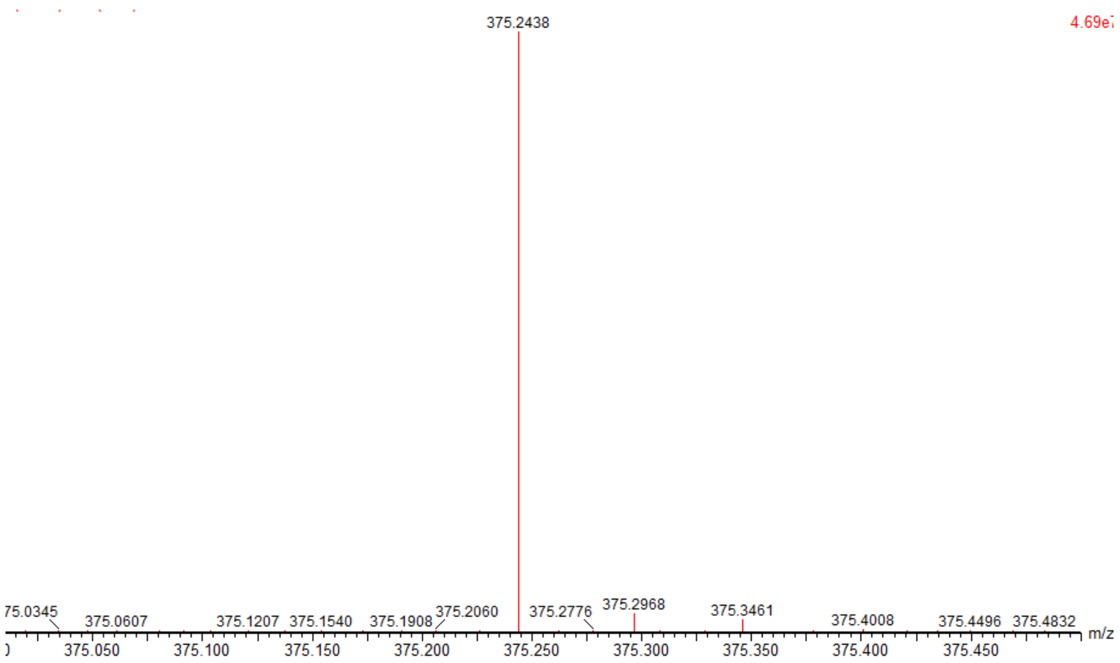

Supplementary Figure 161. HRMS spectra of **4fa**

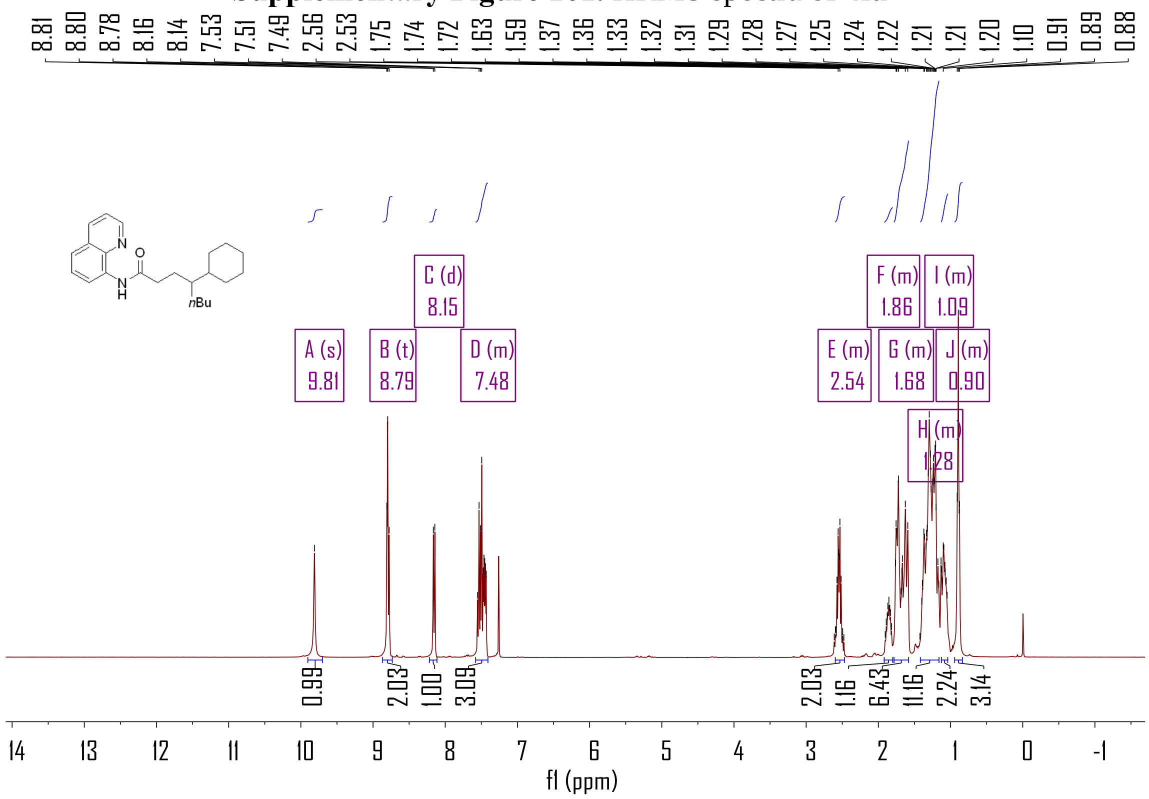

Supplementary Figure 162. ^{1}H NMR spectra of **4ga**

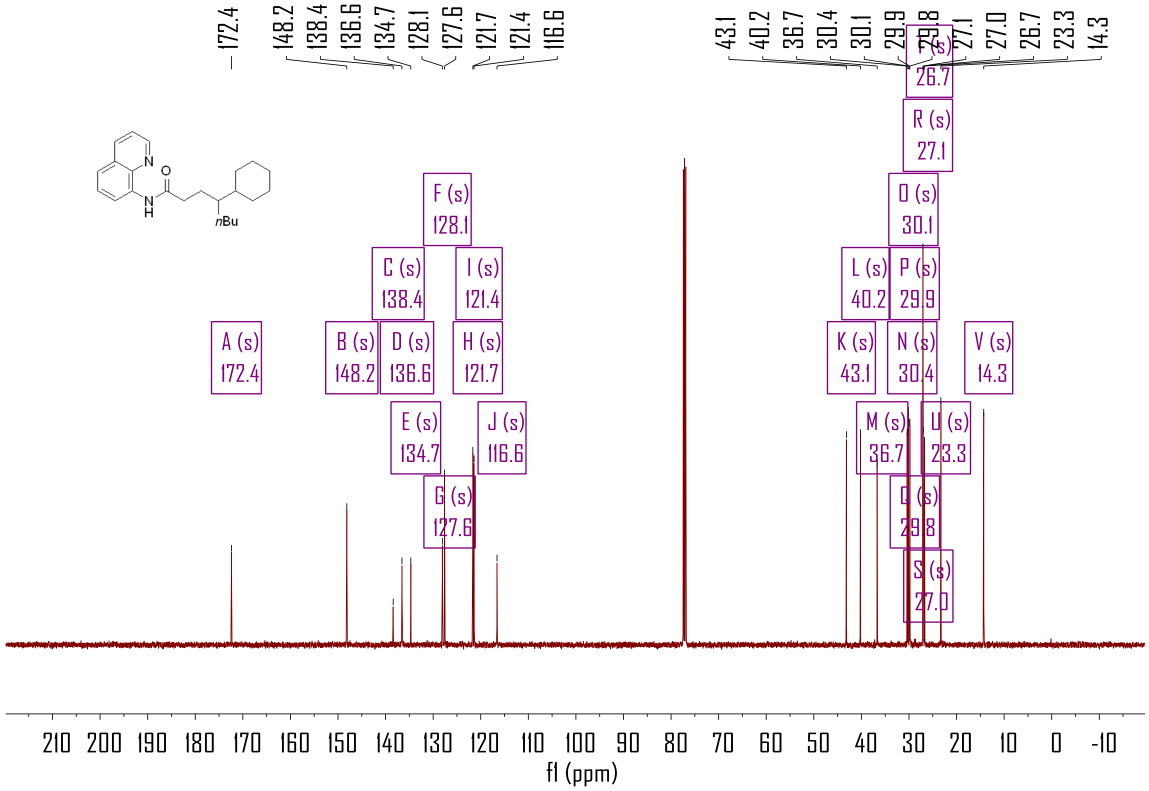

Supplementary Figure 163. ^{13}C NMR spectra of **4ga**

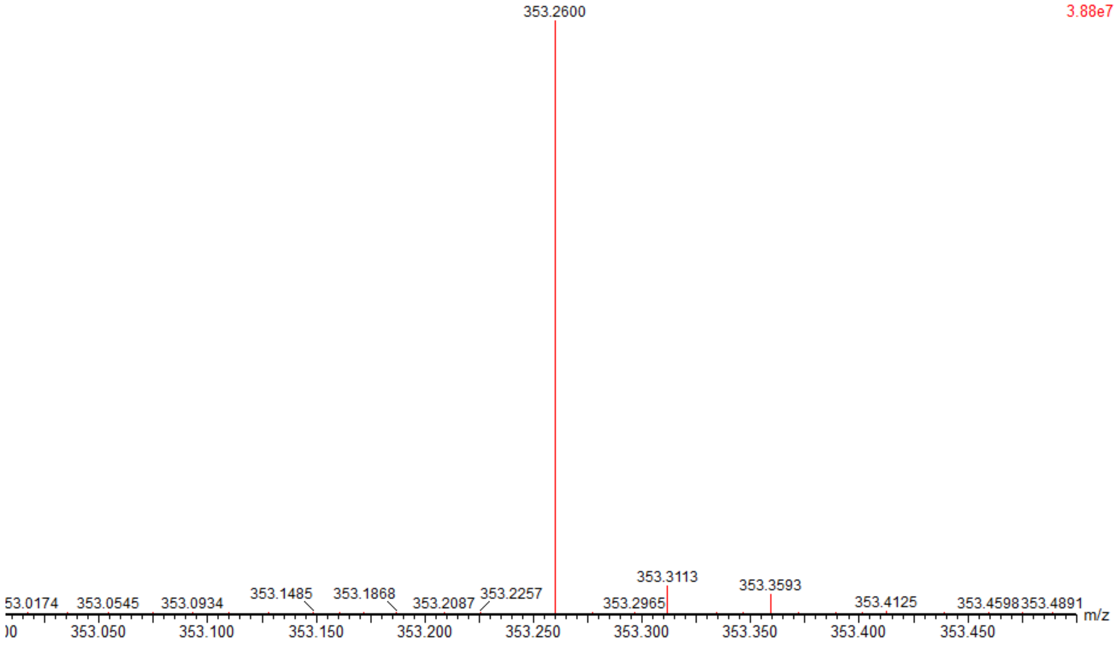

Supplementary Figure 164. HRMS spectra of **4ga**

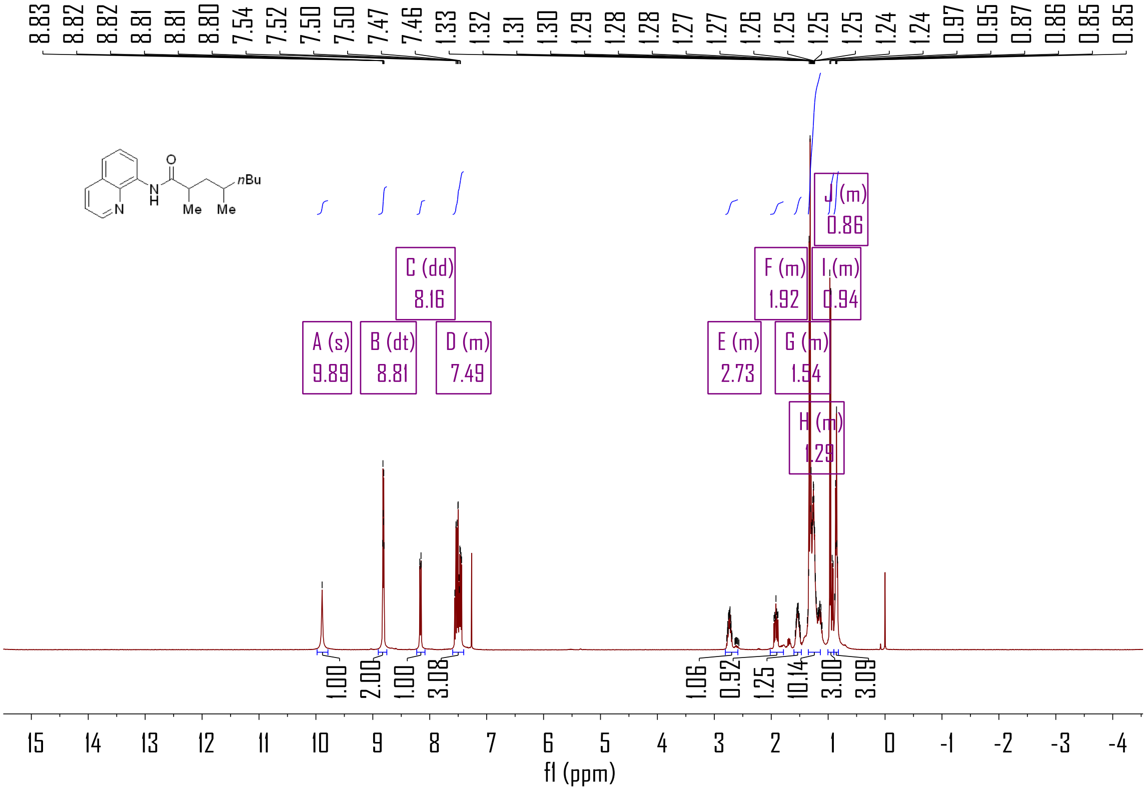

Supplementary Figure 165. ^{1}H NMR spectra of **4ha**

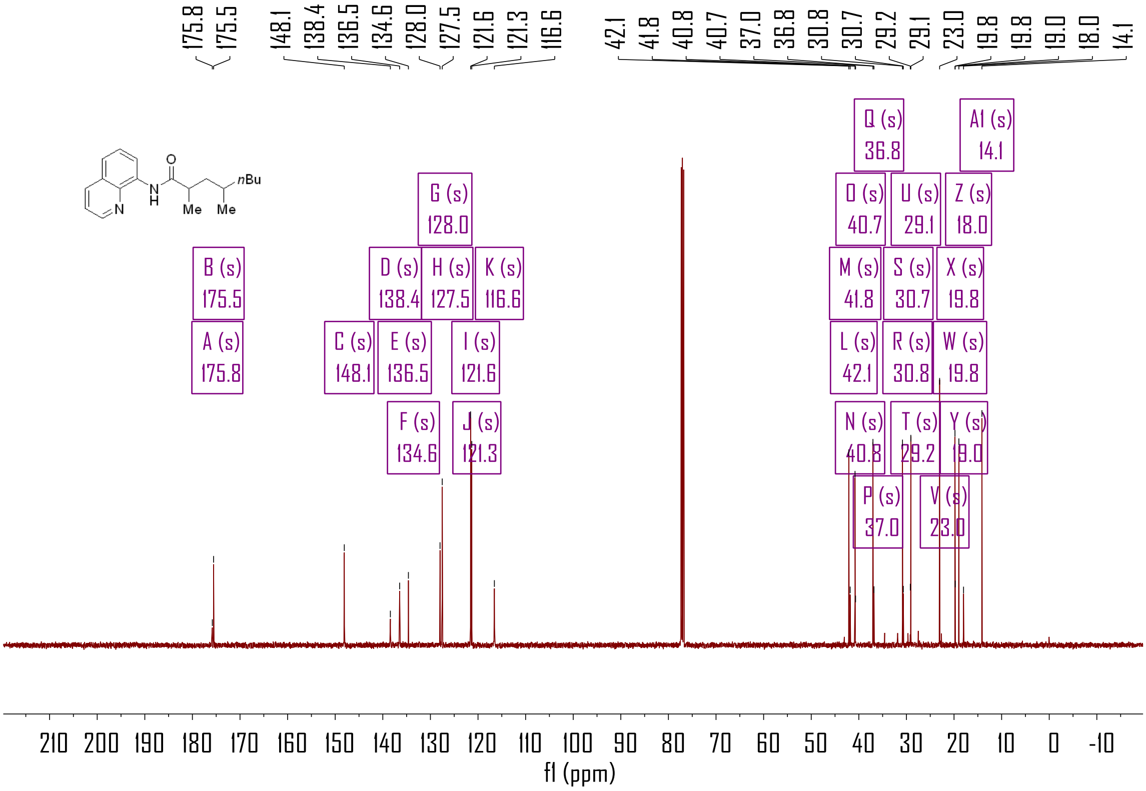

Supplementary Figure 166. ^{13}C NMR spectra of **4ha**

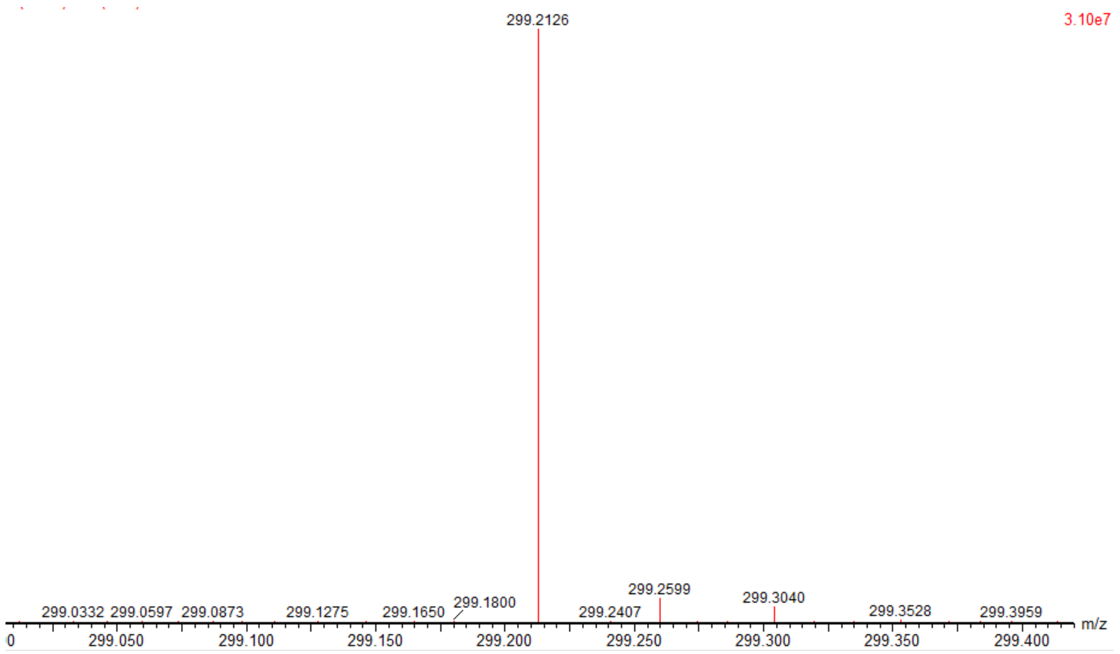

Supplementary Figure 167. HRMS spectra of **4ha**

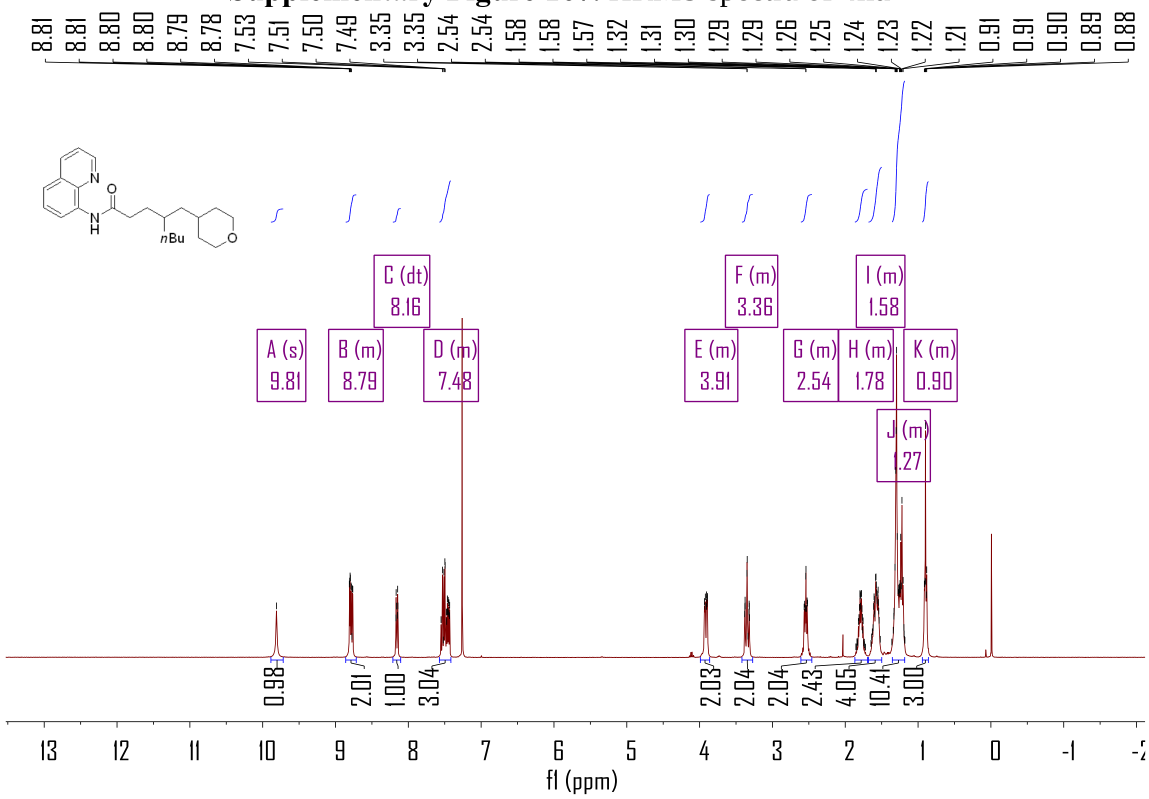

Supplementary Figure 168. ^{1}H NMR spectra of **4ia**

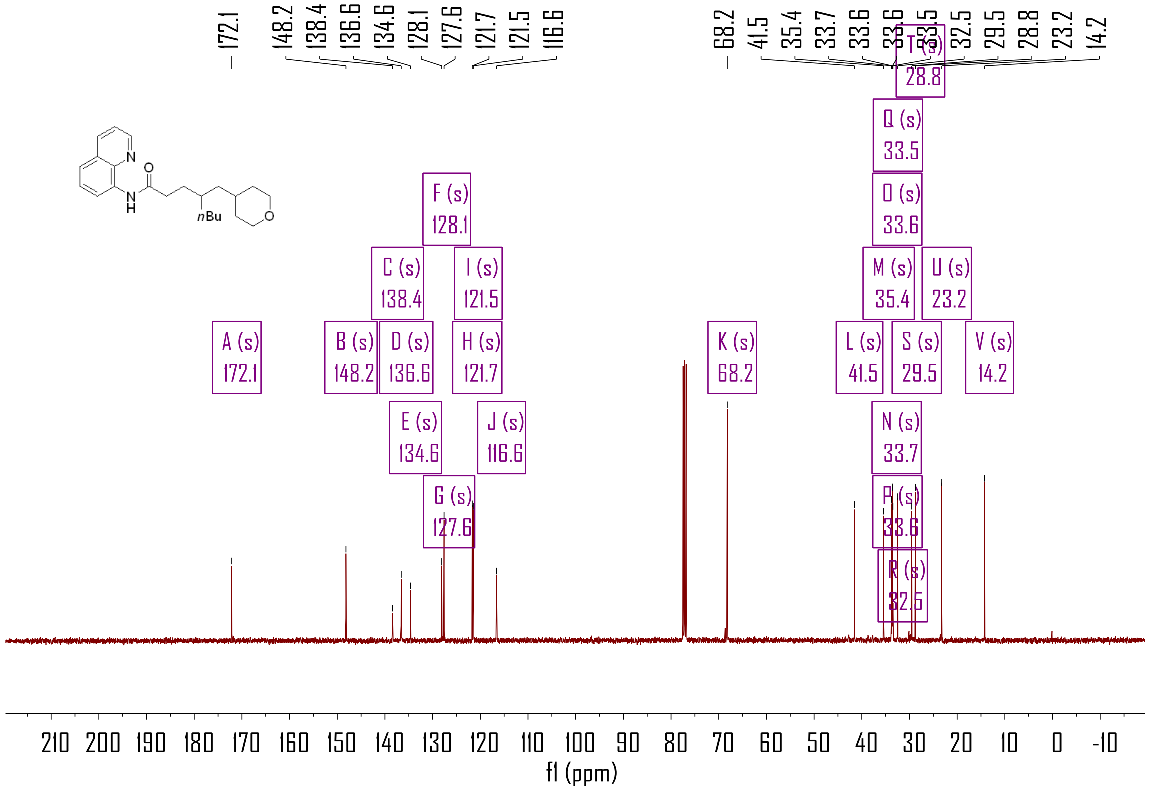

Supplementary Figure 169. ^{13}C NMR spectra of **4ia**

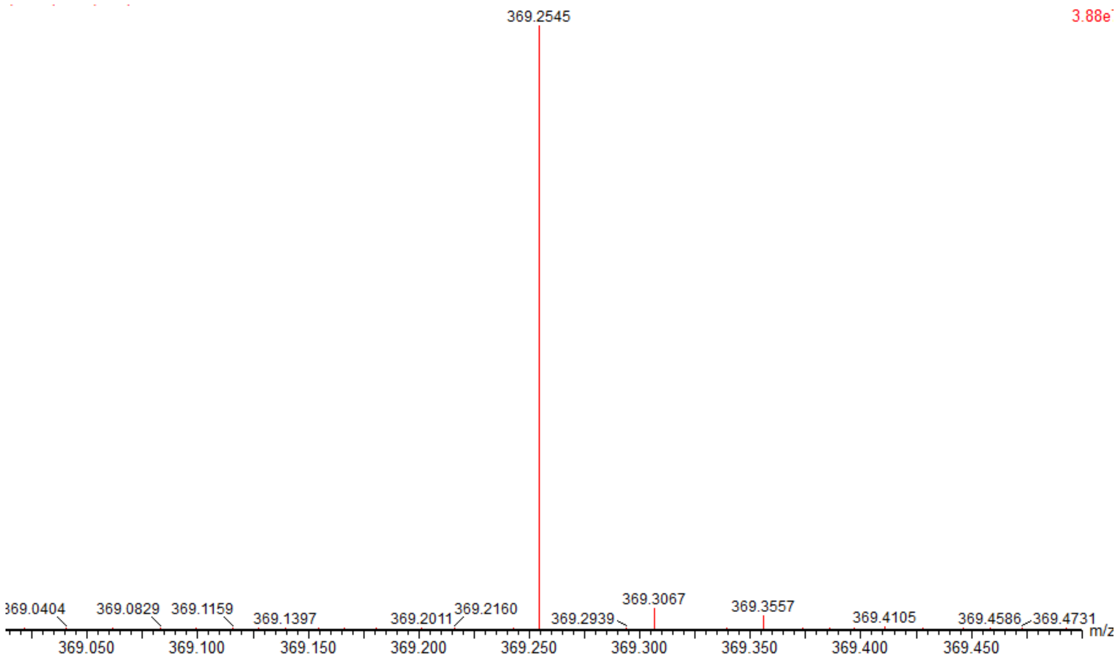

Supplementary Figure 170. HRMS spectra of **4ia**

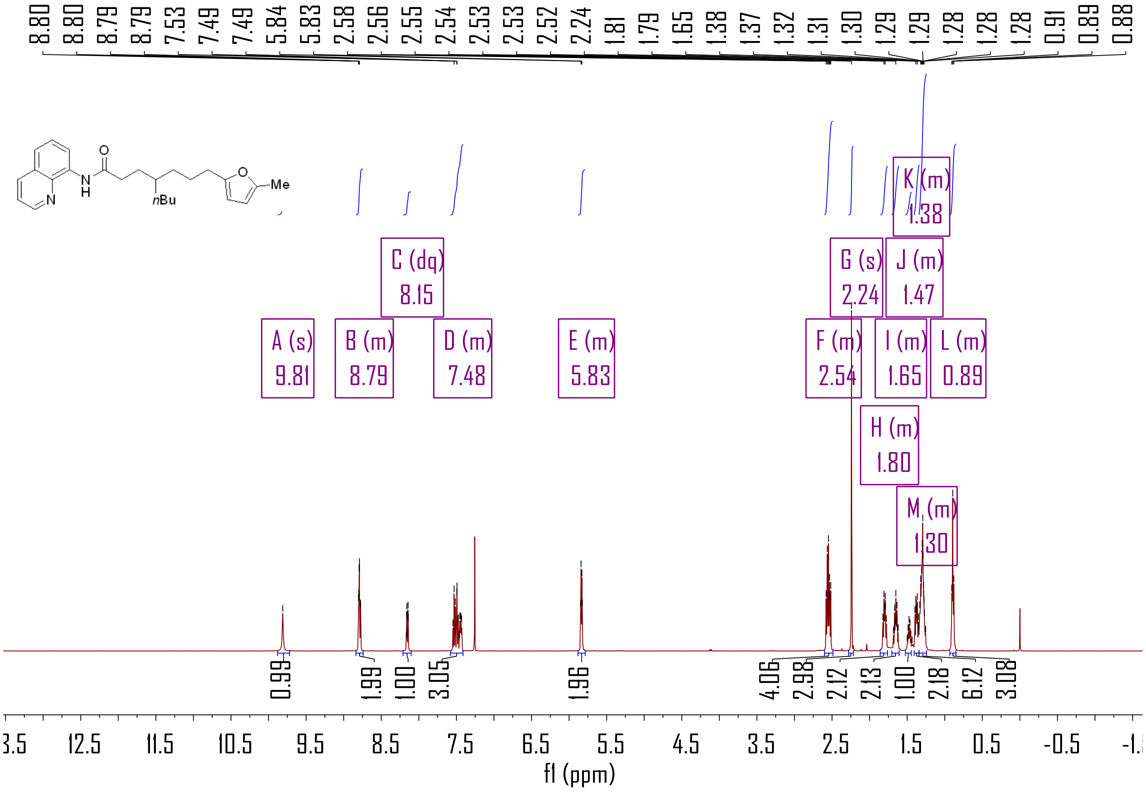

Supplementary Figure 171. ^{1}H NMR spectra of **4ja**

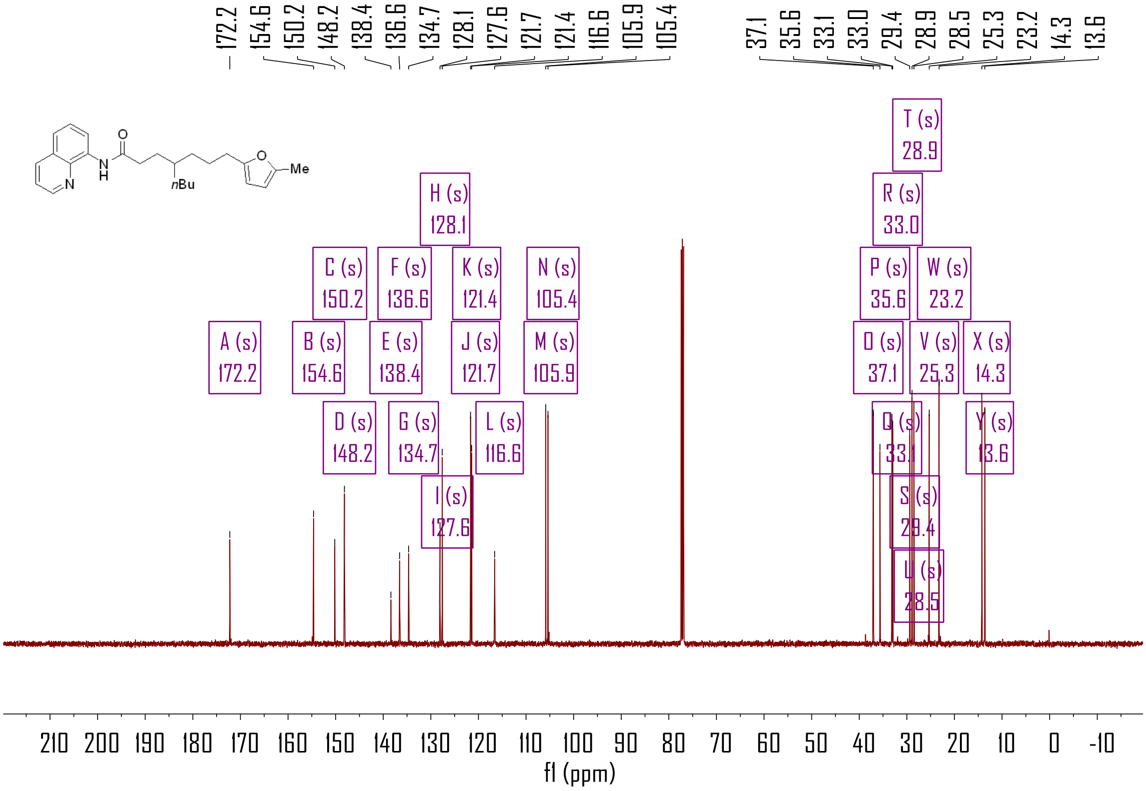

Supplementary Figure 172. ^{13}C NMR spectra of **4ja**

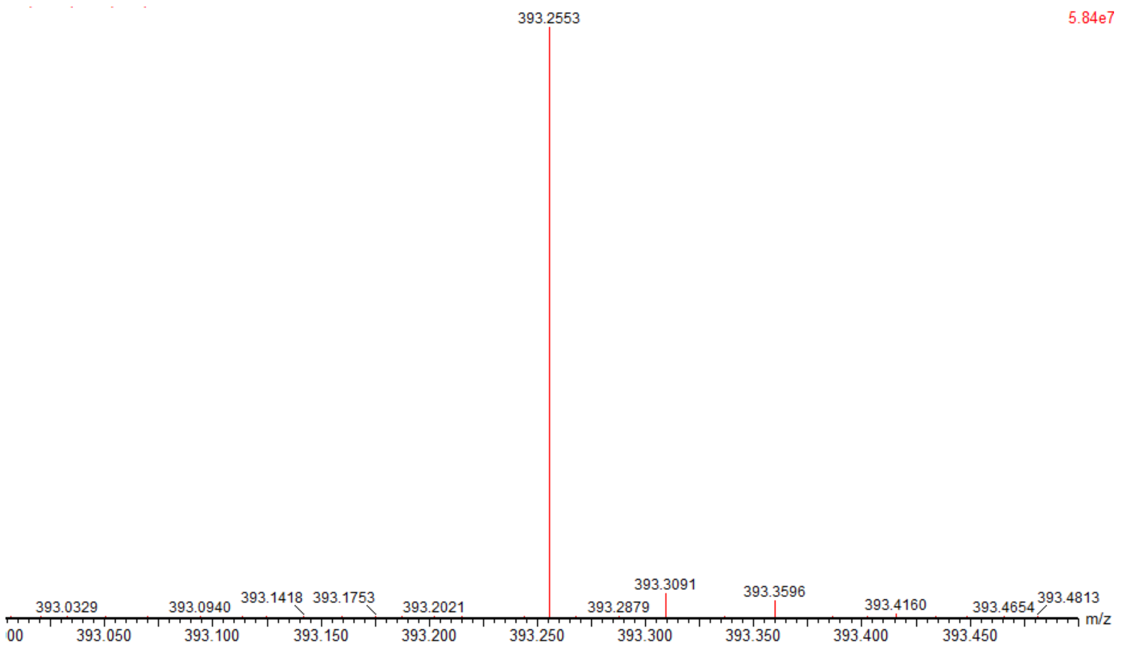

Supplementary Figure 173. HRMS spectra of **4ja**

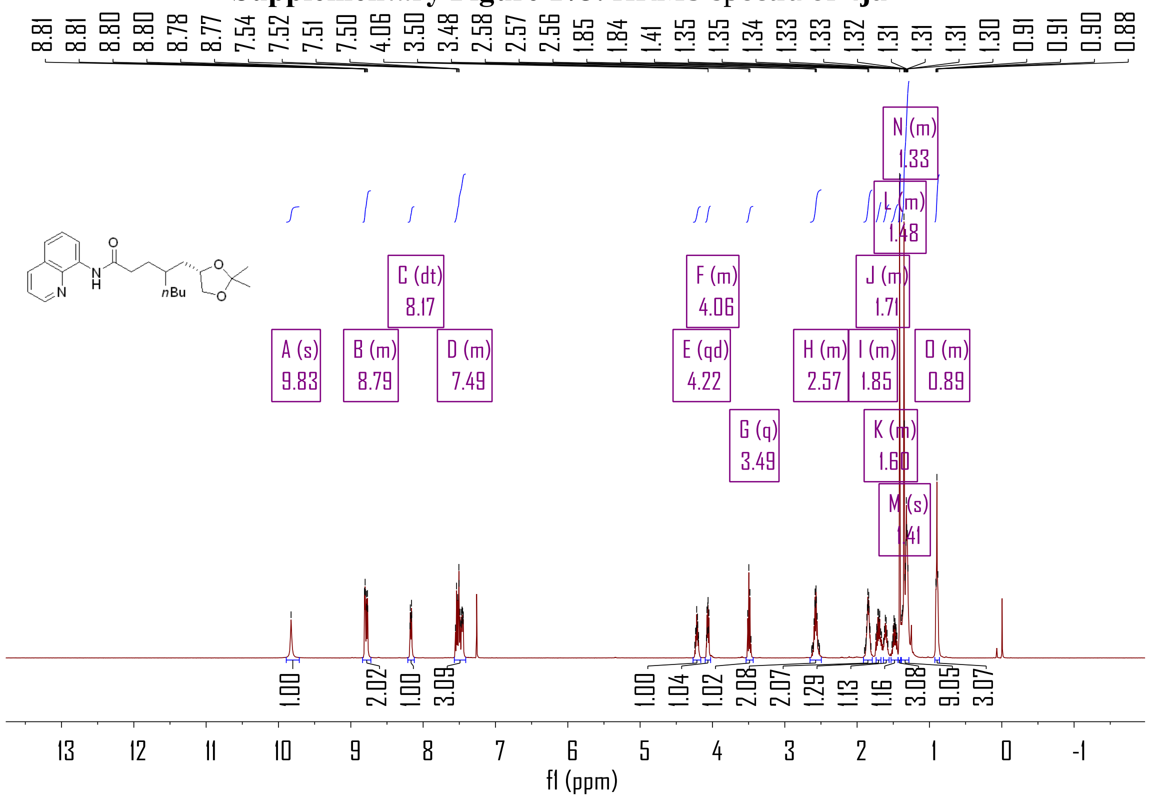

Supplementary Figure 174. ^{1}H NMR spectra of **4ka**

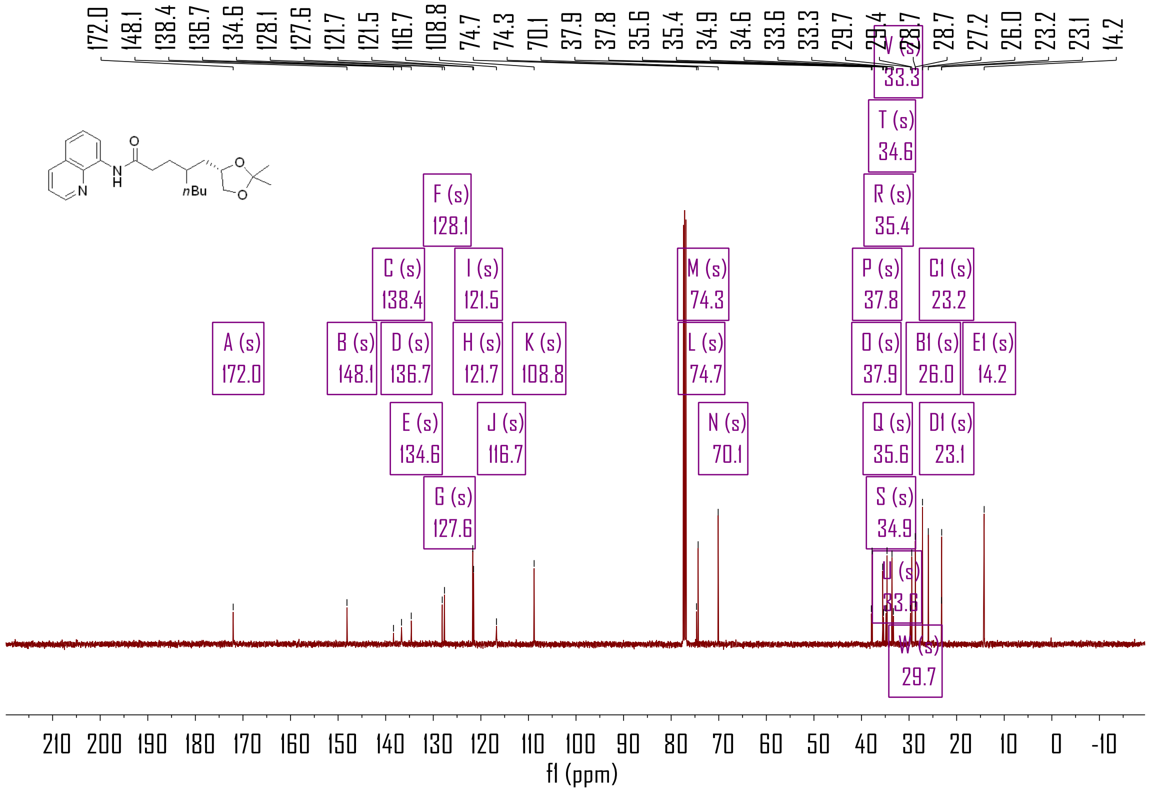

Supplementary Figure 175. ^{13}C NMR spectra of **4ka**

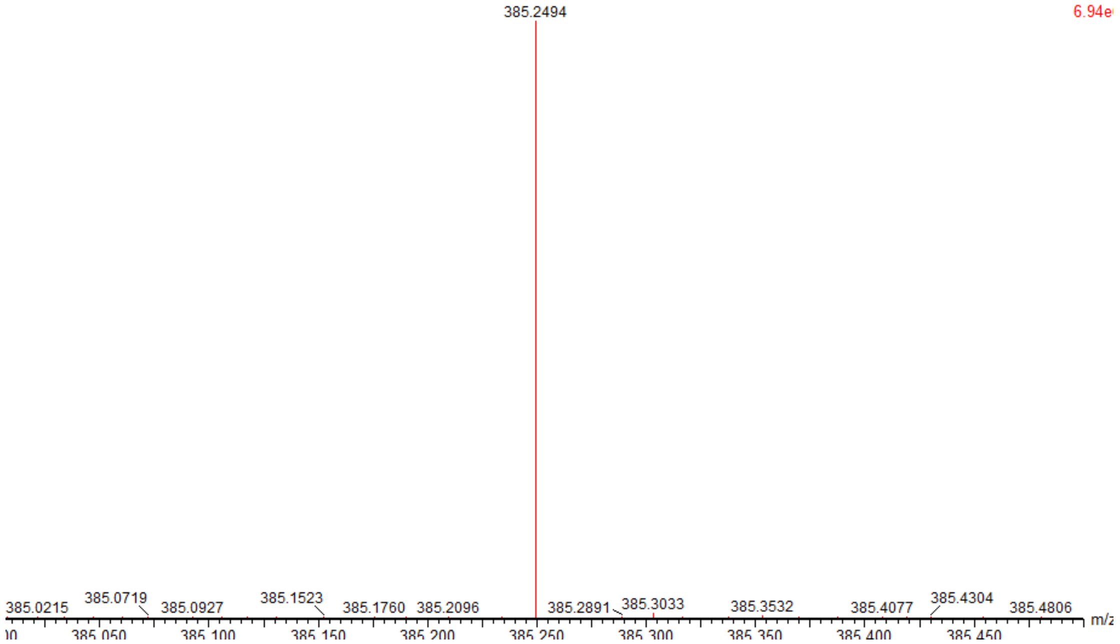

Supplementary Figure 176. HRMS spectra of **4ka**

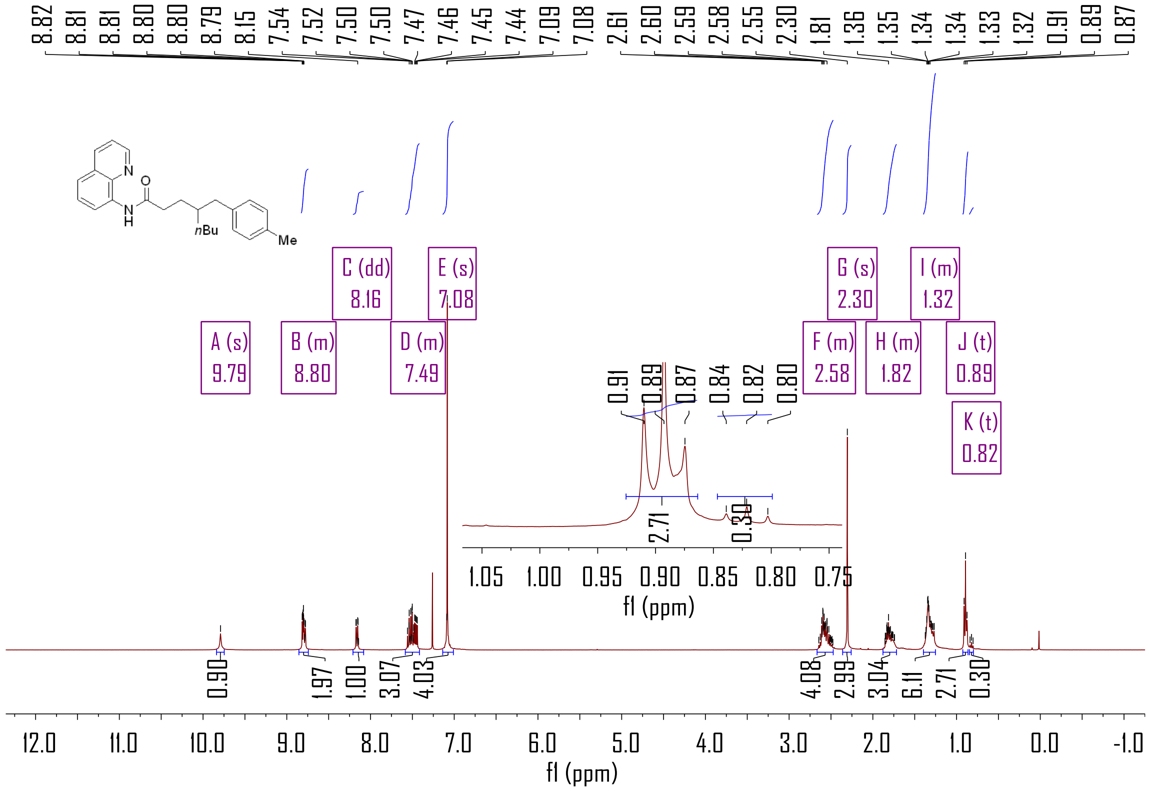

Supplementary Figure 177. ^{1}H NMR spectra of **4la**

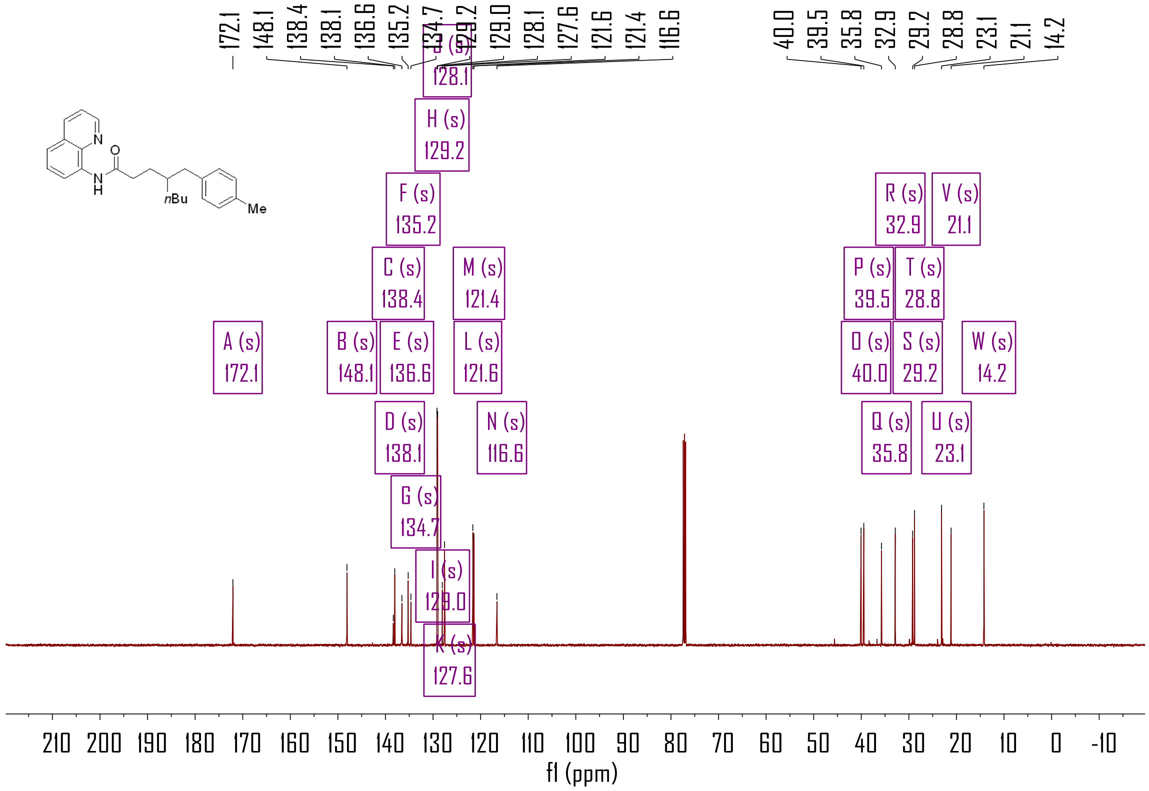

Supplementary Figure 178. ^{13}C NMR spectra of **4la**

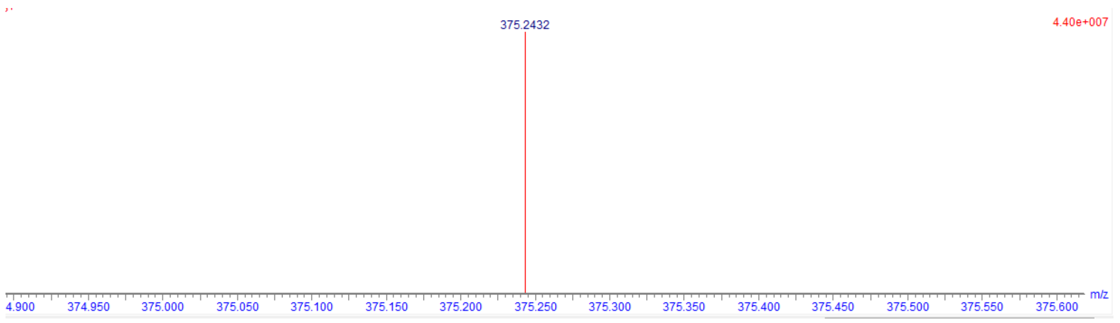

Supplementary Figure 179. HRMS spectra of **4la**

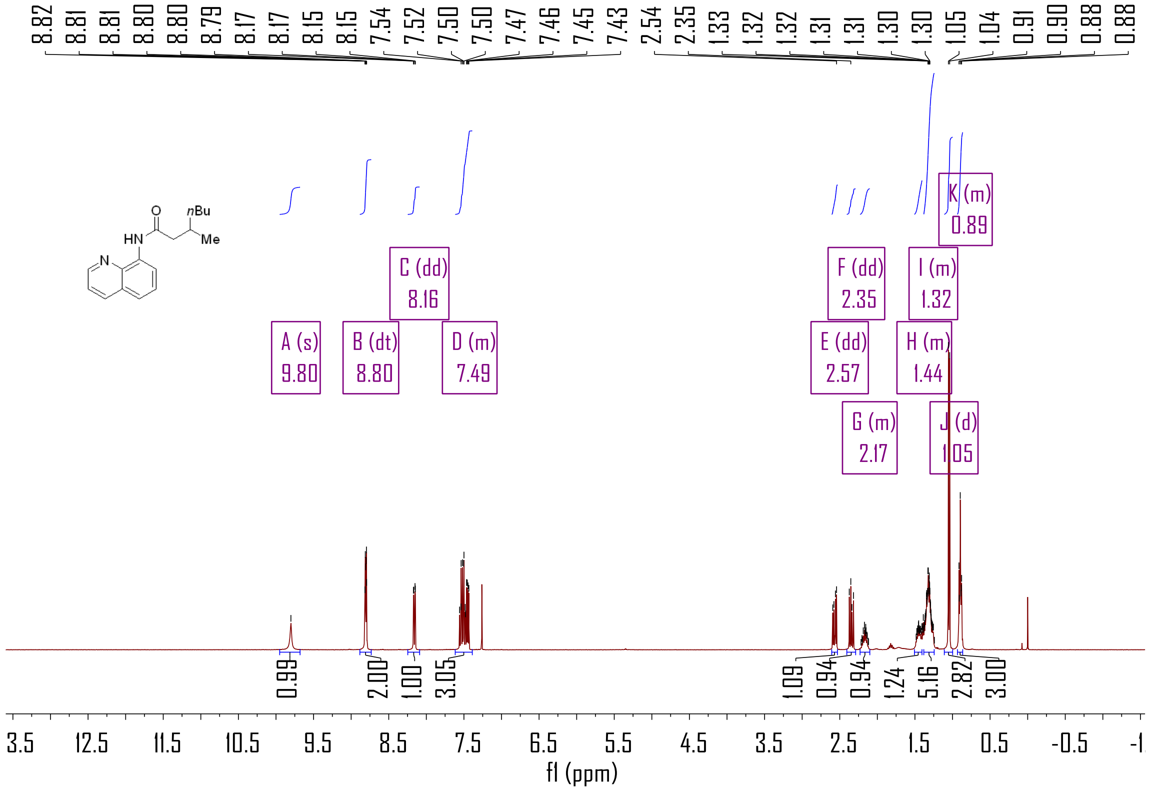

Supplementary Figure 180. ^{1}H NMR spectra of **4ma**

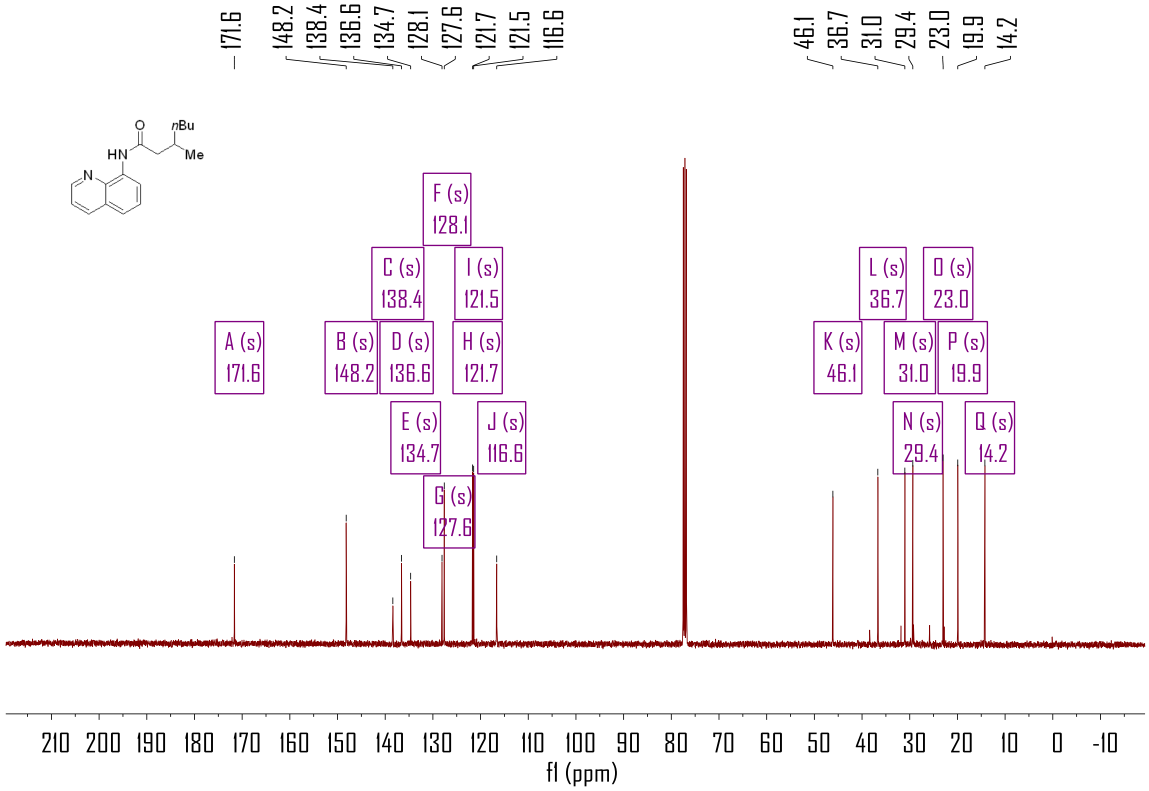

Supplementary Figure 181. ^{13}C NMR spectra of **4ma**

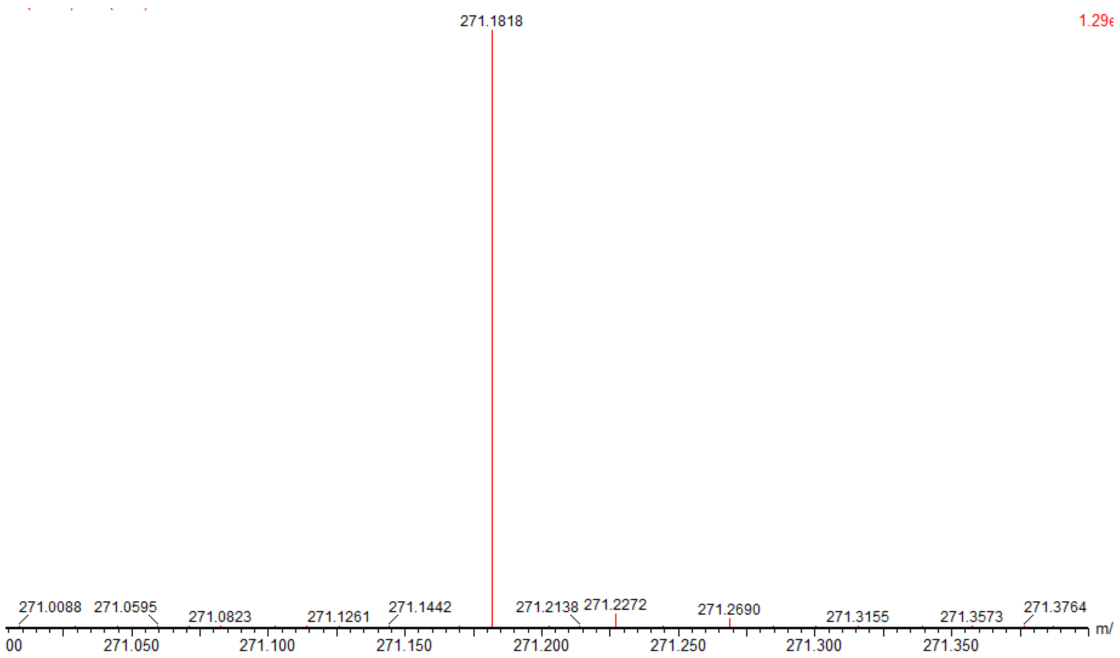

Supplementary Figure 182. HRMS spectra of **4ma**

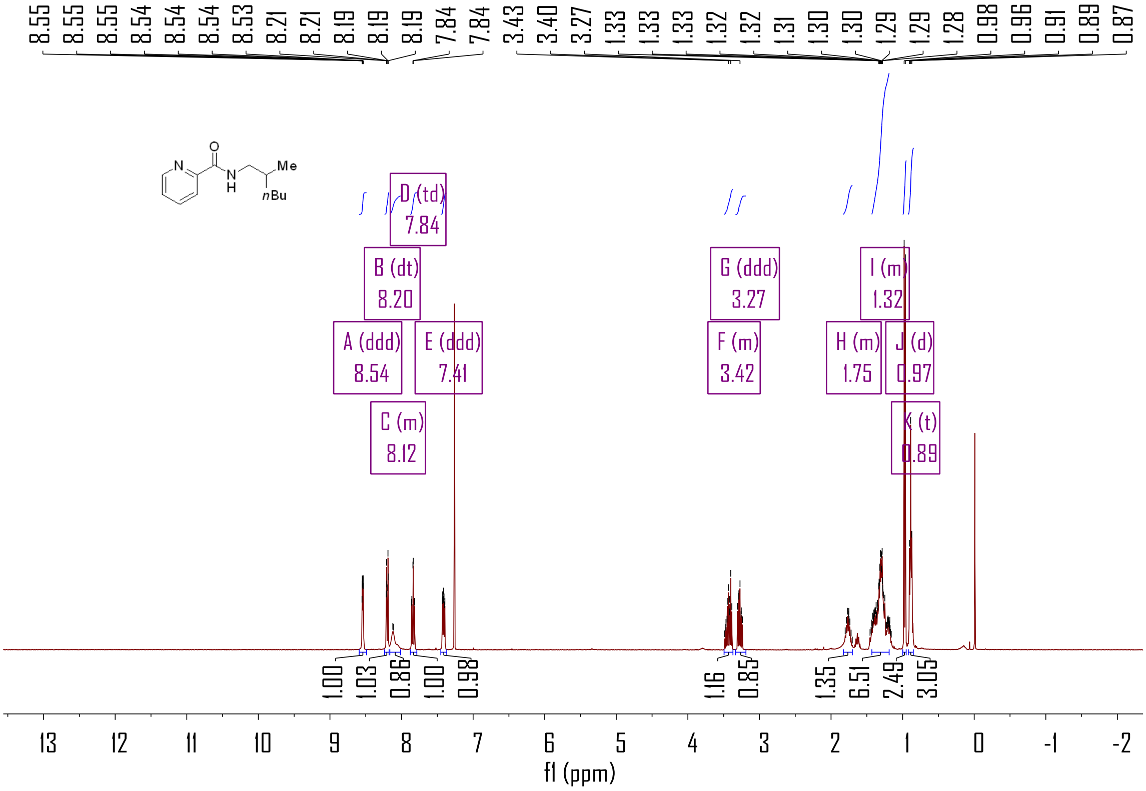

Supplementary Figure 183. ^{1}H NMR spectra of **4na**

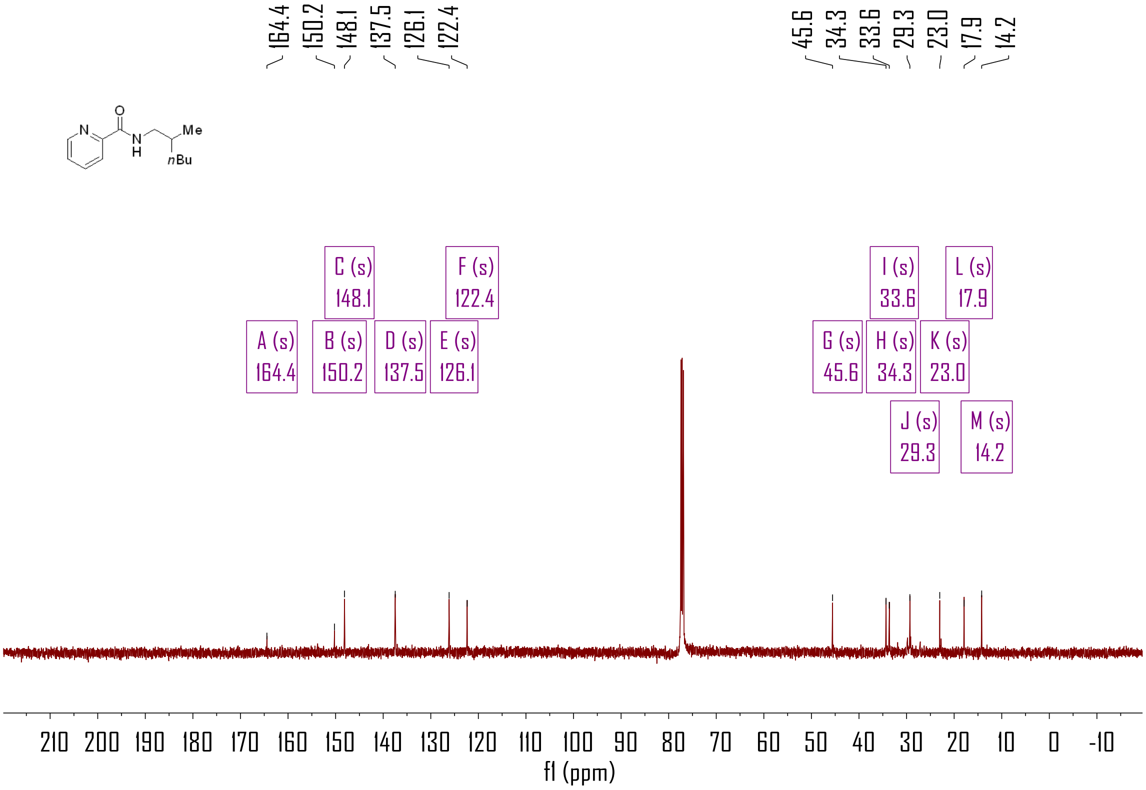

Supplementary Figure 184. ^{13}C NMR spectra of **4na**

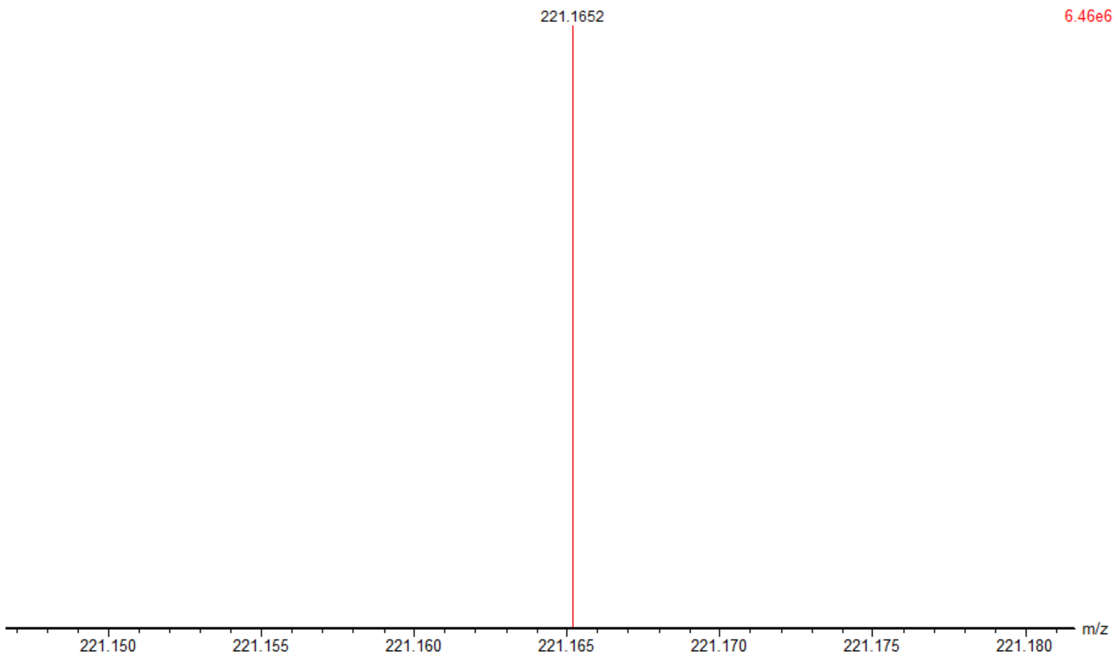

Supplementary Figure 185. HRMS spectra of **4na**

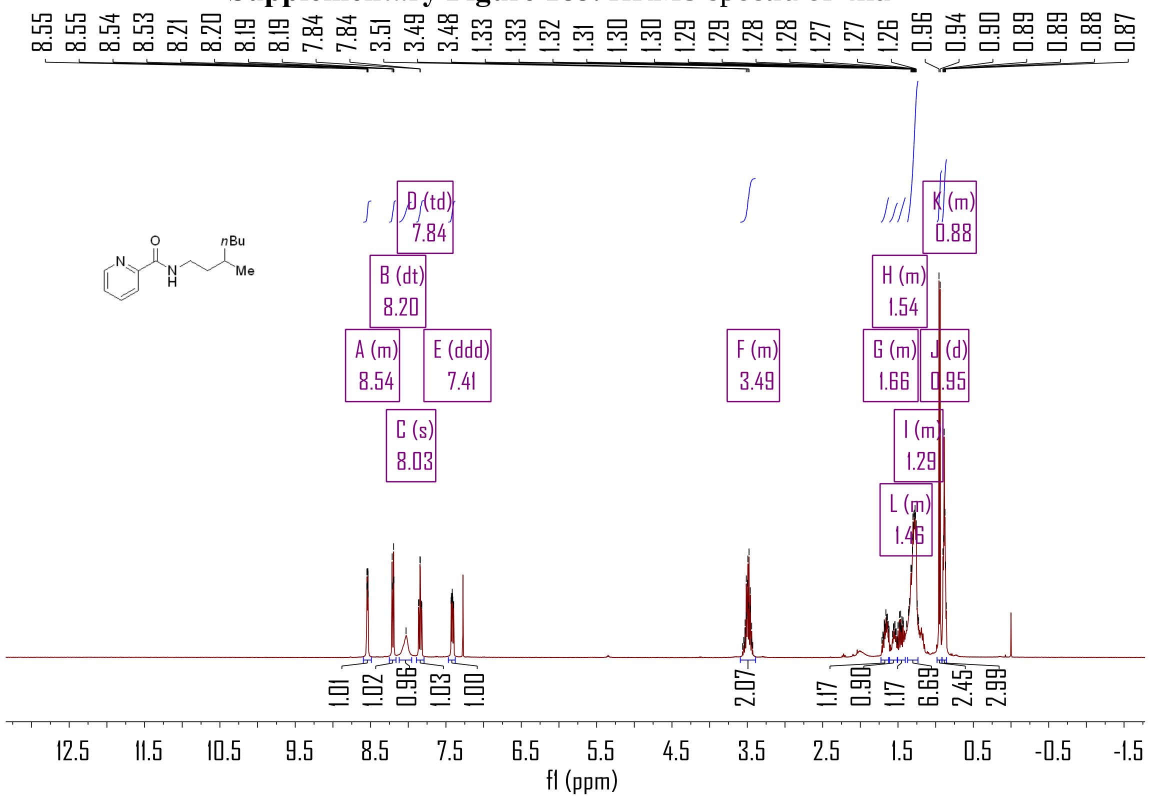

Supplementary Figure 186. ^{1}H NMR spectra of **4oa**

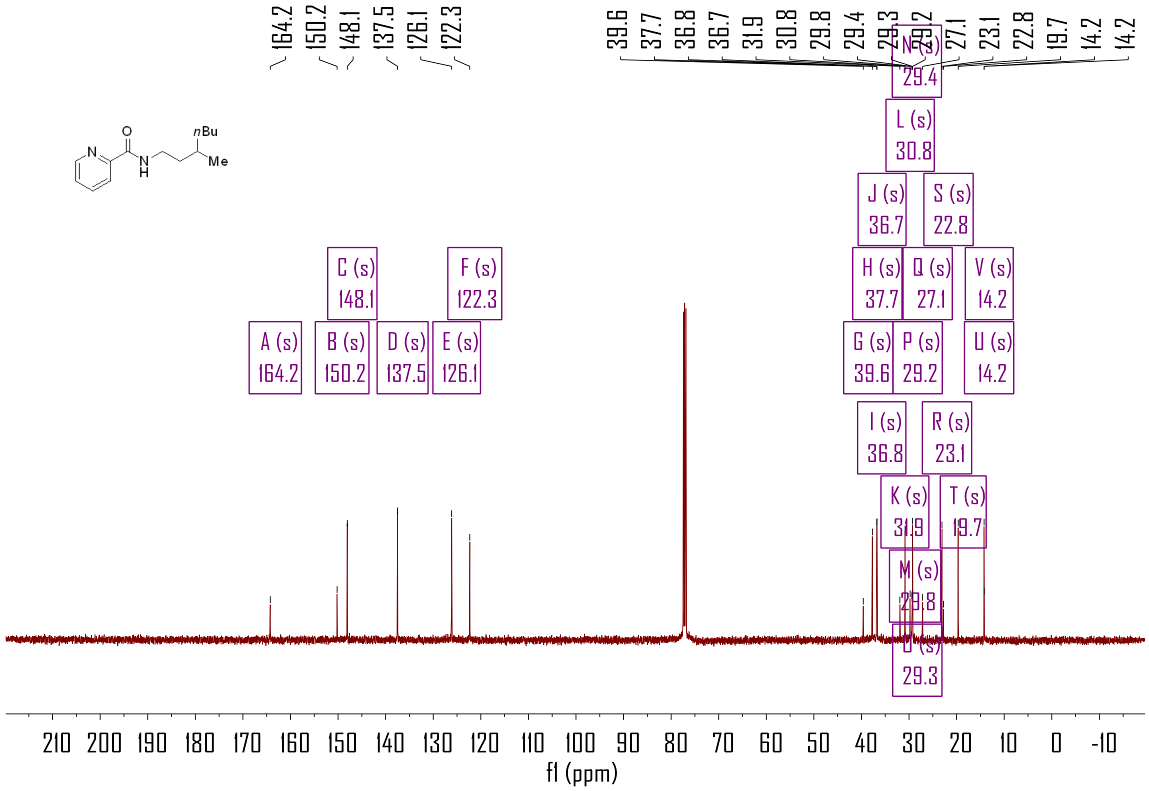

Supplementary Figure 187. ^{13}C NMR spectra of **4oa**

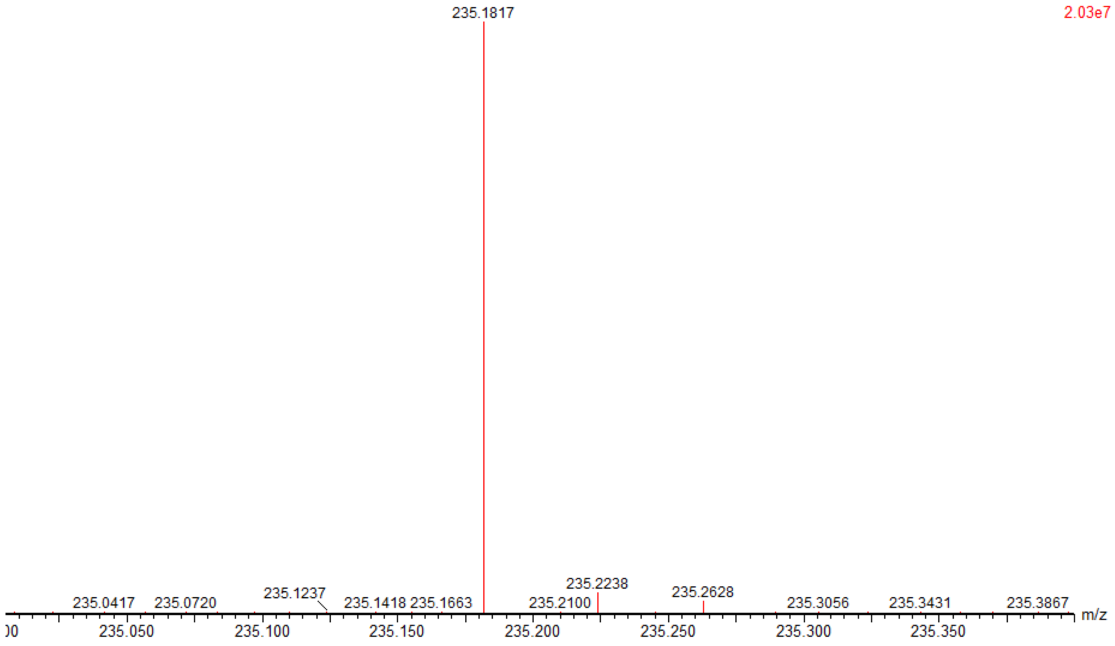

Supplementary Figure 188. HRMS spectra of **4oa**

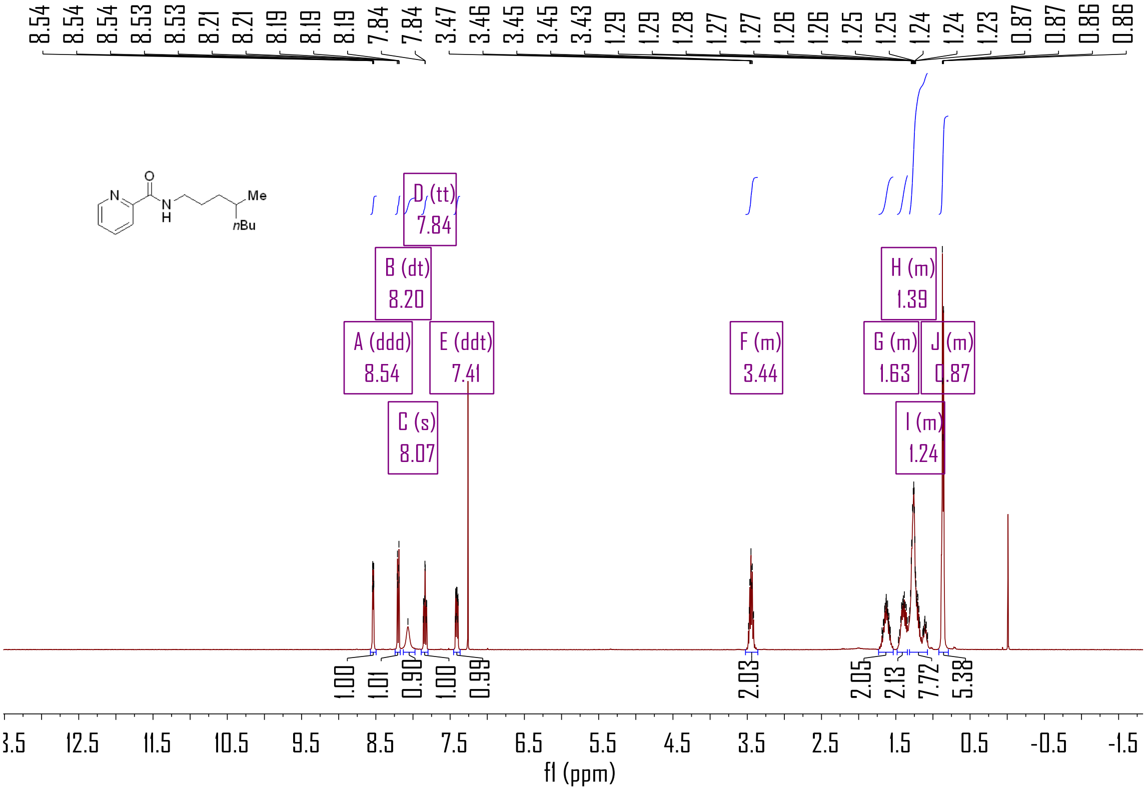

Supplementary Figure 189. ^{1}H NMR spectra of **4pa**

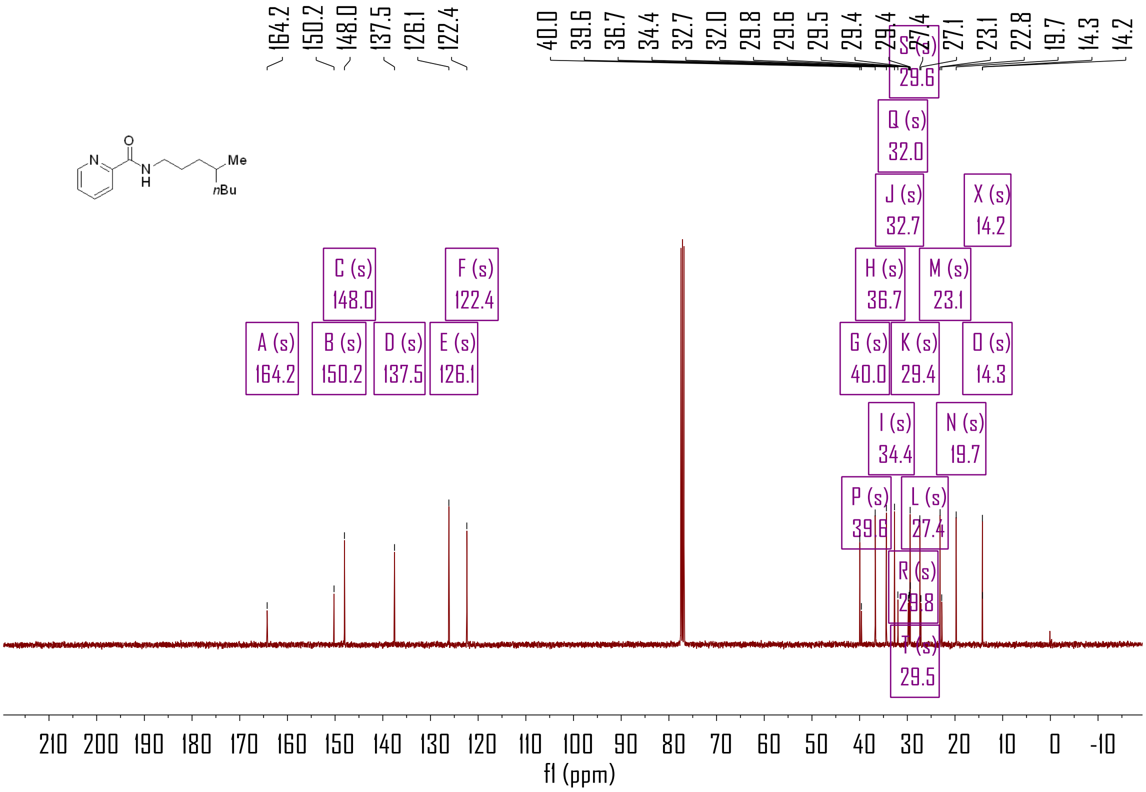

Supplementary Figure 190. ^{13}C NMR spectra of **4pa**

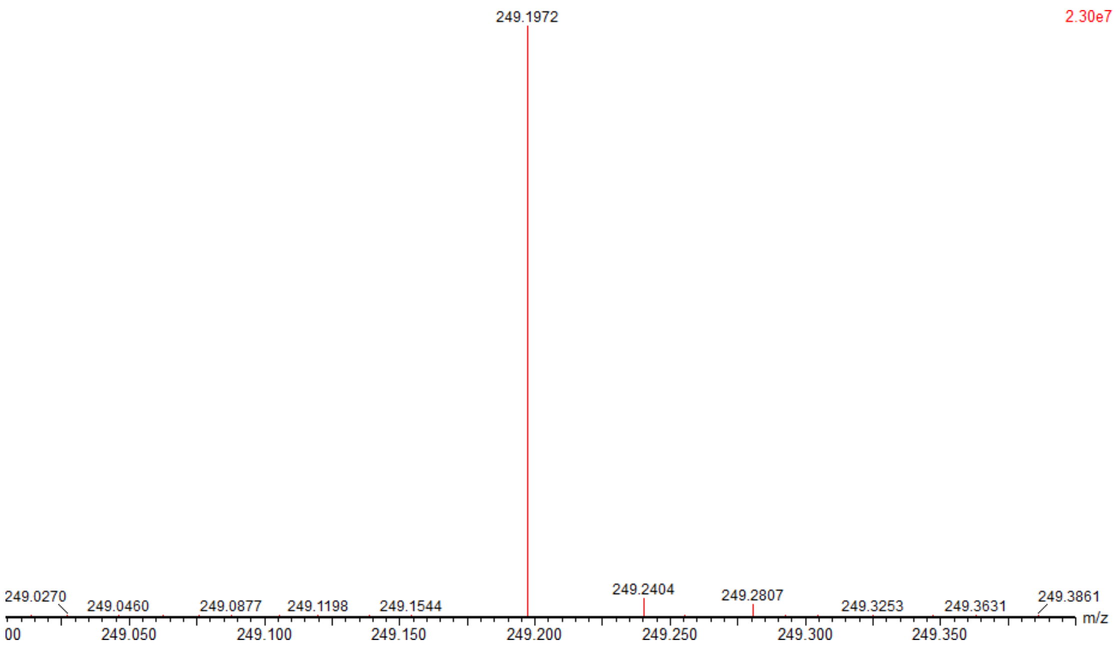

Supplementary Figure 191. HRMS spectra of **4pa**

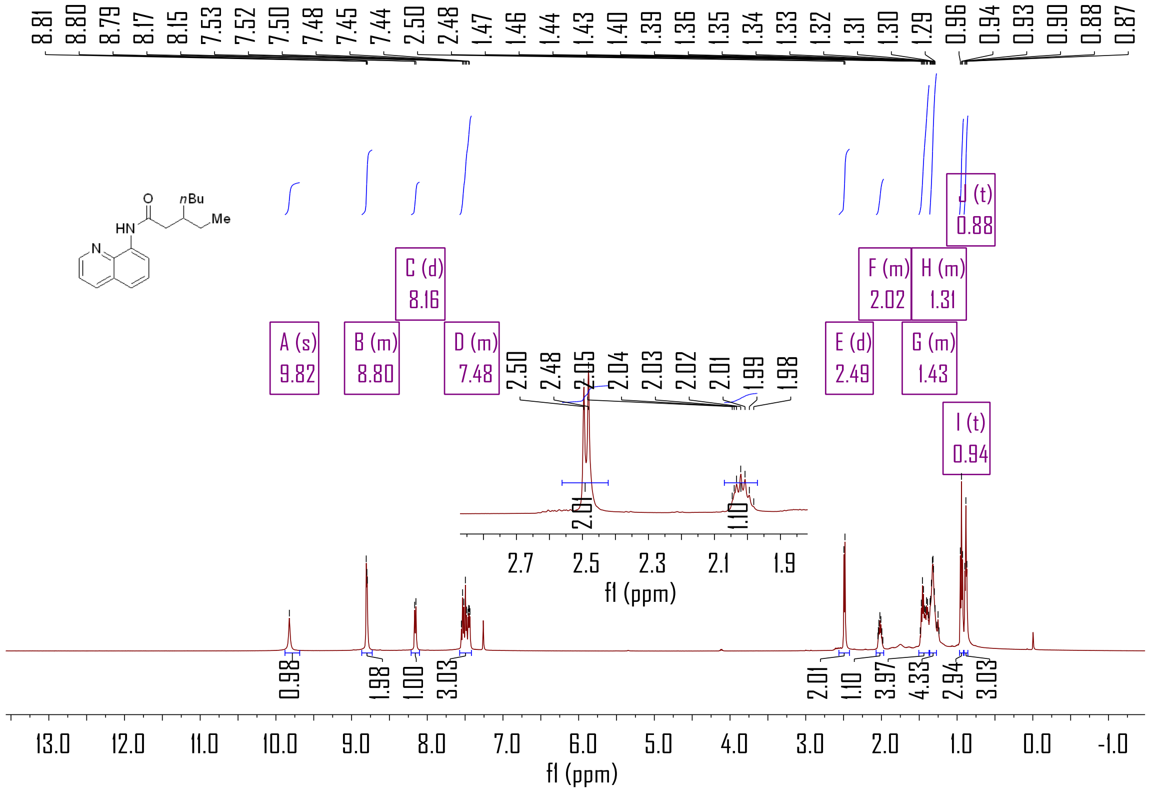

Supplementary Figure 192. ^{1}H NMR spectra of **6aa**

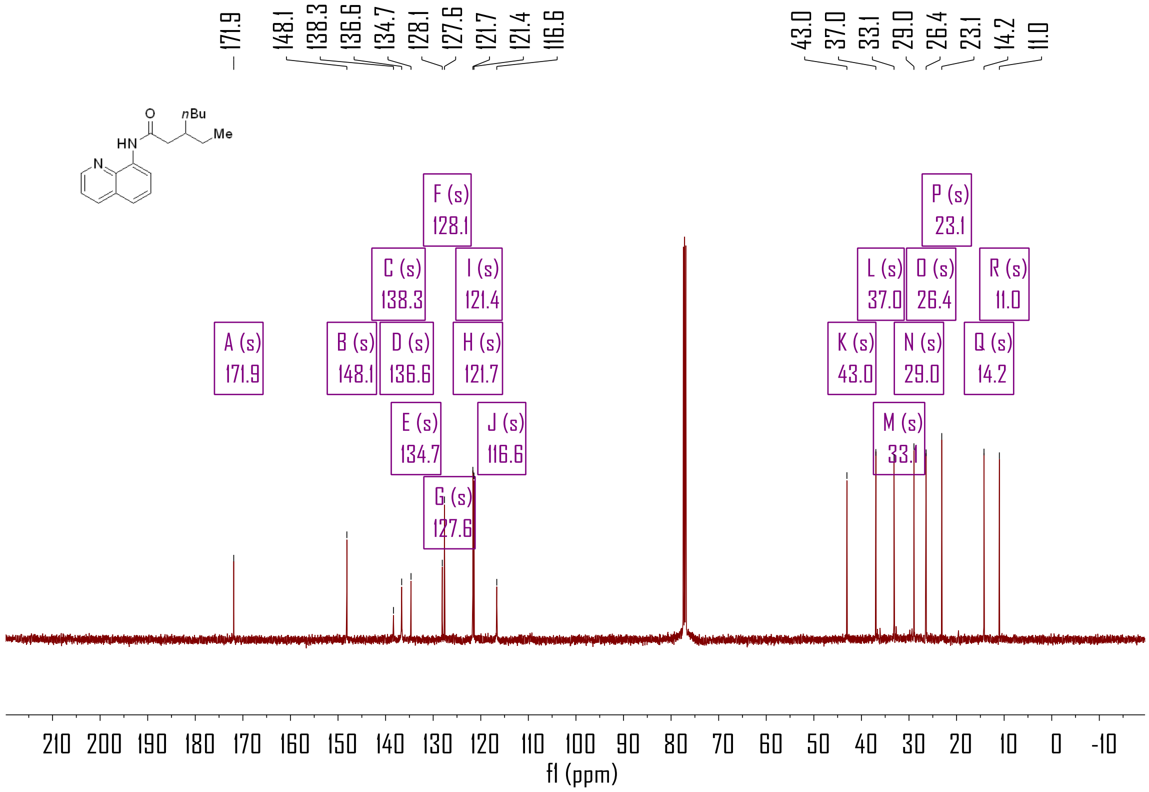

Supplementary Figure 193. ^{13}C NMR spectra of **6aa**

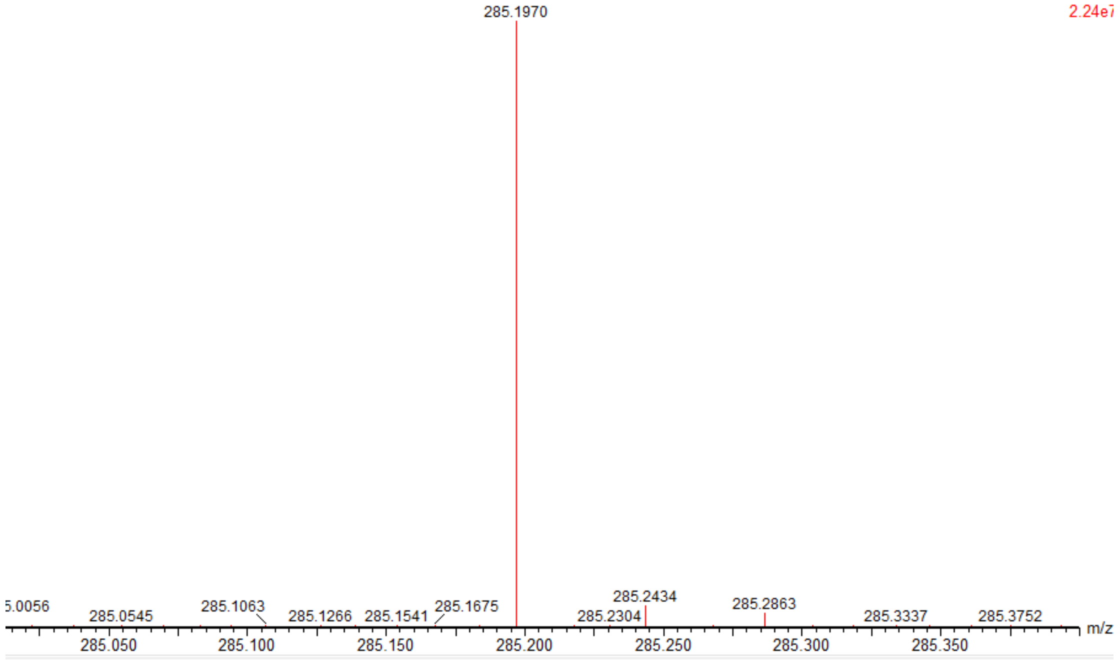

Supplementary Figure 194. HRMS spectra of **6aa**

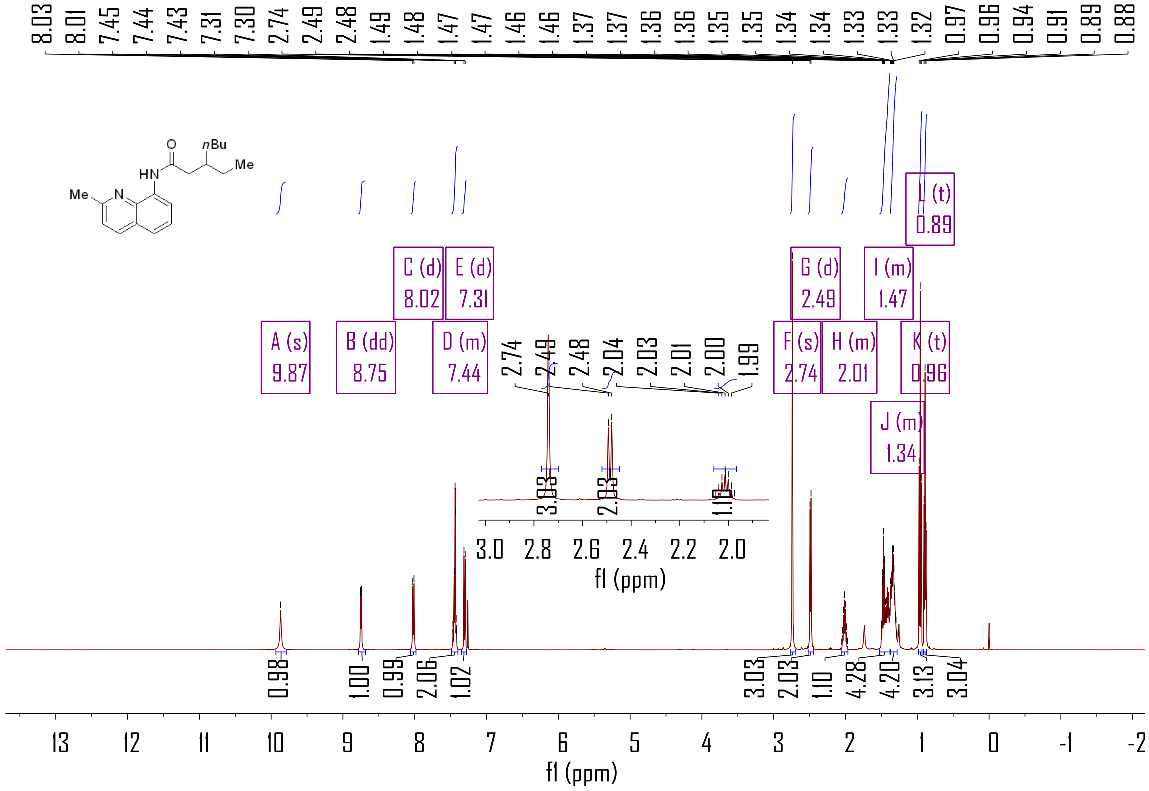

Supplementary Figure 195. ^{1}H NMR spectra of **6ra**

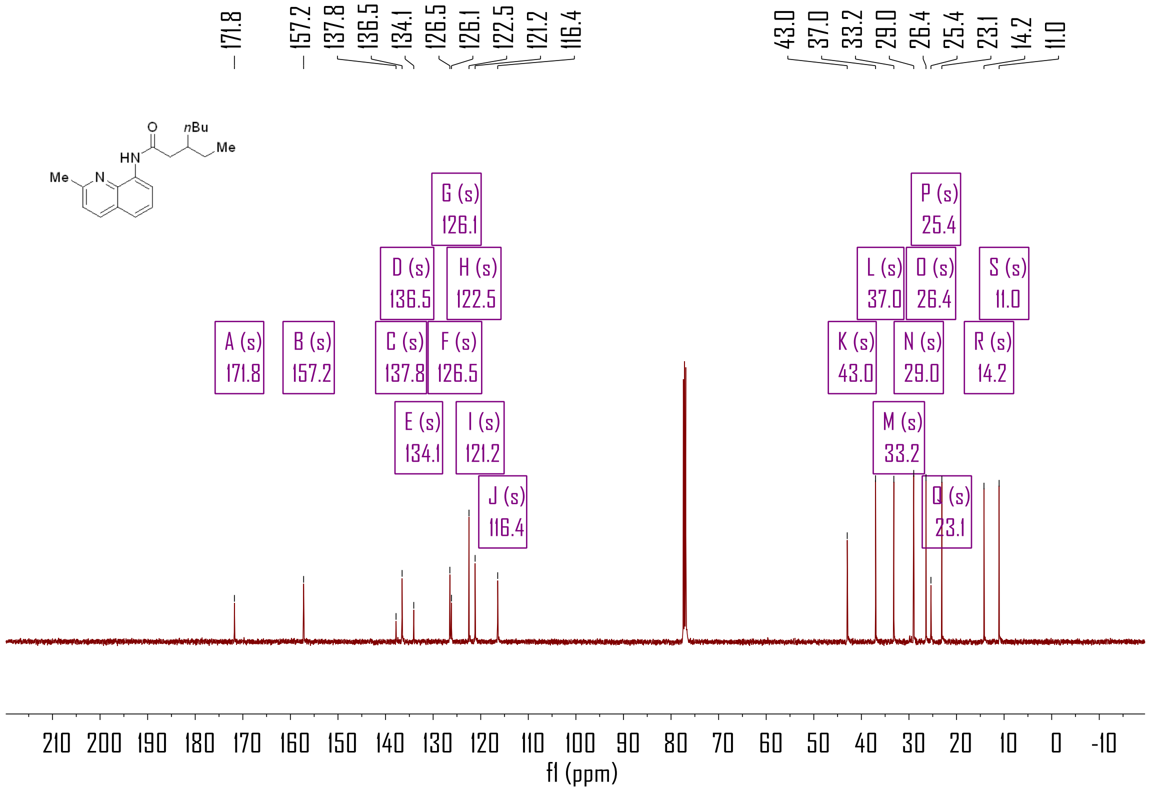

Supplementary Figure 196. ^{13}C NMR spectra of **6ra**

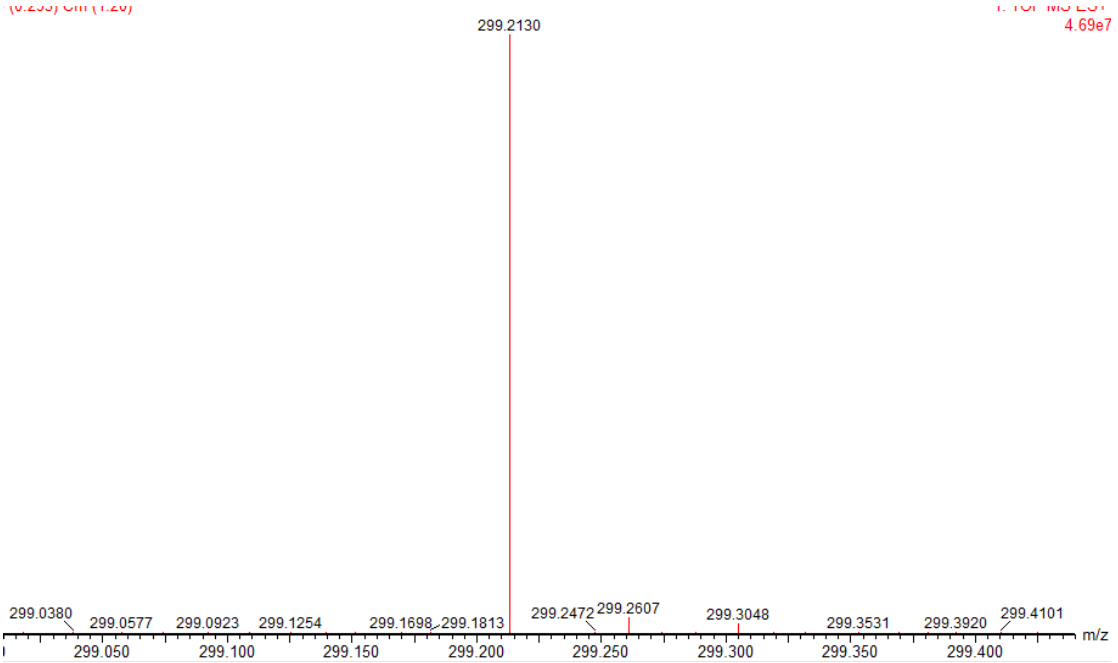

Supplementary Figure 197. HRMS spectra of **6ra**

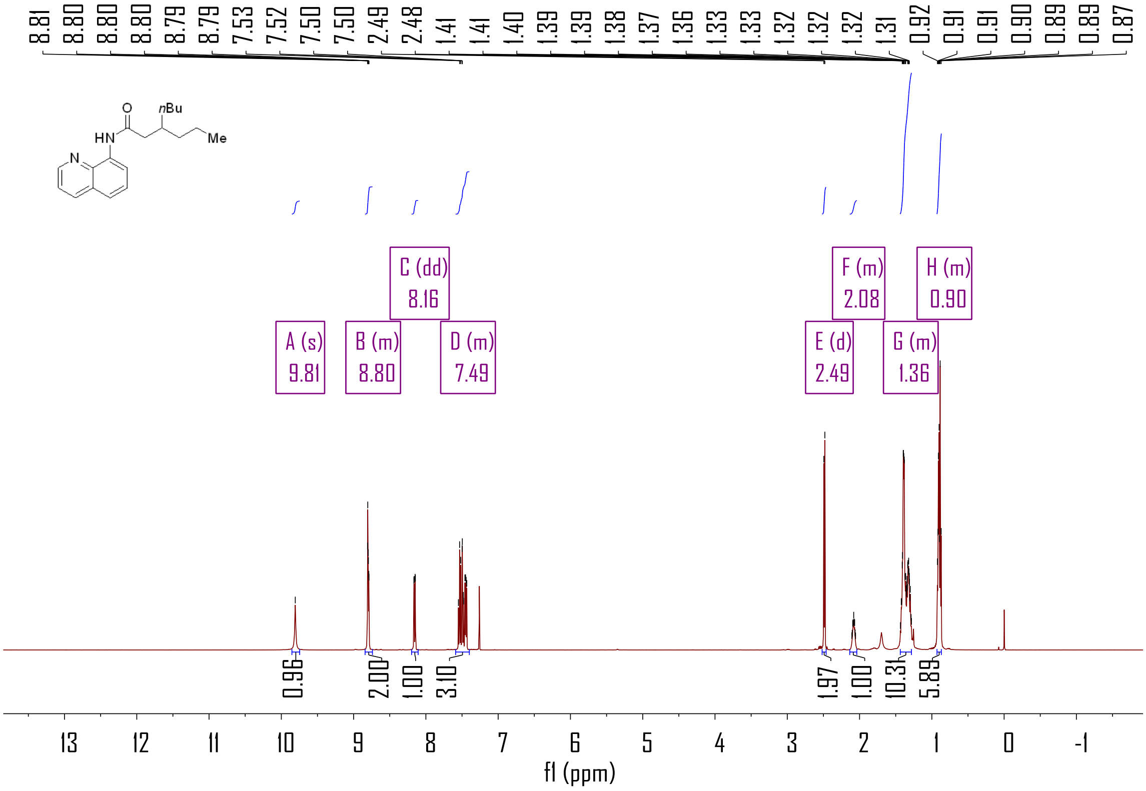

Supplementary Figure 198. ^{1}H NMR spectra of **6qa**

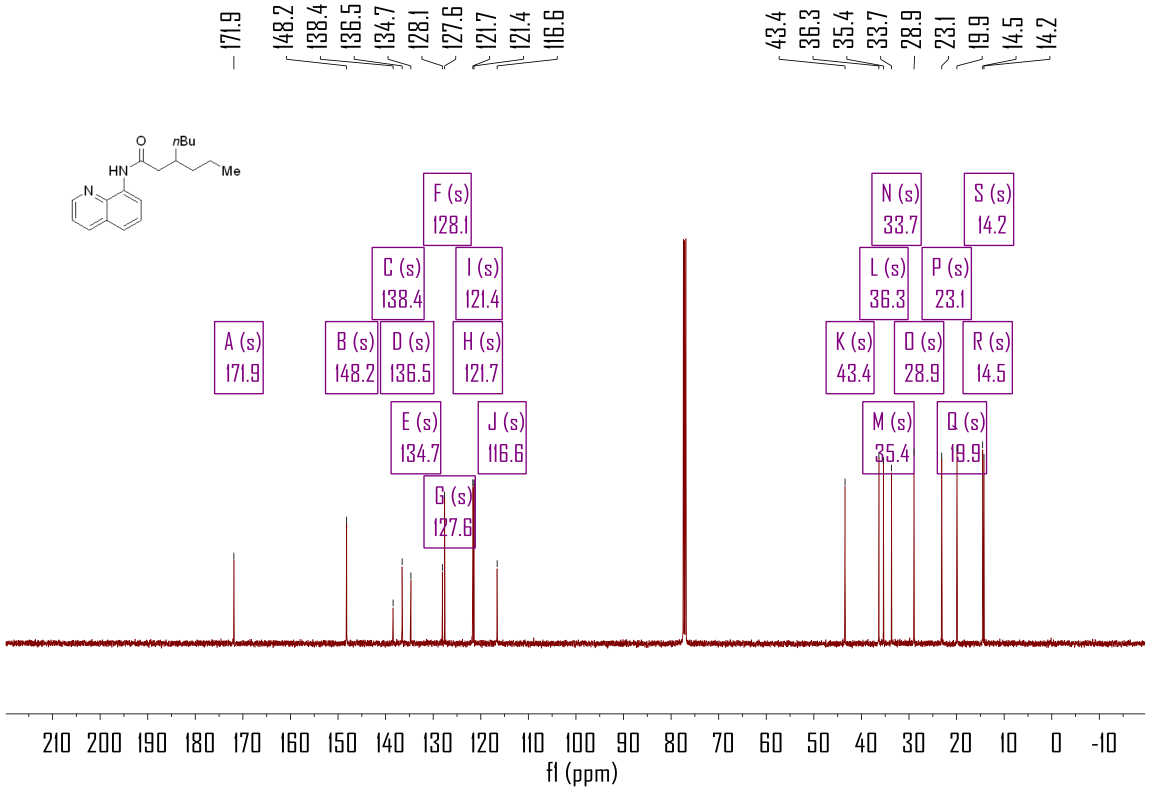

Supplementary Figure 199. ^{13}C NMR spectra of **6qa**

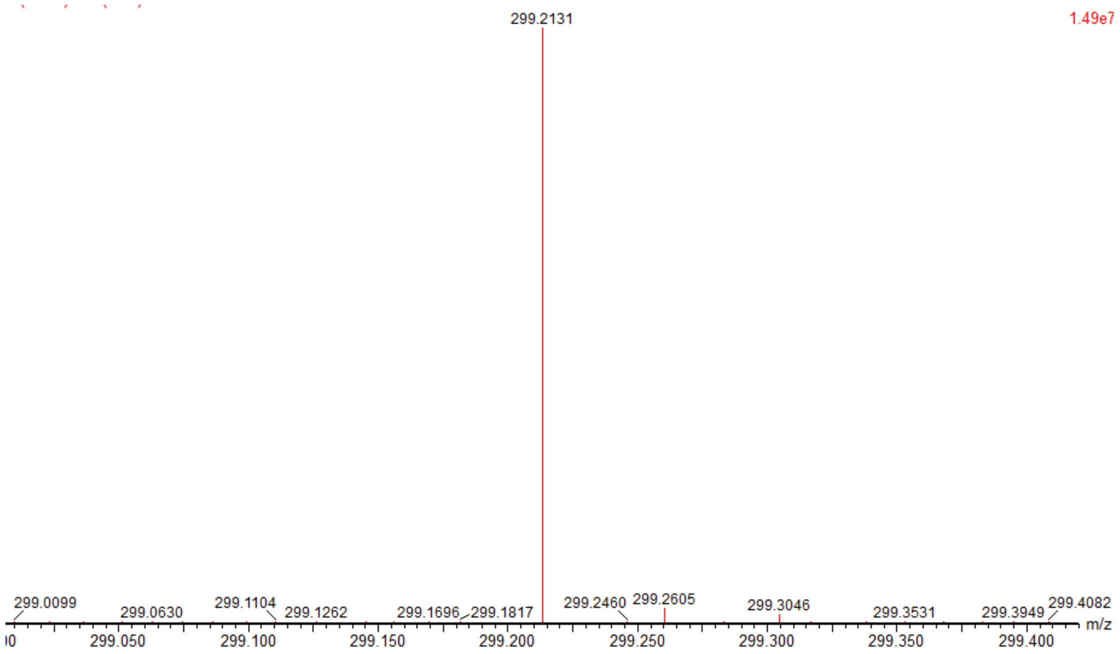

Supplementary Figure 200. HRMS spectra of **6qa**

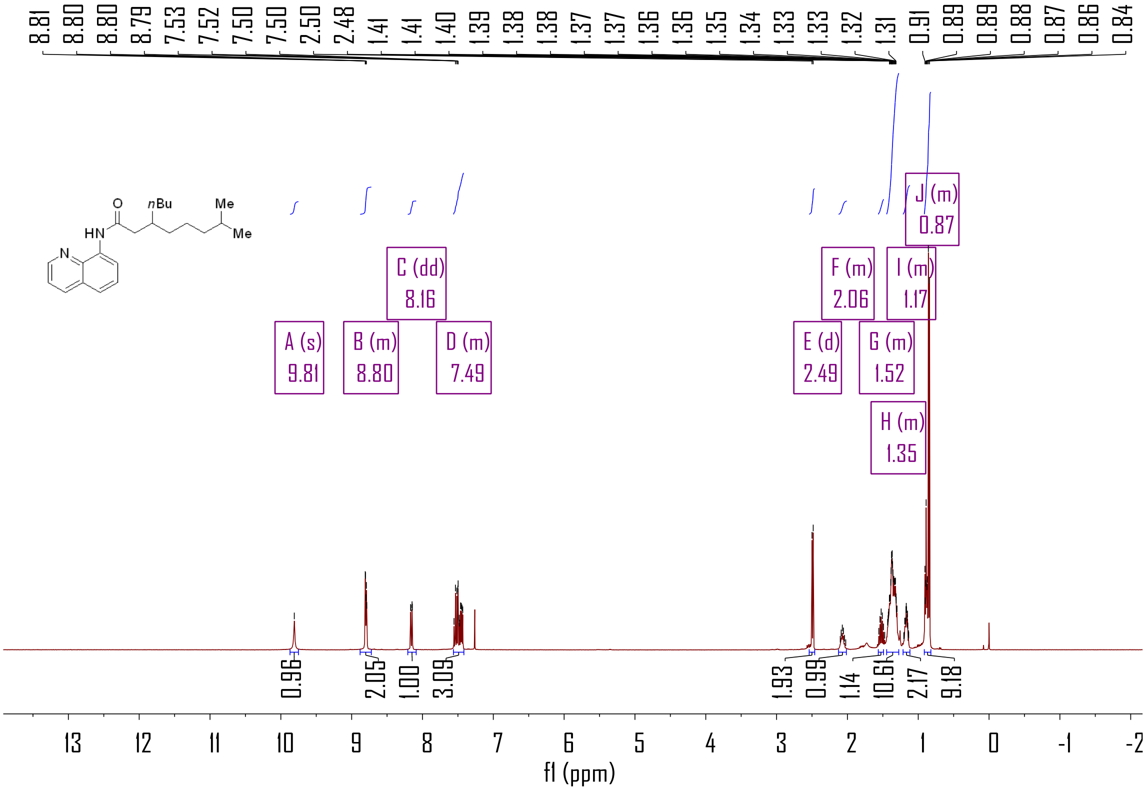

Supplementary Figure 201. ^{1}H NMR spectra of **6ca**

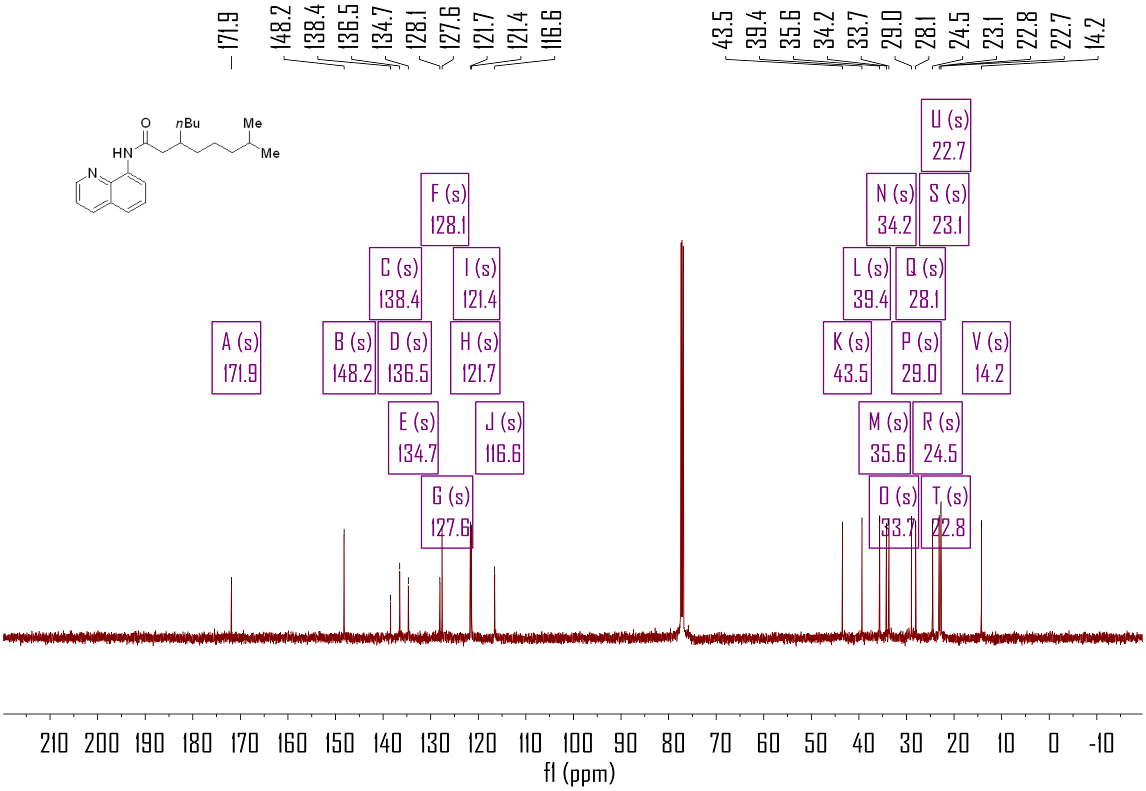

Supplementary Figure 202. ^{13}C NMR spectra of **6ca**

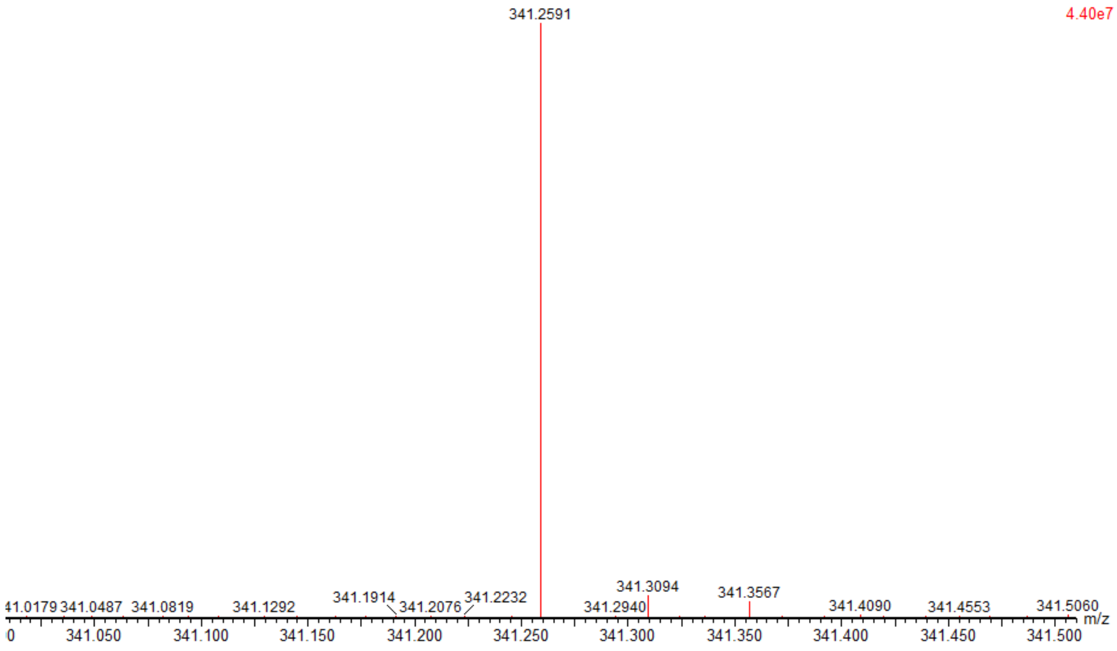

Supplementary Figure 203. HRMS spectra of **6ca**

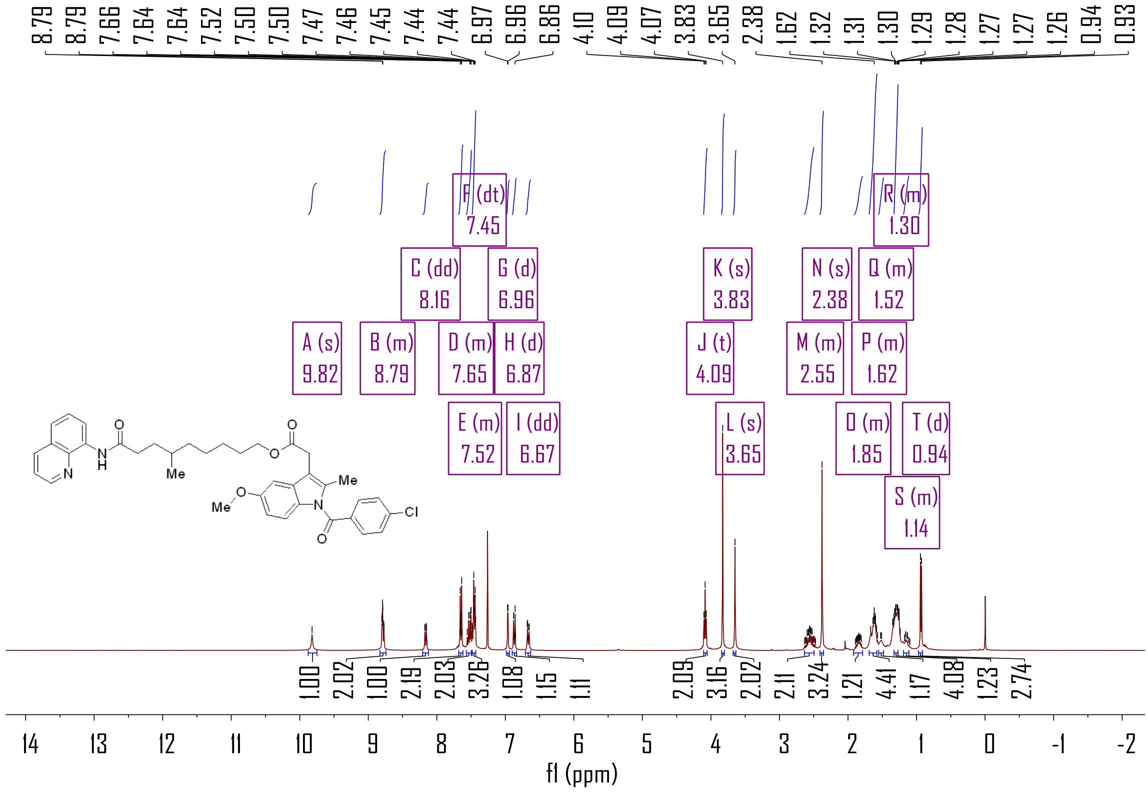

Supplementary Figure 204. ^{1}H NMR spectra of **4au**

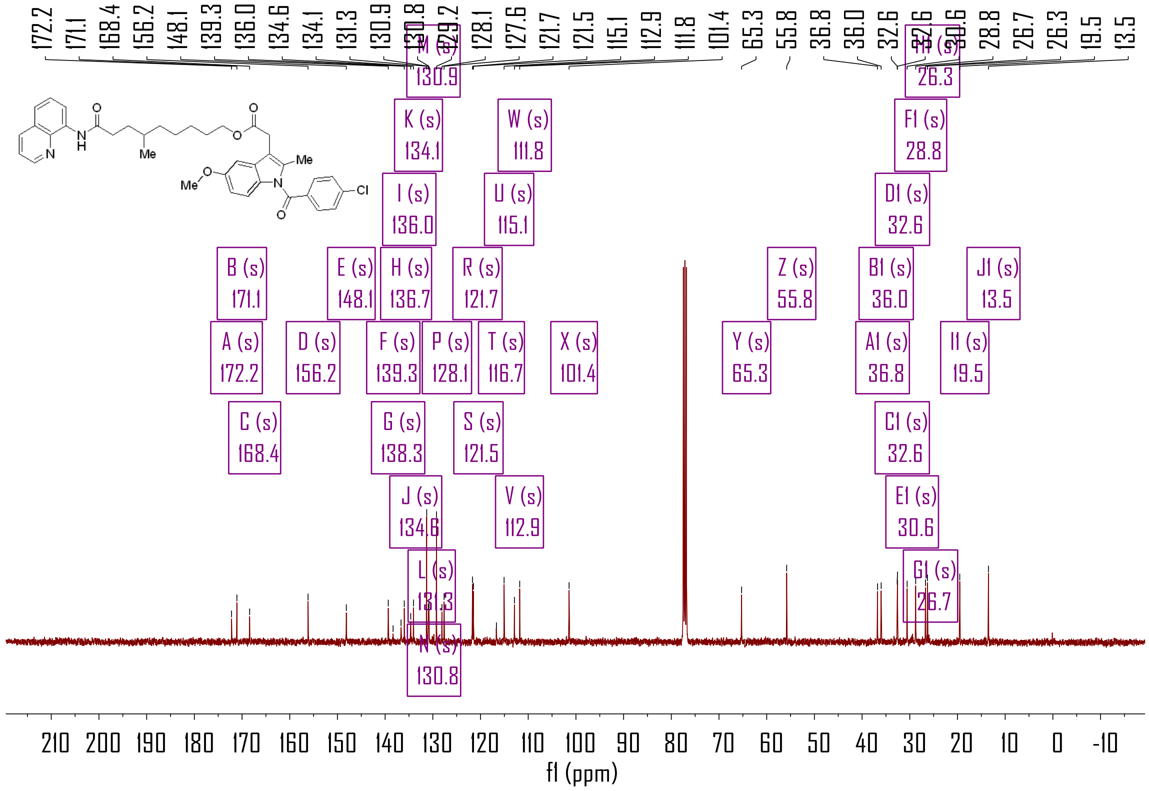

Supplementary Figure 205. ^{13}C NMR spectra of **4au**

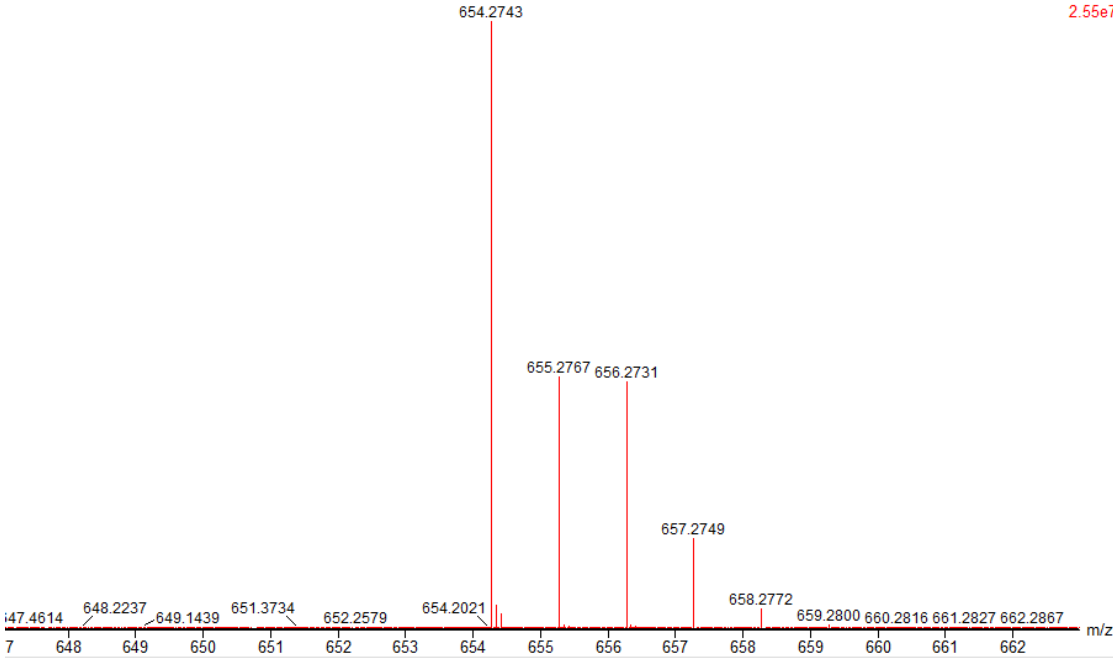

Supplementary Figure 206. HRMS spectra of **4au**

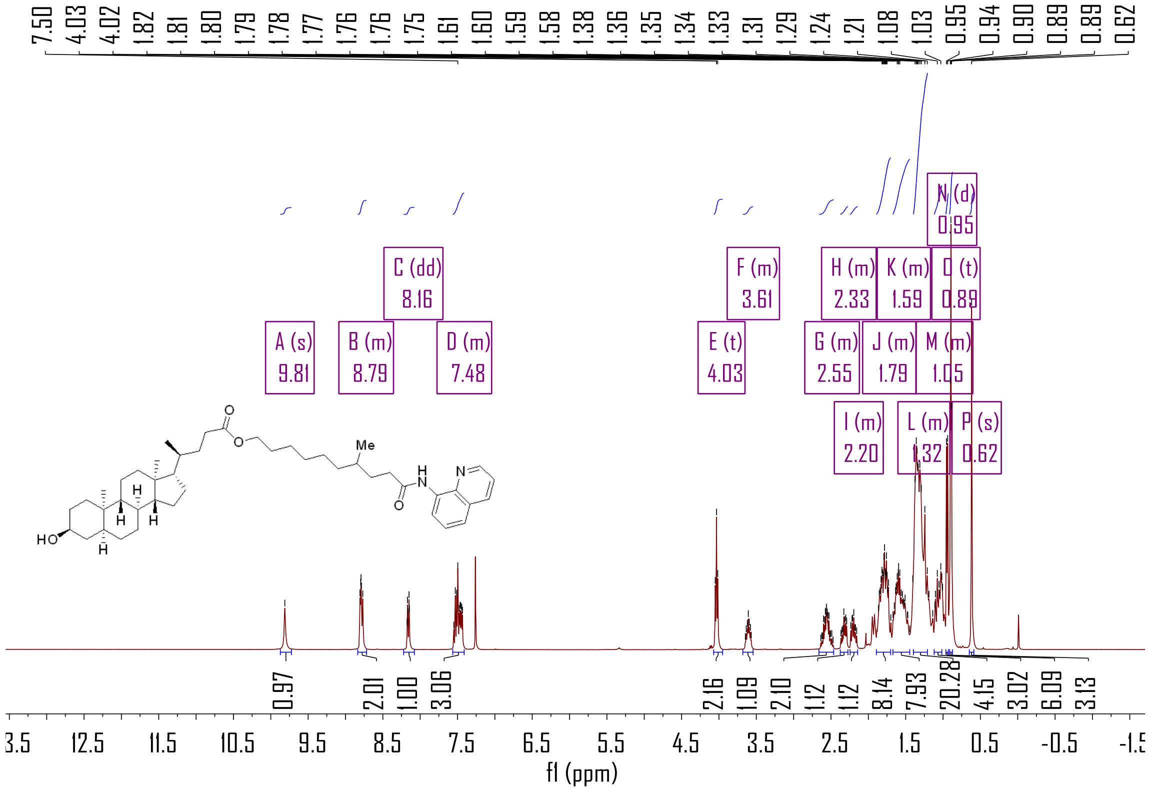

Supplementary Figure 207. ^{1}H NMR spectra of **4av**

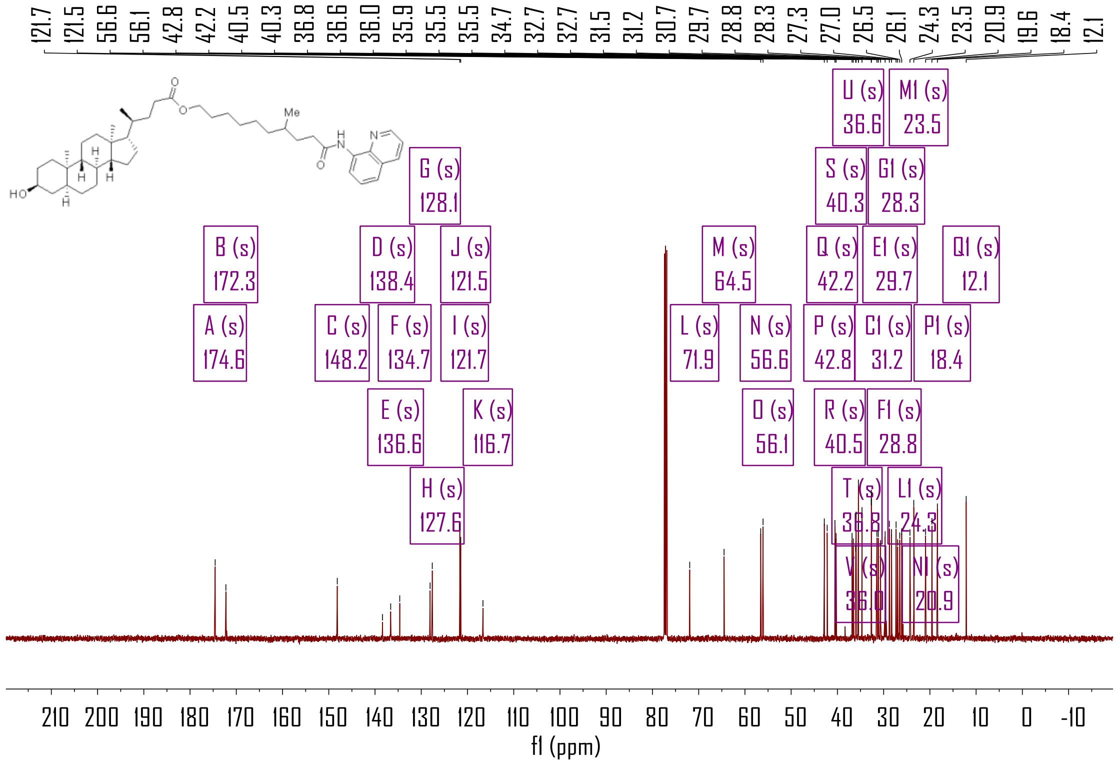

Supplementary Figure 208. ^{13}C NMR spectra of **4av**

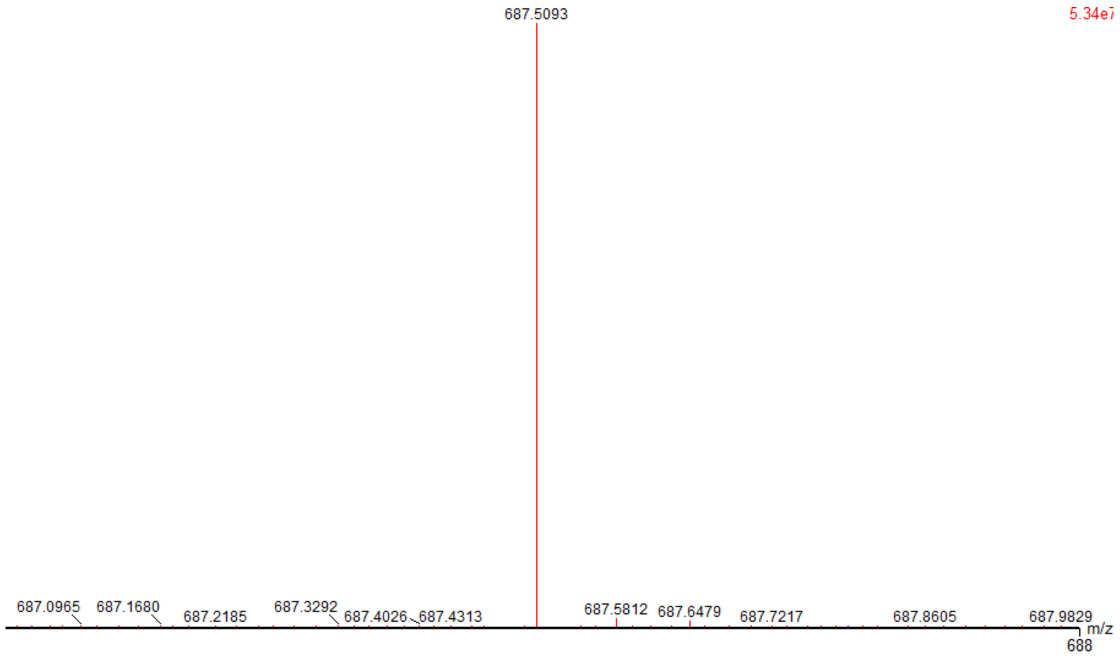

Supplementary Figure 209. HRMS spectra of **4av**

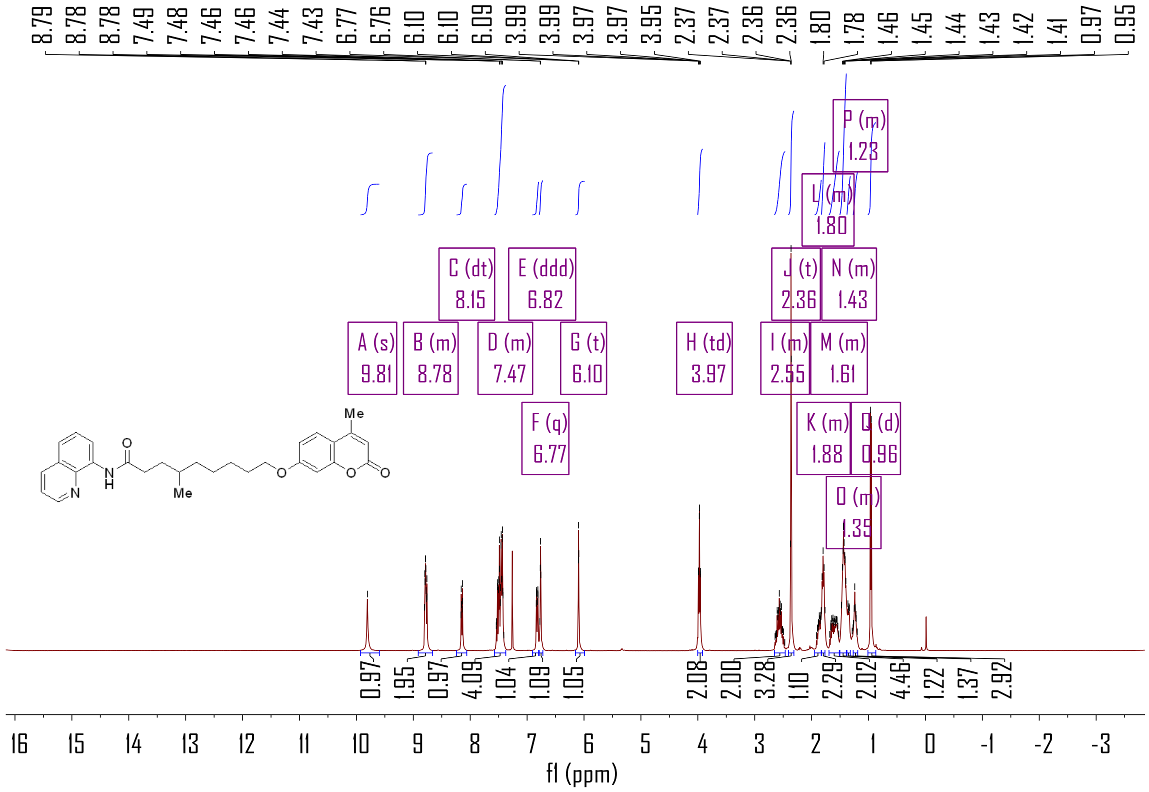

Supplementary Figure 210. ^{1}H NMR spectra of **4aw**

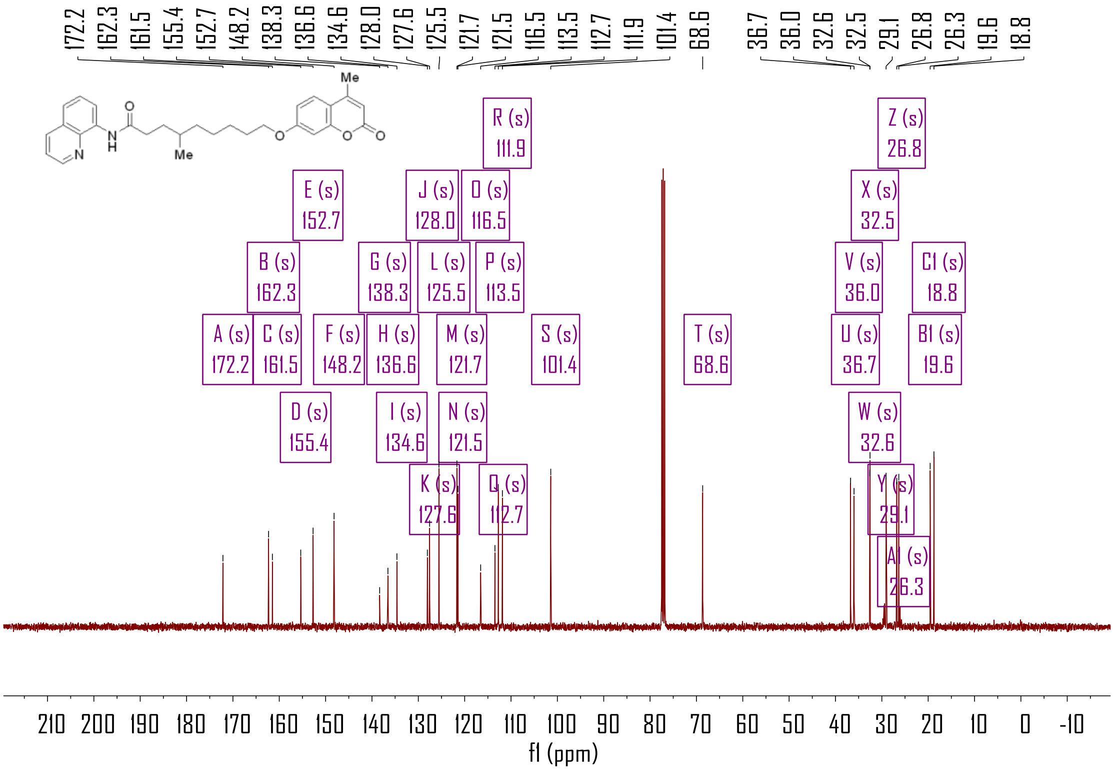

Supplementary Figure 211. ^{13}C NMR spectra of **4aw**

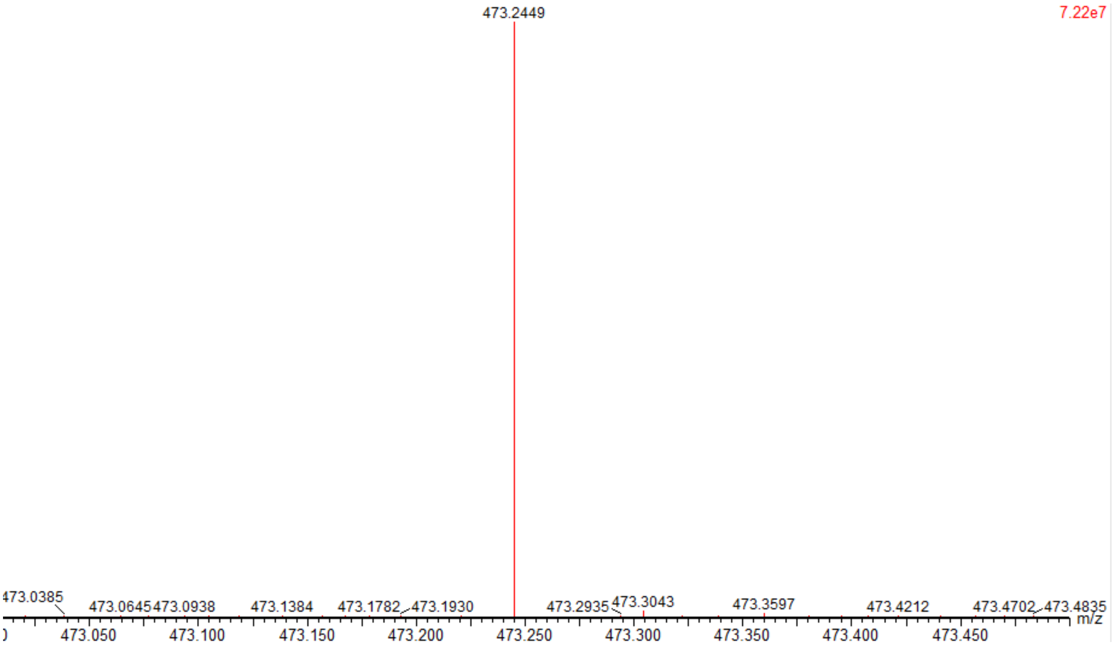

Supplementary Figure 212. HRMS spectra of **4aw**

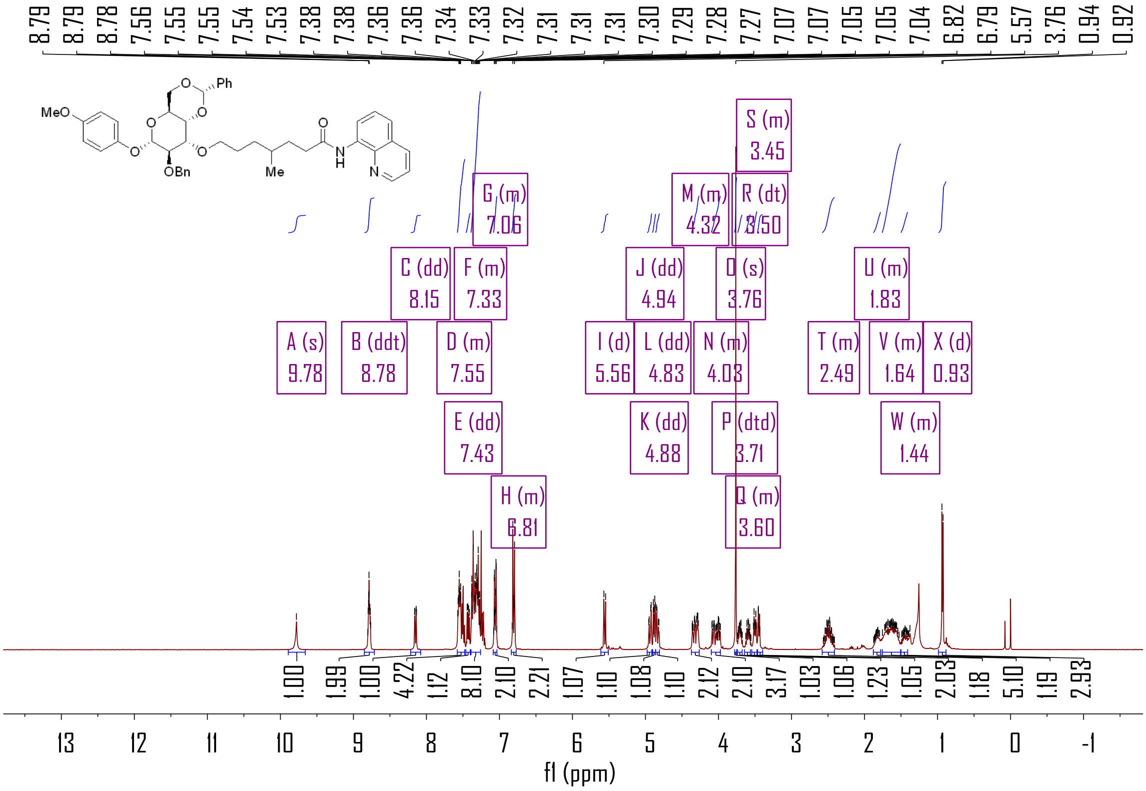

Supplementary Figure 213. ^{1}H NMR spectra of **4ax**

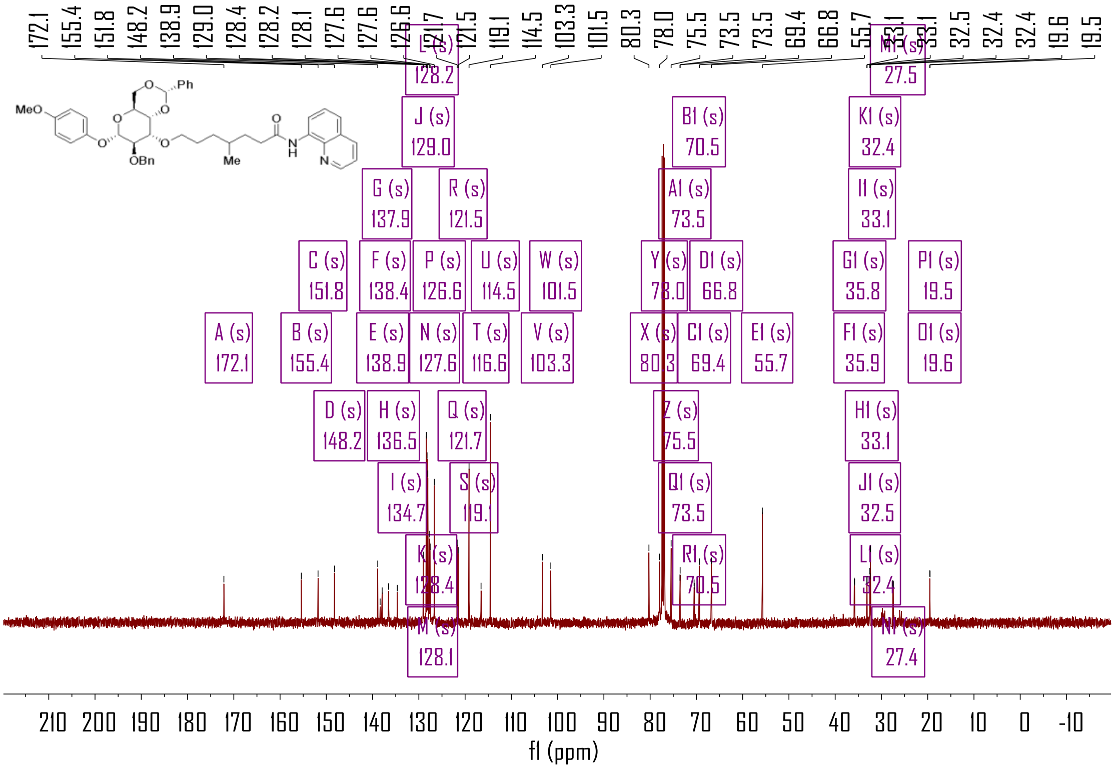

Supplementary Figure 214. ^{13}C NMR spectra of **4ax**

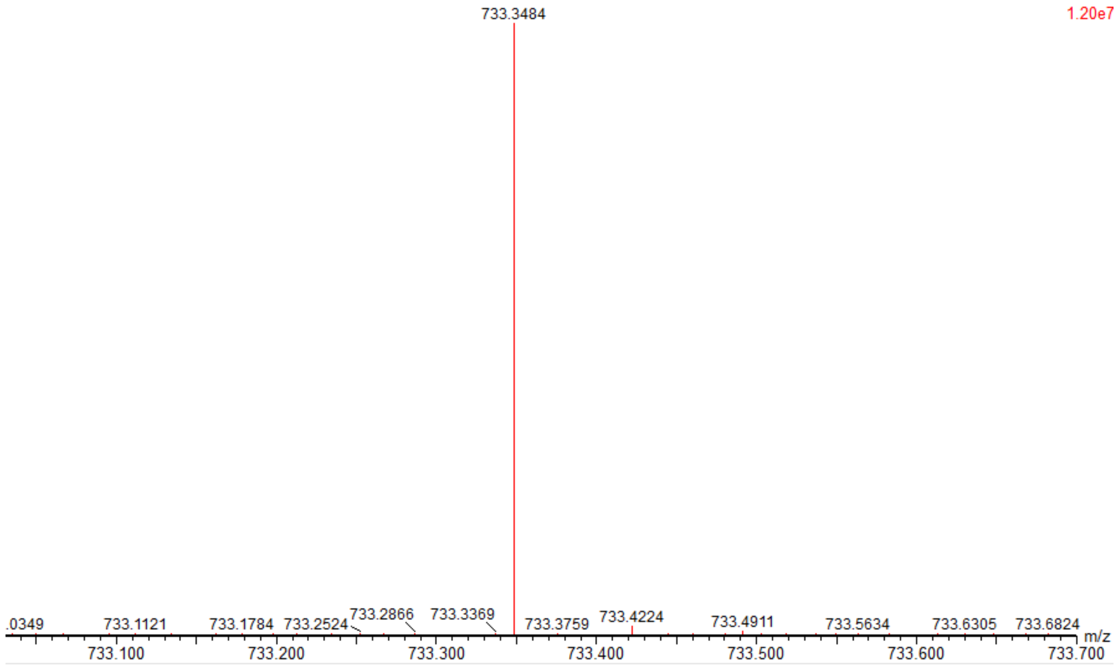

Supplementary Figure 215. HRMS spectra of **4ax**

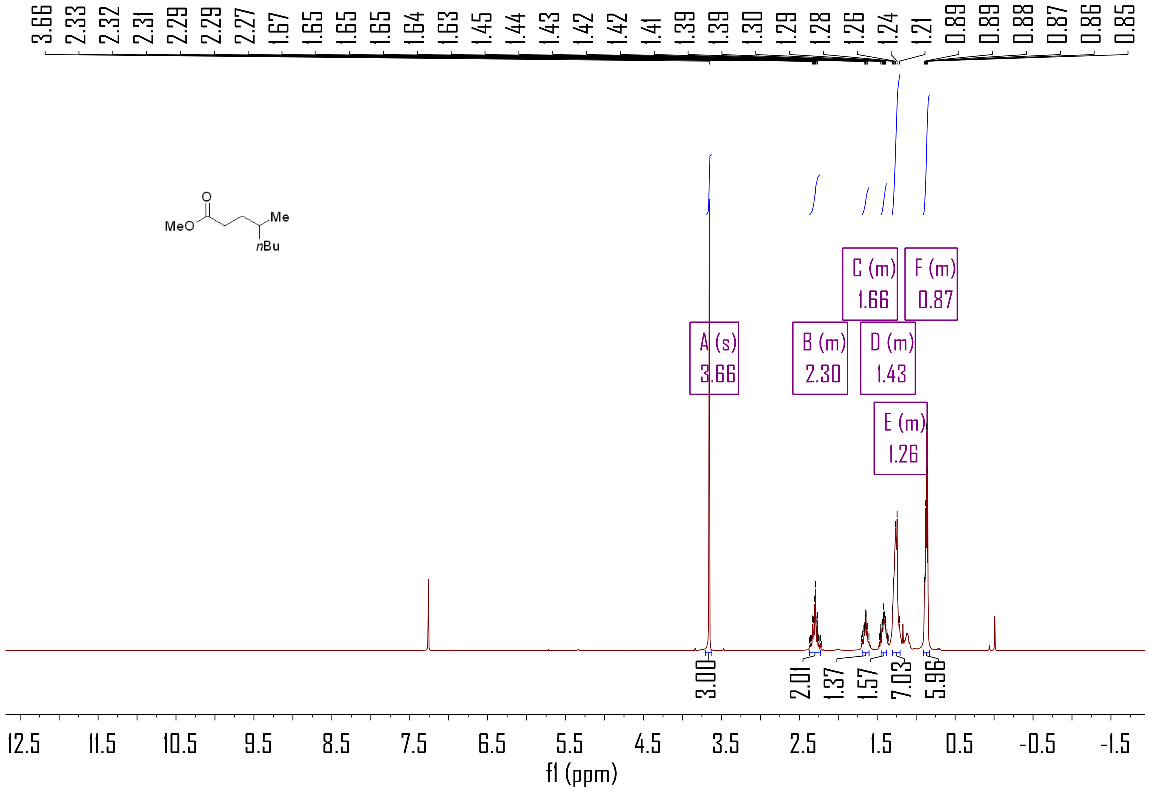

Supplementary Figure 216. ^{1}H NMR spectra of **5a**

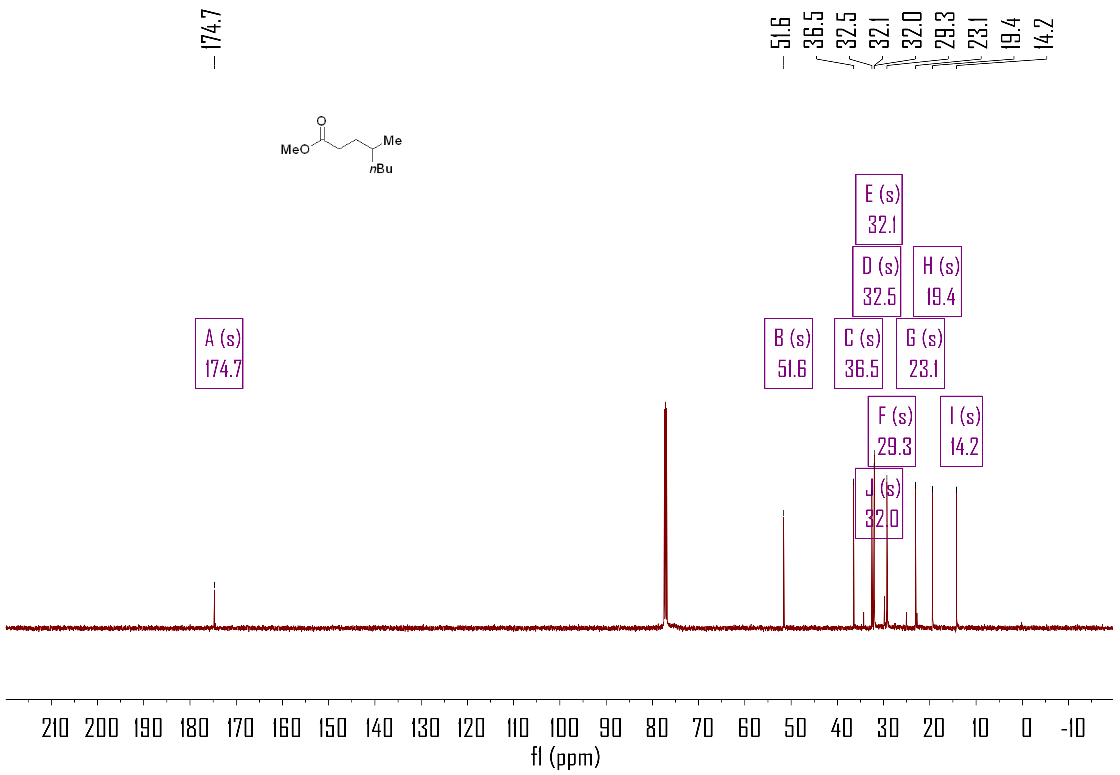

Supplementary Figure 217. ^{13}C NMR spectra of **5a**

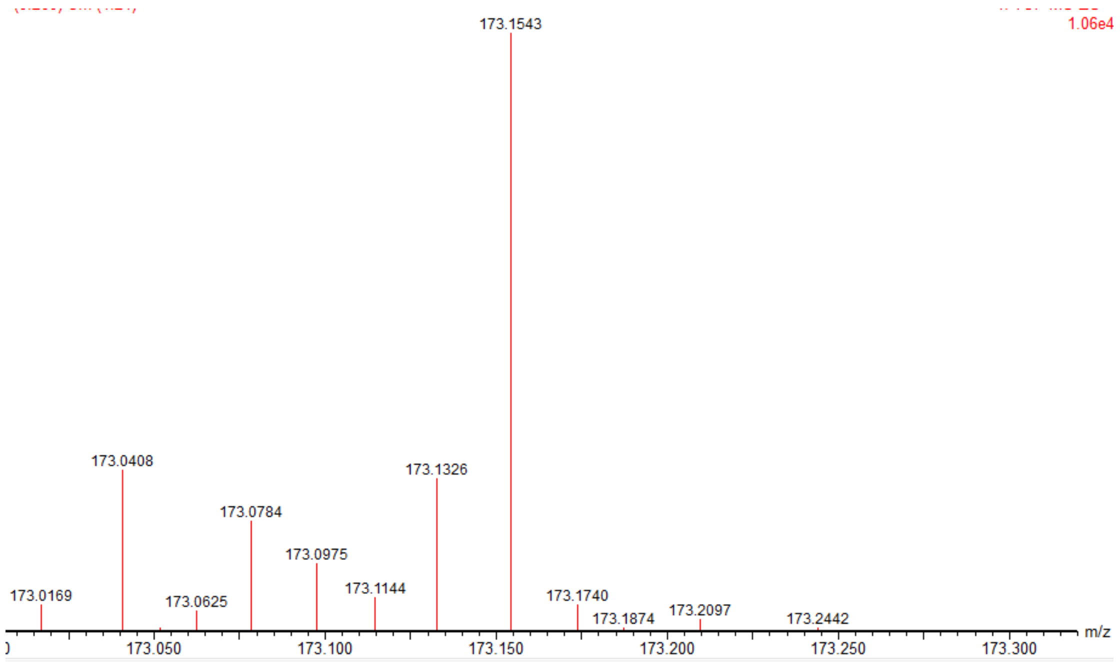

Supplementary Figure 218. HRMS spectra of **5a**

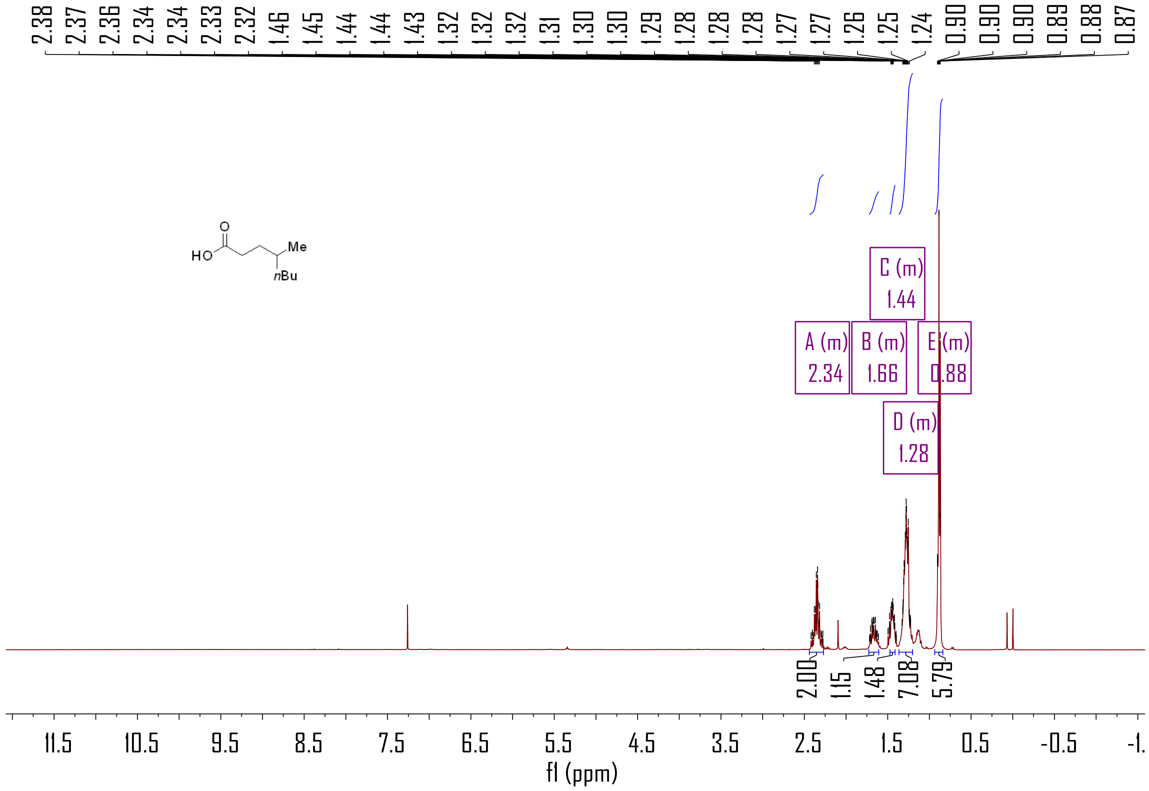

Supplementary Figure 219. ^{1}H NMR spectra of **5b**

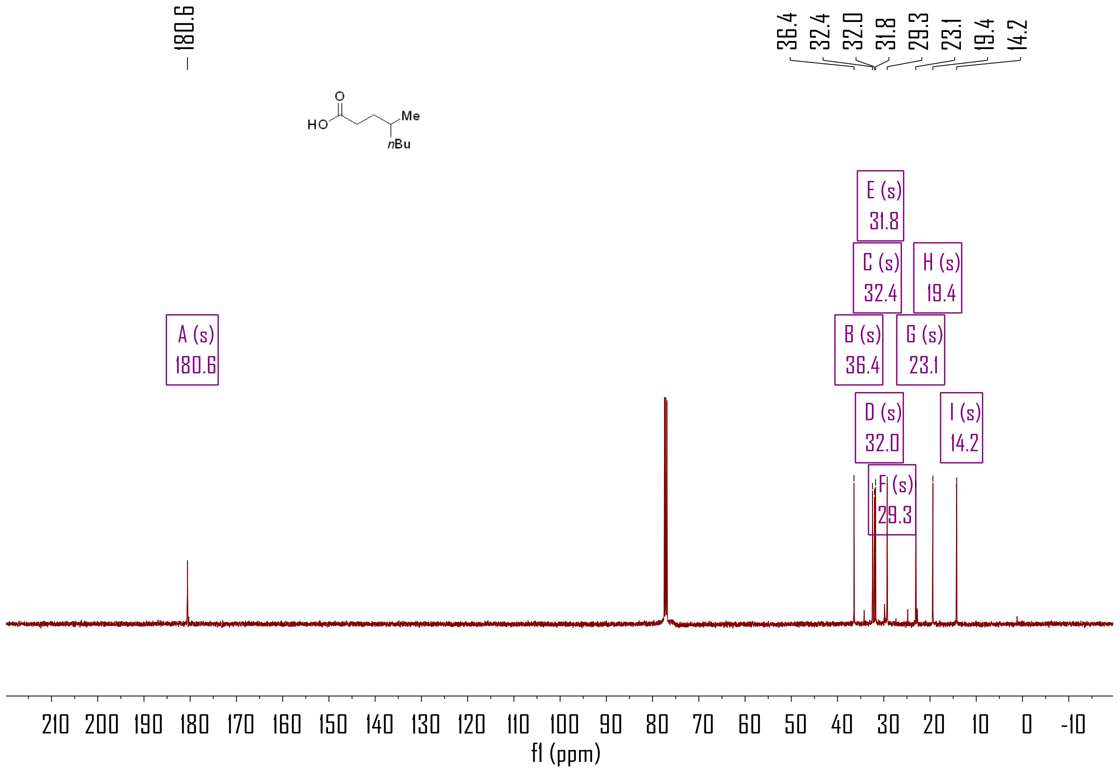

Supplementary Figure 220. ^{13}C NMR spectra of **5b**

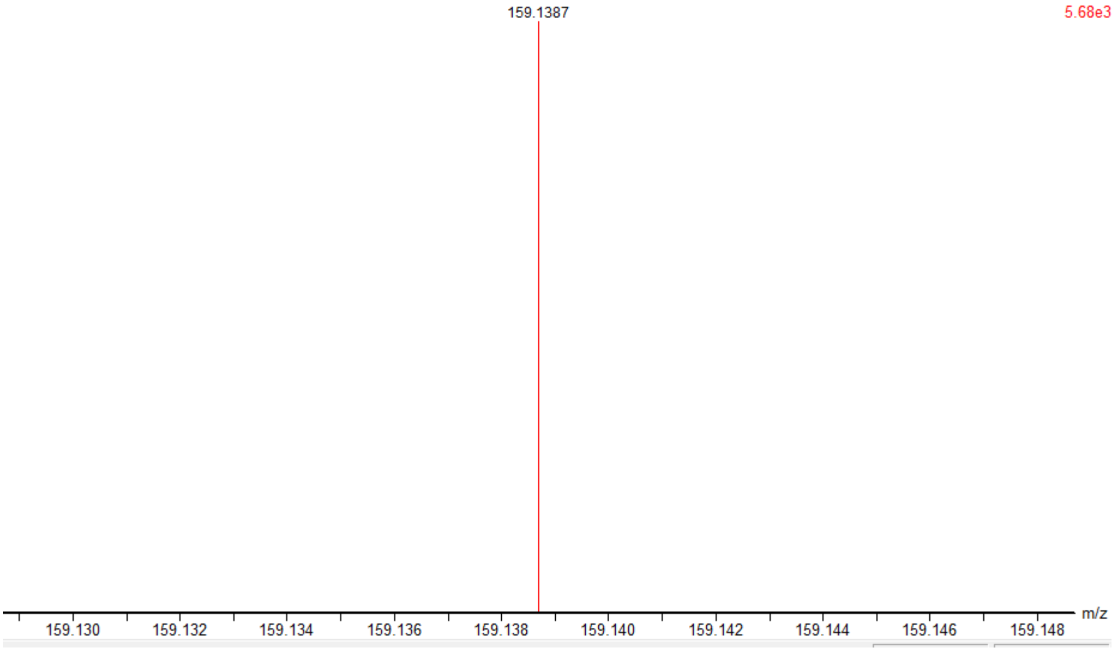

Supplementary Figure 221. HRMS spectra of **5b**

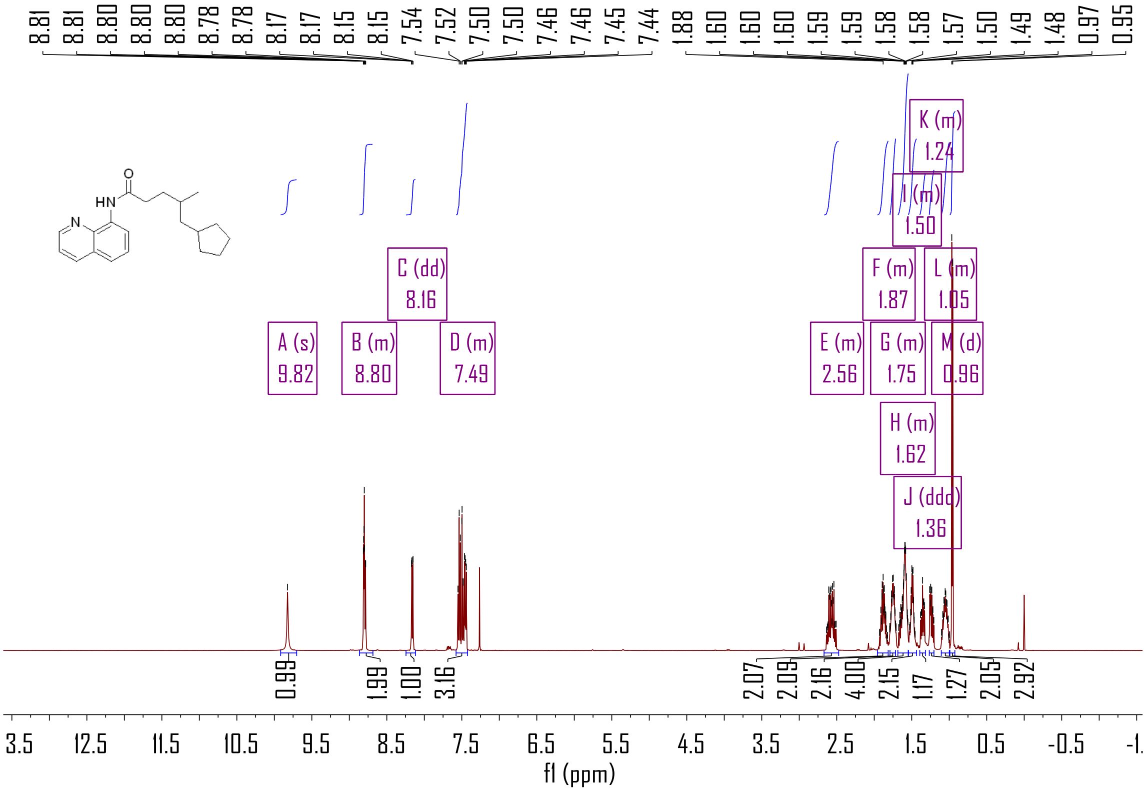

Supplementary Figure 222. ^{1}H NMR spectra of **4az**

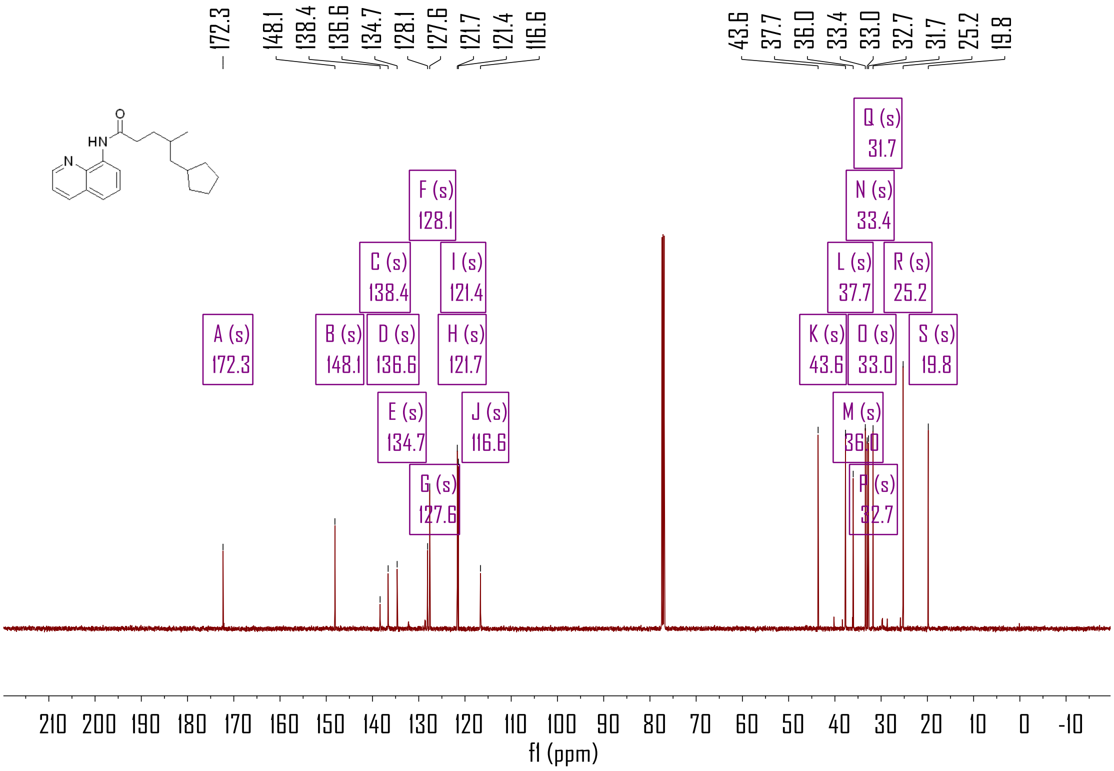

Supplementary Figure 223. ^{13}C NMR spectra of **4az**

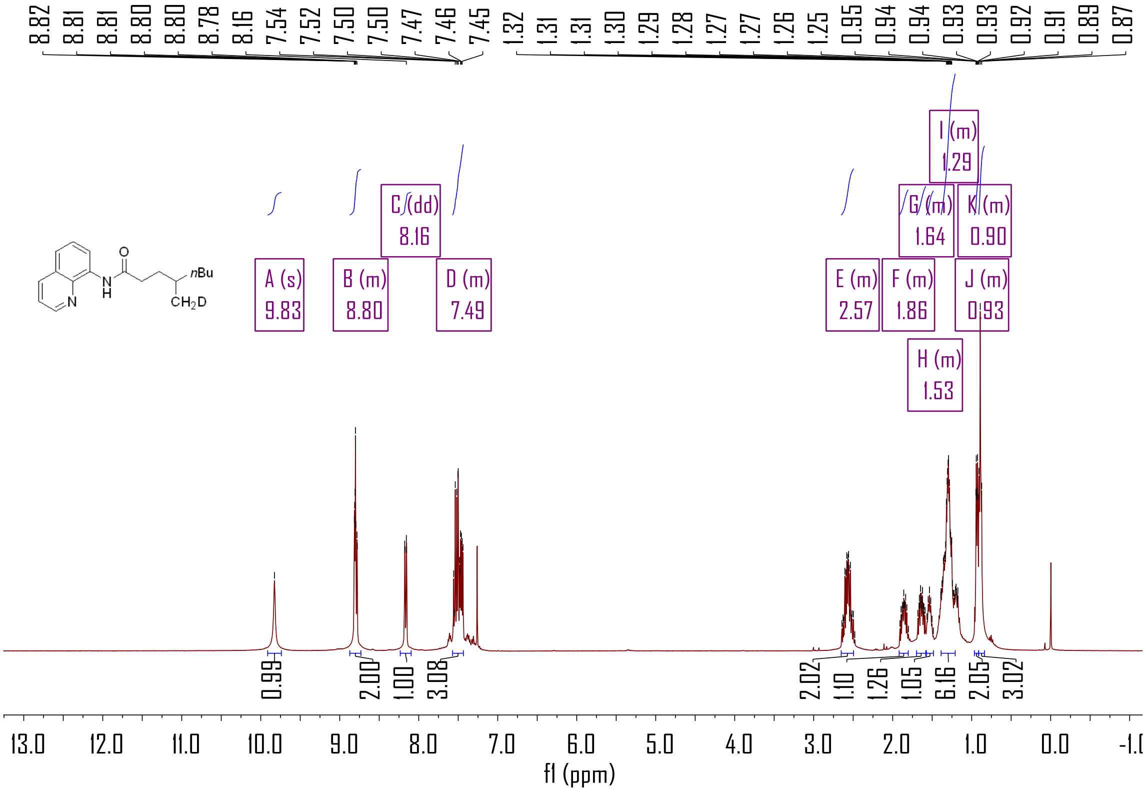

Supplementary Figure 224. ^{1}H NMR spectra of d_1-**4aa**

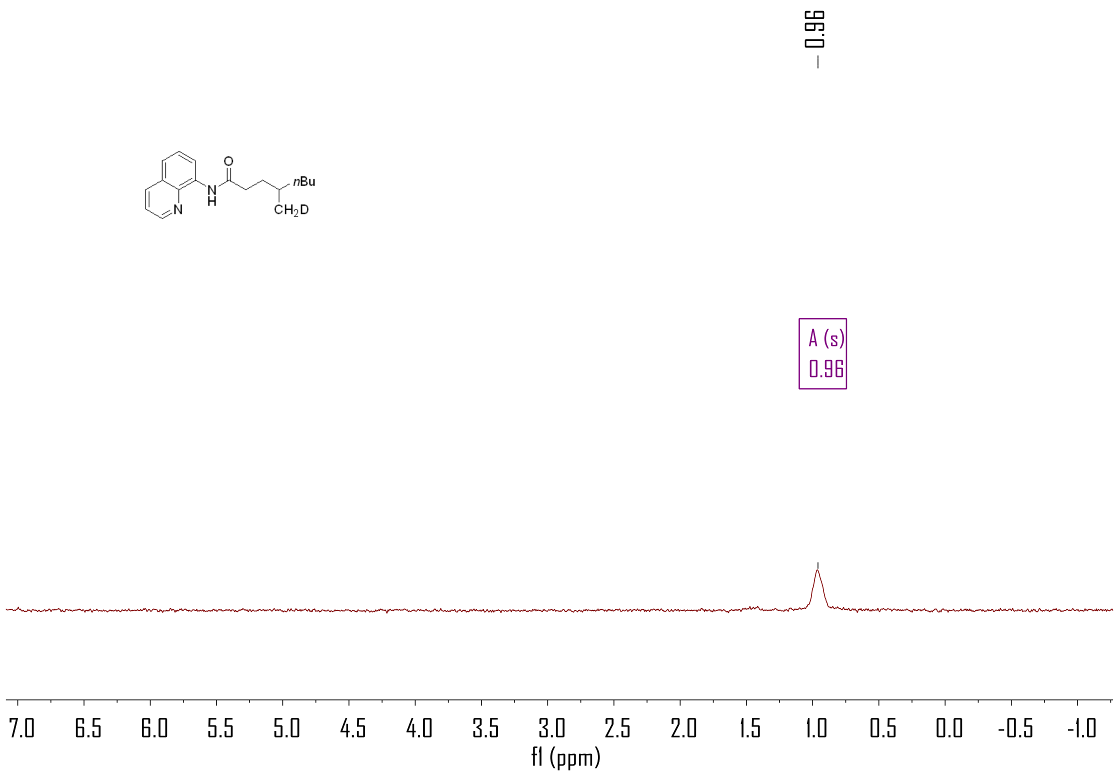

Supplementary Figure 225. ^{2}H NMR spectra of d_1-**4aa**

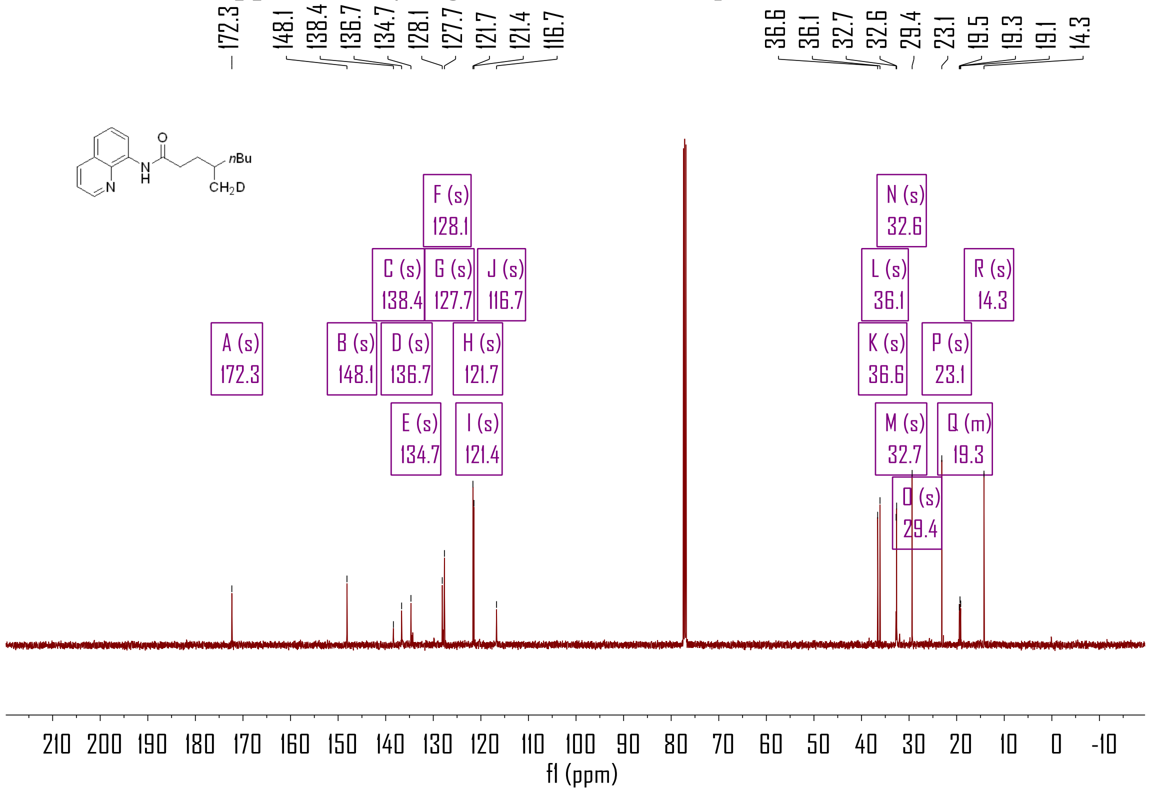

Supplementary Figure 226. ^{13}C NMR spectra of d_1-**4aa**

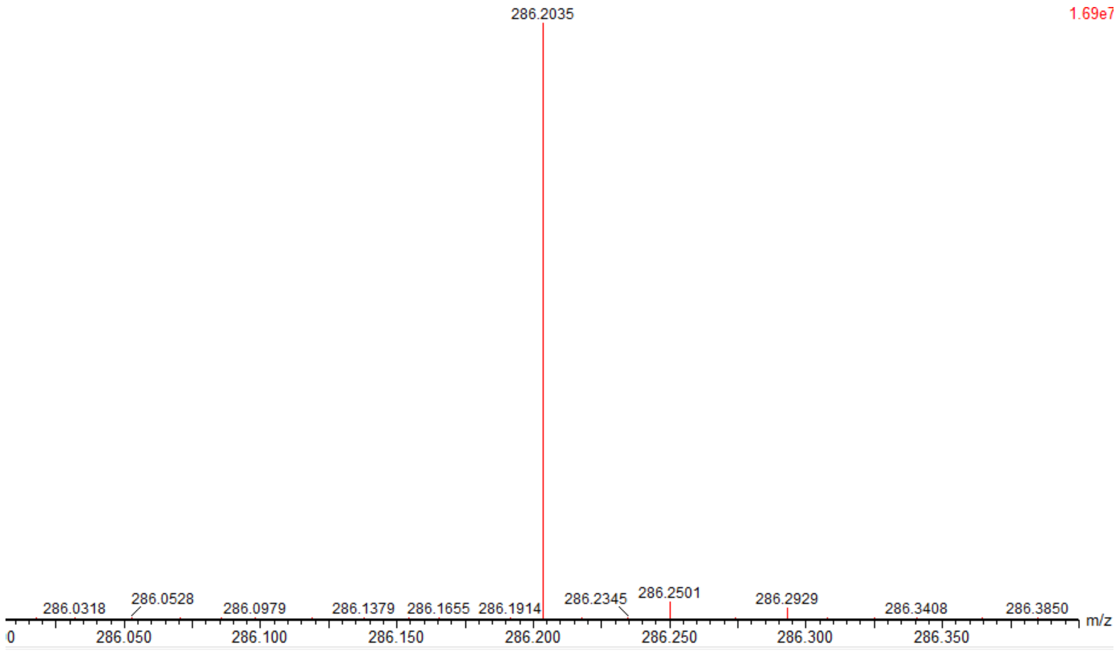

Supplementary Figure 227. HRMS spectra of d_1-**4aa**

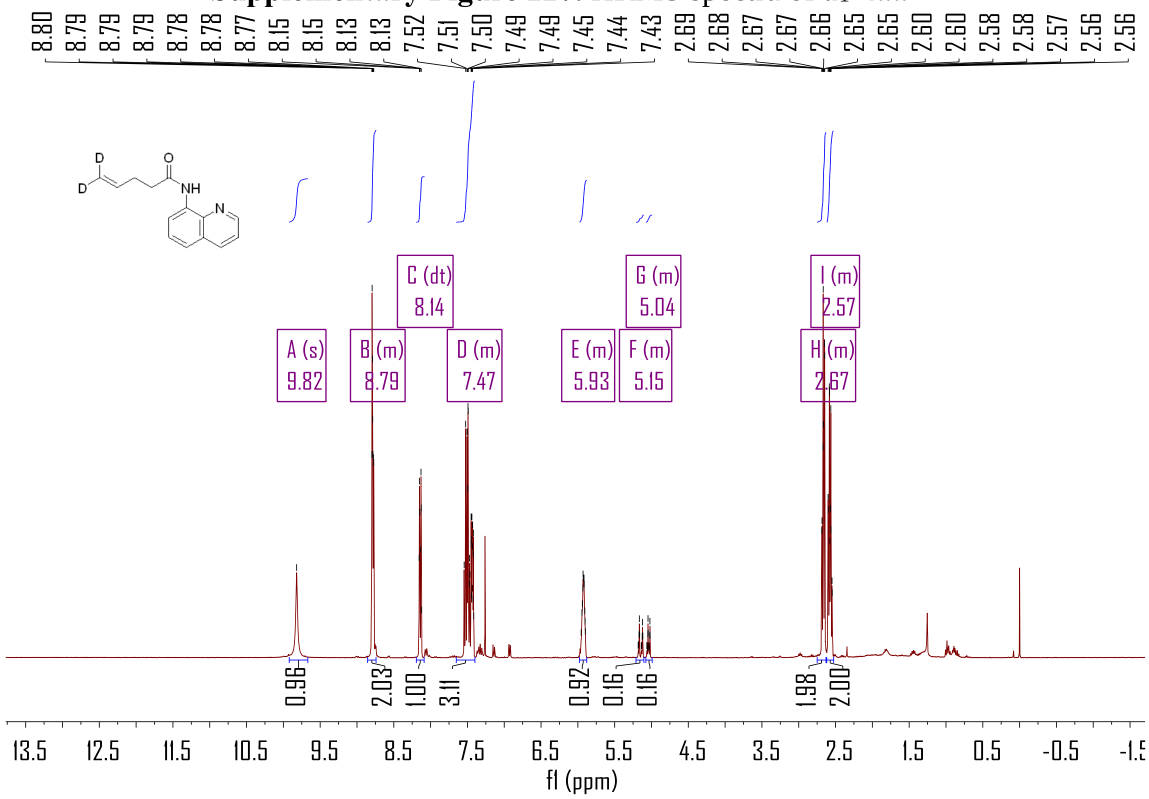

Supplementary Figure 228. ^{1}H NMR spectra of d_2-**1a**

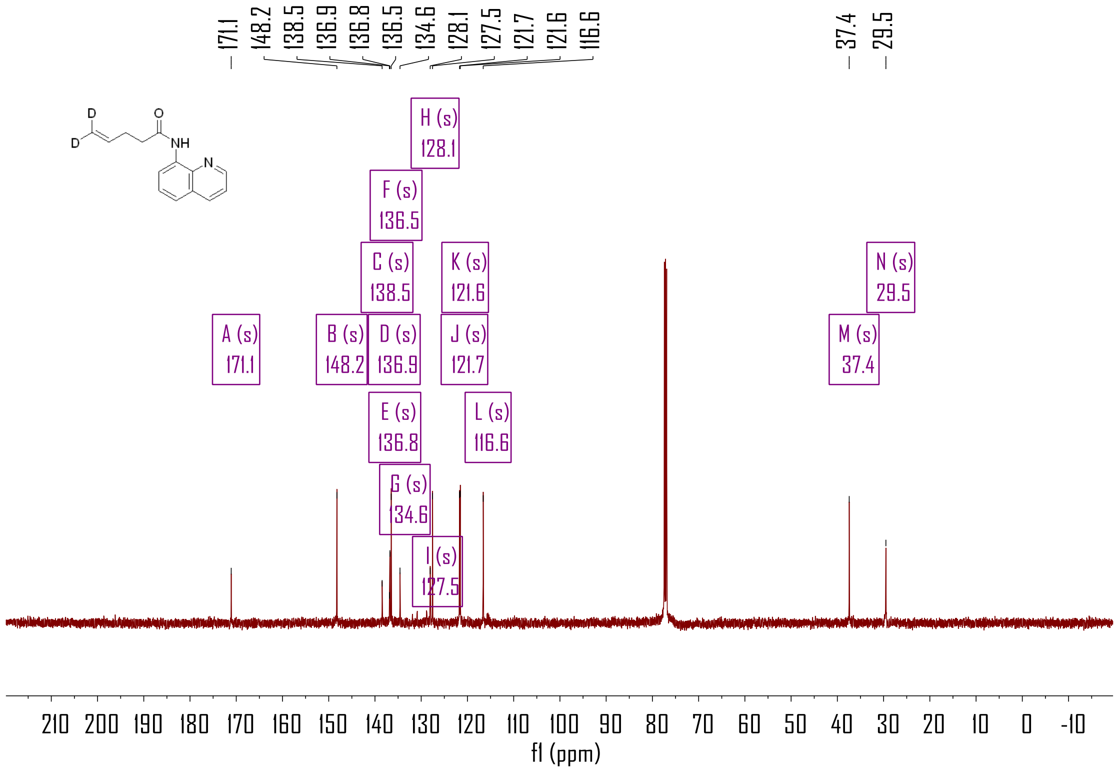

Supplementary Figure 229. ^{13}C NMR spectra of d_2-**1a**

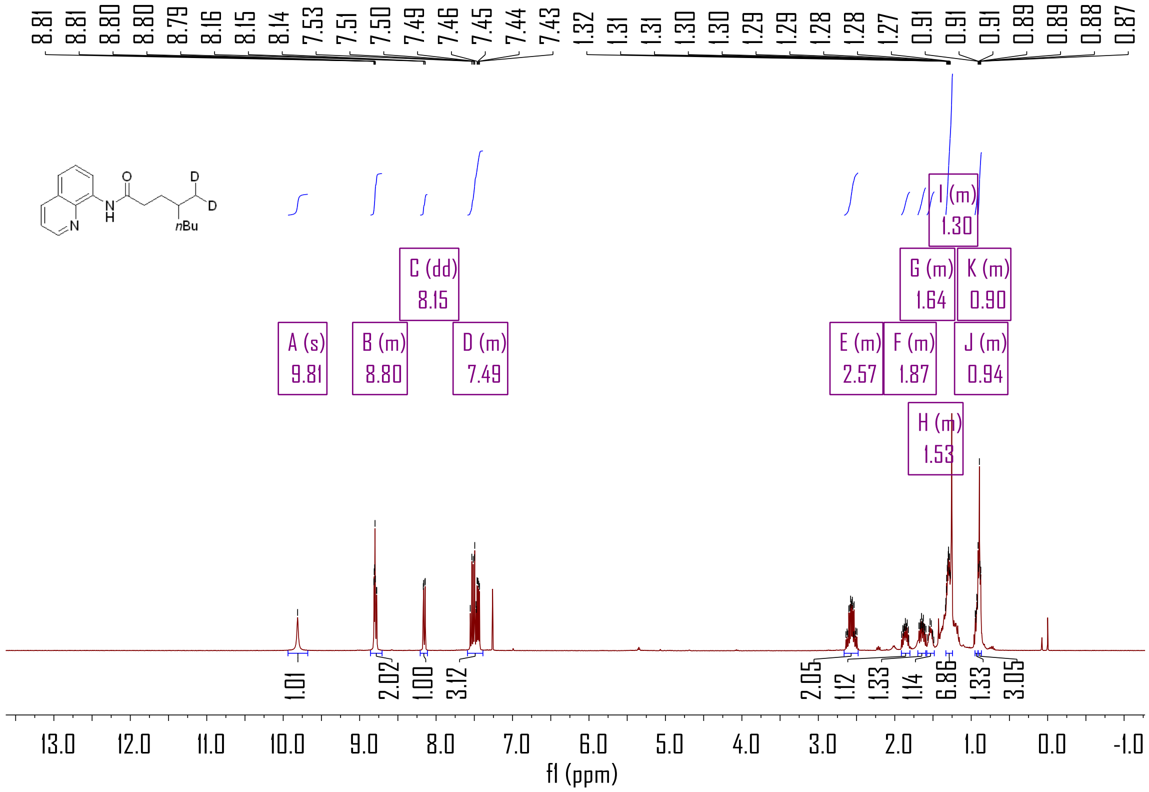

Supplementary Figure 230. ^{1}H NMR spectra of d_2-**4aa**

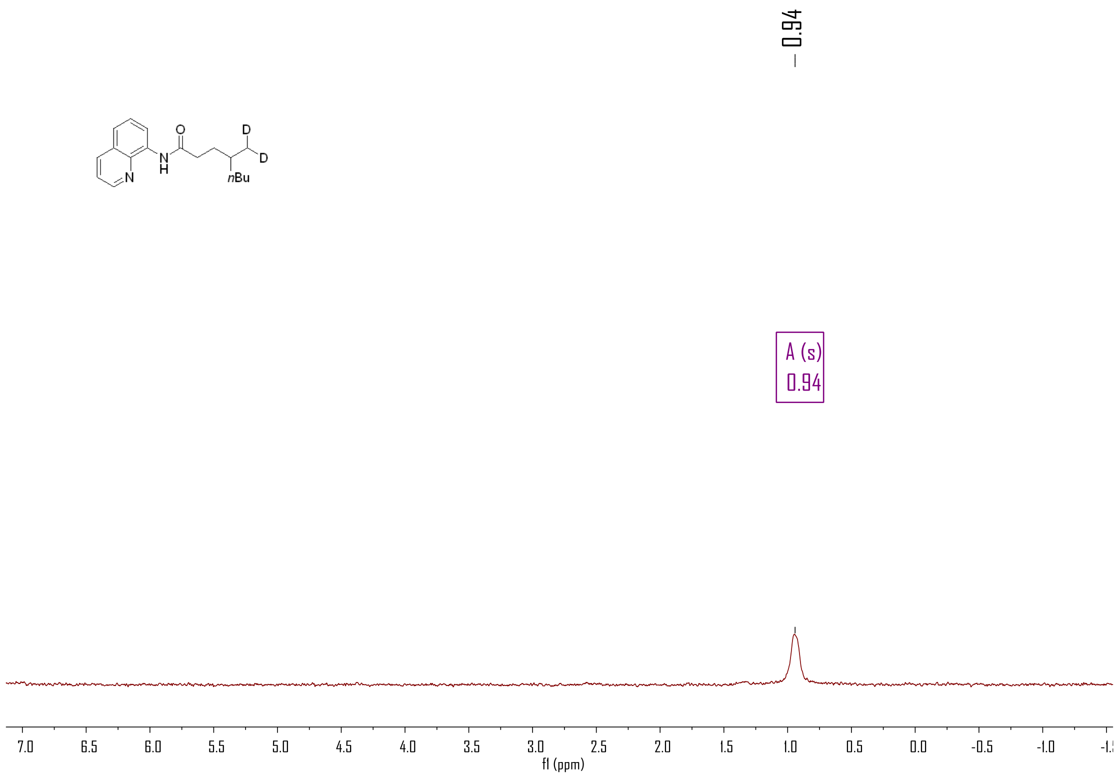

Supplementary Figure 231. ^{2}H NMR spectra of d_2-**4aa**

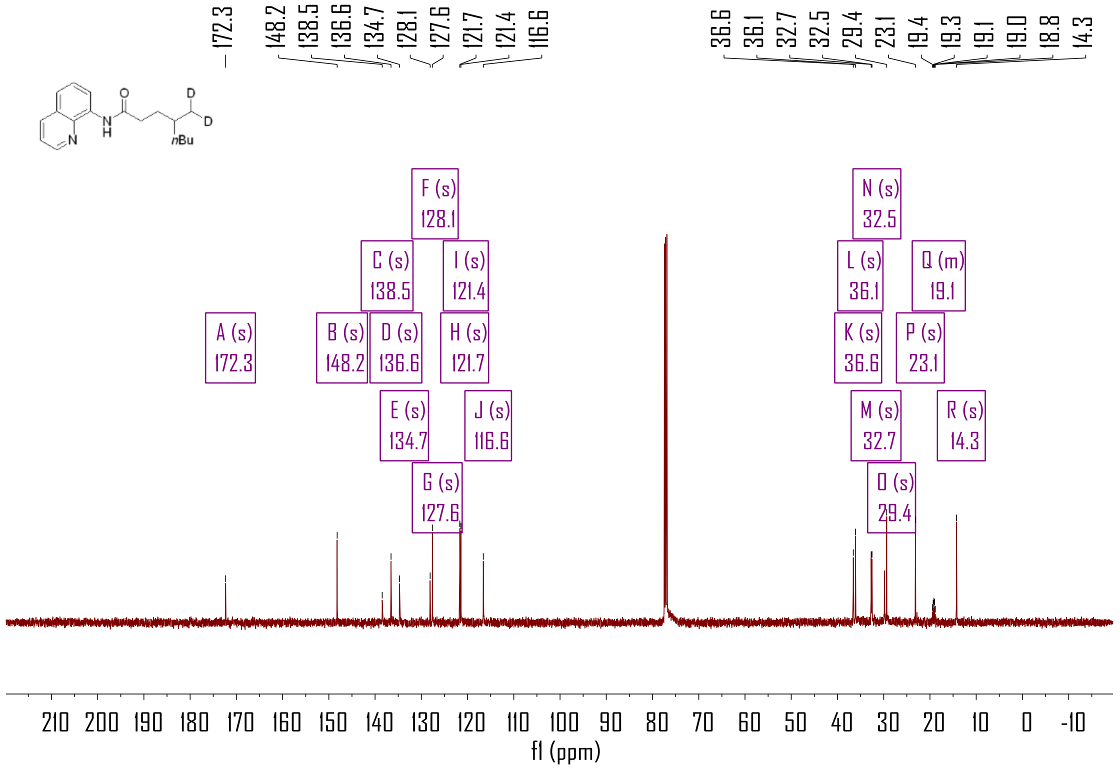

Supplementary Figure 232. ^{13}C NMR spectra of d_2-**4aa**

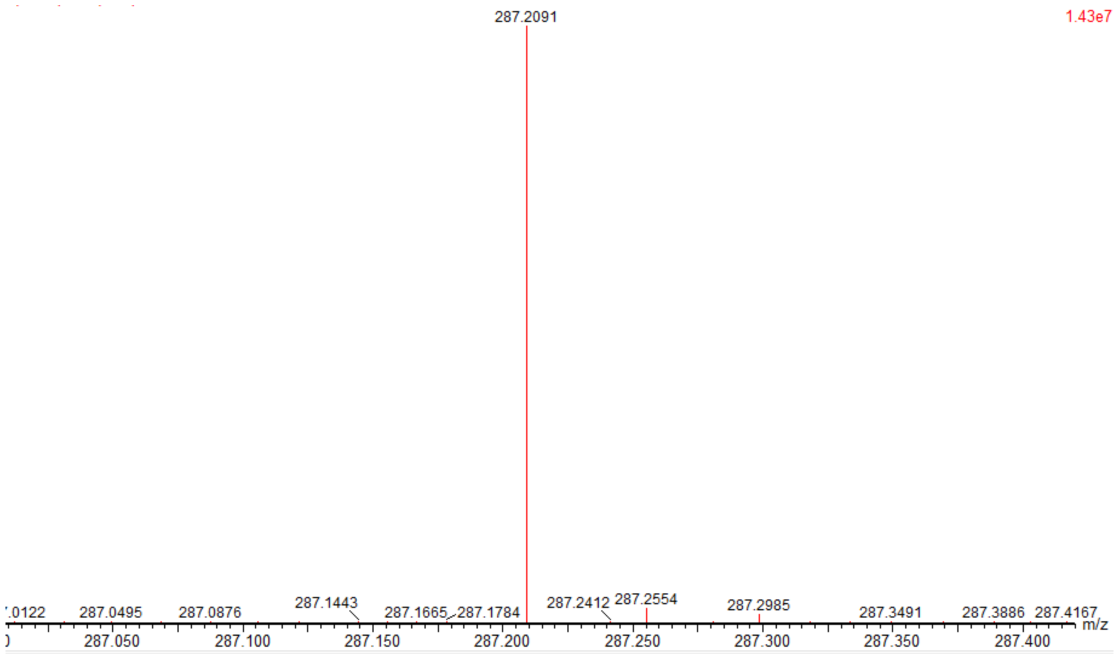

Supplementary Figure 233. HRMS spectra of d_2-**4aa**

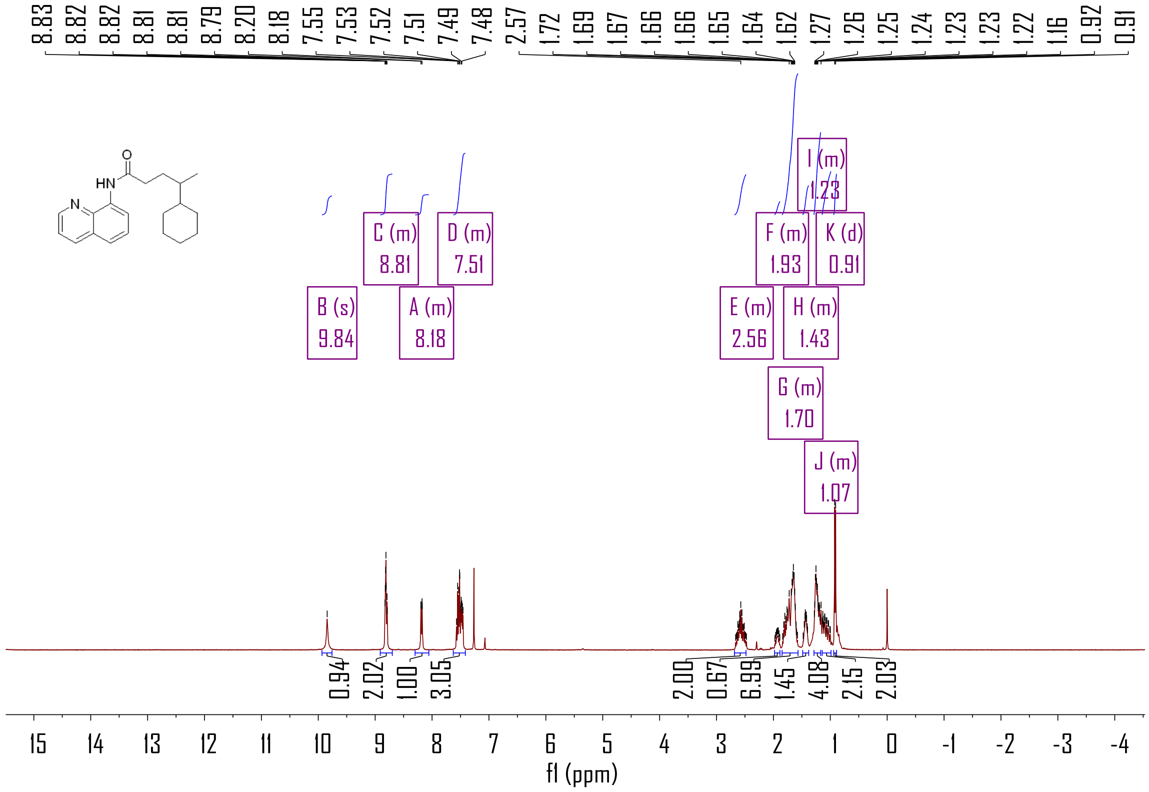

Supplementary Figure 234. ^{1}H NMR spectra of **4aB**

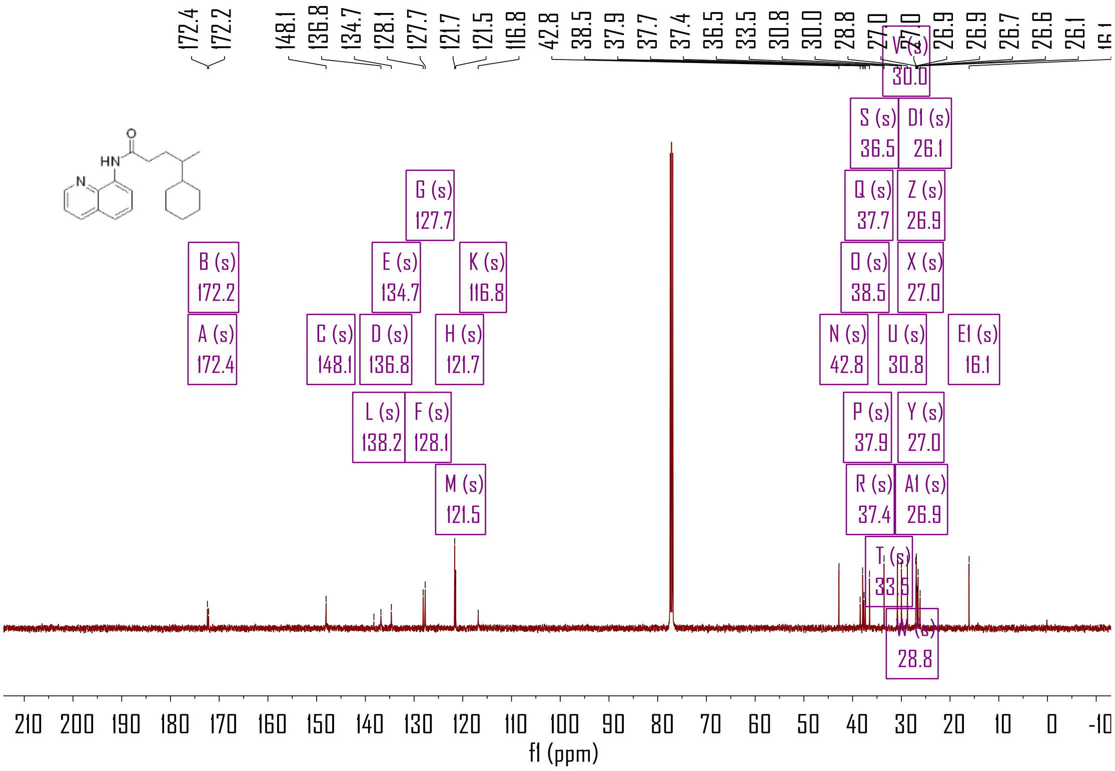

Supplementary Figure 235. ^{13}C NMR spectra of **4aB**

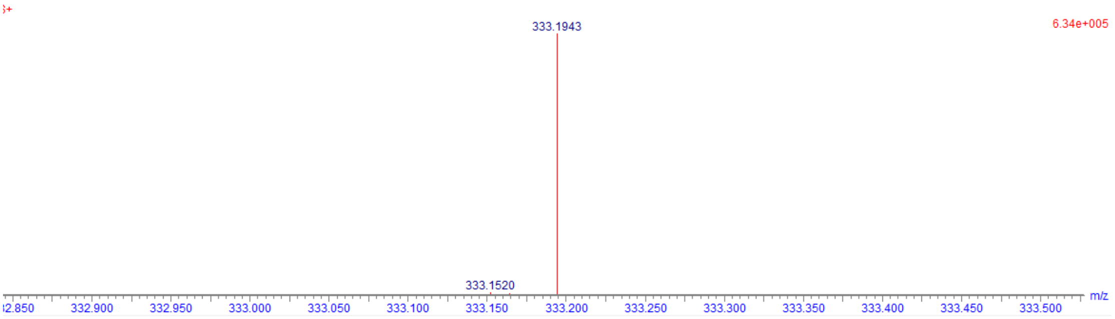

Supplementary Figure 236. HRMS spectra of **4aB**

Supplementary References

1. Jeon, J.; Lee, C.; Seo, H.; Hong, S., NiH-Catalyzed Proximal-Selective Hydroamination of Unactivated Alkenes. *J. Am. Chem. Soc.* **142**, 20470-20480 (2020).

2. Huffman, T. R.; Wu, Y.; Emmerich, A.; Shenvi, R. A., Intermolecular Heck Coupling with Hindered Alkenes Directed by Potassium Carboxylates. *Angew. Chem. Int. Ed.* **58**, 2371-2376 (2019).

3. Wang, X. X.; Lu, X.; He, S. J.; Fu, Y., Nickel-catalyzed three-component olefin reductive dicarbofunctionalization to access alkylborates. *Chem. Sci.* **11**, 7950-7956 (2020).

4. Lu, X.; Xiao, B.; Zhang, Z.; Gong, T.; Su, W.; Yi, J.; Fu, Y.; Liu, L., Practical carbon-carbon bond formation from olefins through nickel-catalyzed reductive olefin hydrocarbonation. *Nat. Commun.* **7**, 11129 (2016).

5. Jadhav, V. H.; Jeong, H.-J.; Lim, S. T.; Sohn, M.-H.; Song, C. E.; Kim, D. W., Polymer-supported oligoethylene glycols as heterogeneous multifunctional catalysts for nucleophilic substitution. *Tetrahedron,* **69**, 3577-3583 (2013).

6. Rezazadeh, S.; Devannah, V.; Watson, D. A., Nickel-Catalyzed C-Alkylation of Nitroalkanes with Unactivated Alkyl Iodides. *J. Am. Chem. Soc.* **139**, 8110-8113 (2017).

7. Xu, M. Y.; Jiang, W. T.; Li, Y.; Xu, Q. H.; Zhou, Q. L.; Yang, S.; Xiao, B., Alkyl Carbagermatranes Enable Practical Palladium-Catalyzed sp^2-sp^3 Cross-Coupling. *J. Am. Chem. Soc.* **141**, 7582-7588 (2019).

8. Dang, H.; Cox, N.; Lalic, G., Copper-catalyzed reduction of alkyl triflates and iodides: an efficient method for the deoxygenation of primary and secondary alcohols. *Angew. Chem. Int. Ed.* **53**, 752-756 (2014).

9. Yu, E.; Mangunuru, H. P. R.; Telang, N. S.; Kong, C. J.; Verghese, J.; Gilliland Iii, S. E.; Ahmad, S.; Dominey, R. N.; Gupton, B. F., High-yielding continuous-flow synthesis of antimalarial drug hydroxychloroquine. *Beilstein J. Org. Chem.* **14**, 583-592 (2018).

10. Lecourt, C.; Boinapally, S.; Dhambri, S.; Boissonnat, G.; Meyer, C.; Cossy, J.; Sautel, F.; Massiot, G.; Ardisson, J.; Sorin, G.; Lannou, M. I., Elaboration of Sterically Hindered delta-Lactones through Ring-Closing Metathesis: Application to the Synthesis of the C1-C27 Fragment of Hemicalide. *J. Org. Chem.* **81**, 12275-12290

(2016).

11. Chen, K.; Li, X.; Huang, B.; Ye, Q.; Xiao, W.; Guan, X.; Chen, L.; Peng, Y., Thiophenic silicon phthalocyanines: synthesis, characterization, and photophysical properties. *New J. Chem.*, **45**, 457-462 (2021).

12. Plocki, S.; Aoun, D.; Ahamada-Himidi, A.; Tavarès-Camarinha, F.; Dong, C.-Z.; Massicot, F.; Huet, J.; Adolphe-Pierre, S.; Chau, F.; Godfroid, J.-J.; Gresh, N.; Ombetta, J. E.; Heymans, F., Molecular Modeling, Design, and Synthesis of Less Lipophilic Derivatives of 3-(4-Tetradecyloxybenzyl)-4H-1,2,4-oxadiazol-5-one (PMS1062) Specific for Group II Enzyme. *Eur. J. Org. Chem.* **2005** (13), 2747-2757 (2005).

13. Thornton, A. R.; Martin, V. I.; Blakey, S. B., π-Nucleophile Traps for Metallonitrene/Alkyne Cascade Reactions: A Versatile Process for the Synthesis of r-Aminocyclopropanes and α-Aminostyrenes. *J. Am. Chem. Soc.* **131**, 2434-2435 (2009).

14. Fu, M. C.; Wang, J. X.; Shang, R., Triphenylphosphine-Catalyzed Alkylative Iododecarboxylation with Lithium Iodide under Visible Light. *Org. Lett.* **22**, 8572-8577 (2020).

15. Li, J.; Ren, Q.; Cheng, X.; Karaghiosoff, K.; Knochel, P., Chromium(II)-Catalyzed Diastereoselective and Chemoselective Csp^2-Csp^3 Cross-Couplings Using Organomagnesium Reagents. *J. Am. Chem. Soc.* **141**, 18127-18135 (2019).

16. Srivastava, P.; Barman, J.; Pathmasiri, W.; Plashkevych, O.; Wenska, M.; Chattopadhyaya, J., Five- and Six-Membered Conformationally Locked 2',4'-Carbocyclic ribo-Thymidines: Synthesis, Structure, and Biochemical Studies. *J. Am. Chem. Soc.* **129**, 8362-8379 (2007).

17. Deguchi, T.; Xin, H.-L.; Morimoto, H.; Ohshima, T., Direct Catalytic Alcoholysis of Unactivated 8-Aminoquinoline Amides. *ACS Catal.* **7**, 3157-3161 (2017).

18. Frisch, M. J.; Trucks, G. W.; Schlegel, H. B.; Scuseria, G. E.; Robb, M. A.; Cheeseman, J. R.; Scalmani, G.; Barone, V.; Petersson, G. A.; Nakatsuji, H.; Li, X.; Caricato, M.; Marenich, A. V.; Bloino, J.; Janesko, B. G.; Gomperts, R.; Mennucci, B.; Hratchian, H. P.; Ortiz, J. V.; Izmaylov, A. F.; Sonnenberg, J. L.; Williams-Young, D.; Ding, F.; Lipparini, F.; Egidi, F.; Goings, J.; Peng, B.; Petrone, A.; Henderson, T.; Ranasinghe, D.; Zakrzewski, V. G.; Gao, J.; Rega, N.; Zheng, G.; Liang, W.; Hada, M.; Ehara, M.; Toyota, K.; Fukuda, R.; Hasegawa, J.; Ishida, M.; Nakajima, T.; Honda, Y.;

Kitao, O.; Nakai, H.; Vreven, T.; Throssell, K.; Montgomery, J. A.; Peralta, J. E.; Ogliaro, F.; Bearpark, M. J.; Heyd, J. J.; Brothers, E. N.; Kudin, K. N.; Staroverov, V. N.; Keith, T. A.; Kobayashi, R.; Normand, J.; Raghavachari, K.; Rendell, A. P.; Burant, J. C.; Iyengar, S. S.; Tomasi, J.; Cossi, M.; Millam, J. M.; Klene, M.; Adamo, C.; Cammi, R.; Ochterski, J. W.; Martin, R. L.; Morokuma, K.; Farkas, O.; Foresman, J. B.; Fox, D. J., , Gaussian 16, Revision C.01; Gaussian, Inc., Wallingford CT, **2019**.

19. Becke, A. D., Density - functional Thermochemistry. III. The Role of Exact Exchange. *J. Chem. Phys.* **98**, 5648-5652 (1993).

20. Lee, C.; Yang, W.; Parr, R. G., Development of the Colle-Salvetti Correlation-Energy Formula into a Functional of the Electron Density. *Phys. Rev. B.* **37**, 785-789 (1988).

21. Andrae, D.; Häußermann, U.; Dolg, M.; Stoll, H.; Preuß, H., Energy-Adjustedab Initio Pseudopotentials for the Second and Third Row Transition Elements. *Theor. Chim. Acta,* **77**, 123-141 (1990).

22. Chai, J.-D.; Head-Gordon, M., Long-Range Corrected Hybrid Density Functionals with Damped Atom–Atom Dispersion Corrections. *Phys. Chem. Chem. Phys.* **10**, 6615 (2008).

23. Marenich, A. V.; Cramer, C. J.; Truhlar, D. G., Universal Solvation Model Based on Solute Electron Density and on a Continuum Model of the Solvent Defined by the Bulk Dielectric Constant and Atomic Surface Tensions. *J. Phys. Chem. B* **113**, 6378-6396 (2009).

24. Fukui, K., Formulation of the Reaction Coordinate. *J. Phys. Chem.* **74**, 4161-4163 (1970).

25. Fukui, K., The Path of Chemical Reactions - the IRC Approach. *Acc. Chem. Res.* **14**, 363-368 (1981).

26. Zhao, Y.; Truhlar, D. G., Density Functionals with Broad Applicability in Chemistry. *Acc. Chem. Res.* **41**, 157-167 (2008).
