# Supplementary material for: NiH-catalysed proximal-selective hydroalkylation of unactivated alkenes and the ligand effects on regioselectivity
Source: Nat Commun. 2022 Apr 7;13:1890. doi: 10.1038/s41467-022-29554-4 (PMC8990077; doi:10.1038/s41467-022-29554-4)
Supplement: Supplementary file 3 — Description of Additional Supplementary Files [file 41467_2022_29554_MOESM3_ESM.pdf]

Description of Additional Supplementary Files

File Name: Supplementary Data 1

Description: Cartesian coordinates of the calculated transition states.
