# Supplementary material for: NiH-catalysed proximal-selective hydroalkylation of unactivated alkenes and the ligand effects on regioselectivity
Source: Nat Commun. 2022 Apr 7;13:1890. doi: 10.1038/s41467-022-29554-4 (PMC8990077; doi:10.1038/s41467-022-29554-4)
Supplement: Supplementary file 4 — Supplementary Data 1 [file 41467_2022_29554_MOESM4_ESM.zip › Supplementary Data 1.docx]

**Cartesian Coordinates**

**Int1**

C 0.80676400 -2.47756600 1.52347600

C 0.73015100 -0.96478700 1.68238700

N -0.05488300 -0.32646900 0.78266300

C -0.30531500 1.04666000 0.82609400

C -1.05265000 1.56696100 -0.28962600

C 0.04614100 1.95718100 1.83083900

C -1.35346700 2.95660700 -0.39969300

C -0.26090200 3.33412500 1.71607500

H 0.53796900 1.59644700 2.72325900

C -2.17912900 1.09427000 -2.27242600

C -2.09342500 3.37813200 -1.53515800

C -0.92911700 3.84321600 0.62382600

H 0.04254700 3.99683300 2.52278500

C -2.50324300 2.45317100 -2.46892900

H -2.33321600 4.43221900 -1.64827700

H -1.15645700 4.90210100 0.53957900

H -3.07283200 2.74472200 -3.34529300

N -1.48310000 0.66808300 -1.22784500

C 1.42942400 -2.89188000 0.17348400

H -2.49514000 0.33512900 -2.98011900

H 2.37855500 -2.35432000 0.04552000

H -0.19404900 -2.91756500 1.61290000

H 1.41889600 -2.85567000 2.34760100

H 1.66415500 -3.96250900 0.22423000

Ni -0.91968200 -1.23062800 -0.76436700

C 0.55177800 -2.64915700 -1.04477300

H 0.03898400 -3.52629700 -1.43981500

C 0.74711100 -1.54493000 -1.88217000

H 0.43542100 -1.57121900 -2.92507600

H 1.53045700 -0.82686900 -1.64067100

H -1.66028200 -1.82481900 -1.88055200

O 1.41237300 -0.41306000 2.58137500

N -2.48389800 -1.85088400 0.44704300

H -2.17028400 -1.75335300 1.41409800

H -2.65994500 -2.84594100 0.30140600

C -3.73786700 -1.09528800 0.26286200

H -3.52704600 -0.03860900 0.44869700

H -4.04461800 -1.19367200 -0.78247500

C -4.85820100 -1.56716400 1.18173500

H -4.53566600 -1.47409600 2.23213700

H -5.06749400 -2.63296400 0.99162400

O -5.99602400 -0.75348700 0.90958000

H -6.71360700 -1.04929900 1.49348100

Br 4.26573700 -0.16177100 -1.02334600

K 3.29620900 1.16838200 1.55029900

**Int2**

C -1.63159500 0.58568900 -1.29219900

C -2.87640400 0.77484800 -1.90719200

C -1.17928300 1.61928700 -0.38560800

H -3.24541800 0.00127000 -2.57133500

C -3.65060200 1.93377400 -1.68571200

C -1.96994400 2.79585000 -0.17760300

H -4.60635700 2.03134700 -2.19533200

C -3.21387300 2.93807100 -0.84498500

H -3.80593300 3.83349800 -0.67677300

C -1.47302700 3.77739000 0.72054300

N -0.00525700 1.43138600 0.29987700

C 0.41860200 2.36552700 1.13682500

C -0.27993200 3.56927100 1.37227000

H -2.05100200 4.68347500 0.88505400

H 1.34472500 2.15239100 1.66139900

H 0.12562900 4.29837800 2.06638300

N -0.78966200 -0.48550000 -1.52619600

C -0.22380700 -2.81938100 -1.92136900

H -0.45413300 -3.14387700 -2.94785600

C 1.26663600 -2.45304400 -1.83286100

H 1.45707300 -1.60918800 -2.50893600

H 1.83322400 -3.30574400 -2.25019000

C 1.81580200 -2.13157200 -0.42327200

C -1.25090400 -1.74307400 -1.56608800

H -0.44784800 -3.68413000 -1.28570500

Ni 1.00967900 -0.43249300 0.11926200

H 2.89479400 -1.92676100 -0.54623300

C 1.69802000 -3.33052500 0.51896600

H 2.16911200 -3.12169600 1.48580700

H 0.65646100 -3.58911600 0.73102400

H 2.18713800 -4.22457600 0.09023500

O -2.42960700 -2.12420700 -1.30719900

N 2.43270600 0.54726200 -0.96559700

H 1.99714000 1.39120200 -1.34358400

H 2.71100300 -0.01325600 -1.77336100

C 3.63590100 0.92079700 -0.19451300

H 3.31954000 1.52798400 0.65804400

H 4.08617100 0.00306800 0.19470300

C 4.65756600 1.69115800 -1.02332400

H 4.97254700 1.07637700 -1.88284800

H 4.19076500 2.60703600 -1.42257200

O 5.75356000 1.99346100 -0.16477600

H 6.40864300 2.48385700 -0.68802300

Br -0.18784500 -1.16060500 2.18618800

K -3.15551400 -1.11110600 0.98000800

**Int2-1**

C 2.73490300 0.63422600 0.95930200

C 4.03201800 0.52442500 0.46031000

C 2.02811900 -0.58390900 1.29185500

H 4.57675500 1.43825200 0.23533700

C 4.63711600 -0.73273500 0.21657700

C 2.64301900 -1.85001700 1.01803700

H 5.64483000 -0.76406300 -0.18973300

C 3.95355200 -1.90526700 0.46901500

H 4.40311800 -2.87384000 0.26648100

C 1.89077100 -3.02063800 1.30895600

N 0.78775500 -0.48980800 1.85349900

C 0.13399300 -1.60384300 2.12232000

C 0.63473700 -2.90222100 1.85400400

H 2.32642100 -3.99522300 1.10143300

H -0.84678100 -1.49106600 2.57994700

H 0.03306000 -3.77438300 2.09122100

N 2.16619700 1.88747300 1.17731500

C 0.42441200 3.53720500 0.89199000

H 1.09473100 4.34719000 0.57316100

C -0.95641800 3.71411200 0.25906700

H -0.84262900 3.87730600 -0.82035500

H -1.40416500 4.63581800 0.66901800

C -1.89545900 2.52722900 0.49174500

C 1.08213000 2.21397300 0.53131100

H 0.36788200 3.60515100 1.98437800

Ni -1.25668500 0.96607800 -0.45969900

H -2.86909000 2.77563100 0.03543900

C -2.11365700 2.21286900 1.97382500

H -2.90022600 1.46219600 2.11367400

H -1.20538500 1.82043100 2.44700600

H -2.41680600 3.11013200 2.54116200

O 0.57274700 1.51606400 -0.45264900

N -3.02666400 0.15227200 -0.35942900

H -3.29222600 -0.11573800 -1.31089500

H -3.74491600 0.80476500 -0.03703600

C -3.05203200 -1.05655700 0.50089300

H -2.26841300 -1.73062500 0.14978500

H -2.80219900 -0.74449000 1.51897200

C -4.40632500 -1.76609800 0.48422400

H -5.20054600 -1.06929700 0.79920900

H -4.64037600 -2.09647900 -0.53457700

O -4.38857300 -2.94058500 1.29008700

H -4.27188200 -2.65405100 2.21218200

Br -0.72863900 -0.99059900 -2.25516000

K 2.17677600 0.10227700 -2.15912800

**Int3**

C -2.03485000 -0.29369900 0.81075200

C -3.43115100 -0.35330800 0.74074100

C -1.45439900 0.95018000 1.22843900

H -3.91606200 -1.26735200 0.42378100

C -4.22462500 0.76427300 1.08959700

C -2.25885700 2.06438200 1.60071500

H -5.30531600 0.66727600 1.02077800

C -3.67074600 1.95433800 1.51498800

H -4.29004900 2.80481600 1.78477100

C -1.58446700 3.23110800 2.04373900

N -0.08785100 0.99678300 1.25621200

C 0.50724900 2.09904500 1.69429000

C -0.20857200 3.24255800 2.10458900

H -2.16509000 4.10171700 2.33738200

H 1.59201800 2.09823800 1.72607000

H 0.33691700 4.11353800 2.45256800

N -1.11590000 -1.32747300 0.61532100

C -0.87378000 -3.79843500 0.58925400

H -0.77764800 -4.48938500 -0.25771000

C 0.44664800 -3.67426600 1.35065600

H 0.63492200 -4.61350900 1.88731600

H 0.34749300 -2.88751800 2.10892700

C -1.52988700 -2.52364000 0.07363600

H -1.63970000 -4.23457700 1.24874800

Ni 0.68692500 -0.62113200 0.35111900

C 1.35098400 -2.21177600 -0.55370100

H 2.27123600 -1.98990300 -1.11589600

H 0.62062200 -2.54466800 -1.31073600

O -2.44768900 -2.62545900 -0.76285500

N 2.32564000 0.30120600 -0.11833000

H 2.11940500 1.30023600 -0.19293300

H 2.56694100 0.03330800 -1.07364000

C 3.47103100 0.06152200 0.78359300

H 3.17099900 0.33639900 1.79930500

H 3.68640300 -1.01021400 0.77543100

C 4.71114600 0.84814200 0.37736200

H 5.00166400 0.57032700 -0.64932100

H 4.47965600 1.92622300 0.37777700

O 5.73348300 0.53119100 1.31680300

H 6.52819800 1.02880400 1.06327800

C 1.63190900 -3.35686100 0.42679700

H 2.50683600 -3.11099200 1.04517500

H 1.89727800 -4.27466500 -0.12708600

Br 0.43795500 1.72935400 -2.60885400

K -1.76076100 -0.46076900 -2.41063700

**Int4**

C -1.41325800 2.36927500 -0.94131200

C 0.06465500 2.07128900 -0.74183600

N 0.42619900 0.76774700 -0.84159700

C 1.77920100 0.44399700 -0.95573500

C 2.23422000 -0.72288100 -0.25396900

C 2.71192400 1.12492200 -1.74215100

C 3.57269200 -1.19885800 -0.40016100

C 4.03480500 0.65500500 -1.88068100

H 2.40549500 2.02579200 -2.26194700

C 1.72158200 -2.39962000 1.27351100

C 3.93413100 -2.36299900 0.32950800

C 4.46952700 -0.48840800 -1.23661900

H 4.72123000 1.20959000 -2.51587500

C 3.01064400 -2.96160500 1.15872600

H 4.94093700 -2.76066800 0.23108100

H 5.48857600 -0.84751300 -1.35089400

H 3.25822000 -3.84792700 1.73438900

N 1.33471900 -1.32590800 0.59118500

C -2.11735200 2.61341300 0.41488700

H 0.98082900 -2.82601500 1.94402800

H -1.88962700 1.78070300 1.09428400

H -1.89604600 1.54812300 -1.47468000

H -1.49686700 3.27133600 -1.55899900

H -1.69835600 3.52731600 0.85903300

Ni -0.57203400 -0.60996200 0.51238300

C -3.60634400 2.73947200 0.26035000

H -3.95307500 3.53642300 -0.40083700

C -4.50112400 1.93327000 0.84091100

H -5.57080200 2.05480900 0.68325500

H -4.19408900 1.12828600 1.50818900

H -1.11732600 -0.90941400 2.06479600

O 0.82464100 3.01797600 -0.40794200

N -2.41965700 -0.79721200 -0.52652400

H -2.23759200 -0.72928700 -1.52952700

H -3.02770300 -0.00604800 -0.30492700

C -3.15383300 -2.04520400 -0.25028200

H -2.51408100 -2.89012500 -0.52644000

H -3.32864800 -2.10259800 0.82864500

C -4.48263500 -2.13444400 -0.99266000

H -4.30252300 -2.07876900 -2.07942500

H -5.11126400 -1.27087200 -0.71637300

O -5.09757000 -3.36764600 -0.62802300

H -5.94226500 -3.42525400 -1.10387900

K 1.67071100 1.85033100 1.84003900

**Int5**

C -7.72238100 1.85194700 4.01250100

C -8.50855600 2.15431200 5.14190800

C -6.42706200 1.29366800 4.19870700

H -9.50506300 2.55760600 4.99895000

C -8.00815600 1.92294900 6.42840300

C -5.91082500 1.06403500 5.51610800

H -8.62390100 2.16796900 7.29116300

C -6.73146600 1.39270600 6.62145800

H -6.35204900 1.22217700 7.62664600

C -4.60043400 0.50406300 5.62825900

N -5.72395800 0.93855100 3.04993500

C -4.46259200 0.42086700 3.21167800

C -3.89064800 0.20236100 4.45572000

H -4.17217700 0.32707400 6.61095200

H -3.94023300 0.17315000 2.29200000

H -2.88867400 -0.21647900 4.50947200

N -8.11172100 2.07067800 2.67416900

C -9.76990100 2.09831000 0.84923800

H -10.56540100 2.84608800 0.98795100

C -8.71406600 2.64238600 -0.12787700

H -8.35538100 3.61406300 0.24334200

H -9.25094800 2.86114000 -1.07227300

C -7.51001000 1.71017900 -0.36479900

C -9.33911100 1.68442000 2.25621300

H -10.27032700 1.22270400 0.41832300

Ni -6.53644400 1.69260700 1.39658600

H -6.88259300 2.19597400 -1.13672300

C -7.94581800 0.34688200 -0.92471000

H -7.07701600 -0.26021000 -1.21509100

H -8.50380700 -0.24453200 -0.18410700

H -8.59898100 0.42388900 -1.81734100

O -10.14910200 0.99223900 2.93090600

N -5.57201100 3.57450500 1.26025000

H -5.71557400 4.09342400 2.12866400

H -6.00455300 4.12685700 0.51819300

C -4.12984400 3.41834000 1.00107400

H -3.70546800 2.81427600 1.80959000

H -4.01369500 2.85386800 0.06919200

C -3.38193600 4.74195100 0.89831400

H -3.81472600 5.34723800 0.08456700

H -3.50792700 5.30715900 1.83672000

O -2.01206600 4.43582000 0.65149600

H -1.53021500 5.27700200 0.59165200

K -8.48861800 -0.98527100 3.58159000

**Int6**

C -8.02382300 1.87037700 4.11467400

C -8.83958900 2.24027900 5.20049800

C -6.68633400 1.45821300 4.36216100

H -9.86725500 2.53333100 5.01420700

C -8.32621500 2.21939300 6.50307200

C -6.15411600 1.44861500 5.69251100

H -8.96561200 2.51440200 7.33225500

C -7.00683800 1.83833900 6.75293600

H -6.61782200 1.83478600 7.76885200

C -4.79676800 1.03279900 5.86028800

N -5.95597100 1.02147000 3.26022100

C -4.65103500 0.65213100 3.47184700

C -4.06066500 0.65233400 4.72702400

H -4.35465200 1.02158100 6.85264600

H -4.10797500 0.33455900 2.58609200

H -3.02426000 0.33772400 4.82242300

N -8.42512000 1.88164700 2.75990600

C -9.95500300 1.35393700 0.93370200

H -9.65274300 2.32160200 0.52216700

C -9.28230600 0.20029200 0.12884500

H -10.05250600 -0.42742100 -0.33916200

H -8.73874900 -0.45313500 0.82772800

C -9.58372500 1.27568600 2.40808800

H -11.04473500 1.28095300 0.86776700

Ni -6.74559200 1.68791400 1.54733300

C -7.24293300 1.70530100 -0.41835300

H -6.34450100 1.64948900 -1.06073500

H -7.63486100 2.72401500 -0.59181800

O -10.30044900 0.57676300 3.17389200

N -5.99432300 3.65274600 1.74802200

H -6.19637500 3.96277700 2.70095200

H -6.49573500 4.28655400 1.12367200

C -4.54562800 3.73313900 1.49312400

H -4.04664300 3.02279900 2.16027700

H -4.36479800 3.40822900 0.46295700

C -3.96916400 5.12775400 1.70499200

H -4.47483600 5.84186100 1.03401500

H -4.16450500 5.44961800 2.74149200

O -2.57178600 5.05440300 1.43453600

H -2.19982700 5.94062500 1.57462100

C -8.27705000 0.67920800 -0.93498700

H -7.75909200 -0.21460900 -1.31456400

H -8.84858800 1.07675200 -1.79252000

K -8.39946200 -1.07808700 4.03935000

**Int7**

C -1.66494900 0.51570100 -1.41757800

C -2.81992700 0.49866600 -2.20001900

C -1.57081800 1.51708700 -0.39631600

H -2.90186600 -0.22654900 -3.00260300

C -3.89752400 1.38099100 -1.94543300

C -2.67764800 2.37083600 -0.10469100

H -4.78003600 1.32854800 -2.57864900

C -3.85210000 2.28167600 -0.89761800

H -4.69151600 2.93635400 -0.67999100

C -2.53920900 3.27075400 0.98523900

N -0.38423500 1.60446700 0.28629400

C -0.29439600 2.46062200 1.29675300

C -1.35912300 3.29909900 1.69385600

H -3.36734600 3.92594400 1.24330600

H 0.67292300 2.51127800 1.78268500

H -1.22138700 3.96909600 2.53662900

N -0.58909200 -0.37429000 -1.53602600

C 0.23925600 -2.68267800 -1.68443600

H 1.18239800 -2.17712600 -1.88618000

C 0.35552500 -3.57754100 -0.42975500

H 0.89979300 -4.48654300 -0.71575500

H -0.65734300 -3.90285200 -0.16054400

C -0.89927400 -1.68578000 -1.57142000

H 0.00755400 -3.32054400 -2.54782900

Ni 1.12082100 0.61424100 -0.64375900

C 2.30183800 -3.74442700 1.19832800

H 2.87402500 -3.23195300 1.97970800

H 2.00953600 -4.73417800 1.57758800

H 2.97364300 -3.90375900 0.34572000

O -2.06238200 -2.17113600 -1.46183400

N 2.70968400 -0.15463000 -1.77860100

H 3.24655900 0.69362800 -1.97387100

H 2.32423200 -0.44982000 -2.67790900

C 3.66090000 -1.20267600 -1.32163700

H 3.08947300 -2.10712800 -1.10587000

H 4.38140500 -1.44181800 -2.11795900

C 4.44879100 -0.79750900 -0.07814300

H 5.14728200 -1.61228100 0.15017300

H 5.04653000 0.10006200 -0.30136200

O 3.65376900 -0.58932700 1.07837800

H 3.28956100 0.31872900 0.98647200

C 1.06759900 -2.92592500 0.79179600

H 1.42622700 -1.92895200 0.50041000

C 0.13619100 -2.73286500 2.00761900

H 0.74961600 -2.55160800 2.90250300

H -0.41408900 -3.66762600 2.19848000

C -0.84425500 -1.56486500 1.86328800

H -1.40933700 -1.67811700 0.92981400

H -0.26958900 -0.63684100 1.74536200

C -1.81767400 -1.41899800 3.03921400

H -2.50144600 -2.28146900 3.06127600

H -1.25617600 -1.45514400 3.98346400

C -2.61687400 -0.11117600 2.99565300

H -3.36677700 -0.06536300 3.79420600

H -1.95355100 0.75470000 3.10111300

H -3.14465800 0.02714600 2.04079800

Br 2.94113100 2.46662400 0.18539500

K -4.14377900 -1.60986800 -0.11878700

**Int7-1**

C -0.87129300 2.40538500 0.31985500

C -1.83061100 3.34773400 0.68732000

C -1.04277800 1.72016400 -0.94117500

H -1.69568500 3.87917700 1.62608100

C -2.97461400 3.60395100 -0.10673500

C -2.21267200 1.97828000 -1.73095800

H -3.70151000 4.33634100 0.23497200

C -3.17828100 2.92402900 -1.29072600

H -4.06006700 3.10436000 -1.89990800

C -2.36201000 1.24827800 -2.94132400

N -0.09105400 0.82489800 -1.33771800

C -0.26231500 0.18485100 -2.48056200

C -1.39039100 0.35169600 -3.31978900

H -3.24318500 1.41714400 -3.55571800

H 0.52281300 -0.51414100 -2.76420400

H -1.46958700 -0.21788700 -4.24073200

N 0.23152600 2.17879400 1.14511900

C 1.53495900 0.78101800 2.65125500

H 1.10993700 0.58776500 3.64517400

C 2.41295300 -0.43301000 2.25848900

H 1.77785100 -1.16579400 1.73968700

H 2.74562300 -0.93348800 3.17755700

C 3.67196800 -0.16862800 1.40051900

C 0.33961800 1.02177100 1.74044000

H 2.13316400 1.69247800 2.73051400

Ni -0.42573200 -1.51043600 0.51804800

H 4.04825000 -1.17191500 1.14971700

C 4.77708500 0.52030000 2.21549200

H 5.68203900 0.66871200 1.61262500

H 4.46192400 1.50340800 2.58417400

H 5.05166900 -0.08745800 3.08705900

O -0.55782900 0.07236500 1.69718800

N 0.41391700 -2.82312800 -0.75705700

H -0.08143900 -2.74416500 -1.64871800

H 0.22036800 -3.77025400 -0.42271100

C 1.86619900 -2.66944300 -0.99389900

H 2.04211900 -1.65981500 -1.37134000

H 2.37314600 -2.75243700 -0.02619600

C 2.44081700 -3.68799000 -1.97754000

H 2.25895200 -4.71141400 -1.60924100

H 1.93806400 -3.59141900 -2.94716900

O 3.82592800 -3.45697300 -2.22270400

H 4.29473400 -3.61197600 -1.38473800

C 3.39899200 0.53427600 0.04481200

H 2.42394400 0.20230200 -0.33161600

H 4.14482100 0.17635300 -0.68094400

C 3.42630400 2.06980700 0.02645000

H 2.69405300 2.46670900 0.73639800

H 4.41304200 2.43485400 0.34310800

C 3.10948000 2.63834300 -1.36321700

H 2.14679100 2.23449900 -1.70315400

H 3.86438100 2.28942800 -2.08376900

C 3.04980900 4.16837200 -1.37705200

H 4.00167800 4.60797800 -1.05038400

H 2.83014300 4.55592600 -2.37992800

H 2.26660700 4.53324300 -0.69941900

Br -2.72078300 -2.53555900 0.52030900

K -3.23640000 0.41347700 1.54412900

**TS1**

C -1.22160300 -1.72436800 -2.53819300

C -0.78567100 -0.28856500 -2.29835400

N 0.01323400 -0.08056600 -1.21743500

C 0.64855400 1.15254200 -1.02065400

C 1.16405000 1.36601100 0.30188600

C 0.86707500 2.16766200 -1.96037600

C 1.84343700 2.56845500 0.65424100

C 1.55166000 3.35373200 -1.60817000

H 0.50315800 2.04561000 -2.97047800

C 1.43078800 0.50202100 2.44941200

C 2.31391800 2.68973600 1.98733100

C 2.02821500 3.57033500 -0.33233100

H 1.69869600 4.10985100 -2.37524400

C 2.10536900 1.66233000 2.87968400

H 2.83641000 3.59481600 2.28546900

H 2.54898900 4.48653400 -0.06914300

H 2.45092200 1.72307400 3.90634200

N 0.97608100 0.35275100 1.20778200

C -1.92880600 -2.33330500 -1.31803400

H 1.25673600 -0.32485700 3.12755400

H -2.65660000 -1.62392500 -0.90296400

H -0.34227800 -2.33342600 -2.79166900

H -1.88594600 -1.71539300 -3.40676200

H -2.49733400 -3.21439400 -1.65192800

Ni 0.19659400 -1.24023100 0.41110400

C -0.97590200 -2.77678600 -0.23423000

H -0.31328600 -3.59938400 -0.51571600

C -1.26178300 -2.60115600 1.12385800

H -0.94714400 -3.34001400 1.85631400

H -2.10103500 -1.97101100 1.42133900

H 0.29900200 -1.88668900 1.70965300

O -1.22146100 0.61210000 -3.05441500

N 2.14539800 -2.17555200 -0.34771400

H 2.04836500 -2.15529400 -1.36264700

H 2.18208600 -3.15867400 -0.07890000

C 3.38256300 -1.49517000 0.05360500

H 3.31203700 -0.44645100 -0.25331900

H 3.44468600 -1.50945800 1.14747500

C 4.64734600 -2.10952800 -0.53626700

H 4.58267600 -2.09333900 -1.63725500

H 4.72374000 -3.16488200 -0.22481900

O 5.75667500 -1.34535600 -0.06732900

H 6.56166700 -1.73492800 -0.44611600

Br -3.96677600 0.19442600 1.11920600

K -2.39991400 1.92314100 -0.88054700

**TS2**

C 1.77534000 3.48487500 0.69351800

C 2.17651900 2.02958600 0.43871800

N 1.61963400 1.10489900 1.22505500

C 1.96701800 -0.21147000 0.92774400

C 1.25949700 -0.93742400 -0.09797700

C 3.00853600 -0.87196400 1.57433000

C 1.68739300 -2.24956500 -0.48386500

C 3.40475800 -2.18228700 1.21963200

H 3.55079500 -0.33638100 2.34898200

C -0.41702000 -0.96494900 -1.72659300

C 1.01882100 -2.87795700 -1.56707900

C 2.77488500 -2.86115100 0.19569600

H 4.23553400 -2.64427800 1.74662000

C -0.01368700 -2.22878400 -2.20344900

H 1.33722200 -3.86822100 -1.88219800

H 3.09406900 -3.85480300 -0.10560800

H -0.53804600 -2.67434200 -3.04191700

N 0.17680200 -0.35072900 -0.70977800

C 0.35091100 3.69658400 1.21411200

H -1.26500900 -0.45201300 -2.16866700

H 0.21392100 3.15406600 2.15462300

H 1.91955700 4.02687900 -0.24796900

H 2.48648100 3.91409700 1.41297000

H 0.20744500 4.76628800 1.42794500

Ni -0.76524500 1.16068600 0.11318700

C -0.74582700 3.30302700 0.24790200

H -0.75246200 3.84292200 -0.69705700

C -1.92507800 2.70496500 0.69673000

H -2.82065800 2.72426900 0.07486800

H -2.09316700 2.60397300 1.76813300

H -0.07898600 2.03593200 -0.84484600

O 3.01190600 1.81916800 -0.48955500

O -2.05567500 -3.49665400 2.75644000

H -2.18506100 -3.96768000 1.91534400

C -2.43687300 -2.14112600 2.54344900

H -3.47563800 -2.06256300 2.18218900

H -2.38936800 -1.65184800 3.52321600

C -1.49245300 -1.44116000 1.56526600

H -1.52392100 -1.94375700 0.59427200

H -0.46830600 -1.49406300 1.94046600

N -1.83782300 -0.01430700 1.36247900

H -2.81146800 0.04733600 1.05226400

H -1.82388700 0.44489000 2.27610100

Br 4.59314600 -1.29866600 -4.61428800

K 4.09606900 -0.07357200 -1.84290000

**TS3**

C -3.58377400 0.26608200 -1.07447100

C -2.28824000 1.03555600 -0.82648600

N -1.17756900 0.31660300 -0.52937300

C 0.08748500 0.95569400 -0.50959200

C 0.98439200 0.46441000 0.46535800

C 0.52672100 1.94535900 -1.41940000

C 2.30514200 1.00599800 0.59452500

C 1.81312400 2.48321000 -1.29303500

H -0.14287600 2.29364600 -2.19730800

C 1.36811000 -1.07653900 2.22336000

C 3.16346100 0.42433600 1.57782100

C 2.68575200 2.04288500 -0.29087100

H 2.13902300 3.25160900 -1.99051400

C 2.65386800 -0.60746800 2.40008400

H 4.16887200 0.81241800 1.71549200

H 3.68663200 2.46064500 -0.21058100

H 3.27508700 -1.05184200 3.17328700

N 0.52600700 -0.57908300 1.25429900

C -3.90680000 -0.85621300 -0.08118100

H 0.96178500 -1.87901400 2.82841100

H -3.99163800 -0.44437900 0.93331500

H -3.51625900 -0.16278400 -2.08590900

H -4.39240600 1.00244600 -1.09346000

H -4.89470000 -1.27002100 -0.33759300

Ni -1.15985400 -1.22004400 0.65353900

C -2.87076300 -1.95471900 -0.10265700

H -2.64039200 -2.36245200 -1.08913900

C -2.57317500 -2.75260500 1.01751100

H -2.20889800 -3.76796000 0.87446300

H -3.12217900 -2.60287400 1.94685500

H -1.14124100 -2.30546700 1.67640400

O -2.31551700 2.28813300 -0.94425000

N 0.61996900 -2.52827100 -1.91926700

H 0.14547100 -1.63868800 -1.75724200

H 0.74310500 -2.59949200 -2.93078000

C 1.93694700 -2.48480700 -1.26708900

H 1.77379700 -2.31492600 -0.19761000

H 2.42009000 -3.46483400 -1.37147600

C 2.88145700 -1.39888400 -1.79881500

H 2.34694900 -0.43745100 -1.83382100

H 3.18565600 -1.64168300 -2.82561000

O 4.08045900 -1.29786600 -1.03469100

H 3.83331000 -0.90198600 -0.17738900

K -0.79757400 3.57082400 0.79750900

**TS4**

C -3.24837300 0.03907200 1.47937000

C -2.00785800 0.92558200 1.31903100

N -0.92542100 0.35845800 0.73830700

C 0.30859100 0.98753200 0.66558700

C 1.12963300 0.60353500 -0.45585300

C 0.85259800 1.87989300 1.59647300

C 2.46073600 1.09774900 -0.59050500

C 2.17752600 2.34428700 1.46753000

H 0.24798700 2.19479800 2.43776500

C 1.31285800 -0.61638500 -2.43793000

C 3.20824700 0.68149700 -1.72428900

C 2.98015300 1.96628300 0.40571600

H 2.57143900 3.01556600 2.22647100

C 2.63097900 -0.16539700 -2.64525300

H 4.22856300 1.03448700 -1.84686100

H 3.99821300 2.33296900 0.30955800

H 3.17501000 -0.50424800 -3.52103400

N 0.57629000 -0.25132200 -1.38728200

C -3.17034500 -1.40930100 0.97764700

H 0.84198800 -1.30532500 -3.13120100

H -2.30803400 -1.90023600 1.44521800

H -4.07555000 0.56710600 0.98487600

H -3.48999200 0.04685700 2.55083900

H -4.06644800 -1.94333200 1.32606800

Ni -1.24711500 -0.81971000 -0.95112200

C -3.07179400 -1.57468400 -0.54486700

H -4.03626700 -1.43833600 -1.03945200

C -2.20656600 -2.63812600 -1.06770000

H -2.52090900 -3.10570700 -2.00364300

H -1.81854000 -3.35572000 -0.34275500

H -2.70821900 -0.20206700 -1.10462100

O -2.10451400 2.13204800 1.65600800

O 3.91092600 -1.12737700 1.50014000

H 3.54250300 -0.23004000 1.42927100

C 2.93391700 -2.02474400 0.97903500

H 2.64923000 -1.75005600 -0.04892400

H 3.41172000 -3.00983700 0.93014600

C 1.68236900 -2.09850300 1.85516400

H 1.33078500 -1.07064900 2.04401400

H 1.95279700 -2.52942200 2.82700800

N 0.67171700 -2.95551600 1.21841000

H -0.12721500 -3.05249700 1.84475400

H 0.29839900 -2.47261700 0.39865200

K -1.69183400 2.81725700 -0.96045000

**TS5**

Ni -1.67464000 -0.51825100 0.27448000

C 2.44445200 1.91400700 -0.98882300

H 2.73249100 2.89055400 -1.37300800

H 1.91452600 1.95114000 -0.04606200

C 3.45551700 0.82064900 -1.16254700

H 3.66993500 0.66590400 -2.23018400

H 3.06635500 -0.11692300 -0.74577200

C 4.78774900 1.13569700 -0.44628900

H 5.19686900 2.07329900 -0.84537700

H 4.58783900 1.30231400 0.61892900

Br 0.62278000 1.48618500 -2.40217700

C 0.84102400 -1.75344900 0.78290500

C 1.94580700 -2.62564500 0.87122400

C 0.96868300 -0.45058900 1.34794400

H 1.87203900 -3.62469300 0.46447800

C 3.13845000 -2.21214400 1.48674700

C 2.18845000 -0.03161700 1.97112200

H 3.97005000 -2.91133600 1.53811600

C 3.27182400 -0.93984400 2.02927600

H 4.20235300 -0.62489700 2.49431100

C 2.24534900 1.29456600 2.48920200

N -0.13157800 0.37391800 1.27127500

C -0.05021800 1.60735900 1.82567600

C 1.09929900 2.09374200 2.43843900

H 3.15863200 1.64569000 2.96105400

H -0.93999500 2.22454900 1.77138200

H 1.09822600 3.09892000 2.85016800

N -0.43976000 -2.07044400 0.25863200

C -1.99037700 -3.70584800 -0.77789700

H -1.95154300 -4.72433700 -0.36557300

C -3.16338000 -2.93182900 -0.18421900

H -3.14100400 -3.01261800 0.91114900

H -4.09415700 -3.42856800 -0.51313600

C -3.16325100 -1.45419000 -0.58289800

C -0.59454600 -3.13425900 -0.57409100

H -2.11866500 -3.83591500 -1.85933800

H -4.08679500 -1.00141800 -0.18232500

C -3.16345900 -1.25245200 -2.10461600

H -3.26622400 -0.19314900 -2.37415400

H -2.23232900 -1.60340600 -2.56836100

H -3.99117500 -1.79319600 -2.59723500

O 0.34172300 -3.70766400 -1.19095300

N -2.71781400 1.17014000 0.14248300

H -3.28313400 1.15504100 -0.70873100

H -2.05767500 1.94098800 0.01196000

C -3.60424500 1.45993400 1.29384300

H -4.33438500 0.64974600 1.36710300

H -3.00188100 1.45116100 2.20595000

C -4.32121400 2.79757700 1.15621100

H -3.57729700 3.60755800 1.07612800

H -4.91811800 2.80102700 0.22905000

O -5.14189900 2.95175700 2.30964900

H -5.59860900 3.80545800 2.23169300

C 5.80531600 0.00419200 -0.61663700

H 6.03911800 -0.16183700 -1.67628300

H 6.74366600 0.23363700 -0.09726800

H 5.41467700 -0.93553500 -0.20662400

K 1.45306700 -1.66272500 -2.43922300

**TS6**

C 3.22530500 0.16412600 1.23085600

C 3.97050700 1.30677100 1.52958500

C 3.50653100 -0.50829800 -0.02015200

H 3.76807900 1.81596500 2.46828100

C 4.94759400 1.82470200 0.65003400

C 4.49299700 0.03610800 -0.91324300

H 5.48624600 2.72639300 0.93061300

C 5.20931200 1.21094400 -0.55906600

H 5.94796000 1.61085200 -1.24875100

C 4.67967600 -0.61633600 -2.16338300

N 2.79253700 -1.63025000 -0.33733500

C 3.00359100 -2.20290100 -1.50777300

C 3.93258300 -1.72868800 -2.46824200

H 5.41301300 -0.22016100 -2.86189200

H 2.41621200 -3.09305700 -1.72509900

H 4.04633300 -2.24819600 -3.41482000

N 2.28527100 -0.30430300 2.14163100

C 0.05459300 -0.78854200 2.91241100

H 0.50756400 -0.61407000 3.89423900

C -1.35148200 -0.17540100 2.87231800

H -1.25559000 0.91336800 2.78228100

H -1.82662400 -0.36027700 3.84950200

C -2.30998000 -0.71723100 1.79328400

C 1.00513800 -0.23064400 1.85549600

H -0.00179800 -1.87719100 2.79427400

Ni -1.49032800 0.37631400 0.19363800

H -3.11830800 0.00630100 1.62244200

C -2.98998200 -2.00312600 2.26278100

H -3.68780200 -2.41005000 1.52398000

H -2.27788800 -2.79006600 2.53040500

H -3.57072800 -1.77873300 3.16958100

O 0.50819800 0.29812200 0.79044200

N -3.35621900 0.81097400 -0.84031100

H -3.07522300 1.25364700 -1.71657300

H -3.74675000 1.57227700 -0.28226900

C -4.40033200 -0.19425100 -1.10528600

H -3.98956500 -0.95186600 -1.77958500

H -4.64223200 -0.69064500 -0.15781100

C -5.67215900 0.39539400 -1.71808100

H -6.07761100 1.17577400 -1.05218200

H -5.43565700 0.87176300 -2.67698100

O -6.64556500 -0.60664800 -2.00206300

H -6.92755200 -0.98083300 -1.14983900

C -1.62478000 -1.61749900 -0.05073900

H -1.29521000 -1.13125500 -1.00422700

H -2.61135800 -2.03507800 -0.24557300

C -0.60897100 -2.70128100 0.27578800

H 0.37542100 -2.25653800 0.43655500

H -0.88069800 -3.23689400 1.19174600

C -0.49065600 -3.73323400 -0.86114800

H -0.11964300 -3.23206700 -1.76681800

H -1.48659900 -4.12771700 -1.11007200

C 0.44072100 -4.89296900 -0.49307100

H 0.04165000 -5.46054200 0.35771500

H 0.56836400 -5.59073600 -1.33006900

H 1.43396600 -4.52615100 -0.20526000

Br -1.14901400 2.99210100 -0.13598900

K 1.62202000 1.79893300 -1.08383500

***n*BuBr**

C 3.29586400 -0.54508100 -0.14366400

H 3.25660200 -0.81635000 -1.20661100

H 3.92937200 0.34659500 -0.05152700

H 3.79083400 -1.36409200 0.39194800

C 1.89247900 -0.28754200 0.41053000

H 1.29012400 -1.20141000 0.33270600

H 1.95364600 -0.04375400 1.48062000

C 1.17692700 0.85135000 -0.32195800

H 1.77469300 1.77223600 -0.23552400

H 1.10237100 0.62673400 -1.39357900

C -0.19703300 1.19751300 0.22436300

H -0.64287600 2.05301100 -0.28395300

H -0.18628600 1.37468200 1.30135400

Br -1.52222600 -0.28700300 -0.03860200

***n*Bu·**

C -1.88802500 -0.13228400 0.01926900

H -2.03043700 -0.86524100 -0.78554900

H -2.01322100 -0.65968900 0.97396900

H -2.69096000 0.61076300 -0.06017500

C -0.50842900 0.52433000 -0.07076300

H -0.41762600 1.07035200 -1.01980700

H -0.40127000 1.27197600 0.72801800

C 0.64258100 -0.49087200 0.03732400

H 0.51730500 -1.05368700 0.98371900

H 0.54524800 -1.24631400 -0.75711000

C 1.99648100 0.13174600 -0.01266100

H 2.16852800 1.11295000 0.42463000

H 2.86678000 -0.43862900 -0.32670900
